# Host cell transcriptional profiling during malaria liver stage infection reveals a coordinated and sequential set of biological events

**DOI:** 10.1186/1471-2164-10-270

**Published:** 2009-06-17

**Authors:** Sónia S Albuquerque, Céline Carret, Ana Rita Grosso, Alice S Tarun, Xinxia Peng, Stefan HI Kappe, Miguel Prudêncio, Maria M Mota

**Affiliations:** 1 Unidade de Malária, Instituto de Medicina Molecular, Faculdade de Medicina da Universidade de Lisboa, 1649-028 Lisboa, Portugal; 2 Unidade de Biologia Celular, Instituto de Medicina Molecular, Faculdade de Medicina da Universidade de Lisboa, 1649-028 Lisboa, Portugal; 3 Seattle Biomedical Research Institute, Seattle, Washington 98109, USA; 4 Instituto Gulbenkian de Ciência, 2780-156 Oeiras, Portugal

## Abstract

**Background:**

*Plasmodium *sporozoites migrate to the liver where they traverse several hepatocytes before invading the one inside which they will develop and multiply into thousands of merozoites. Although this constitutes an essential step of malaria infection, the requirements of *Plasmodium *parasites in liver cells and how they use the host cell for their own survival and development are poorly understood.

**Results:**

To gain new insights into the molecular host-parasite interactions that take place during malaria liver infection, we have used high-throughput microarray technology to determine the transcriptional profile of *P. berghei*-infected hepatoma cells. The data analysis shows differential expression patterns for 1064 host genes starting at 6 h and up to 24 h post infection, with the largest proportion correlating specifically with the early stages of the infection process. A considerable proportion of those genes were also found to be modulated in liver cells collected from *P. yoelii-*infected mice 24 and 40 h after infection, strengthening the data obtained with the *in vitro *model and highlighting genes and pathways involved in the host response to rodent *Plasmodium *parasites.

**Conclusion:**

Our data reveal that host cell infection by *Plasmodium *sporozoites leads to a coordinated and sequential set of biological events, ranging from the initial stage of stress response up to the engagement of host metabolic processes and the maintenance of cell viability throughout infection.

## Background

Malaria remains a major health problem worldwide with 35% of the human population being at risk of becoming infected [1]. The disease is caused by parasitic protozoa of the genus *Plasmodium*, which reach their mammalian host through the bite of an infected female *Anopheles *mosquito. The biological events that occur between the bite of a malaria-infected mosquito and the release of *Plasmodium *merozoites into the bloodstream are obligatory steps in the establishment of a malaria infection. Recently, our knowledge of the initial steps occurring in the skin immediately after mosquito bite increased (reviewed in [2]). We now also have a greater understanding not only of how sporozoites traverse the liver sinusoids and reach the hepatocytes before invading a final cell and forming a parasitophorous vacuole [3,4], but also of how, inside hepatocytes, the so-called exoerythrocytic forms (EEFs) of the *Plasmodium *parasite develop and multiply. There, EEFs grow in size and undergo a series of morphological changes that culminates in the release of several thousand merozoites into the blood, where they will invade erythrocytes and cause the symptoms of the disease (reviewed in [5]). This gain of knowledge was only possible due to recent advances in imaging technologies [6], together with the development of tools for the genetic manipulation of *Plasmodium *species [7]. However, in spite of these and other [8,9] recent achievements little is known about the host molecules involved in *Plasmodium *liver development. This gap in our understanding is mainly due to the fact that the proportion of infected hepatic cells is very low both *in vitro *and *in vivo*. The reasons why hepatocytes are able to provide an appropriate environment for sporozoite development remain generally unknown. Given its clinically silent nature, the hepatic stage of *Plasmodium *life cycle constitutes an ideal target for potential anti-malarial vaccines or prophylactic treatments [10]. Therefore, it is important to identify host hepatic factors that may influence liver infection and to understand their underlying molecular mechanisms, as a means to impair the parasite's development in the liver. Furthermore, an understanding of the molecular processes at play during the liver stage of malaria, may provide important information regarding the establishment and activation of hypnozoites, dormant forms of the parasite that occur with certain *Plasmodium *species and that are capable of causing a subsequent relapse in infection.

We took a genome-wide microarray approach to look into the liver-parasite molecular interactions and obtain a time-dependent profile of the transcriptional landscape of murine hepatoma cells infected by *Plasmodium berghei *sporozoites. Hepatoma cell lines constitute an invaluable and widely used tool to study the molecular aspects of hepatic infection by *Plasmodium *[11]. This transcriptional analysis of the host cell response to *Plasmodium *infection and development revealed a coordinated and sequential set of biological events. We have identified several host genes and pathways with clearly modulated expression profiles as a result of infection and, thus, constitute a repertoire of novel host factors that are prime candidates for intervention strategies.

## Results and discussion

### Cell infection for microarray analysis

**Figure 1:**
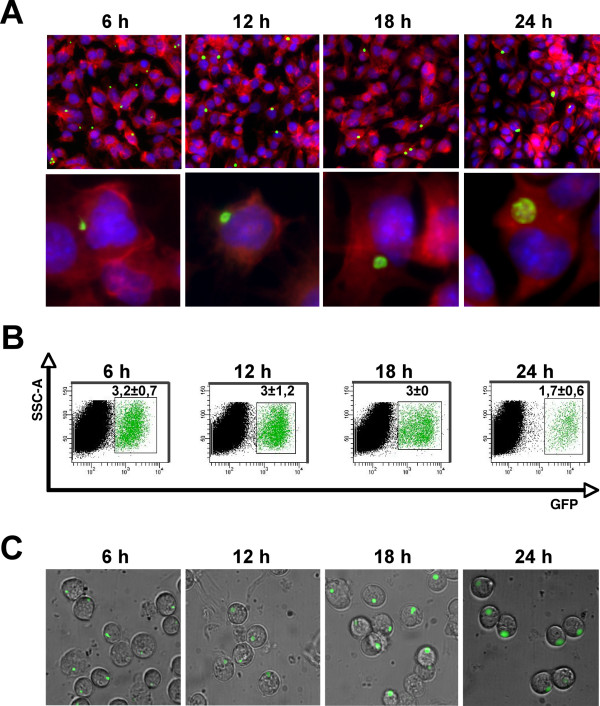
**Isolation of GFP-expressing *P. berghei *ANKA sporozoites using FACS at 6, 12, 18 and 24 h p.i**. (A) Pre-sorting images of infected cell populations (top panel, bar = 60 μm) or isolated cells (bottom panel, bar = 10 μm) at the respective time points p.i. (B) FACS plot of sorting experiments at the respective time points post-infection with the average reading obtained from 3 or more independent samples with results expressed as mean ± SD of green fluorescence. (C) Post-sorting images of infected cells.

To understand the extent of host gene modulation by the murine model malaria parasite *P. berghei *ANKA, a mouse hepatoma cell line, Hepa1-6, was infected with sporozoites obtained from *Anopheles stephensi *salivary glands, freshly dissected as previously described [12]. *Plasmodium *sporozoite infection *in vitro *shows very low efficiency ([11] and Figure 1A, top panel, parasites are green), with an average of 2.7 ± 0.7% infected cells (n = 8) in our chosen model. To overcome the "dilution effect" of infected cells by non-infected ones, Hepa1-6 cells were infected with GFP-expressing *P. berghei *ANKA parasites [13] and infected cells were purified by Fluorescence Activated Cell Sorting (FACS) at 6, 12, 18 and 24 h post-infection (p.i., Figure 1B) to capture as many parasite-induced cellular events as possible: at 6 h p.i. the parasite starts to change its elongated sporozoite shape into a rounder form, which grows in size (12 h p.i) and starts multiplying, with several nuclei present at 18 h p.i.; by 24 h p.i. sporozoite specific organelles are no longer visible and the parasite is bigger and round-shaped (Figure 1A, bottom panel, parasites are green). The viability of sorted infected and non-infected cells was confirmed by microscopic observation of cells seeded on coverslips immediately after sorting and allowed to adhere for at least 2 hours (Figure 1C).

### Global gene expression analysis of hepatoma cells during *Plasmodium *infection

Expression profiling of *P. berghei *ANKA-infected and non-infected Hepa1-6 cells was performed at the four developmental time-points selected, using the Mouse Genome 430 2.0 Array (Affymetrix, Santa Clara, CA). To exclude potential false positives due to cross-hybridization of parasite RNA with mouse probes, cRNA obtained from axenic cultures of *P. berghei *sporozoites [14] at the same selected time points was pooled and hybridized as an extra sample on the Mouse 430 2.0 array. No cross-hybridization was found in the list of genes differentially expressed (DE) in *P. berghei*-infected cells with the parasite cRNA control array (data not shown).

The experimental design aimed at two complementary goals: (i) identifying host genes DE in *Plasmodium *infected compared to non-infected hepatoma cells, and (ii) following the patterns of differential expression across infection and parasite development within the cell. After robust normalization ~55% of the 45000 probesets represented on the array were classified as present by the MAS5.0 software (Affymetrix). Empirical Bayes moderated *t*-statistics ("limma" software package of R/Bioconductor [15]) were used to detect up- and down-regulated transcripts in response to *Plasmodium *infection at any of the four selected time-points. One thousand one hundred and seven probesets (or transcripts) representing 1064 unique genes proved to be significantly DE in at least one time point during the course of Hepa1-6 cells infection by GFP^+ ^*P. berghei *ANKA (see Additional File 1). This set of data was used in all subsequent analyses.

**Figure 2:**
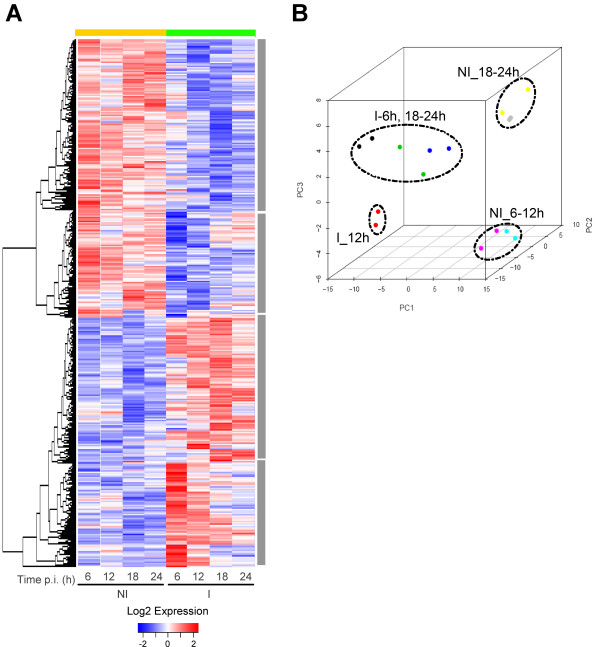
**Transcription profile analysis of the significant differentially expressed genes in *P. berghei *infected vs. non infected hepatoma cells**. (A) Hierarchical clustering using Euclidean distances for both samples (column, expressed as mean of replicates) and genes (probesets) showing differential expression (B stat > 0, FC >1.5×). (B) Principal component analysis (PCA) of transcript profiles from infected and non-infected Hepa1-6 cells. The plot shows simplified dataset structure of the significant differentially expressed probesets and clearly groups the samples in a 3-dimensional space between infected and non-infected cells but also along *Plasmodium *developmental time. Black, red, green and blue dots represent infected cells at 6, 12, 18 and 24 h p.i. respectively, while turquoise, pink, yellow and grey dots represent non-infected cells at 6, 12, 18 and 24 h p.i.

We used the expression values of each of the 1107 transcripts for hierarchical clustering where each time point was plotted for non-infected and infected samples (Figure 2A). Different profiles can clearly be identified between cells that either have or have not been infected with sporozoites (Figure 2A, green and yellow bars, respectively). Two main clusters separate genes that are highly expressed as a result of malaria infection (Figure 2A, red bars), while their expression in non-infected cells is relatively low (Figure 2A, blue bars), and vice versa. In addition, within the two large clusters, it is possible to identify smaller groups of probesets displaying an increasing (or decreasing) signal throughout time within both the infected and the non-infected cell categories (Figure 2A, grey shaded side bars). These changes indicate not only that malaria parasites modify the host cell transcriptome during the course of infection but also that they manipulate it in a timely fashion, presumably to fit their developmental needs.

In order to evaluate the overall structure of the data, we have plotted the first three principal components of a principal component analysis (PCA), capturing the overall variance of the samples in 3 dimensions (Figure 2B). This analysis clearly separates the data into 4 subgroups, clustering together the biological replicates, segregating infected from non-infected cells, and separating the samples by time after infection. Intriguingly, PCA separates genes expressed at 12 h p.i. in infected cells (Figure 2B, red dots) from those expressed at 6, 18 and 24 h p.i. (Figure 2B, black, green and blue dots, respectively). In non-infected cells, early to mid-term cellular events (6–12 h p.i, turquoise and pink dots, respectively) can be differentiated from later ones (18–24 h p.i., yellow and grey dots, respectively).

**Figure 3:**
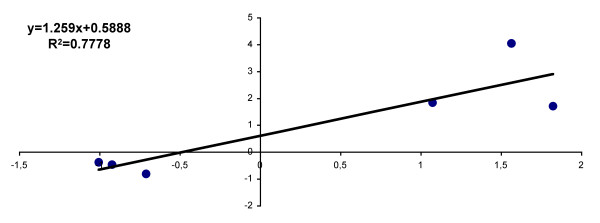
**Quantification of gene expression of *P. berghei *infected hepatocytes. Relation between Microarray and qRT-PCR Fold change values for 6 genes**. The qRT-PCR fold-changes were normalised using the expression of a housekeeping gene (*hprt1*) and compared with those obtained from non-infected cells.

To validate the microarray results, we analyzed 6 transcripts by quantitative RT-PCR (qRT-PCR), selected for their top ranking positions on the DE gene list at 6 h p.i. (see Additional File 1) or for their known function in the cell. Correlation analysis was performed by comparing expression ratios from microarray results with the ratios determined by the qRT-PCR analysis (Figure 3). Although the fold changes were under-estimated in the microarray results, a significant correlation was observed between the two assays (Pearson correlation coefficient r = 0.88, R^2 ^= 0.78).

**Figure 4:**
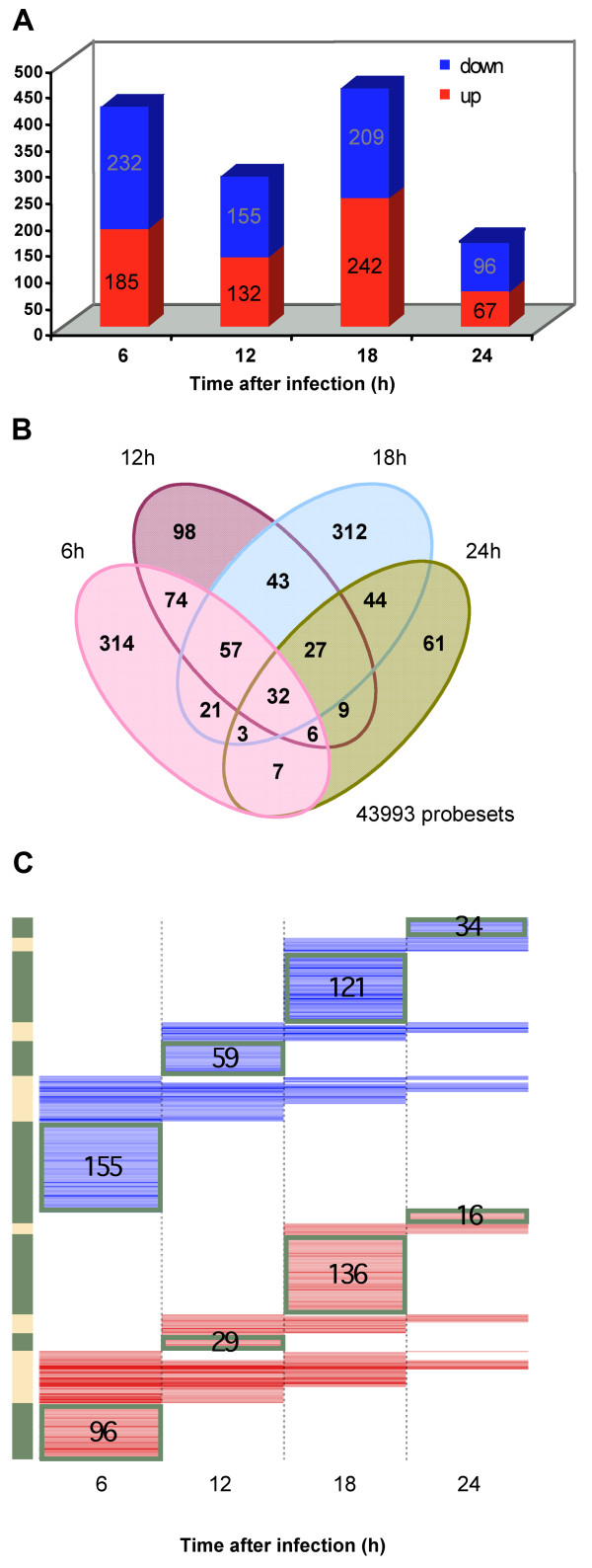
**Over- and under-expressed host genes across time in infected cells compared to non-infected ones**. (A) Venn diagram showing the repartition of the differentially expressed host transcripts at the 4 time points in malaria infected vs. non infected cells. The intersections show the number of probesets present in more than 1 time point. The numbers within separated ellipses correspond to differentially expressed genes that are unique to each time point. (B) Number of genes DE for each time point after normalisation and linear model fitting. Genes with more than one DE probeset presenting a consistent fold change were counted. Up- and down-regulated genes are shown in red and blue, respectively. The y-axis represents the number of differentially expressed genes (DEG). (C) Heatmap comparing the genes significantly altered between *P. berghei *infected cells vs. non infected ones. Each row of the plot is a gene and was coloured according to the log2 ratio of expression, with red meaning up-regulation in infected cells relative to the non-infected ones and blue meaning down-regulation. The heatmaps were generated using the heatmap.2 function of the "gplots" package in R. The dendrograms were generated using Euclidean distance and "complete" agglomeration method. The 3D PCA was generated using "scatterplot3d" package in R.

Overall, these results show more under-expressed than up-regulated genes in infected hepatoma cells (Figure 4A, blue stacked bars); seven hundred and eighty five transcripts are uniquely DE at a single time point while 323 probesets are so in at least two time points (Figure 4B). We also find that the number of DE host genes steadily decreases, from 417 at 6 h p.i. to 163 at 24 h p.i. (Figure 4A). These data suggest that host responses to parasite infection are more prominent at early time-points, causing more profound changes in gene expression during the initial stages of infection than during subsequent developmental processes occurring within the study's timeframe. In summary, our global analysis indicates that infection by *Plasmodium *leads to a sequence of cellular events that involves both discrete and sustained expression modulation responses in the host cell, as well as responses that are controlled in a temporal fashion during parasite development (Figure 4C).

### Time-course independent host cell responses to *Plasmodium *infection

Interestingly, 24 genes (32 transcripts) playing a role in transcription/translation, transport, signaling, apoptosis, cell growth and differentiation, proteolysis, cytoskeleton, and steroid biosynthesis are DE at all times in infected cells compared to non-infected ones (Figure 4B and see Additional File 2). Although no common pathway or gene ontology can be clearly identified for the whole set, 14 genes are up-regulated in all the time points assessed. They include three endoplasmic reticulum (ER)-stress related genes: *trib3*, *nupr1 *and *ddit3 *that can act in concert to induce apoptosis under the right stimuli [16] as shown during Hepatitis C virus infection [17]. As a matter of fact two of these genes, *trib3 *and *nupr1*, systematically ranked in the top 5 of genes DE throughout parasite development (see Additional File 3). However, the same ER-stress induced complex, involving Atf4, which binds to Ddit3 through a DNAzip binding domain, has been shown to regulate the heme oxygenase 1 (*hmox-1*) gene expression as a defense mechanism against oxidative stress [18]. Interestingly, *atf4 *is also up-regulated in our analysis from 6 to 18 h p.i, as is *hmox-1 *(top ranking at 18 h p.i; see Additional Files 4 and 1), suggesting a clear definitive and sustained response to oxidative stress caused by the parasite (see Additional File 1). Those ER-stress induced factors are attractive candidates to stimulate Hmox-1 expression, which we have shown to promote *P. berghei *liver infection [19]. Trib3 has also a clear negative modulatory effect on the insulin signaling pathway in hepatocytes that is responsible for glucose homeostasis [20]. Whether these metabolic pathways are important for *Plasmodium *development inside the hepatocyte remains to be elucidated.

It is also compelling to find that some host molecules are down-regulated in infected cells throughout the entire period of the experiment. Among those 10 transcripts, there are: 2 G-protein coupled receptors, which have a role in signal transduction; *protein C*, which is a regulator of the blood coagulation cascade; *maf*, which encodes an oncoprotein promoting cell differentiation; and 2 solute carriers, MCT4 and ZIP8, which, if down-modulated, have been shown respectively to help restore calcium homeostasis [21] and impair mitochondrion function in response to TNFα [22]. *Mct4 *and *zip8 *rank in the top 5 DE genes in our analysis, along with an ABC transporter (*abcd2*) that is also down-regulated during the whole course of parasite development, and *trib3 *and *nupr1 *(see above) that are up-regulated at all the time points assessed (see Additional file 3 and 1).

### Time-course specific host cell responses to *Plasmodium *infection

**Figure 5:**
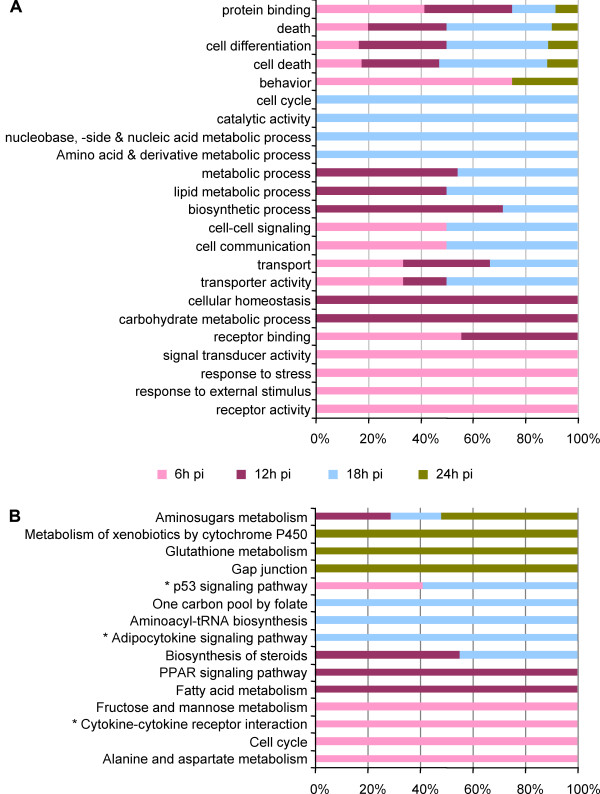
**Gene set enrichment analysis**. (A) Gene Ontology enrichment analysis (p < 0.01) based on GOslim. Genes found significant in the analysis were grouped by function following GO annotation. The stacked bars show the contribution of each time point across categories. Time points are colored as indicated on the graph. (B) KEGG metabolic pathway enrichment analysis calculated using the DEG having a KEGG annotation. Significantly enriched pathways are marked with a * (for p < 0.01).

To characterize all the DE transcripts biologically, an enrichment analysis of modulated genes by their functional annotation with Gene Ontology (GO) and KEGG metabolic pathways was performed at each time point (Figure 5A and 5B, respectively).

**Figure 6:**
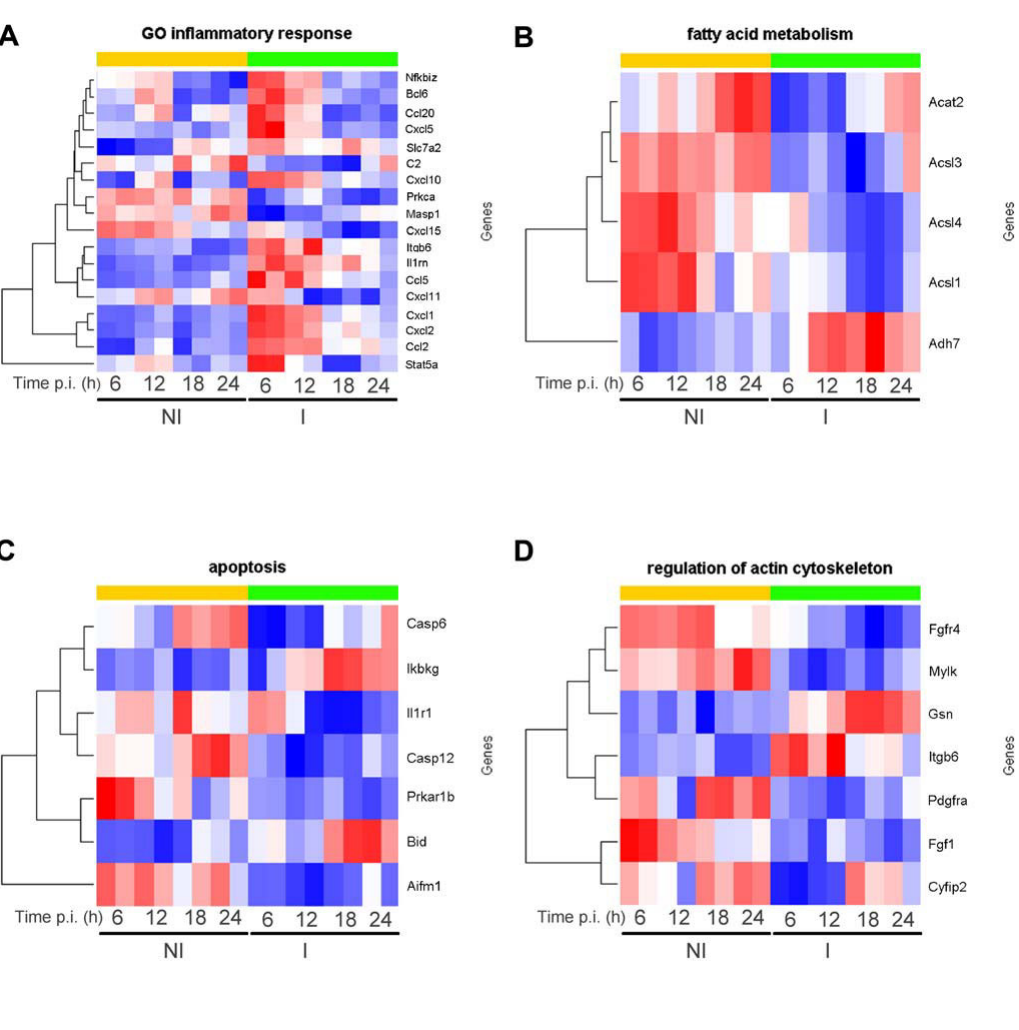
**Heatmaps of DE genes in *P. berghei *infected vs. non-infected cells**. for (A) Inflammatory response (GO:0006954), (B) Fatty acid metabolism (KEGG metabolic pathway mmu00071), (C) Apoptosis (KEGG metabolic pathway mmu04210), (D) Regulation of actin cytoskeleton (KEGG metabolic pathway mmu04810).

According to the GOslim analysis, most receptor binding activity, as well as stress and stimulus response-related genes, are modulated by the parasite in the early stages of infection. (Figure 5A, pink stacked bars). On the other hand, this analysis further shows that metabolic processes are then engaged at a later stage of the infection process. (Figure 5A, purple and blue stacked bars). The early responses are possibly mediated by cytokine and subsequent pro-inflammatory signaling events (Figure 6A, 6 and 12 h p.i in infected cells) that, in turn, induce fast cell cycling, as indicated by the KEGG metabolic pathways specifically enriched at 6 h p.i. (Figure 5B, pink stacked bars). This type of response is somewhat similar to that of human foreskin fibroblasts (HFF) infected by *Toxoplasma gondii *where both stress and cytokine/chemokine host genes are DE in the early stages of infection [23]. In *T. gondii *infection this response seems to be triggered by induction of *il-1β *and *tnf-α *and followed by the JAK/STAT1 signaling pathway [24]. However, in *P. berghei*-infected cells only TNF signaling is significantly modulated. Both *tnfaip2 *and *tnfaip3 *are up-regulated at 6 h p.i. and *tnf *receptors are down-regulated from 6 to 18 h p.i. (except for the anti-apoptotic receptor molecule *traf1*, which is up-regulated at 6 h p.i.). Neither *jak *nor *stat1 *are in our final list of DE genes (see Additional File 1) although *stat5a *is up-regulated at 6 h p.i. and *jak2 *is down-regulated at 12 and 18 h p.i., albeit not significantly. Thus,, it is not clear from the present experiment whether malaria parasites modulate the JAK/STAT1 pathway as happens during *Toxoplasma *infection [24].

Similarly to *T. gondii *[25,26], *P. berghei *parasites manipulate host cell NF-κB signaling. NF-κB transcription factors have been proposed to act as a "first responder" to the cellular stimuli caused by traversal of host cells by *Plasmodium *prior to invasion [27]. To identify targets or interacting molecules, we cross-checked the repertoire of cytokines and chemokines produced by infected liver cells with known target genes of NF-κB . *Ccl2*, a member of the CC subfamily of chemokines, is up-regulated throughout parasite development and *ccl5 *(*rantes*) is up-regulated between 6 and 12 h p.i (see Additional Files 1 and 5). *Cxcl1 *(*gro-α*), and *cxcl2 *are members of the CXC subfamily of chemokines that are also up-regulated, from 6 to 18 h p.i. or at all time points, respectively (see Additional Files 3, 1 and 5). Additional NF-κB-induced factors DE at early stages of infection include cell adhesion molecules *icam1*, and *vcam1*, as well as the transcription factors *nfkb1b *and *nfkb1z *(see Additional File 1).

Interestingly, it has been previously shown that *T. gondii *also manipulates the MAPK signaling pathway through LPS/TLR4 engagement [28]. Although in the present experiment none of the Toll-Like Receptors were DE, the MAPK pathway seems to be up-regulated through the production of *gadd45α/β *and *γ *from 6 to 24 h p.i. (see Additional File 4), *mkp *from 6 to 12 h p.i. and *ikk *from 18 to 24 h p.i. (see Additional Files 1 and 5). The MAPK pathway was also shown to be up-regulated in B-type hepatocellular carcinoma and during Hepatitis B Virus (HBV) infection [29,30], indicating that up-regulation of signal transduction-related genes is not pathogen-specific. This suggests that cell traversal or invasion by sporozoites may engage several host receptors and that these events constitute, perhaps not surprisingly, a stress-inducing stimulus for the cells, leading to a cascade of signaling events through chemokines and cytokines that might have positive or negative effects on the establishment of the liver infection. In fact, one such gene is *hmox-1*, which is up-regulated in infected cells at all the time points assessed, significantly so at 18 h p.i. (see Additional Files 4, 1 and 5), as well as in macrophages and Kupffer cells in the liver [19]. As mentioned above, Hmox-1 is a major stress-response molecule that we have shown to promote liver infection by controlling the extent of the host inflammatory response [19]. Interestingly, Hmox-1 has pronounced antiviral activity in HBV infection [31] while it can promote *Mycobacterium tuberculosis *latent infection in host [32].

At 12 h p.i., with 287 DE genes, the parasite seems to primarily modulate the host cell's transcriptome towards biosynthetic pathways including carbohydrate and fatty acid metabolism. This correlates well with host responses observed during early *T. gondii *infection that has been reported to be a general reaction to stress rather than a specific parasite induced response [23]. Genes involved in cellular homeostasis, transport and cell communication are also modulated following productive invasion.

At 18 h of parasite development 451 DE genes playing a role in lipid metabolism (35 genes), amino acid and nucleic acid metabolism (39 genes), as well as cell cycle (58 genes) and catalytic activity (33 genes) are significantly modulated (Figure 5; see Additional Files 1 and 5). This picture may suggest that once a host cell is successfully infected by *Plasmodium*, its resources remain engaged in energy production and homeostasis, possibly in an attempt to cope with the imbalance caused by the intracellular presence of the parasite. Indeed, the liver stage parasite goes through an extensive proliferative phase during this period, likely requiring large amounts of proteins and lipids to produce tens of thousands of merozoites that will in turn invade the red blood cells (reviewed in [5]). Consistent with this notion, our analysis shows that the parasite's presence down-modulates signaling trough the PPAR and adipocytokine pathways around 12 to 18 h p.i. (Figure 5B, purple and blue stacked bars) leading to increased lipid metabolic processes (Figure 5A, purple and blue stacked bars; Figure 6B; see Additional File 5). It has been described that a parasitophorous vacuole membrane protein encoded by the parasite, UIS3, specifically binds to the liver-fatty-acid binding protein (L-FABP) [33]. By engaging L-FABP, the parasite would reduce PPAR activity, resulting in an increase in fatty acids available to it. As such, it is tempting to speculate that the parasite influences host cell metabolism in its own interest. Also, transport molecules are engaged from 6 to 18 h p.i Whether this is important to provide the developing parasite with the components it requires for its rapid proliferation and/or host cell survival remains to be established (Figure 5A; see Additional File 5).

At 24 h p.i., with 163 DE genes, glutathione metabolism is the major host pathway modulated by the parasite (Figure 5B). Glutathione metabolism and transport participate in many cellular reactions including antioxidant defence of the cell, drug detoxification and cell signalling (involved in the regulation of gene expression, apoptosis and cell proliferation). Alterations in glutathione concentration have also been shown to be a common feature of many pathological conditions including diabetes, cancer, AIDS, neurodegenerative and liver diseases [34]. Therefore, one can speculate that the parasite stimulates the glutathione pathway to help sustain the host cell in the final stages of development.

Finally, it is interesting to note that genes involved in cell differentiation and death, through the p53 signalling pathway, are DE throughout the whole time span covered in this study (Figure 5A and 5B, pink, purple, blue and green stacked bars; see Additional Files 5). From the earliest stages of infection, cell survival and apoptosis mechanisms are actively at play (Figure 6C, mainly down-regulated transcripts in infected cells in early time points). Malaria hepatic infection involves the activation of the tyrosine kinase HGF receptor (MET) by secreted HGF [35] and down modulation of MET leads to a decrease in infection [8,35]. In our analysis, *met *is over-expressed at 6 h p.i (see Additional File 4). It has been shown that the malaria parasite exploits MET not as a primary binding site, but as a mediator of signals that make the host susceptible to infection. This signalling pathway might influence host actin cytoskeleton [35] and apoptosis [36] during infection (Figure 6D and 6C, respectively) and play an important role in the survival of the infected cell. Indeed, *P. berghei*-infected hepatocytes are more resistant to apoptosis induced by different stimuli throughout infection [36,37], and require an actively developing parasite [38]. Hence, one might therefore suggest that pro- and anti-apoptotic genes are actively modulated throughout infection, in a constant struggle between the infected cell's tendency to undergo apoptosis and the parasite's need for the host cell to survive (Figure 6A and 6C).

### Similarities between the rodent malaria species *P. yoelii *and *P. berghei*

A complementary *in vivo *assay was carried out on liver cells from mice infected with *P. yoelii *at 24 h, when most liver stage parasites are at a small early schizont stage and have undergone two to four rounds of nuclear division, and 40 h p.i., when most liver stage parasites are large late schizonts and have undergone up to 13 rounds of nuclear division [39], and compared to the findings described above. We found that 67% of genes DE in *P. berghei*-infected Hepa1-6 cells are also expressed in *P. yoelii*-infected livers. Although parasite development in cell culture and *in vivo *is known to proceed at different rates [40], and two different microarray platforms were used, for two different parasites species, a sizeable 42% of the genes DE in *P. berghei *infected cells are also found DE in *P. yoelii*-infected livers in at least one time point (see Additional File 6). Twenty-six transcripts are found DE in both experiments with the highest agreement in terms of up or down-modulation (16 transcripts) and common time point (10 transcripts at 24 h p.i.) (Grey shaded boxes in Additional Files 6 and 7). It is reassuring to find that some of those transcripts have been discussed above as identified in top ranking positions during *P. berghei *infection and include *ccl2*, *cxcl2*, *G protein-coupled receptor gpr137B*, and *gpr155*, *nr4a2*, *abcd2*, *hmox-1 *and *klf4*. These findings corroborate the validity and strength of our *in vitro *model, and highlight those genes or the pathways they are involved in as common and important factors in host response to rodent parasites.

## Conclusion

In summary, we have used a microarray-based approach to profile the host cell response to a malaria sporozoite infection in a time course experiment. Our data shows that host cell response to *Plasmodium *can broadly be divided into 3 major components, including an initial stress response to the presence of the parasite, a subsequent engagement of host cell metabolic processes and a constant management of host cell viability. Our data suggest that, following an initial stage in which the infected cells respond to the stress caused by the presence of the parasite, the outcome of infection may depend on the parasite's ability to engage the cell's resources to its own benefit thereby fulfilling its molecular needs for multiplication. On the other hand, *Plasmodium *seems to influence the host cell's survival for as long as the parasite requires an intracellular shelter for expansion. Targeting host factors during the initial stages of parasite development has already proven to modulate the fate of infection [19]. Our data reveal, for the first time, a number of host genes, from modulators of the immune system to genes involved in lipid metabolism, which may constitute possible targets for future drug development strategies. It is therefore tempting to speculate that those genes may constitute possible candidates for the design of new strategies for future anti-malarial intervention. Indeed, targeting host proteins specifically required for infection/maintenance of the parasite, but non-essential to the host itself, holds several key advantages. Most importantly it offers inherently lower susceptibility of those drugs to induce resistance in the parasite, arguably the biggest problem faced in all infectious disease fields today. Furthermore, targeting host factors also offers much higher potential for accelerated therapeutics discovery or development through synergies with ongoing output from mainstream disease pipelines. Host proteins are more likely to have been the focus of previous or even ongoing therapeutic development work in other fields, thus opening the possibility of finding anti-malarial activities as second medical uses for existing drugs.

## Methods

### Parasites and hepatoma cell culture

Parasites were maintained by alternate cyclic passages in mosquitoes and mice. GFP^+ ^*Plasmodium berghei *ANKA sporozoites were obtained from dissection of infected *Anopheles stephensi *mosquito salivary glands in RPMI 1640 (Gibco) medium. Hepa1-6 cells were cultured in DMEM supplemented with 10% Foetal Calf Serum (Gibco), 1% Penicillin/Streptomycin (Gibco) and 1 mM glutamine (Gibco) and maintained at 37°C with 5% CO2.

### Cell infection and sample collection

*In vitro *infections of Hepa1-6 cells for microarray experiments were carried out by adding 2 × 10^5 ^*P. berghei *sporozoites per well to cells seeded 24 h earlier in 24- well plates (2 × 10^5 ^cells/well). Salivary glands from non-infected mosquitoes were extracted and the same volume of the extraction solution was added to cells used as controls. All the *in vitro *infection plates were spun down at 3000 rpm for 5 min and incubated at 37°C with 5% CO2. Following incubation, the medium was aspirated, cells were washed with PBS and 100 μl Trypsin (Gibco) was added. After 3 min incubation at 37°C and 5% CO_2_, cells were washed with 10% FCS in PBS, collected and centrifuged at 800 rpm for 5 min at 4°C. The supernatant was removed and the pellet was resuspended in 2% FCS in PBS.

### Cell sorting and RNA extraction

Hepa1-6 cells infected with GFP-expressing *P. berghei *sporozoites [13] were collected at 6, 12, 18 and 24 hr post-infection and then selected using fluorescence activated cell-sorting (FACS) on a High speed cell sorter; Dako-Cytomation; Mo-Flo MLS leading to ~90% purity. Non-infected samples were collected using a similar protocol. After sorting, cells were collected in cell lysate (buffer RLT, Qiagen), vortexed for 1 min and stored at -80°C. Total RNA was extracted from the sorted cells with Rneasy MicroKit (Qiagen), according to the manufacturer's instructions. RNA concentration and quality of all replicates were determined on a NanoDrop ND-1000 UV-Vis Spectrophotometer (NanoDrop Technologies) and only samples with a 260/280 ratio greater than 1.8 were further processed. Each sample was diluted to be within the dynamic range of the RNA 6000 Nano Assay Kit (Agilent Technologies), with a target of 100 ng. The Nano Labchip protocol was followed according to the manufacturer's instructions and all samples were evaluated in a Bioanalyser 2100 (Agilent Technologies). Samples with the highest concentration, two distinct 18 S and 28 S subunit peaks and no evidence of degradation were further processed. For each time point two independent biological replicates were obtained.

### Hepatoma cell RNA hybridization

The GeneChip^® ^Mouse Expression 430 2.0 array contains 45000 probesets, covering 39000 transcripts and variants from over 34000 well characterized mouse genes. Duplicate cultures, infection processes and sorting events were analyzed for each condition (4 time points, 2 conditions, 2 replicates). Total RNA (100 ng) was used to generate biotin-labelled cRNA using the Two-cycle protocol as described by Affymetrix (Santa Clara, CA). Washing, staining and scanning of the DNA chips was performed as recommended by the manufacturer. Signal intensity for each feature on the arrays was determined using the 70th percentile method provided by GCOS software (Affymetrix). All microarray procedures were performed at the Instituto Gulbenkian de Ciência (Oeiras, Portugal) Affymetrix Core facility 144.

### Liver samples preparation

Total RNA from sorted liver stage-infected hepatocytes was isolated from PyGFP-infected BALB/c mice as described in [39]. RNA from liver stage-infected hepatocytes was isolated at two time points post-infection: 24 and 40 h p.i. As control, RNA from hepatocytes isolated from mock-infected mice was also isolated following the same procedure. Total RNA was then subjected to two rounds of linear amplification using the Amino Allyl Message Amp II aRNA Amplification Kit (Ambion) according to manufacturer's directions. The quality of total and amplified RNAs was examined with the RNA 6000 Nano Assay kit (Agilent Technologies) and the amount of RNA in the samples was assessed on a NanoDrop ND-1000 UV-Vis Spectrophotometer prior to microarray hybridization (NanoDrop Technologies). For each time point two independent biological replicates were obtained.

### Liver's microarray analysis

Customised 60-mer mouse microarrays were manufactured by NimbleGen (Madison, WI) and contain 188608 probes representing 32650 mouse transcripts. On average, each transcript is represented by 6 probes. To minimize the cross-hybridization with parasite RNAs, probes were screened against annotated genes (PlasmoDB version 5.0) and TIGR Gene Indices (Release 5.0) from both malaria parasites *P. yoelii *and *P. berghei*. To incorporate the latest annotation information, probes were re-mapped to Entrez Gene  prior to data analysis. Entrez Gene is NCBI's gene-centric database and each record represents a single gene from a given organism. Probes were mapped to mouse genes by blasting against mouse transcripts from NCBI's Reference Sequence (RefSeq) collection and all sequences in mouse UniGene clusters (all downloaded from NCBI on May, 2007). In total, 158685 probes were perfectly matched to 31473 mouse transcripts. 157412 probes were specifically mapped to 19203 mouse genes (8 probes per gene on average) and 1273 probes were specifically mapped to 366 transcripts which were not linked to an annotated mouse gene. Those un-mapped probes were excluded in this analysis. The possible reasons for not mapping those probes are either current annotation is not complete, or some of annotated sequence records retired after the array was designed.

### Data Analyses

Data analysis was performed using R/Bioconductor [15]. Microarrays from each platform were pre-processed separately using a Robust Multiarray Averaging program (RMA; [41]. The expression values were calculated using the "affycoretools" package package (MacDonald, JW. Affycoretools: Functions useful for those doing repetitive analyses with Affymetrix GeneChips. R package version 1.14.0). The differential expression was assessed and variability estimated by fitting a linear model to the data that fully models the systematic part of each gene, using the "limma" package [42]. By fitting a linear model to the data set, the "limma" package ranks significantly DE genes in infected vs. non-infected cells for each of the time points. From the "limma" output, log-fold changes from one condition compared with another and B-statistic (log-odds that a gene is differentially expressed) can be obtained and used to determine up- and down-regulation of genes. Fold changes of 1.5 × and B-statistics >0 for probesets were used as cut-off. Gene sets enrichment was analyzed using "GOstats" [43] and "GSEABase" packages (Morgan, M., Falcon, S. and Gentleman, R. GSEABase: Gene set enrichment data structures and methods. R package version 1.4.0). Heatmaps and PCA were plotted using the "gplots" and "scatterplot3d" packages (Warnes, GR. Includes R source code and/or documentation contributed by Bolker, B. and Lumley, T. gplots: Various R programming tools for plotting data. R package version 2.6.0; [44]

### Quantitative real time RT-PCR (qRT-PCR)

SYBR^® ^Green PCR Master Mix (Applied Biosystems) was used according to the manufacturer's instructions to quantify the expression of nuclear protein 1 (*nupr1*), tribbles homolog 3 (*trib3*), vascular cell adhesion molecule (*vcam1*), ATP-binding cassette, sub-family D (*abcd2*), cytochrome P450 1a1 (*cyp1a1*) and stearoyl-coenzyme A desaturase 2 (*scd2*). Gene expression was normalized against the expression of mouse hypoxanthine guanine phosphoribosyl transferase 1 (*hprt1*) as the housekeeping standard. Primers were designed using the Oligo 6 Primer Analysis Software (Molecular Biology Insights) and sequences were as presented in Additional File 8. Total RNA from the non-infected and *P. berghei*-infected hepatocytes (independent samples were collected at 6 h p.i., sorted and RNA extracted as described before) was reverse-transcribed to single strand cDNA using the AMV Reverse Transcriptase (Roche Applied Science) protocol. The single strand cDNA from the reverse transcriptase reaction was amplified by real-time quantitative fluorogenic PCR. The reaction was performed on an ABIPrism 7000 Sequence Detection System (Applied Biosystems) with a total volume of 25 μl using the following cycling parameters: one cycle of 2 min at 50°C, one cycle of 10 min at 95°C, 45 cycles of 15 s at 95°C, 1 min at 57°C. The reaction consisted on SYBR^® ^Green PCR Master Mix (12.5 μl), 0.8 μl of forward and reverse primer, 1 μl of template and 10.7 μl of water per sample. All reactions were carried out in duplicate. RNA expression level fold changes were calculated using the ABIPrism 7000 SDS Software. Non-infected sample transcripts levels were used as the gene expression base line.

## Authors' contributions

SSA performed the vast majority of the experimental work and participated in data analysis. CC and ARG performed the microarray analysis. CC participated in manuscript writing. AST, XP and SHIK conducted the *P. yoelii *microarray experiment. MP drafted and wrote the manuscript. MMM conceived the study, and participated in its design and coordination. All authors read and approved the final manuscript.

## Supplementary Material

Additional file 1 **Expression ratios of the 1108 DE probesets in *P. berghei *infected vs. non-infected hepatoma cells**. The data provided represent the expression ratios of the 1108 DE probesets in *P. berghei *infected vs. non-infected hepatoma cells. logFC = log base of the fold changes of each transcript (rows), B-stat = log odds that a transcript is differentially expressed (B-stat>0), Gene Symbol, Gene Title, and gene ontology terms have been retrieved from the NetAffx centre at Affymetrix.com. The list is sorted in alphabetical order by the gene symbol identifying the transcripts. Click here for file

Additional file 2 **List of DE genes at all time in *P. berghei *ANKA infected cells compared to non-infected ones**. The data provided represent 24 DE genes at all time in *P. berghei *ANKA infected cells compared to non-infected ones. The annotation was obtained from NetAffx Centre  and Ensembl . The rows highlighted in grey indicate an up-regulation of a gene at all the time points assessed. Click here for file

Additional file 3 **Expression profiles of the top 5 ranked genes for each time point**. The data provided represent the expression profiles of the top 5 ranked genes for each time point. Expression profiles of each gene are shown in black (solid) for infected cells time course and in red (dashed) for non-infected cells time course. Note that the expressions of the biological values are given by the symbols while the average of the 2 is drawn to join the time points. Expression values are given as log2. Click here for file

Additional file 4 **Expression profiles of further genes mentioned in the body text**. The data provided represent the expression profiles of further genes mentioned in the body text. Expression profiles of each gene are shown in black (solid) for infected cells time course and in red (dashed) for non-infected cells time course. Note that the expressions of the biological values are given by the symbols while the average of the 2 is drawn to join the time points. Expression values are given as log2. Click here for file

Additional file 5 **Enrichment analysis of Gene Ontology terms for every transcript differentailly expressed (DE) at any of the time points**. The data provided represent the enrichment analysis of Gene Ontology terms for every transcript differentailly expressed (DE) at any of the time points. Gostats package for R was used to compute the hypergeometric test. Each list of DE transcripts at each time point was tested against the total list of transcripts in our analysis after filtering out transcripts acting as control, transcripts showing little variation accross samples, and transcripts without Entrez Gene ID or GO annotation. Click here for file

Additional file 6 **Similarities found between *P. berghei *infected cells and *P. yoelii *infected livers analysis**. The data provided represent the similarities found between *P. berghei *infected cells and *P. yoelii *infected livers analysis. Click here for file

Additional file 7 **List of *P. berghei*-infected cells modulated genes in *P. yoelii*-liver infected experiment**. The data provided represent the differentially expressed *P. berghei*-infected cell modulated genes in *P. yoelii*-liver infected experiment. Click here for file

Additional file 8 **Primer Sequences**. The data provided represent all the primer sequences used in the qRT-PCR throughout the work. Click here for file
