# Supplementary material for: Host cell transcriptional profiling during malaria liver stage infection reveals a coordinated and sequential set of biological events
Source: BMC Genomics. 2009 Jun 17;10:270. doi: 10.1186/1471-2164-10-270 (PMC2706893; doi:10.1186/1471-2164-10-270)
Supplement: Additional file 1 — Expression ratios of the 1108 DE probesets in P. berghei infected vs. non-infected hepatoma cells. The data provided represent the expression ratios of the 1108 DE probesets in P. berghei infected vs. non-infected hepatoma cells. logFC = log base of the fold changes of each transcript (rows), B-stat = log odds that a transcript is differentially expressed (B-stat>0), Gene Symbol, Gene Title, and gene ontology terms have been retrieved from the NetAffx centre at Affymetrix.com. The list is sorted in alphabetical order by the gene symbol identifying the transcripts. [file 1471-2164-10-270-S1.doc]

| **Additional File 1**: Expression ratios of the 1108 DE probesets in *P. berghei* infecetd vs non-infecetd hepatoma cells. logFc= log base of the fold changes of each transcript (rows), B-stat= log odds that a transcript is differentially expressed (B-stat>0), Gene Symbol, Gene Title, and gene ontology terms have been retrieved from the NetAffx centre at Affymetrix.com. The list is sorted in alphabetical order by the gene symbol characterising the transcripts. | | | | | | | | | | | | | |
| --- | --- | --- | --- | --- | --- | --- | --- | --- | --- | --- | --- | --- | --- |
|  |  |  |  |  |  |  |  |  |  |  |  |  |  |
|  | Inf. vs. non-Inf. at 6hpi | | Inf. vs. non-Inf. at 12hpi | | Inf. vs. non-Inf. at 18hpi | | Inf. vs. non-Inf. at 24hpi | |  |  |  |  |  |
| probesets | logFC | B-stat | logFC | B-stat | logFC | B-stat | logFC | B-stat | Gene Symbol | Gene Title | GO biological process term | GO molecular function term | GO cellular component term |
| 1430038_at | 0.88 | 3.54 | 0.03 | -6.25 | 0.30 | -3.96 | 0.07 | -5.94 | --- | --- | --- | --- | --- |
| 1440076_at | 1.24 | 1.69 | 1.07 | 0.44 | -0.13 | -6.15 | 0.57 | -3.41 | --- | --- | --- | --- | --- |
| 1446339_at | -0.29 | -5.27 | -1.05 | 1.76 | -0.30 | -5.10 | -0.16 | -5.71 | --- | --- | --- | --- | --- |
| 1452426_x_at | 0.57 | -3.04 | -1.23 | 2.74 | -0.27 | -5.39 | -0.08 | -6.02 | --- | --- | DNA integration | nucleic acid binding / DNA binding | --- |
| 1458050_at | -0.36 | -4.05 | -0.96 | 2.65 | -0.41 | -3.35 | -0.44 | -2.94 | --- | --- | --- | --- | --- |
| 1458886_at | 0.22 | -5.12 | -0.61 | 0.15 | -0.03 | -6.29 | -0.15 | -5.49 | --- | --- | --- | --- | --- |
| 1459497_at | -0.09 | -6.22 | -0.23 | -4.86 | -0.69 | 1.01 | -0.19 | -5.17 | --- | --- | --- | --- | --- |
| 1436798_at | -0.13 | -5.84 | 0.66 | 1.60 | 0.21 | -4.93 | 0.05 | -6.01 | --- | CDNA clone IMAGE:5039327 | translation | RNA binding / structural constituent of ribosome | intracellular / ribosome / ribonucleoprotein complex |
| 1455706_at | -0.75 | 1.78 | -0.31 | -3.88 | -0.20 | -5.20 | -0.25 | -4.54 | --- | RIKEN cDNA 6030470M02 gene, mRNA (cDNA clone IMAGE:30945724) | protein targeting / intracellular signaling cascade / insulin receptor signaling pathway / glucose transport | protein binding | --- |
| 1425113_x_at | -0.06 | -6.31 | -0.11 | -5.80 | -0.67 | 2.23 | -0.29 | -3.44 | --- | Transcribed locus | --- | --- | --- |
| 1436148_at | -0.78 | -4.62 | -1.57 | -0.90 | -1.86 | 0.38 | -1.59 | -0.77 | --- | Transcribed locus | --- | --- | --- |
| 1436571_at | 0.35 | -2.19 | 0.35 | -2.08 | 0.79 | 4.74 | 0.36 | -1.78 | --- | Transcribed locus | --- | --- | --- |
| 1437606_at | 0.11 | -6.05 | 0.27 | -4.20 | 0.70 | 1.82 | 0.31 | -3.59 | --- | Transcribed locus | --- | --- | --- |
| 1437673_at | -0.78 | 2.73 | -0.51 | -0.87 | -1.11 | 6.11 | -0.92 | 4.07 | --- | Transcribed locus | --- | --- | --- |
| 1438738_at | -0.13 | -5.92 | -0.11 | -5.93 | -0.64 | 0.82 | 0.21 | -4.86 | --- | Transcribed locus | --- | --- | --- |
| 1439050_at | -0.33 | -5.07 | 0.95 | 0.60 | 1.16 | 2.35 | 0.39 | -4.27 | --- | Transcribed locus | --- | --- | --- |
| 1439960_at | 0.10 | -6.03 | 0.28 | -3.56 | 0.61 | 1.72 | 0.31 | -2.91 | --- | Transcribed locus | --- | --- | --- |
| 1440027_at | -0.69 | 0.38 | -0.42 | -2.87 | -0.21 | -5.30 | -0.61 | -0.52 | --- | Transcribed locus | --- | --- | --- |
| 1440411_at | -1.17 | 3.91 | -0.95 | 1.94 | -0.79 | 0.35 | -0.41 | -3.60 | --- | Transcribed locus | --- | --- | --- |
| 1440662_at | -0.27 | -5.81 | -0.69 | -2.91 | -1.08 | 0.03 | -0.68 | -2.88 | --- | Transcribed locus | --- | --- | --- |
| 1441288_at | 0.56 | -0.99 | 0.67 | 0.48 | 0.70 | 0.76 | 0.99 | 3.70 | --- | Transcribed locus | --- | --- | --- |
| 1441623_at | -0.40 | -5.16 | -0.43 | -4.88 | -1.23 | 0.81 | -0.73 | -2.72 | --- | Transcribed locus | --- | --- | --- |
| 1442350_at | 0.06 | -6.34 | -0.43 | -1.80 | -0.82 | 3.49 | -0.73 | 2.40 | --- | Transcribed locus | --- | --- | --- |
| 1442431_at | 0.27 | -4.46 | 0.33 | -3.60 | 0.60 | 0.15 | 0.02 | -6.09 | --- | Transcribed locus | --- | --- | --- |
| 1442537_at | 0.16 | -5.68 | 0.71 | 1.41 | 0.49 | -1.51 | 0.10 | -5.79 | --- | Transcribed locus | --- | --- | --- |
| 1442783_x_at | 0.16 | -5.95 | 0.30 | -4.71 | 0.75 | 0.10 | 0.31 | -4.52 | --- | Transcribed locus | --- | --- | --- |
| 1442835_at | -0.18 | -5.36 | -0.64 | 1.38 | -0.46 | -1.20 | -0.24 | -4.33 | --- | Transcribed locus | --- | --- | --- |
| 1443196_at | -0.20 | -5.42 | -0.51 | -1.60 | -0.69 | 0.68 | -0.58 | -0.65 | --- | Transcribed locus | --- | --- | --- |
| 1443546_at | 0.09 | -6.26 | -0.15 | -5.79 | -0.74 | 0.55 | -0.49 | -2.28 | --- | Transcribed locus | --- | --- | --- |
| 1443602_at | -0.11 | -6.11 | -0.35 | -3.61 | -1.00 | 4.04 | -0.20 | -5.09 | --- | Transcribed locus | --- | --- | --- |
| 1447340_at | -0.43 | -3.13 | -0.74 | 0.55 | -0.52 | -1.98 | -0.59 | -1.05 | --- | Transcribed locus | --- | --- | --- |
| 1452886_at | 0.20 | -4.76 | 0.01 | -6.27 | 0.59 | 2.02 | -0.01 | -6.09 | --- | Transcribed locus | --- | --- | --- |
| 1455165_at | -0.35 | -3.68 | 0.21 | -5.20 | -0.66 | 0.42 | -0.41 | -2.61 | --- | Transcribed locus | --- | --- | --- |
| 1455249_at | -0.33 | -3.06 | -0.40 | -1.68 | -0.54 | 0.48 | -0.85 | 4.32 | --- | Transcribed locus | --- | --- | --- |
| 1455320_at | -0.53 | -1.61 | -0.20 | -5.38 | -0.73 | 0.85 | -0.40 | -3.02 | --- | Transcribed locus | --- | --- | --- |
| 1455781_at | -0.58 | 0.22 | -0.24 | -4.51 | -0.30 | -3.83 | -0.02 | -6.08 | --- | Transcribed locus | --- | --- | --- |
| 1456521_at | 0.18 | -5.57 | -0.77 | 1.76 | -0.58 | -0.50 | -0.67 | 0.63 | --- | Transcribed locus | --- | --- | --- |
| 1456778_at | 1.47 | 5.51 | 0.81 | 0.07 | 0.43 | -3.77 | 0.20 | -5.47 | --- | Transcribed locus | --- | --- | --- |
| 1457141_at | -1.24 | 1.61 | -1.14 | 0.93 | -1.50 | 3.37 | -0.74 | -2.08 | --- | Transcribed locus | --- | --- | --- |
| 1459681_at | 0.82 | 6.48 | -0.05 | -6.13 | 0.02 | -6.28 | -0.28 | -2.40 | --- | Transcribed locus | --- | --- | --- |
| 1460595_at | -0.31 | -4.13 | -0.09 | -6.05 | -0.60 | -0.31 | -0.68 | 0.75 | --- | Transcribed locus | --- | --- | --- |
| 1437967_at | -1.31 | 1.60 | -1.04 | -0.22 | -0.99 | -0.64 | -0.48 | -4.30 | --- | Transcribed locus, moderately similar to XP_001066067.1 PREDICTED: hypothetical protein [Rattus norvegicus] | --- | --- | --- |
| 1455869_at | -0.47 | -4.50 | -0.61 | -3.23 | -1.52 | 3.50 | -1.19 | 1.22 | --- | Transcribed locus, moderately similar to XP_001477892.1 PREDICTED: hypothetical protein [Mus musculus] | --- | --- | --- |
| 1434100_x_at | 0.05 | -6.41 | 0.07 | -6.21 | 0.83 | 0.12 | 0.19 | -5.54 | --- | Transcribed locus, strongly similar to NP_037393.1 peroxisome proliferator-activated receptor gamma, coactivator 1 alpha [Homo sapiens] | respiratory electron transport chain / positive regulation of transcription / positive regulation of transcription from RNA polymerase II promoter | DNA binding / transcription activator activity | nucleus |
| 1433900_at | 0.77 | 2.37 | 0.39 | -2.72 | 0.35 | -3.19 | -0.04 | -6.05 | 0610007L01Rik | RIKEN cDNA 0610007L01 gene | --- | --- | membrane / integral to membrane |
| 1454161_s_at | -0.60 | 3.48 | -0.47 | 1.24 | -0.34 | -1.27 | -0.19 | -4.18 | 0610007P14Rik | RIKEN cDNA 0610007P14 gene | steroid biosynthetic process / lipid biosynthetic process / sterol biosynthetic process | --- | endoplasmic reticulum / endoplasmic reticulum membrane / membrane / integral to membrane / transport vesicle |
| 1437835_a_at | 0.16 | -5.73 | 0.28 | -4.36 | 0.62 | 0.02 | 0.43 | -2.32 | 0610011L14Rik | RIKEN cDNA 0610011L14 gene | --- | protein binding | --- |
| 1454126_at | -0.28 | -5.59 | -0.66 | -2.61 | -1.07 | 0.76 | -1.15 | 1.33 | 0710001A04Rik | RIKEN cDNA 0710001A04 gene | --- | --- | --- |
| 1459890_s_at | 0.18 | -5.63 | 0.26 | -4.65 | 0.59 | -0.51 | 0.63 | 0.11 | 1110008P14Rik | RIKEN cDNA 1110008P14 gene | --- | --- | --- |
| 1437286_x_at | -0.85 | -1.71 | -0.91 | -1.13 | -1.14 | 0.57 | -0.40 | -4.74 | 1110020G09Rik | RIKEN cDNA 1110020G09 gene | metabolic process | NAD+ kinase activity | mitochondrion |
| 1437287_at | -0.69 | 0.44 | -0.77 | 1.34 | -0.47 | -2.24 | -0.88 | 2.49 | 1110020G09Rik | RIKEN cDNA 1110020G09 gene | metabolic process | NAD+ kinase activity | mitochondrion |
| 1434340_at | 0.08 | -6.22 | 0.58 | 0.61 | 0.76 | 2.97 | 0.65 | 1.50 | 1110020P15Rik | RIKEN cDNA 1110020P15 gene | mitochondrial electron transport, ubiquinol to cytochrome c / transport / oxidation reduction | ubiquinol-cytochrome-c reductase activity / ubiquinol-cytochrome-c reductase activity / oxidoreductase activity | mitochondrion / mitochondrial envelope / mitochondrial inner membrane / mitochondrial respiratory chain / membrane / organelle inner membrane |
| 1429242_at | 0.70 | 1.90 | 0.53 | -0.32 | 0.95 | 4.79 | 0.47 | -1.21 | 1110054O05Rik | RIKEN cDNA 1110054O05 gene | --- | --- | --- |
| 1429243_at | 0.46 | -2.81 | 0.53 | -1.92 | 0.74 | 0.55 | 0.37 | -3.64 | 1110054O05Rik | RIKEN cDNA 1110054O05 gene | --- | --- | --- |
| 1436203_a_at | -1.91 | 2.84 | -1.11 | -1.53 | -0.66 | -4.19 | -0.52 | -4.75 | 1110059G02Rik | RIKEN cDNA 1110059G02 gene | --- | --- | --- |
| 1436204_at | -1.51 | 4.91 | -1.02 | 1.26 | -0.36 | -4.68 | -0.58 | -2.69 | 1110059G02Rik | RIKEN cDNA 1110059G02 gene | --- | --- | --- |
| 1433581_at | -1.17 | 1.34 | -1.03 | 0.32 | -1.80 | 5.40 | -0.94 | -0.39 | 1190002N15Rik / LOC100044725 | RIKEN cDNA 1190002N15 gene / hypothetical protein LOC100044725 | --- | --- | --- |
| 1433582_at | -0.75 | 1.81 | -1.05 | 4.95 | -0.62 | 0.28 | -0.59 | -0.17 | 1190002N15Rik / LOC100044725 | RIKEN cDNA 1190002N15 gene / hypothetical protein LOC100044725 | --- | --- | --- |
| 1429065_at | -0.46 | -3.26 | -0.24 | -5.31 | -0.93 | 1.78 | -0.28 | -4.81 | 1200009F10Rik | RIKEN cDNA 1200009F10 gene | induction of apoptosis / response to X-ray | protein binding | endoplasmic reticulum |
| 1452418_at | 1.95 | 5.11 | 0.45 | -4.74 | 0.01 | -6.31 | -0.22 | -5.68 | 1200016E24Rik | RIKEN cDNA 1200016E24 gene | --- | --- | --- |
| 1453339_at | -0.53 | -0.55 | -0.70 | 1.69 | -0.46 | -1.62 | -0.39 | -2.44 | 1700008I05Rik | RIKEN cDNA 1700008I05 gene | --- | --- | --- |
| 1419294_at | -0.91 | 0.06 | -0.57 | -2.98 | -0.11 | -6.15 | -0.14 | -5.84 | 1700011H14Rik | RIKEN cDNA 1700011H14 gene | --- | --- | --- |
| 1430261_at | 0.11 | -6.36 | -0.28 | -5.74 | -1.50 | 1.75 | -0.39 | -5.11 | 1700024J04Rik | RIKEN cDNA 1700024J04 gene | --- | receptor activity | --- |
| 1434422_at | -0.59 | 1.89 | -0.19 | -4.68 | -0.39 | -1.30 | -0.17 | -4.87 | 1700066M21Rik | RIKEN cDNA 1700066M21 gene | --- | --- | extracellular region |
| 1456875_at | 0.25 | -4.79 | 0.44 | -2.11 | 0.60 | 0.05 | 0.18 | -5.17 | 1810013L24Rik | RIKEN cDNA 1810013L24 gene | --- | --- | --- |
| 1425120_x_at | -0.49 | -3.30 | -0.53 | -2.79 | -0.83 | 0.24 | -0.60 | -1.97 | 1810023F06Rik | RIKEN cDNA 1810023F06 gene | --- | --- | extracellular space / integral to membrane |
| 1429008_at | -1.00 | 0.02 | -0.79 | -1.64 | -0.94 | -0.37 | -0.52 | -3.71 | 1810074P20Rik | RIKEN cDNA 1810074P20 gene | --- | --- | --- |
| 1429261_at | 0.04 | -6.36 | 0.38 | -1.90 | 0.46 | -0.51 | 0.65 | 2.30 | 2210411K11Rik | RIKEN cDNA 2210411K11 gene | --- | --- | --- |
| 1453511_at | -0.93 | 0.56 | -0.13 | -6.04 | 0.37 | -4.54 | 0.12 | -5.90 | 2310007B03Rik | RIKEN cDNA 2310007B03 gene | --- | --- | --- |
| 1452889_at | -0.58 | 1.25 | -0.41 | -1.45 | 0.17 | -5.16 | 0.05 | -5.98 | 2310007H09Rik | RIKEN cDNA 2310007H09 gene | metabolic process | catalytic activity / inorganic diphosphatase activity / hydrolase activity | extracellular space |
| 1429898_at | -0.03 | -6.43 | -0.27 | -5.21 | -1.01 | 1.80 | -0.42 | -3.77 | 2310008M10Rik | RIKEN cDNA 2310008M10 gene | --- | --- | membrane / integral to membrane |
| 1443339_at | -0.12 | -6.26 | -0.98 | 0.46 | -0.30 | -5.24 | 0.06 | -6.06 | 2310056P07Rik | RIKEN cDNA 2310056P07 gene | --- | --- | membrane / integral to membrane |
| 1429671_at | 1.10 | 4.32 | 0.32 | -4.21 | 0.78 | 1.14 | 0.66 | -0.20 | 2410018M08Rik | RIKEN cDNA 2410018M08 gene | --- | --- | --- |
| 1420113_s_at | 0.11 | -6.20 | 0.50 | -2.31 | 0.68 | -0.15 | 0.78 | 0.89 | 2410022L05Rik | RIKEN cDNA 2410022L05 gene | --- | --- | --- |
| 1428404_at | 0.03 | -6.42 | 0.17 | -5.53 | 0.64 | 0.15 | 0.44 | -2.37 | 2410025L10Rik | RIKEN cDNA 2410025L10 gene | --- | --- | --- |
| 1455935_at | 0.30 | -3.72 | 0.33 | -3.04 | 0.59 | 0.91 | 0.40 | -1.95 | 2410131K14Rik | RIKEN cDNA 2410131K14 gene | --- | --- | extracellular region |
| 1434144_s_at | 0.90 | 6.03 | 0.35 | -1.96 | -0.05 | -6.19 | -0.07 | -5.86 | 2410187C16Rik | RIKEN cDNA 2410187C16 gene | --- | --- | --- |
| 1455657_at | 0.85 | 0.73 | 0.54 | -2.45 | 0.10 | -6.15 | 0.18 | -5.57 | 2610207I05Rik | RIKEN cDNA 2610207I05 gene | nuclear-transcribed mRNA catabolic process, nonsense-mediated decay / DNA repair / response to DNA damage stimulus / protein amino acid autophosphorylation / phosphoinositide phosphorylation | inositol or phosphatidylinositol kinase activity / protein serine/threonine kinase activity / binding / protein binding / kinase activity / transferase activity / phosphotransferase activity, alcohol group as acceptor / manganese ion binding / metal ion binding | nucleus / cytoplasm |
| 1428519_at | 0.06 | -6.34 | 0.54 | -0.75 | 1.00 | 4.53 | 0.73 | 1.57 | 2610528E23Rik | RIKEN cDNA 2610528E23 gene | --- | --- | --- |
| 1459902_at | 0.76 | 2.13 | 0.48 | -1.49 | 0.41 | -2.49 | 0.25 | -4.41 | 2700007P21Rik | RIKEN cDNA 2700007P21 gene | --- | --- | --- |
| 1451556_a_at | 0.16 | -5.44 | 0.14 | -5.51 | 0.74 | 3.22 | 0.41 | -1.47 | 2700078E11Rik | RIKEN cDNA 2700078E11 gene | ubiquitin-dependent protein catabolic process | DNA binding / ubiquitin protein ligase binding | nucleus / cullin-RING ubiquitin ligase complex |
| 1453241_a_at | -0.59 | 1.70 | -0.29 | -3.14 | 0.08 | -6.04 | -0.16 | -5.02 | 2810047C21Rik / EG665577 | RIKEN cDNA 2810047C21 gene / predicted gene, EG665577 | regulation of transcription, DNA-dependent | nucleic acid binding / zinc ion binding | intracellular |
| 1445606_a_at | -0.13 | -5.98 | -0.58 | -0.26 | -1.06 | 5.02 | -0.57 | -0.38 | 2900009J06Rik | RIKEN cDNA 2900009J06 gene | --- | --- | --- |
| 1429503_at | -0.94 | 7.40 | -0.29 | -2.67 | -0.24 | -3.64 | -0.19 | -4.39 | 2900024C23Rik | RIKEN cDNA 2900024C23 gene | --- | protein binding | membrane / integral to membrane |
| 1437261_at | -0.32 | -3.64 | -0.59 | 0.34 | -0.36 | -2.92 | -0.40 | -2.30 | 2900024O10Rik | RIKEN cDNA 2900024O10 gene | --- | zinc ion binding / hydrolase activity / metal ion binding | --- |
| 1440917_at | 0.35 | -4.14 | 0.47 | -2.55 | 0.22 | -5.24 | 0.72 | 0.41 | 2900093K20Rik | RIKEN cDNA 2900093K20 gene | --- | --- | --- |
| 1432646_a_at | 0.42 | -2.89 | 0.15 | -5.72 | 0.78 | 1.63 | 0.35 | -3.53 | 2900097C17Rik | RIKEN cDNA 2900097C17 gene | --- | --- | endoplasmic reticulum / membrane / integral to endoplasmic reticulum membrane |
| 1453192_at | -0.72 | 0.22 | -0.23 | -5.15 | 0.14 | -5.86 | -0.02 | -6.09 | 3110032G18Rik | RIKEN cDNA 3110032G18 gene | transport | receptor activity / transporter activity | membrane |
| 1428468_at | 0.21 | -4.86 | -0.49 | -0.41 | -0.66 | 2.10 | -0.54 | 0.36 | 3110043O21Rik | RIKEN cDNA 3110043O21 gene | --- | --- | --- |
| 1429771_at | -0.45 | -1.42 | -0.71 | 2.31 | -0.51 | -0.44 | -0.34 | -2.92 | 3110073H01Rik | RIKEN cDNA 3110073H01 gene | --- | --- | --- |
| 1434550_at | -0.58 | -0.49 | -0.63 | 0.22 | -0.35 | -3.55 | -0.19 | -5.12 | 3830406C13Rik | RIKEN cDNA 3830406C13 gene | --- | --- | --- |
| 1452092_at | -0.75 | 1.07 | -0.37 | -3.61 | -0.57 | -1.14 | -0.38 | -3.26 | 4631426J05Rik | RIKEN cDNA 4631426J05 gene | hexose biosynthetic process | sulfotransferase activity / transferase activity / 3'-phosphoadenosine 5'-phosphosulfate binding / N-acetylgalactosamine 4-sulfate 6-O-sulfotransferase activity | Golgi membrane / Golgi apparatus / membrane / integral to membrane |
| 1429734_at | 0.27 | -5.73 | 0.41 | -4.77 | 1.06 | 0.24 | 0.46 | -4.30 | 4632434I11Rik | RIKEN cDNA 4632434I11 gene | apoptosis / cell cycle / cell cycle arrest | --- | nucleus / cytoplasm |
| 1434240_at | 0.46 | -2.84 | 0.34 | -4.14 | 0.82 | 1.39 | 0.45 | -2.68 | 4632434I11Rik | RIKEN cDNA 4632434I11 gene | apoptosis / cell cycle / cell cycle arrest | --- | nucleus / cytoplasm |
| 1433672_at | -0.73 | 4.05 | -0.26 | -3.66 | -0.33 | -2.42 | -0.28 | -3.16 | 4732479N06Rik | RIKEN cDNA 4732479N06 gene | --- | methyltransferase activity / S-adenosylmethionine-dependent methyltransferase activity / transferase activity | --- |
| 1454841_at | 0.64 | 2.13 | 0.00 | -6.28 | 0.48 | -0.40 | 0.48 | -0.32 | 4921511H13Rik | RIKEN cDNA 4921511H13 gene | --- | --- | nucleus |
| 1423484_at | -0.71 | 0.04 | -0.29 | -4.64 | -0.44 | -3.05 | -0.08 | -5.97 | 4930533K18Rik / Bicc1 | RIKEN cDNA 4930533K18 gene / bicaudal C homolog 1 (Drosophila) | multicellular organismal development | nucleic acid binding / RNA binding | --- |
| 1450409_a_at | -0.09 | -6.05 | 0.50 | 0.70 | 0.65 | 2.93 | 0.65 | 2.86 | 4930570C03Rik | RIKEN cDNA 4930570C03 gene | --- | --- | --- |
| 1432059_x_at | 0.68 | 1.52 | 0.34 | -3.22 | 0.59 | 0.28 | 0.44 | -1.73 | 5031425E22Rik | RIKEN cDNA 5031425E22 gene | --- | --- | --- |
| 1436999_at | -1.30 | 9.78 | -1.29 | 9.28 | -0.85 | 5.44 | -0.79 | 4.44 | 5033414K04Rik | RIKEN cDNA 5033414K04 gene | --- | --- | cytoplasm |
| 1444969_at | -0.33 | -4.02 | -0.57 | -0.83 | -0.64 | 0.10 | -0.13 | -5.66 | 5033414K04Rik | RIKEN cDNA 5033414K04 gene | --- | --- | cytoplasm |
| 1439071_at | 0.98 | 0.72 | 0.87 | -0.20 | 0.80 | -0.82 | 0.26 | -5.28 | 5430416N02Rik | RIKEN cDNA 5430416N02 gene | --- | --- | --- |
| 1434756_at | -0.95 | 0.74 | -0.63 | -2.24 | -0.63 | -2.25 | -0.45 | -3.70 | 5430421B17 | hypothetical protein 5430421B17 | --- | --- | --- |
| 1428796_at | -0.65 | 0.86 | 0.03 | -6.24 | 0.10 | -5.97 | -0.17 | -5.24 | 5530401J07Rik | RIKEN cDNA 5530401J07 gene | --- | --- | --- |
| 1452750_at | -0.09 | -6.09 | -0.05 | -6.16 | 0.59 | 1.28 | 0.30 | -3.24 | 5530601H04Rik | RIKEN cDNA 5530601H04 gene | --- | --- | --- |
| 1447774_x_at | -0.40 | -1.27 | -0.65 | 2.82 | -0.50 | 0.58 | -0.31 | -2.69 | 5730469M10Rik | RIKEN cDNA 5730469M10 gene | --- | --- | extracellular region |
| 1428189_at | -0.74 | 3.70 | -0.54 | 0.72 | -0.07 | -6.12 | -0.06 | -5.93 | 5730494M16Rik | RIKEN cDNA 5730494M16 gene | --- | --- | nucleus / cytoplasm / cytoskeleton / microtubule |
| 1441960_x_at | -0.78 | 2.67 | -0.38 | -2.79 | 0.01 | -6.31 | 0.11 | -5.74 | 5730494M16Rik | RIKEN cDNA 5730494M16 gene | --- | --- | nucleus / cytoplasm / cytoskeleton / microtubule |
| 1444013_at | -0.35 | -3.36 | -0.41 | -2.38 | -0.59 | 0.19 | -0.21 | -4.89 | 5930412G12Rik | RIKEN cDNA 5930412G12 gene | --- | --- | --- |
| 1453783_at | -0.65 | 2.66 | -0.64 | 2.50 | -0.33 | -2.55 | -0.37 | -1.65 | 6330411E07Rik | RIKEN cDNA 6330411E07 gene | --- | --- | --- |
| 1454609_x_at | 0.46 | 0.17 | 0.28 | -3.02 | 0.65 | 3.29 | 0.07 | -5.81 | 6430527G18Rik | RIKEN cDNA 6430527G18 gene | --- | protein binding / zinc ion binding / metal ion binding | nucleus |
| 1451456_at | -0.09 | -6.24 | -0.41 | -2.92 | -0.64 | 0.09 | -0.17 | -5.42 | 6430706D22Rik | RIKEN cDNA 6430706D22 gene | --- | --- | --- |
| 1433962_at | 1.02 | -0.38 | 0.86 | -1.53 | 1.30 | 1.71 | 0.44 | -4.48 | 6720458F09Rik | RIKEN cDNA 6720458F09 gene | tRNA processing | protein binding / methyltransferase activity / S-adenosylmethionine-dependent methyltransferase activity / tRNA (adenine-N1-)-methyltransferase activity / transferase activity | nucleus |
| 1453953_at | 0.19 | -5.49 | 0.15 | -5.68 | -0.61 | 0.01 | -0.47 | -1.75 | 9130015A21Rik | RIKEN cDNA 9130015A21 gene | --- | --- | --- |
| 1427425_at | 0.27 | -5.37 | -0.31 | -4.90 | -0.85 | 0.18 | -0.14 | -5.81 | 9130208E07Rik | RIKEN cDNA 9130208E07 gene | --- | --- | --- |
| 1428891_at | -0.75 | 0.22 | -0.46 | -2.94 | -0.53 | -2.16 | -0.52 | -2.18 | 9130213B05Rik | RIKEN cDNA 9130213B05 gene | --- | --- | extracellular space / membrane / integral to membrane |
| 1434574_at | 0.46 | -1.48 | 0.58 | 0.33 | 0.26 | -4.34 | 0.15 | -5.41 | 9430008C03Rik | RIKEN cDNA 9430008C03 gene | --- | --- | --- |
| 1429792_at | 0.39 | -2.70 | 0.39 | -2.50 | 0.62 | 0.69 | 0.30 | -3.69 | 9530048O09Rik | RIKEN cDNA 9530048O09 gene | --- | --- | --- |
| 1417212_at | 0.18 | -5.42 | 0.36 | -2.77 | 0.66 | 1.46 | 0.52 | -0.44 | 9530058B02Rik | RIKEN cDNA 9530058B02 gene | --- | protein binding | --- |
| 1439808_at | -1.50 | 7.37 | -1.59 | 7.69 | -0.99 | 3.22 | -1.18 | 4.62 | A130090K04Rik | RIKEN cDNA A130090K04 gene | --- | --- | cytoplasm / plasma membrane / membrane |
| 1441638_at | -0.31 | -2.82 | -0.45 | -0.31 | -0.59 | 1.90 | -0.49 | 0.30 | A630014C17Rik | RIKEN cDNA A630014C17 gene | --- | --- | --- |
| 1438531_at | -1.52 | 1.38 | -0.82 | -2.96 | -0.37 | -5.45 | -0.39 | -5.17 | A730054J21Rik | RIKEN cDNA A730054J21 gene | --- | --- | --- |
| 1439514_at | -0.60 | -1.63 | -0.84 | 1.07 | -0.05 | -6.25 | -0.07 | -5.99 | A930004D18Rik | RIKEN cDNA A930004D18 gene | --- | --- | --- |
| 1438747_at | 0.07 | -6.29 | 0.27 | -4.43 | -0.76 | 1.96 | 0.27 | -4.23 | A930006K02Rik | RIKEN cDNA A930006K02 gene | --- | --- | --- |
| 1436942_at | 0.00 | -6.45 | -0.46 | -2.35 | -0.65 | 0.00 | -0.57 | -0.92 | A930035D04Rik | RIKEN cDNA A930035D04 gene | --- | --- | --- |
| 1454628_at | 0.11 | -6.07 | 0.35 | -3.25 | 0.60 | 0.27 | 0.29 | -3.91 | A930037G23Rik | RIKEN cDNA A930037G23 gene | --- | --- | --- |
| 1455180_at | -0.76 | 2.80 | -0.42 | -1.90 | -0.41 | -1.98 | -0.44 | -1.45 | AA407270 | expressed sequence AA407270 | --- | --- | --- |
| 1423685_at | 0.86 | 2.58 | 0.78 | 1.73 | 0.99 | 3.87 | 0.70 | 0.78 | Aars | alanyl-tRNA synthetase | translation / alanyl-tRNA aminoacylation / tRNA aminoacylation | nucleotide binding / nucleic acid binding / aminoacyl-tRNA ligase activity / alanine-tRNA ligase activity / ATP binding / ligase activity, forming aminoacyl-tRNA and related compounds | cytoplasm |
| 1451083_s_at | 0.96 | 5.52 | 0.85 | 4.19 | 1.01 | 5.92 | 0.74 | 2.81 | Aars | alanyl-tRNA synthetase | translation / alanyl-tRNA aminoacylation / tRNA aminoacylation | nucleotide binding / nucleic acid binding / aminoacyl-tRNA ligase activity / alanine-tRNA ligase activity / ATP binding / ligase activity, forming aminoacyl-tRNA and related compounds | cytoplasm |
| 1434474_at | -0.48 | -2.18 | -0.65 | 0.14 | -0.99 | 3.81 | -0.75 | 1.26 | Abca5 | ATP-binding cassette, sub-family A (ABC1), member 5 | transport / transport | nucleotide binding / nucleotide binding / ATP binding / ATP binding / ATPase activity / nucleoside-triphosphatase activity | Golgi membrane / lysosome / lysosomal membrane / endosome / Golgi apparatus / membrane / integral to membrane / late endosome membrane |
| 1427546_at | -0.80 | -0.19 | -1.12 | 2.73 | -1.07 | 2.33 | -0.53 | -2.77 | Abca8b | ATP-binding cassette, sub-family A (ABC1), member 8b | transport / transport | nucleotide binding / ATP binding / ATP binding / ATPase activity / nucleoside-triphosphatase activity / ATPase activity, coupled to transmembrane movement of substances | mitochondrial inner membrane / plasma membrane / integral to membrane |
| 1443870_at | 0.24 | -5.21 | 0.69 | 0.20 | 0.64 | -0.43 | 0.53 | -1.66 | Abcc4 | ATP-binding cassette, sub-family C (CFTR/MRP), member 4 | transport / ion transport / multidrug transport | nucleotide binding / chloride channel activity / ATP binding / multidrug efflux pump activity / ATPase activity / nucleoside-triphosphatase activity / ATPase activity, coupled to transmembrane movement of substances | membrane fraction / plasma membrane / membrane / integral to membrane / platelet dense granule membrane |
| 1419748_at | -2.28 | 5.19 | -1.94 | 3.52 | -1.55 | 1.51 | -1.18 | -0.65 | Abcd2 | ATP-binding cassette, sub-family D (ALD), member 2 | transport / transport | nucleotide binding / nucleotide binding / protein binding / protein binding / ATP binding / ATPase activity / nucleoside-triphosphatase activity / ATPase activity, coupled to transmembrane movement of substances | peroxisome / peroxisomal membrane / membrane / membrane / integral to membrane |
| 1438431_at | -2.17 | 9.30 | -1.78 | 7.09 | -2.04 | 8.43 | -1.61 | 5.80 | Abcd2 | ATP-binding cassette, sub-family D (ALD), member 2 | transport / transport | nucleotide binding / nucleotide binding / protein binding / protein binding / ATP binding / ATPase activity / nucleoside-triphosphatase activity / ATPase activity, coupled to transmembrane movement of substances | peroxisome / peroxisomal membrane / membrane / membrane / integral to membrane |
| 1456812_at | -0.71 | 1.02 | -0.58 | -0.57 | -0.60 | -0.29 | -0.35 | -3.45 | Abcd2 | ATP-binding cassette, sub-family D (ALD), member 2 | transport / transport | nucleotide binding / nucleotide binding / protein binding / protein binding / ATP binding / ATPase activity / nucleoside-triphosphatase activity / ATPase activity, coupled to transmembrane movement of substances | peroxisome / peroxisomal membrane / membrane / membrane / integral to membrane |
| 1417565_at | 0.21 | -4.98 | 0.40 | -2.19 | 0.59 | 0.67 | 0.22 | -4.63 | Abhd5 | abhydrolase domain containing 5 | proteolysis / lipid metabolic process / positive regulation of lipoprotein lipase activity | aminopeptidase activity / protein binding / hydrolase activity | cytoplasm / lipid particle / lipid particle / cytosol / monolayer-surrounded lipid storage body |
| 1417566_at | 0.05 | -6.39 | 0.22 | -5.40 | 0.89 | 1.65 | 0.76 | 0.36 | Abhd5 | abhydrolase domain containing 5 | proteolysis / lipid metabolic process / positive regulation of lipoprotein lipase activity | aminopeptidase activity / protein binding / hydrolase activity | cytoplasm / lipid particle / lipid particle / cytosol / monolayer-surrounded lipid storage body |
| 1438506_s_at | -0.05 | -6.34 | -0.63 | 2.58 | 0.27 | -3.39 | -0.11 | -5.49 | Abi1 | abl-interactor 1 | somitogenesis / cellular process / cellular process / peptidyl-tyrosine phosphorylation | protein binding / protein tyrosine kinase activator activity | intracellular / nucleus / cytoplasm / cytoskeleton / synaptosome / lamellipodium / lamellipodium / cell junction / leading edge / cell projection / synapse |
| 1433453_a_at | 1.75 | 3.95 | 0.74 | -2.77 | 0.91 | -1.52 | 0.50 | -4.27 | Abtb2 | ankyrin repeat and BTB (POZ) domain containing 2 | --- | DNA binding / protein binding | --- |
| 1433454_at | 0.78 | 2.31 | 0.62 | 0.33 | 0.14 | -5.71 | 0.03 | -6.07 | Abtb2 | ankyrin repeat and BTB (POZ) domain containing 2 | --- | DNA binding / protein binding | --- |
| 1435630_s_at | -0.60 | -0.10 | -0.66 | 0.72 | -0.48 | -1.63 | -0.40 | -2.58 | Acat2 | acetyl-Coenzyme A acetyltransferase 2 | metabolic process | catalytic activity / acetyl-CoA C-acetyltransferase activity / acyltransferase activity / transferase activity | cytoplasm |
| 1439478_at | 0.48 | -4.10 | 0.97 | 0.26 | 1.37 | 3.32 | 0.65 | -2.36 | Acot2 | acyl-CoA thioesterase 2 | lipid metabolic process / acyl-CoA metabolic process | carboxylesterase activity / palmitoyl-CoA hydrolase activity / acyl-CoA thioesterase activity / hydrolase activity | mitochondrion / mitochondrion / mitochondrion / mitochondrial matrix |
| 1456735_x_at | -0.79 | 2.99 | -0.18 | -5.22 | -0.10 | -5.96 | -0.02 | -6.08 | Acpl2 | acid phosphatase-like 2 | --- | acid phosphatase activity / hydrolase activity | extracellular region |
| 1422526_at | -1.24 | 0.67 | -0.96 | -1.20 | -0.71 | -3.02 | -0.35 | -5.14 | Acsl1 | acyl-CoA synthetase long-chain family member 1 | lipid metabolic process / fatty acid metabolic process / metabolic process | magnesium ion binding / catalytic activity / long-chain-fatty-acid-CoA ligase activity / ligase activity / acetate-CoA ligase (ADP-forming) activity | mitochondrion / mitochondrion / mitochondrial outer membrane / peroxisome / peroxisomal membrane / endoplasmic reticulum / endoplasmic reticulum membrane / microsome / membrane / integral to membrane |
| 1423883_at | -0.90 | 4.12 | -0.81 | 3.00 | -0.61 | 0.57 | -0.50 | -0.87 | Acsl1 | acyl-CoA synthetase long-chain family member 1 | lipid metabolic process / fatty acid metabolic process / metabolic process | magnesium ion binding / catalytic activity / long-chain-fatty-acid-CoA ligase activity / ligase activity / acetate-CoA ligase (ADP-forming) activity | mitochondrion / mitochondrial outer membrane / peroxisome / peroxisomal membrane / endoplasmic reticulum / endoplasmic reticulum membrane / microsome / membrane / integral to membrane |
| 1450643_s_at | -1.04 | 0.33 | -1.00 | 0.06 | -0.71 | -2.30 | -0.65 | -2.69 | Acsl1 | acyl-CoA synthetase long-chain family member 1 | lipid metabolic process / fatty acid metabolic process / metabolic process | magnesium ion binding / catalytic activity / long-chain-fatty-acid-CoA ligase activity / ligase activity / acetate-CoA ligase (ADP-forming) activity | mitochondrion / mitochondrial outer membrane / peroxisome / peroxisomal membrane / endoplasmic reticulum / endoplasmic reticulum membrane / microsome / membrane / integral to membrane |
| 1452771_s_at | -0.60 | -0.74 | -0.56 | -1.08 | -0.75 | 1.15 | -0.32 | -3.98 | Acsl3 | acyl-CoA synthetase long-chain family member 3 | lipid metabolic process / fatty acid metabolic process / metabolic process | magnesium ion binding / catalytic activity / long-chain-fatty-acid-CoA ligase activity / long-chain-fatty-acid-CoA ligase activity / protein binding / ligase activity | mitochondrion / mitochondrial outer membrane / peroxisome / peroxisomal membrane / endoplasmic reticulum / endoplasmic reticulum membrane / microsome / membrane / integral to membrane |
| 1433531_at | -0.34 | -2.91 | -0.62 | 1.38 | -0.52 | -0.12 | -0.33 | -2.88 | Acsl4 | acyl-CoA synthetase long-chain family member 4 | lipid metabolic process / fatty acid metabolic process / metabolic process / regulation of fatty acid metabolic process | magnesium ion binding / catalytic activity / long-chain-fatty-acid-CoA ligase activity / long-chain-fatty-acid-CoA ligase activity / ligase activity | mitochondrion / mitochondrial outer membrane / peroxisome / peroxisomal membrane / endoplasmic reticulum / endoplasmic reticulum membrane / microsome / membrane / integral to membrane / integral to membrane |
| 1422478_a_at | -1.00 | 0.34 | -1.10 | 1.15 | -0.67 | -2.41 | -0.73 | -1.82 | Acss2 | acyl-CoA synthetase short-chain family member 2 | acetyl-CoA biosynthetic process / metabolic process | catalytic activity / acetate-CoA ligase activity / acetate-CoA ligase activity / AMP binding / ligase activity | cytoplasm / cytoplasm / integral to membrane |
| 1417976_at | -0.13 | -6.28 | 0.26 | -5.63 | 1.09 | 0.52 | 0.89 | -0.91 | Ada | adenosine deaminase | purine nucleotide metabolic process / immune response / nucleotide metabolic process / purine ribonucleoside monophosphate biosynthetic process | adenosine deaminase activity / adenosine deaminase activity / adenosine deaminase activity / hydrolase activity / metal ion binding | cytoplasm |
| 1450716_at | 1.45 | 6.85 | 0.84 | 1.55 | 0.36 | -3.96 | 0.40 | -3.37 | Adamts1 | a disintegrin-like and metallopeptidase (reprolysin type) with thrombospondin type 1 motif, 1 | ovulation from ovarian follicle / kidney development / proteolysis / integrin-mediated signaling pathway / negative regulation of angiogenesis | metalloendopeptidase activity / protein binding / heparin binding / heparin binding / peptidase activity / metallopeptidase activity / zinc ion binding / hydrolase activity / metal ion binding | extracellular region / proteinaceous extracellular matrix / basement membrane / extracellular matrix / extracellular matrix / cytoplasmic vesicle |
| 1452595_at | 0.80 | 3.66 | 0.43 | -1.53 | 0.03 | -6.26 | -0.23 | -4.41 | Adamts4 | a disintegrin-like and metallopeptidase (reprolysin type) with thrombospondin type 1 motif, 4 | skeletal development / proteolysis / proteolysis / proteolysis / integrin-mediated signaling pathway | peptidase activity / metallopeptidase activity / zinc ion binding / hydrolase activity / hydrolase activity / metal ion binding | extracellular region / proteinaceous extracellular matrix / proteinaceous extracellular matrix / extracellular matrix |
| 1455965_at | 1.16 | 5.38 | 0.28 | -4.53 | -0.02 | -6.30 | -0.03 | -6.08 | Adamts4 | a disintegrin-like and metallopeptidase (reprolysin type) with thrombospondin type 1 motif, 4 | skeletal development / proteolysis / proteolysis / proteolysis / integrin-mediated signaling pathway | peptidase activity / metallopeptidase activity / zinc ion binding / hydrolase activity / hydrolase activity / metal ion binding | extracellular region / proteinaceous extracellular matrix / proteinaceous extracellular matrix / extracellular matrix |
| 1420336_at | 0.22 | -4.93 | 0.33 | -3.19 | 0.62 | 1.08 | 0.48 | -0.81 | Adamtsl5 | ADAMTS-like 5 | --- | metallopeptidase activity / zinc ion binding | proteinaceous extracellular matrix / extracellular space / extracellular matrix |
| 1421058_at | 0.23 | -5.10 | 0.77 | 1.64 | 0.74 | 1.32 | 0.39 | -2.98 | Adh7 | alcohol dehydrogenase 7 (class IV), mu or sigma polypeptide | ethanol catabolic process / metabolic process / retinol metabolic process / retinoic acid metabolic process | catalytic activity / alcohol dehydrogenase activity / alcohol dehydrogenase activity / alcohol dehydrogenase activity / retinol dehydrogenase activity / binding / zinc ion binding / oxidoreductase activity / oxidoreductase activity / metal ion binding | cytoplasm |
| 1416319_at | -0.70 | 1.11 | -0.52 | -1.18 | 0.05 | -6.25 | -0.07 | -5.97 | Adk | adenosine kinase | purine ribonucleoside salvage / purine ribonucleoside salvage | magnesium ion binding / adenosine kinase activity / kinase activity / transferase activity / metal ion binding | nucleus |
| 1452619_a_at | -0.72 | 0.92 | -0.95 | 3.47 | -0.34 | -3.82 | -0.05 | -6.04 | Agbl3 | ATP/GTP binding protein-like 3 | proteolysis | carboxypeptidase activity / carboxypeptidase A activity / peptidase activity / metallopeptidase activity / zinc ion binding / hydrolase activity / metal ion binding | cytoplasm |
| 1457042_at | -0.45 | -1.38 | -0.28 | -3.90 | -0.48 | -0.85 | -0.61 | 1.08 | AI256396 | EST AI256396 | --- | --- | --- |
| 1460406_at | 0.08 | -6.33 | -0.39 | -3.82 | -0.68 | -0.63 | -0.86 | 1.28 | AI427122 | expressed sequence AI427122 | --- | actin binding / calcium ion binding | --- |
| 1435417_at | -0.62 | 0.10 | 0.03 | -6.25 | -0.23 | -4.91 | -0.03 | -6.08 | AI464131 | expressed sequence AI464131 | carbohydrate metabolic process / metabolic process | hydrolase activity, hydrolyzing O-glycosyl compounds / hydrolase activity / hydrolase activity, acting on glycosyl bonds | membrane / integral to membrane |
| 1433897_at | -0.28 | -4.34 | -0.57 | -0.18 | -0.66 | 1.05 | -0.48 | -1.37 | AI597468 | expressed sequence AI597468 | --- | --- | membrane / integral to membrane |
| 1417247_at | 0.78 | 6.08 | 0.35 | -1.03 | 0.49 | 1.59 | 0.18 | -4.31 | AI597479 | expressed sequence AI597479 | --- | --- | --- |
| 1442726_s_at | 2.03 | 9.82 | 1.18 | 4.32 | 0.82 | 0.95 | 0.53 | -2.13 | AI845619 | expressed sequence AI845619 | --- | --- | --- |
| 1418127_a_at | -0.51 | -1.00 | -0.62 | 0.61 | -0.35 | -3.10 | -0.22 | -4.66 | Aifm1 | apoptosis-inducing factor, mitochondrion-associated 1 | DNA fragmentation during apoptosis / apoptosis / apoptotic mitochondrial changes / cell redox homeostasis | DNA binding / electron-transferring-flavoprotein dehydrogenase activity / electron carrier activity / oxidoreductase activity / FAD binding | soluble fraction / nucleus / cytoplasm / mitochondrion / mitochondrial outer membrane / mitochondrial intermembrane space / microsome / cytosol |
| 1449168_a_at | 1.00 | 0.44 | 0.54 | -3.45 | 0.38 | -4.73 | 0.29 | -5.19 | Akap2 / Palm2 / Palm2-akap2 | A kinase (PRKA) anchor protein 2 / paralemmin 2 / Palm2-Akap2 protein | actin filament organization / transmembrane receptor protein serine/threonine kinase signaling pathway / protein localization / regulation of cell shape / regulation of cell shape | protein binding / kinase activity / protein kinase A binding | membrane |
| 1455870_at | 0.97 | 2.40 | 0.32 | -4.51 | 0.51 | -2.45 | 0.13 | -5.77 | Akap2 / Palm2 / Palm2-akap2 | A kinase (PRKA) anchor protein 2 / paralemmin 2 / Palm2-Akap2 protein | actin filament organization / transmembrane receptor protein serine/threonine kinase signaling pathway / protein localization / regulation of cell shape / regulation of cell shape | protein binding / kinase activity / protein kinase A binding | membrane |
| 1425260_at | -0.22 | -5.60 | -0.36 | -4.18 | -0.72 | -0.31 | -0.75 | 0.05 | Alb | albumin | transport / cellular response to starvation / response to organic substance / hemolysis by symbiont of host red blood cells / negative regulation of apoptosis / positive regulation of circadian sleep/wake cycle, non-REM sleep / response to mercury ion / maintenance of mitochondrion location | DNA binding / copper ion binding / protein binding / drug binding / zinc ion binding / lipid binding / toxin binding / oxygen binding / pyridoxal phosphate binding / cell surface binding / metal ion binding | extracellular region / basement membrane / extracellular space / cytoplasm / protein complex |
| 1437466_at | -0.30 | -4.80 | -0.74 | 0.11 | -0.59 | -1.51 | -0.50 | -2.42 | Alcam | activated leukocyte cell adhesion molecule | cell adhesion / axon guidance / motor axon guidance | protein binding | external side of plasma membrane / membrane / integral to membrane / axon / cell soma |
| 1437467_at | -0.07 | -6.36 | -0.42 | -3.64 | -0.82 | 0.71 | -0.51 | -2.52 | Alcam | activated leukocyte cell adhesion molecule | cell adhesion / axon guidance / motor axon guidance | protein binding | external side of plasma membrane / membrane / integral to membrane / axon / cell soma |
| 1415836_at | 0.61 | 1.42 | 0.63 | 1.81 | 0.64 | 1.94 | 0.36 | -2.19 | Aldh18a1 | aldehyde dehydrogenase 18 family, member A1 | proline biosynthetic process / metabolic process / amino acid biosynthetic process / amino acid biosynthetic process | catalytic activity / glutamate 5-kinase activity /glutamate-5-semialdehyde dehydrogenase activity / kinase activity / oxidoreductase activity / transferase activity | cytoplasm / mitochondrion / mitochondrial inner membrane / membrane |
| 1435855_x_at | 0.75 | 1.91 | 0.75 | 1.91 | 0.53 | -0.79 | 0.34 | -3.33 | Aldh18a1 | aldehyde dehydrogenase 18 family, member A1 | proline biosynthetic process / metabolic process / amino acid biosynthetic process / amino acid biosynthetic process | catalytic activity / glutamate 5-kinase activity / glutamate-5-semialdehyde dehydrogenase activity / kinase activity / oxidoreductase activity / transferase activity | cytoplasm / mitochondrion / mitochondrial inner membrane / membrane |
| 1437325_x_at | 0.89 | 3.44 | 0.80 | 2.36 | 0.65 | 0.58 | 0.36 | -3.21 | Aldh18a1 | aldehyde dehydrogenase 18 family, member A1 | proline biosynthetic process / metabolic process / amino acid biosynthetic process / amino acid biosynthetic process | catalytic activity / glutamate 5-kinase activity / glutamate-5-semialdehyde dehydrogenase activity / kinase activity / oxidoreductase activity / transferase activity | cytoplasm / mitochondrion / mitochondrial inner membrane / membrane |
| 1437333_x_at | 0.67 | 0.11 | 0.63 | -0.36 | 0.59 | -0.84 | 0.30 | -4.17 | Aldh18a1 | aldehyde dehydrogenase 18 family, member A1 | proline biosynthetic process / metabolic process / amino acid biosynthetic process / amino acid biosynthetic process | catalytic activity / glutamate 5-kinase activity / glutamate-5-semialdehyde dehydrogenase activity / kinase activity / oxidoreductase activity / transferase activity | cytoplasm / mitochondrion / mitochondrial inner membrane / membrane |
| 1418601_at | -0.73 | 0.31 | -0.54 | -1.79 | -0.02 | -6.30 | -0.19 | -5.33 | Aldh1a7 | aldehyde dehydrogenase family 1, subfamily A7 | metabolic process / metabolic process / retinal metabolic process | aldehyde dehydrogenase (NAD) activity / oxidoreductase activity | cytoplasm |
| 1436119_at | 0.65 | -0.17 | 1.18 | 5.15 | 1.33 | 6.39 | 0.66 | -0.02 | Aldh1l2 | aldehyde dehydrogenase 1 family, member L2 | one-carbon compound metabolic process / metabolic process / biosynthetic process / 10-formyltetrahydrofolate catabolic process | catalytic activity / methyltransferase activity / formyltetrahydrofolate dehydrogenase activity / oxidoreductase activity / hydroxymethyl-, formyl- and related transferase activity / phosphopantetheine binding / cofactor binding | cytoplasm |
| 1451461_a_at | -0.96 | -4.93 | -1.22 | -4.03 | -2.80 | 1.12 | -1.98 | -1.44 | Aldoc | aldolase 3, C isoform | glycolysis / metabolic process | catalytic activity / fructose-bisphosphate aldolase activity / protein binding / lyase activity | mitochondrion |
| 1439007_at | -0.36 | -1.91 | -0.61 | 2.39 | -0.43 | -0.49 | -0.27 | -3.27 | Alg6 | asparagine-linked glycosylation 6 homolog (yeast, alpha-1,3,-glucosyltransferase) | protein amino acid N-linked glycosylation | transferase activity / transferase activity, transferring glycosyl groups / glucosyltransferase activity | endoplasmic reticulum / endoplasmic reticulum membrane / membrane / integral to membrane |
| 1433544_at | -0.78 | 2.62 | -0.13 | -5.78 | -0.07 | -6.14 | -0.12 | -5.62 | Als2cr2 | amyotrophic lateral sclerosis 2 (juvenile) chromosome region, candidate 2 (human) | cell morphogenesis / protein amino acid phosphorylation / protein export from nucleus / anti-apoptosis / cytoskeleton organization and biogenesis / cell cycle / JNK cascade / activation of protein kinase activity | nucleotide binding / protein kinase activity / protein serine/threonine kinase activity / ATP binding | nucleus / cytoplasm / cytoplasm |
| 1434443_at | -0.59 | 1.00 | -0.10 | -5.91 | -0.21 | -4.73 | -0.08 | -5.85 | Anapc1 | anaphase promoting complex subunit 1 | ubiquitin cycle / cell cycle / mitosis / cell division | protein binding | nucleus |
| 1439066_at | -1.66 | 2.96 | -1.16 | -0.12 | -0.82 | -2.49 | -0.95 | -1.50 | Angpt1 | angiopoietin 1 | angiogenesis / signal transduction / transmembrane receptor protein tyrosine kinase signaling pathway / multicellular organismal development / endoderm development / cell differentiation | receptor binding / vascular endothelial growth factor receptor binding | extracellular region / extracellular space |
| 1417130_s_at | 0.19 | -5.40 | -0.24 | -4.70 | -0.72 | 1.54 | -0.37 | -2.95 | Angptl4 | angiopoietin-like 4 | signal transduction / cellular response to starvation / negative regulation of apoptosis / negative regulation of apoptosis / positive regulation of lipid metabolic process / negative regulation of lipoprotein lipase activity | enzyme inhibitor activity / enzyme inhibitor activity / receptor binding | extracellular region / extracellular region / proteinaceous extracellular matrix / extracellular space |
| 1424785_at | 0.55 | -2.24 | 0.61 | -1.45 | 1.09 | 3.36 | 0.76 | 0.25 | Angptl6 | angiopoietin-like 6 | angiogenesis / signal transduction / multicellular organismal development / cell differentiation | receptor binding | extracellular region / extracellular space / secretory granule |
| 1452124_at | -0.25 | -4.54 | -0.28 | -4.04 | -0.26 | -4.31 | -0.66 | 1.30 | Ank3 | ankyrin 3, epithelial | signal transduction / axon guidance / synapse organization and biogenesis | protein binding | axon / synapse |
| 1451724_at | -0.68 | 1.75 | -0.02 | -6.26 | -0.01 | -6.30 | -0.12 | -5.59 | Ankmy2 | ankyrin repeat and MYND domain containing 2 | --- | zinc ion binding / metal ion binding | --- |
| 1443867_at | 0.69 | -1.55 | 0.85 | 0.05 | 0.93 | 0.77 | 0.91 | 0.55 | Ankrd12-like / Ankrd12 | ankyrin repeat domain 12-like / ankyrin repeat domain 12 | --- | --- | --- |
| 1436538_at | -1.17 | -2.69 | -1.48 | -1.09 | -1.78 | 0.29 | -0.95 | -3.49 | Ankrd37 | ankyrin repeat domain 37 | --- | --- | nucleus / cytoplasm |
| 1452938_at | -0.37 | -1.82 | -0.61 | 2.10 | -0.26 | -3.65 | -0.36 | -1.84 | Anks1b | ankyrin repeat and sterile alpha motif domain containing 1B | --- | --- | nucleus / cytoplasm / membrane / cell junction / cell projection / synapse / postsynaptic membrane |
| 1460449_at | -1.03 | 3.54 | -1.01 | 3.34 | -0.74 | 0.55 | -0.43 | -3.00 | Anks1b | ankyrin repeat and sterile alpha motif domain containing 1B | --- | --- | nucleus / cytoplasm / membrane / cell junction / cell projection / synapse / postsynaptic membrane |
| 1451446_at | -0.65 | 0.14 | -0.03 | -6.26 | -0.47 | -2.12 | -0.20 | -5.12 | Antxr1 | anthrax toxin receptor 1 | --- | receptor activity / metal ion binding | extracellular space / plasma membrane / membrane / integral to membrane / integral to membrane |
| 1424176_a_at | -0.44 | -3.26 | -0.58 | -1.51 | -0.53 | -2.11 | -0.73 | 0.24 | Anxa4 | annexin A4 | kidney development | calcium ion binding / calcium-dependent phospholipid binding | apical plasma membrane |
| 1457658_x_at | -0.52 | -2.03 | -0.55 | -1.56 | -0.37 | -3.74 | -0.73 | 0.59 | Anxa4 | annexin A4 | kidney development | calcium ion binding / calcium-dependent phospholipid binding | apical plasma membrane |
| 1439214_a_at | 0.51 | -0.11 | 0.23 | -4.44 | 0.59 | 1.22 | 0.36 | -2.23 | Api5 | apoptosis inhibitor 5 | apoptosis / anti-apoptosis | binding / fibroblast growth factor binding / fibroblast growth factor binding | nucleus / spliceosome / cytoplasm |
| 1451755_a_at | 0.96 | 5.66 | 0.56 | 0.67 | 0.20 | -4.84 | -0.20 | -4.73 | Apobec1 | apolipoprotein B editing complex 1 | mRNA processing / response to gamma radiation / cytidine to uridine editing / mRNA modification / lipoprotein metabolic process / lipoprotein biosynthetic process / lipoprotein transport / mRNA stabilization | catalytic activity / cytidine deaminase activity / protein binding / zinc ion binding / hydrolase activity / hydrolase activity, acting on carbon-nitrogen (but not peptide) bonds, in cyclic amidines / AU-rich element binding / metal ion binding | --- |
| 1418708_at | -0.17 | -5.46 | -0.31 | -3.54 | -0.77 | 2.91 | -0.35 | -2.80 | Apoc4 | apolipoprotein C-IV | transport / lipid transport | --- | extracellular region / extracellular space |
| 1426743_at | -0.10 | -6.42 | -0.78 | -4.76 | -2.26 | 1.16 | -1.34 | -2.49 | Appl2 | adaptor protein, phosphotyrosine interaction, PH domain and leucine zipper containing 2 | cell cycle / cell proliferation / cell proliferation / cell proliferation | protein binding / protein binding / Rab GTPase binding | nucleus / cytoplasm / endosome / endosome membrane / membrane / NuRD complex / early endosome membrane |
| 1429254_at | -1.38 | 3.39 | -1.19 | 1.93 | -1.18 | 1.87 | -0.69 | -2.09 | Aqp11 | aquaporin 11 | kidney development / transport / endosomal lumen acidification | transporter activity / peptidase activity | cytoplasm / endoplasmic reticulum / plasma membrane / membrane / integral to membrane |
| 1426534_a_at | -0.41 | -3.56 | -0.41 | -3.41 | -0.76 | 0.50 | -0.56 | -1.63 | Arfgap3 / LOC100045474 | ADP-ribosylation factor GTPase activating protein 3 / similar to ADP-ribosylation factor GTPase activating protein 3 | transport / protein secretion / protein transport / vesicle-mediated transport / regulation of ARF GTPase activity | GTPase activator activity / ARF GTPase activator activity / zinc ion binding / metal ion binding | Golgi membrane / cytoplasm / Golgi apparatus / cytosol / membrane |
| 1415711_at | -0.35 | -1.97 | -0.19 | -4.65 | -0.31 | -2.69 | -0.65 | 2.90 | Arfgef1 | ADP-ribosylation factor guanine nucleotide-exchange factor 1(brefeldin A-inhibited) | regulation of ARF protein signal transduction | ARF guanyl-nucleotide exchange factor activity / binding | intracellular |
| 1425508_s_at | 0.68 | 0.99 | 0.52 | -1.09 | 0.45 | -2.01 | 0.36 | -3.14 | Arfrp1 | ADP-ribosylation factor related protein 1 | small GTPase mediated signal transduction / gastrulation | nucleotide binding / GTPase activity / GTP binding | intracellular / membrane fraction / Golgi apparatus |
| 1434488_at | 0.67 | 1.31 | 0.27 | -4.31 | 0.62 | 0.65 | 0.31 | -3.54 | Arfrp1 | ADP-ribosylation factor related protein 1 | small GTPase mediated signal transduction / gastrulation | nucleotide binding / GTPase activity / GTP binding | intracellular / membrane fraction / Golgi apparatus |
| 1426952_at | -0.83 | 2.03 | -0.68 | 0.41 | -0.28 | -4.60 | -0.55 | -1.16 | Arhgap18 | Rho GTPase activating protein 18 | signal transduction | GTPase activator activity | intracellular |
| 1450896_at | -0.34 | -4.61 | -0.79 | 0.33 | -0.06 | -6.24 | -0.32 | -4.43 | Arhgap5 | Rho GTPase activating protein 5 | positive regulation of mesenchymal cell proliferation / signal transduction / small GTPase mediated signal transduction / Rho protein signal transduction / regulation of cell size / positive regulation of cell migration / mammary gland development | GTPase activator activity / Rho GTPase activator activity / GTP binding | intracellular / cytoplasm / membrane |
| 1424249_a_at | -0.62 | 1.61 | -0.22 | -4.51 | 0.14 | -5.50 | 0.11 | -5.64 | Arhgap9 | Rho GTPase activating protein 9 | signal transduction | GTPase activator activity | intracellular |
| 1436097_x_at | -0.59 | 0.60 | -0.14 | -5.61 | 0.11 | -5.90 | 0.07 | -5.90 | Arhgap9 | Rho GTPase activating protein 9 | signal transduction | GTPase activator activity | intracellular |
| 1452302_at | -0.56 | -2.14 | -0.63 | -1.22 | -0.86 | 1.15 | -0.42 | -3.42 | Arhgef10 | Rho guanine nucleotide exchange factor (GEF) 10 | proteolysis / intracellular signaling cascade / regulation of Rho protein signal transduction | cysteine-type endopeptidase activity / guanyl-nucleotide exchange factor activity / Rho guanyl-nucleotide exchange factor activity | intracellular |
| 1421043_s_at | 0.25 | -4.92 | 0.70 | 0.76 | 0.68 | 0.50 | 0.49 | -1.78 | Arhgef2 | rho/rac guanine nucleotide exchange factor (GEF) 2 | cell morphogenesis / actin filament organization / actin filament organization / negative regulation of microtubule depolymerization / intracellular signaling cascade / regulation of Rho protein signal transduction / regulation of Rho protein signal transduction | guanyl-nucleotide exchange factor activity / Rho guanyl-nucleotide exchange factor activity / protein binding / microtubule binding / zinc ion binding / Rho GTPase binding / diacylglycerol binding / Rac guanyl-nucleotide exchange factor activity / metal ion binding / Rac GTPase binding | intracellular / cytoplasm / microtubule / cell soma / dendritic shaft |
| 1453590_at | 0.71 | 1.71 | 0.26 | -4.35 | 0.52 | -0.87 | 0.39 | -2.52 | Arl5b | ADP-ribosylation factor-like 5B | small GTPase mediated signal transduction | nucleotide binding / GTP binding | intracellular |
| 1424759_at | 0.90 | 2.84 | 0.75 | 1.15 | 0.82 | 2.00 | 0.61 | -0.47 | Arrdc4 | arrestin domain containing 4 | --- | --- | --- |
| 1426818_at | 0.93 | 2.45 | 0.75 | 0.55 | 0.81 | 1.19 | 0.53 | -1.92 | Arrdc4 | arrestin domain containing 4 | --- | --- | --- |
| 1427002_s_at | -0.63 | -0.57 | -0.40 | -3.28 | -0.80 | 1.46 | -0.88 | 2.28 | Arsg | arylsulfatase G | metabolic process | catalytic activity / arylsulfatase activity / dolichol kinase activity / calcium ion binding / sulfuric ester hydrolase activity / hydrolase activity / phosphoric monoester hydrolase activity / metal ion binding | endoplasmic reticulum |
| 1452277_at | -0.77 | -1.27 | -0.76 | -1.21 | -0.92 | 0.20 | -0.80 | -0.79 | Arsg | arylsulfatase G | metabolic process | catalytic activity / arylsulfatase activity / dolichol kinase activity / calcium ion binding / sulfuric ester hydrolase activity / hydrolase activity / phosphoric monoester hydrolase activity / metal ion binding | endoplasmic reticulum |
| 1434961_at | -0.73 | 1.00 | -0.51 | -1.68 | -0.44 | -2.57 | -0.45 | -2.29 | Asb1 | ankyrin repeat and SOCS box-containing protein 1 | ubiquitin cycle / intracellular signaling cascade / multicellular organismal development / male genitalia development | --- | --- |
| 1427156_s_at | 0.21 | -5.08 | 0.32 | -3.55 | 0.64 | 0.93 | 0.22 | -4.75 | Ascc2 | activating signal cointegrator 1 complex subunit 2 | transcription / regulation of transcription, DNA-dependent | --- | --- |
| 1457069_at | -0.46 | -1.99 | -0.71 | 1.36 | -0.50 | -1.38 | -0.54 | -0.75 | Ascc3 | activating signal cointegrator 1 complex subunit 3 | --- | nucleic acid binding / helicase activity / ATP binding / ATP-dependent helicase activity / hydrolase activity | --- |
| 1418472_at | 0.83 | 0.17 | -0.23 | -5.47 | -0.39 | -4.18 | -0.48 | -3.16 | Aspa | aspartoacylase | acetate metabolic process / aspartate catabolic process / metabolic process | aminoacylase activity / hydrolase activity / hydrolase activity, acting on ester bonds / aspartoacylase activity | --- |
| 1426015_s_at | -0.34 | -2.18 | -0.34 | -2.10 | -0.46 | 0.03 | -0.60 | 2.15 | Asph | aspartate-beta-hydroxylase | peptidyl-amino acid modification | peptide-aspartate beta-dioxygenase activity / binding / iron ion binding / oxidoreductase activity / oxidoreductase activity, acting on single donors with incorporation of molecular oxygen, incorporation of two atoms of oxygen | endoplasmic reticulum / endoplasmic reticulum membrane / membrane / integral to membrane / integral to endoplasmic reticulum membrane |
| 1426583_at | 0.93 | 3.41 | 0.21 | -5.22 | -0.10 | -6.05 | -0.39 | -3.02 | Atf2 / LOC100047997 | activating transcription factor 2 / similar to Cyclic AMP-dependent transcription factor ATF-2 (Activating transcription factor 2) (cAMP response element-binding protein CRE-BP1) (MXBP protein) | transcription / regulation of transcription, DNA-dependent | nucleic acid binding / DNA binding / transcription factor activity / protein binding / zinc ion binding / sequence-specific DNA binding / metal ion binding / protein dimerization activity | intracellular / membrane fraction / nucleus / nucleus / transcription factor complex |
| 1452116_s_at | 0.70 | 4.11 | 0.23 | -3.83 | 0.07 | -6.04 | -0.05 | -5.98 | Atf2 / LOC100047997 | activating transcription factor 2 / similar to Cyclic AMP-dependent transcription factor ATF-2 (Activating transcription factor 2) (cAMP response element-binding protein CRE-BP1) (MXBP protein) | transcription / regulation of transcription, DNA-dependent | nucleic acid binding / DNA binding / transcription factor activity / protein binding / zinc ion binding / sequence-specific DNA binding / metal ion binding / protein dimerization activity | intracellular / membrane fraction / nucleus / nucleus / transcription factor complex |
| 1449363_at | 2.08 | 3.84 | 1.51 | 0.92 | 1.53 | 1.02 | 0.88 | -2.74 | Atf3 | activating transcription factor 3 | gluconeogenesis / transcription / regulation of transcription, DNA-dependent | DNA binding / transcription factor activity / transcription repressor activity / sequence-specific DNA binding / protein dimerization activity | nucleus / transcription factor complex |
| 1438992_x_at | 0.99 | 6.55 | 0.67 | 2.66 | 0.57 | 1.29 | 0.38 | -1.70 | Atf4 | activating transcription factor 4 | gluconeogenesis / transcription / regulation of transcription, DNA-dependent / positive regulation of transcription from RNA polymerase II promoter | DNA binding / transcription factor activity / specific RNA polymerase II transcription factor activity / protein binding / transcription activator activity / sequence-specific DNA binding / protein dimerization activity | nucleus / transcription factor complex / cytoplasm / plasma membrane / membrane |
| 1448135_at | 0.91 | 3.76 | 0.94 | 4.01 | 0.56 | -0.54 | 0.30 | -3.89 | Atf4 | activating transcription factor 4 | gluconeogenesis / transcription / regulation of transcription, DNA-dependent / positive regulation of transcription from RNA polymerase II promoter | DNA binding / transcription factor activity / specific RNA polymerase II transcription factor activity / protein binding / transcription activator activity / sequence-specific DNA binding / protein dimerization activity | nucleus / transcription factor complex / cytoplasm / plasma membrane / membrane |
| 1425927_a_at | 0.93 | 2.06 | 0.84 | 1.11 | 0.67 | -0.72 | 0.77 | 0.44 | Atf5 | activating transcription factor 5 | transcription / regulation of transcription, DNA-dependent / regulation of transcription from RNA polymerase II promoter / anti-apoptosis / regulation of cell proliferation / regulation of cell cycle | DNA binding / transcription factor activity / RNA polymerase II transcription factor activity / sequence-specific DNA binding / protein dimerization activity | nucleus / transcription factor complex / transcription factor complex / cytoplasm |
| 1456021_at | 0.66 | 0.24 | 0.47 | -2.06 | 0.16 | -5.69 | 0.21 | -5.06 | Atf6 | activating transcription factor 6 | regulation of transcription, DNA-dependent / regulation of transcription from RNA polymerase II promoter / protein folding / response to stress / response to unfolded protein / positive regulation of gene-specific transcription involved in unfolded protein response / signal transduction | DNA binding / transcription factor activity / RNA polymerase II transcription factor activity / transcription coactivator activity / sequence-specific DNA binding / protein dimerization activity | nucleus / nuclear envelope lumen / nucleoplasm / endoplasmic reticulum / endoplasmic reticulum membrane / integral to membrane |
| 1447272_s_at | -0.51 | -1.74 | -0.69 | 0.67 | -0.81 | 2.10 | -0.45 | -2.30 | Atp10a | ATPase, class V, type 10A | transport / phospholipid transport | nucleotide binding / magnesium ion binding / phospholipid-translocating ATPase activity / ATP binding / ATPase activity, coupled to transmembrane movement of ions, phosphorylative mechanism / hydrolase activity / metal ion binding | membrane / integral to membrane |
| 1447851_x_at | -0.43 | -0.42 | -0.70 | 3.82 | -0.50 | 0.72 | -0.43 | -0.31 | Atp10a | ATPase, class V, type 10A | transport / phospholipid transport | nucleotide binding / magnesium ion binding / phospholipid-translocating ATPase activity / ATP binding / ATPase activity, coupled to transmembrane movement of ions, phosphorylative mechanism / hydrolase activity / metal ion binding | membrane / integral to membrane |
| 1452013_at | -0.53 | 0.35 | -0.74 | 3.40 | -0.60 | 1.53 | -0.51 | 0.19 | Atp10a | ATPase, class V, type 10A | transport / phospholipid transport | nucleotide binding / magnesium ion binding / phospholipid-translocating ATPase activity / ATP binding / ATPase activity, coupled to transmembrane movement of ions, phosphorylative mechanism / hydrolase activity / metal ion binding | membrane / integral to membrane |
| 1455083_at | -0.48 | -3.11 | -0.63 | -1.43 | -0.62 | -1.48 | -0.83 | 0.72 | Atp11c | Atpase, class VI, type 11C | transport / metabolic process / phospholipid transport | nucleotide binding / magnesium ion binding / catalytic activity / phospholipid-translocating ATPase activity / ATP binding / ATPase activity, coupled to transmembrane movement of ions, phosphorylative mechanism / hydrolase activity / metal ion binding | membrane / integral to membrane |
| 1427251_at | -0.65 | 1.49 | -0.23 | -4.54 | -0.29 | -3.80 | -0.27 | -3.88 | Atp2a2 | ATPase, Ca++ transporting, cardiac muscle, slow twitch 2 | regulation of the force of heart contraction / transport / ion transport / cation transport / calcium ion transport / cellular calcium ion homeostasis / regulation of muscle contraction / ER-nuclear signaling pathway / metabolic process / proton transport / negative regulation of heart contraction | nucleotide binding / magnesium ion binding / catalytic activity / calcium-transporting ATPase activity / calcium ion binding / protein binding / ATP binding / ATPase activity, coupled to transmembrane movement of ions, phosphorylative mechanism / hydrolase activity / hydrolase activity, acting on acid anhydrides, catalyzing transmembrane movement of substances / metal ion binding | endoplasmic reticulum / endoplasmic reticulum membrane / microsome / membrane / integral to membrane / sarcoplasmic reticulum / sarcoplasmic reticulum membrane |
| 1428937_at | 0.60 | 0.16 | 0.49 | -1.29 | 0.08 | -6.13 | 0.10 | -5.77 | Atp2b1 | ATPase, Ca++ transporting, plasma membrane 1 | transport / cation transport / calcium ion transport / calcium ion transport / metabolic process | nucleotide binding / catalytic activity / calcium-transporting ATPase activity / calcium ion binding / protein binding / ATP binding / calcium ion transmembrane transporter activity / ATPase activity, coupled to transmembrane movement of ions, phosphorylative mechanism / hydrolase activity / hydrolase activity, acting on acid anhydrides, catalyzing transmembrane movement of substances | integral to plasma membrane / membrane / integral to membrane |
| 1416769_s_at | 0.44 | -3.16 | 0.63 | -0.74 | 0.62 | -0.85 | 0.79 | 0.99 | Atp6v0b | ATPase, H+ transporting, lysosomal V0 subunit B | ATP biosynthetic process / ATP biosynthetic process / transport / ion transport / ATP synthesis coupled proton transport / ATP hydrolysis coupled proton transport / proton transport / proton transport | ATP binding / hydrogen-exporting ATPase activity, phosphorylative mechanism / hydrogen ion transmembrane transporter activity / hydrogen ion transmembrane transporter activity / hydrolase activity / ATPase activity, coupled to transmembrane movement of substances / metal ion binding / hydrogen ion transporting ATP synthase activity, rotational mechanism / hydrogen ion transporting ATPase activity, rotational mechanism | endosome / vacuole / vacuolar membrane / membrane / membrane / integral to membrane / integral to membrane / integral to membrane / proton-transporting two-sector ATPase complex / vacuolar proton-transporting V-type ATPase complex |
| 1437013_x_at | 0.52 | -1.29 | 0.70 | 1.23 | 0.59 | -0.24 | 0.78 | 2.04 | Atp6v0b | ATPase, H+ transporting, lysosomal V0 subunit B | ATP biosynthetic process / ATP biosynthetic process / transport / ion transport / ATP synthesis coupled proton transport / ATP hydrolysis coupled proton transport / proton transport | ATP binding / hydrogen-exporting ATPase activity, phosphorylative mechanism / hydrogen ion transmembrane transporter activity / hydrogen ion transmembrane transporter activity / hydrolase activity / ATPase activity, coupled to transmembrane movement of substances / metal ion binding / hydrogen ion transporting ATP synthase activity, rotational mechanism / hydrogen ion transporting ATPase activity, rotational mechanism | endosome / vacuole / vacuolar membrane / membrane / membrane / integral to membrane / integral to membrane / integral to membrane / proton-transporting two-sector ATPase complex / vacuolar proton-transporting V-type ATPase complex |
| 1436921_at | -0.53 | -1.99 | -0.69 | 0.06 | -0.68 | -0.08 | -0.48 | -2.34 | Atp7a | ATPase, Cu++ transporting, alpha polypeptide | blood vessel development / blood vessel remodeling / regulation of oxidative phosphorylation / tryptophan metabolic process / tyrosine metabolic process / catecholamine metabolic process / transport / ion transport / copper ion transport / cellular copper ion homeostasis / mitochondrion organization and biogenesis / locomotory behavior / metabolic process / detoxification of copper ion / copper ion import / mercury ion transport / peptidyl-lysine modification / removal of superoxide radicals / cerebellar Purkinje cell differentiation / pyramidal neuron development / central nervous system neuron development / metal ion transport / extracellular matrix organization and biogenesis / collagen fibril organization / hair follicle morphogenesis / hindlimb morphogenesis / T-helper cell differentiation / epinephrine metabolic process / norepinephrine metabolic process / dopamine metabolic process / norepinephrine biosynthetic process / serotonin metabolic process / positive regulation of catalytic activity / pigmentation / neuroprotection / skin development / elastic fiber assembly / alveolus development / negative regulation of metalloenzyme activity / positive regulation of metalloenzyme activity / neurite morphogenesis / dendrite morphogenesis / cartilage development / elastin biosynthetic process / copper ion export | nucleotide binding / magnesium ion binding / catalytic activity / copper-exporting ATPase activity / copper ion transmembrane transporter activity / copper ion transmembrane transporter activity / copper ion binding / protein binding / ATP binding / mercury ion transmembrane transporter activity / ATPase activity, coupled to transmembrane movement of ions, phosphorylative mechanism / superoxide dismutase copper chaperone activity hydrolase activity, acting on acid anhydrides, catalyzing transmembrane movement of substances / metal ion binding / metal ion transmembrane transporter activity | integral to membrane of membrane fraction / membrane fraction / Golgi apparatus / trans-Golgi network / trans-Golgi network / plasma membrane / plasma membrane / membrane / membrane / integral to membrane / trans-Golgi network transport vesicle / cytoplasmic vesicle / neuron projection / cell soma |
| 1423597_at | -0.31 | -5.23 | -0.71 | -1.52 | -1.54 | 5.02 | -1.06 | 1.51 | Atp8a1 | ATPase, aminophospholipid transporter (APLT), class I, type 8A, member 1 | transport / metabolic process / phospholipid transport | nucleotide binding / magnesium ion binding / catalytic activity / phospholipid-translocating ATPase activity / ATP binding / ATPase activity, coupled to transmembrane movement of ions, phosphorylative mechanism / hydrolase activity / hydrolase activity, acting on acid anhydrides, catalyzing transmembrane movement of substances / metal ion binding | membrane / integral to membrane / cytoplasmic vesicle |
| 1433827_at | -0.03 | -6.42 | -0.30 | -4.10 | -0.82 | 2.37 | -0.81 | 2.19 | Atp8a1 | ATPase, aminophospholipid transporter (APLT), class I, type 8A, member 1 | transport / metabolic process / phospholipid transport | nucleotide binding / magnesium ion binding / catalytic activity / phospholipid-translocating ATPase activity / ATP binding / ATPase activity, coupled to transmembrane movement of ions, phosphorylative mechanism / hydrolase activity / hydrolase activity, acting on acid anhydrides, catalyzing transmembrane movement of substances / metal ion binding | membrane / integral to membrane / cytoplasmic vesicle |
| 1433965_at | -0.42 | -1.87 | -0.43 | -1.62 | -0.68 | 2.03 | -0.40 | -1.90 | Atp8a1 | ATPase, aminophospholipid transporter (APLT), class I, type 8A, member 1 | transport / metabolic process / phospholipid transport | nucleotide binding / magnesium ion binding / catalytic activity / phospholipid-translocating ATPase activity / ATP binding / ATPase activity, coupled to transmembrane movement of ions, phosphorylative mechanism / hydrolase activity / hydrolase activity, acting on acid anhydrides, catalyzing transmembrane movement of substances / metal ion binding | membrane / integral to membrane / cytoplasmic vesicle |
| 1454728_s_at | -0.16 | -6.23 | -0.67 | -3.18 | -1.34 | 1.62 | -1.31 | 1.35 | Atp8a1 | ATPase, aminophospholipid transporter (APLT), class I, type 8A, member 1 | transport / metabolic process / phospholipid transport | nucleotide binding / magnesium ion binding / catalytic activity / phospholipid-translocating ATPase activity / ATP binding / ATPase activity, coupled to transmembrane movement of ions, phosphorylative mechanism / hydrolase activity / hydrolase activity, acting on acid anhydrides, catalyzing transmembrane movement of substances / metal ion binding | membrane / integral to membrane / cytoplasmic vesicle |
| 1418603_at | -1.12 | 4.37 | -0.62 | -0.85 | -0.57 | -1.44 | -0.34 | -3.95 | Avpr1a | arginine vasopressin receptor 1A | regulation of systemic arterial blood pressure by vasopressin / signal transduction / G-protein coupled receptor protein signaling pathway / elevation of cytosolic calcium ion concentration / calcium-mediated signaling / social behavior / cellular response to water deprivation | rhodopsin-like receptor activity / signal transducer activity / receptor activity / G-protein coupled receptor activity / vasopressin receptor activity / peptide hormone binding / V1A vasopressin receptor binding | plasma membrane / membrane / integral to membrane |
| 1418604_at | -0.98 | 2.90 | -0.73 | 0.35 | -0.74 | 0.43 | -0.32 | -4.23 | Avpr1a | arginine vasopressin receptor 1A | regulation of systemic arterial blood pressure by vasopressin / signal transduction / G-protein coupled receptor protein signaling pathway / elevation of cytosolic calcium ion concentration / calcium-mediated signaling / social behavior / cellular response to water deprivation | rhodopsin-like receptor activity / signal transducer activity / receptor activity / G-protein coupled receptor activity / vasopressin receptor activity / peptide hormone binding / V1A vasopressin receptor binding | plasma membrane / membrane / integral to membrane |
| 1434372_at | 0.95 | 0.24 | 0.23 | -5.62 | -0.28 | -5.37 | -0.14 | -5.85 | AW112010 | expressed sequence AW112010 | --- | --- | --- |
| 1421754_at | 0.13 | -6.11 | 0.12 | -5.98 | 0.19 | -5.61 | 0.78 | 0.67 | AY036118 | cDNA sequence AY036118 | positive regulation of cell proliferation / hemopoiesis | --- | --- |
| 1434373_at | -0.84 | 2.57 | -0.28 | -4.34 | -0.45 | -2.27 | -0.28 | -4.27 | B930006L02Rik | RIKEN cDNA B930006L02 gene | --- | --- | --- |
| 1442418_at | -0.11 | -6.32 | -0.33 | -5.12 | -1.25 | 2.21 | -0.16 | -5.82 | B930096F20Rik | RIKEN cDNA B930096F20 gene | --- | --- | --- |
| 1418991_at | -0.07 | -6.25 | -0.27 | -3.92 | 0.64 | 1.67 | 0.18 | -4.93 | Bak1 | BCL2-antagonist/killer 1 | release of cytochrome c from mitochondria / apoptosis / apoptosis / caspase activation / mitochondrial fusion / cell proliferation / caspase activation via cytochrome c / response to fungus / response to mycotoxin / establishment and/or maintenance of transmembrane electrochemical gradient / regulation of apoptosis / regulation of apoptosis / regulation of mitochondrial membrane permeability / regulation of mitochondrial membrane potential | protein binding / protein heterodimerization activity | mitochondrion / mitochondrion / membrane / integral to membrane / pore complex |
| 1434200_at | 0.71 | 0.67 | 0.23 | -5.03 | 0.44 | -2.59 | 0.03 | -6.07 | BC010981 | cDNA sequence BC010981 | --- | --- | --- |
| 1426734_at | -0.29 | -4.22 | -0.72 | 1.62 | -0.46 | -1.85 | -0.46 | -1.74 | BC022623 | cDNA sequence BC022623 | --- | --- | --- |
| 1424940_s_at | 0.60 | -0.54 | 0.66 | 0.38 | 0.88 | 2.78 | 0.58 | -0.66 | BC022687 | cDNA sequence BC022687 | --- | --- | --- |
| 1451533_at | 0.90 | 2.28 | 0.56 | -1.51 | 0.57 | -1.35 | 0.31 | -4.21 | BC022687 | cDNA sequence BC022687 | --- | --- | --- |
| 1437950_at | -0.89 | 1.32 | -0.53 | -2.47 | -0.19 | -5.63 | -0.37 | -4.00 | BC035537 | cDNA sequence BC035537 | --- | --- | --- |
| 1460713_at | 0.02 | -6.44 | 0.36 | -4.14 | 0.76 | 0.26 | 0.52 | -2.28 | BC048355 | cDNA sequence BC048355 | --- | --- | nucleus |
| 1436098_at | -0.52 | -0.30 | -0.97 | 5.34 | -1.07 | 6.33 | -0.51 | -0.34 | Bche | butyrylcholinesterase | --- | carboxylesterase activity / cholinesterase activity / hydrolase activity | nuclear envelope lumen / endoplasmic reticulum |
| 1437863_at | -0.70 | 0.51 | -0.93 | 3.07 | -1.02 | 4.02 | -0.45 | -2.35 | Bche | butyrylcholinesterase | --- | carboxylesterase activity / cholinesterase activity / hydrolase activity | nuclear envelope lumen / endoplasmic reticulum |
| 1426334_a_at | 1.19 | 5.98 | 0.62 | -0.09 | 0.48 | -1.90 | 0.36 | -3.25 | Bcl2l11 | BCL2-like 11 (apoptosis facilitator) | apoptosis / apoptosis / cell-matrix adhesion / mammary gland development / lumen formation / regulation of apoptosis / positive regulation of apoptosis / positive regulation of apoptosis / positive regulation of apoptosis / regulation of organ growth / post-embryonic organ morphogenesis | protein binding / microtubule binding | membrane |
| 1435449_at | 1.11 | 0.93 | 0.72 | -2.16 | 0.16 | -6.04 | 0.02 | -6.09 | Bcl2l11 | BCL2-like 11 (apoptosis facilitator) | apoptosis / apoptosis / cell-matrix adhesion / mammary gland development / lumen formation / regulation of apoptosis / positive regulation of apoptosis / positive regulation of apoptosis / positive regulation of apoptosis / regulation of organ growth / post-embryonic organ morphogenesis | protein binding / microtubule binding | membrane |
| 1456005_a_at | 0.83 | 3.38 | 0.31 | -3.59 | 0.11 | -5.88 | -0.06 | -5.98 | Bcl2l11 | BCL2-like 11 (apoptosis facilitator) | apoptosis / apoptosis / cell-matrix adhesion / mammary gland development / lumen formation / regulation of apoptosis / positive regulation of apoptosis / positive regulation of apoptosis / positive regulation of apoptosis / regulation of organ growth / post-embryonic organ morphogenesis | protein binding / microtubule binding | membrane |
| 1452614_at | 0.65 | -0.90 | 0.41 | -3.49 | 0.74 | 0.19 | 0.67 | -0.48 | Bcl2l15 | Bcl2-like 15 | --- | --- | nucleus / cytosol |
| 1421818_at | 1.06 | 3.78 | 0.03 | -6.26 | 0.42 | -3.22 | 0.15 | -5.65 | Bcl6 | B-cell leukemia/lymphoma 6 | protein import into nucleus, translocation / negative regulation of transcription from RNA polymerase II promoter / negative regulation of transcription from RNA polymerase II promoter / negative regulation of transcription from RNA polymerase II promoter / negative regulation of transcription from RNA polymerase II promoter / negative regulation of transcription from RNA polymerase II promoter / cell morphogenesis / cell morphogenesis / negative regulation of cell-matrix adhesion / germinal center formation / negative regulation of T-helper 2 type immune response / transcription / regulation of transcription, DNA-dependent / response to DNA damage stimulus / Rho protein signal transduction / spermatogenesis / protein localization / negative regulation of cell proliferation / actin cytoskeleton organization and biogenesis / B cell differentiation / negative regulation of cell growth / positive regulation of B cell proliferation / regulation of Rho GTPase activity / negative regulation of mast cell cytokine production / negative regulation of Rho protein signal transduction / T-helper 2 type immune response / regulation of cell proliferation / positive regulation of apoptosis / negative regulation of apoptosis / negative regulation of apoptosis / regulation of memory T cell differentiation / negative regulation of cell differentiation / negative regulation of T-helper 2 cell differentiation / negative regulation of isotype switching to IgE isotypes / negative regulation of isotype switching to IgE isotypes / erythrocyte development / regulation of inflammatory response / positive regulation of cell motility | nucleic acid binding / DNA binding / DNA binding / chromatin binding / chromatin binding / chromatin binding / protein binding / protein binding / protein binding / zinc ion binding / transcription repressor activity / transcription repressor activity / transcription repressor activity / chromatin DNA binding / sequence-specific DNA binding / sequence-specific DNA binding / metal ion binding | intracellular / nucleus / nucleus / nucleus / replication fork |
| 1452257_at | -0.45 | -2.73 | -0.38 | -3.47 | -0.73 | 0.67 | -0.65 | -0.23 | Bdh1 | 3-hydroxybutyrate dehydrogenase, type 1 | metabolic process / metabolic process | catalytic activity / 3-hydroxybutyrate dehydrogenase activity / 3-hydroxybutyrate dehydrogenase activity / binding / oxidoreductase activity / oxidoreductase activity | mitochondrion / mitochondrion / mitochondrion / mitochondrial inner membrane / mitochondrial matrix |
| 1453011_at | -0.08 | -6.33 | -0.07 | -6.20 | 0.17 | -5.78 | 0.78 | 0.14 | Bdh2 | 3-hydroxybutyrate dehydrogenase, type 2 | fatty acid beta-oxidation / fatty acid beta-oxidation / metabolic process | catalytic activity / 3-hydroxybutyrate dehydrogenase activity / 3-hydroxybutyrate dehydrogenase activity / binding / oxidoreductase activity / NAD binding / NAD binding | cytoplasm / cytoplasm |
| 1416866_at | -0.53 | 1.12 | -0.49 | 0.59 | -0.45 | -0.12 | -0.63 | 2.60 | Bet1 | blocked early in transport 1 homolog (S. cerevisiae) | transport / ER to Golgi vesicle-mediated transport / ER to Golgi vesicle-mediated transport / protein transport / vesicle-mediated transport / vesicle-mediated transport | protein binding | Golgi membrane / Golgi membrane / endoplasmic reticulum / Golgi apparatus / membrane / integral to membrane / integral to membrane |
| 1418271_at | -0.02 | -6.43 | -0.42 | -1.89 | -0.72 | 2.37 | -0.69 | 1.93 | Bhlhb5 | basic helix-loop-helix domain containing, class B5 | transcription / regulation of transcription, DNA-dependent / regulation of transcription, DNA-dependent / neuron differentiation / regulation of transcription / retinal bipolar neuron differentiation / retina morphogenesis in camera-type eye | transcription repressor activity / transcription regulator activity | nucleus / nucleus |
| 1441137_at | -0.82 | 2.54 | -0.55 | -0.78 | -0.34 | -3.61 | -0.26 | -4.40 | Bicc1 | bicaudal C homolog 1 (Drosophila) | multicellular organismal development | nucleic acid binding / RNA binding / RNA binding | --- |
| 1417045_at | 0.67 | -1.00 | 0.47 | -3.01 | 0.77 | 0.15 | 0.31 | -4.53 | Bid | BH3 interacting domain death agonist | apoptosis / apoptosis / regulation of apoptosis / regulation of apoptosis / positive regulation of apoptosis | protein binding | cytoplasm / mitochondrion / membrane / mitochondrial membrane |
| 1447873_x_at | 0.51 | -3.11 | 0.39 | -4.13 | 0.82 | 0.19 | 0.73 | -0.64 | Bid | BH3 interacting domain death agonist | apoptosis / apoptosis / regulation of apoptosis / regulation of apoptosis / positive regulation of apoptosis | protein binding | cytoplasm / mitochondrion / membrane / mitochondrial membrane |
| 1451780_at | 1.27 | 0.51 | 1.05 | -0.88 | 1.09 | -0.63 | 0.77 | -2.73 | Blnk | B-cell linker | intracellular signaling cascade / B cell activation | protein binding / protein binding | cytoplasm / membrane |
| 1448733_at | -0.64 | 2.48 | -0.53 | 0.83 | -0.08 | -5.99 | -0.18 | -4.80 | Bmi1 | Bmi1 polycomb ring finger oncogene | negative regulation of transcription from RNA polymerase II promoter / skeletal development / transcription / regulation of transcription, DNA-dependent / humoral immune response / brain development / cellular process / chromatin modification / histone acetylation / histone ubiquitination / rostrocaudal neural tube patterning / positive regulation of B cell proliferation / positive regulation of immature T cell proliferation in the thymus / somatic stem cell division | chromatin binding / ubiquitin-protein ligase activity / protein binding / protein binding / zinc ion binding / transcription repressor activity / metal ion binding | ubiquitin ligase complex / nucleus / nucleus / cytoplasm / nuclear body / PcG protein complex |
| 1416923_a_at | -0.55 | -2.56 | -0.93 | 1.36 | -0.95 | 1.51 | -0.57 | -2.16 | Bnip3l | BCL2/adenovirus E1B interacting protein 3-like | apoptosis / apoptosis / induction of apoptosis / negative regulation of survival gene product expression / regulation of apoptosis / positive regulation of apoptosis / defense response to virus | protein binding | nucleus / nuclear envelope / mitochondrion / mitochondrion / mitochondrion / mitochondrial envelope / endoplasmic reticulum / membrane / integral to membrane / integral to membrane |
| 1448525_a_at | -0.74 | -2.35 | -0.93 | -0.71 | -1.03 | 0.01 | -0.53 | -3.79 | Bnip3l | BCL2/adenovirus E1B interacting protein 3-like | apoptosis / apoptosis / induction of apoptosis / negative regulation of survival gene product expression / regulation of apoptosis / positive regulation of apoptosis / defense response to virus | protein binding | nucleus / nuclear envelope / mitochondrion / mitochondrion / mitochondrion / mitochondrial envelope / endoplasmic reticulum / membrane / integral to membrane / integral to membrane |
| 1417040_a_at | 0.20 | -5.38 | 0.43 | -2.51 | 0.72 | 1.11 | 0.75 | 1.41 | Bok | Bcl-2-related ovarian killer protein | apoptosis / induction of apoptosis / regulation of apoptosis | --- | --- |
| 1434544_at | 0.25 | -4.46 | 0.60 | 0.73 | 0.36 | -2.76 | 0.44 | -1.53 | Bola2 | bolA-like 2 (E. coli) | --- | --- | --- |
| 1456738_s_at | 0.53 | -0.43 | 0.62 | 0.88 | 0.66 | 1.36 | 0.22 | -4.66 | Brp16 | brain protein 16 | --- | binding | --- |
| 1439775_at | -0.34 | -4.80 | -0.50 | -3.13 | -0.54 | -2.77 | -0.92 | 0.92 | Brwd3 | bromodomain and WD repeat domain containing 3 | --- | --- | --- |
| 1449453_at | -0.56 | -3.65 | -0.54 | -3.66 | -1.12 | 1.10 | -0.35 | -4.87 | Bst1 | bone marrow stromal cell antigen 1 | metabolic process | catalytic activity / NAD+ nucleosidase activity / binding / hydrolase activity | plasma membrane / membrane / anchored to membrane |
| 1441980_at | -0.74 | 0.04 | -0.13 | -5.94 | 0.21 | -5.46 | 0.00 | -6.10 | C030007I09Rik | RIKEN cDNA C030007I09 gene | --- | --- | --- |
| 1436196_at | 0.96 | 5.10 | 0.06 | -6.16 | -0.16 | -5.39 | 0.10 | -5.73 | C030046G05 | hypothetical protein C030046G05 | --- | --- | --- |
| 1428490_at | -0.71 | 2.00 | -0.60 | 0.56 | -0.54 | -0.22 | -0.30 | -3.63 | C1galt1 | core 1 synthase, glycoprotein-N-acetylgalactosamine 3-beta-galactosyltransferase, 1 | angiogenesis / angiogenesis / kidney development / multicellular organismal development / O-glycan processing, core 1 / cell differentiation | magnesium ion binding / galactosyltransferase activity / glycoprotein-N-acetylgalactosamine 3-beta-galactosyltransferase activity / transferase activity / transferase activity, transferring glycosyl groups / metal ion binding | extracellular space / membrane / integral to membrane |
| 1416051_at | -0.28 | -4.12 | -0.26 | -4.27 | -0.66 | 1.31 | -0.24 | -4.41 | C2 | complement component 2 (within H-2S) | proteolysis / immune response / complement activation / complement activation, classical pathway / innate immune response | classical-complement-pathway C3/C5 convertase activity / catalytic activity / serine-type endopeptidase activity / peptidase activity / hydrolase activity | extracellular region / extracellular space |
| 1457664_x_at | -0.21 | -5.10 | -0.22 | -4.84 | -0.61 | 0.44 | -0.21 | -4.84 | C2 | Complement component 2 (within H-2S) | proteolysis / immune response / complement activation / complement activation, classical pathway / innate immune response | classical-complement-pathway C3/C5 convertase activity / catalytic activity / serine-type endopeptidase activity / peptidase activity / hydrolase activity | extracellular region / extracellular space |
| 1443951_at | -0.10 | -6.17 | -0.01 | -6.28 | -0.61 | 0.31 | 0.01 | -6.09 | C230076A16Rik | RIKEN cDNA C230076A16 gene | protein amino acid lipidation / lipid metabolic process / triacylglycerol metabolic process / lipid transport / lipoprotein metabolic process / cholesterol homeostasis / lipoprotein transport | lipid transporter activity / lipid transporter activity | --- |
| 1426894_s_at | 0.20 | -5.86 | 0.23 | -5.52 | 1.04 | 1.64 | 0.40 | -4.14 | C230093N12Rik | RIKEN cDNA C230093N12 gene | --- | --- | --- |
| 1430752_at | -0.07 | -6.39 | -0.04 | -6.26 | -0.91 | 0.19 | -0.18 | -5.68 | C330006D17Rik | RIKEN cDNA C330006D17 gene | --- | --- | --- |
| 1434171_at | 0.14 | -6.11 | 0.22 | -5.47 | 0.94 | 1.53 | 0.30 | -4.74 | C330011K17Rik | RIKEN cDNA C330011K17 gene | regulation of transcription, DNA-dependent | nucleic acid binding / zinc ion binding / metal ion binding | intracellular / nucleus |
| 1442546_at | -1.33 | 2.05 | -0.98 | -0.46 | -1.16 | 0.81 | -0.68 | -2.76 | C730027H18Rik | RIKEN cDNA C730027H18 gene | --- | --- | --- |
| 1436030_at | 0.55 | -1.17 | 0.86 | 2.60 | 0.79 | 1.84 | 0.42 | -2.63 | Cachd1 | cache domain containing 1 | transport / ion transport / calcium ion transport | calcium ion binding | membrane / integral to membrane |
| 1436031_at | 0.57 | 0.16 | 0.86 | 3.87 | 0.54 | -0.23 | 0.58 | 0.41 | Cachd1 / LOC100047857 | cache domain containing 1 / similar to Cache domain containing 1 | transport / ion transport / calcium ion transport | calcium ion binding | membrane / integral to membrane |
| 1440397_at | 0.85 | 0.92 | 0.12 | -6.03 | -0.22 | -5.45 | -0.94 | 1.82 | Cacna2d1 | calcium channel, voltage-dependent, alpha2/delta subunit 1 | transport / ion transport / calcium ion transport / regulation of calcium ion transport | ion channel activity / voltage-gated ion channel activity / calcium channel activity / calcium ion binding / metal ion binding | extracellular space / membrane / membrane / integral to membrane / sarcoplasmic reticulum / T-tubule |
| 1417377_at | -0.49 | 1.15 | -0.59 | 2.71 | -0.17 | -4.80 | -0.09 | -5.60 | Cadm1 | cell adhesion molecule 1 | apoptosis / immune response / cell cycle / cell adhesion / cell adhesion / homophilic cell adhesion / heterophilic cell adhesion / multicellular organismal development / spermatogenesis / synaptogenesis / cell recognition / cell-cell adhesion / calcium-independent cell-cell adhesion / cell differentiation / susceptibility to natural killer cell mediated cytotoxicity / negative regulation of cell cycle / positive regulation of natural killer cell mediated cytotoxicity / detection of stimulus | receptor binding / protein binding / protein binding / protein C-terminus binding / PDZ domain binding / protein homodimerization activity | plasma membrane / plasma membrane / intercellular junction / synaptic vesicle / membrane / integral to membrane / integral to membrane / basolateral plasma membrane / axon / dendrite / synapse |
| 1418489_a_at | -0.62 | 0.04 | -0.24 | -4.84 | -0.34 | -3.65 | -0.53 | -0.99 | Calcrl | calcitonin receptor-like | signal transduction / G-protein coupled receptor protein signaling pathway / G-protein coupled receptor protein signaling pathway / G-protein signaling, adenylate cyclase activating pathway / heart development / positive regulation of cell proliferation / positive regulation of smooth muscle cell proliferation | calcitonin gene-related polypeptide receptor activity / signal transducer activity / receptor activity / G-protein coupled receptor activity / G-protein coupled receptor activity / calcitonin receptor activity | extracellular space / plasma membrane / membrane / membrane / integral to membrane / integral to membrane |
| 1425814_a_at | -0.65 | -1.55 | -1.03 | 2.18 | -0.84 | 0.37 | -0.80 | 0.04 | Calcrl | calcitonin receptor-like | signal transduction / G-protein coupled receptor protein signaling pathway / G-protein coupled receptor protein signaling pathway / G-protein signaling, adenylate cyclase activating pathway / heart development / positive regulation of cell proliferation / positive regulation of smooth muscle cell proliferation | calcitonin gene-related polypeptide receptor activity / signal transducer activity / receptor activity / G-protein coupled receptor activity / G-protein coupled receptor activity / calcitonin receptor activity | extracellular space / plasma membrane / membrane / membrane / integral to membrane / integral to membrane |
| 1424474_a_at | 0.50 | -3.15 | 0.16 | -5.87 | 0.98 | 1.81 | 0.30 | -4.77 | Camkk2 | calcium/calmodulin-dependent protein kinase kinase 2, beta | protein amino acid phosphorylation / protein amino acid phosphorylation / protein amino acid autophosphorylation | nucleotide binding / protein kinase activity / protein serine/threonine kinase activity / calmodulin-dependent protein kinase activity / calcium ion binding / calmodulin binding / ATP binding / kinase activity / transferase activity | cytoplasm |
| 1448752_at | 1.72 | 10.21 | 1.42 | 7.99 | 0.68 | 1.11 | 0.05 | -6.03 | Car2 | carbonic anhydrase 2 | morphogenesis of an epithelium / one-carbon compound metabolic process / carbon dioxide transport / secretion | carbonate dehydratase activity / zinc ion binding / lyase activity / metal ion binding | cytoplasm |
| 1427482_a_at | -0.18 | -5.35 | -0.16 | -5.40 | -0.80 | 3.35 | -0.66 | 1.56 | Car8 / LOC676792 | carbonic anhydrase 8 / similar to Carbonic anhydrase-related protein (CARP) (CA-VIII) | one-carbon compound metabolic process / phosphoinositide-mediated signaling | carbonate dehydratase activity / protein binding / zinc ion binding / metal ion binding | cytoplasm |
| 1452394_at | 0.58 | 0.55 | 0.60 | 0.87 | 0.62 | 1.17 | 0.40 | -1.90 | Cars | cysteinyl-tRNA synthetase | translation / translation / tRNA aminoacylation for protein translation / cysteinyl-tRNA aminoacylation / cysteinyl-tRNA aminoacylation | tRNA binding / nucleotide binding / aminoacyl-tRNA ligase activity / cysteine-tRNA ligase activity / cysteine-tRNA ligase activity / ATP binding / ATP binding / zinc ion binding / ligase activity / ligase activity / metal ion binding | soluble fraction / cytoplasm / cytoplasm |
| 1418981_at | -0.41 | -1.89 | -0.66 | 1.79 | -0.83 | 3.91 | -0.74 | 2.73 | Casp12 / LOC100044205 | caspase 12 / hypothetical protein LOC100044205 | proteolysis / proteolysis / apoptosis / induction of apoptosis / induction of apoptosis / virus-infected cell apoptosis / unfolded protein response / regulation of apoptosis | protein binding / protein binding / peptidase activity / cysteine-type peptidase activity / hydrolase activity / caspase activity / caspase activity | intracellular / endoplasmic reticulum |
| 1449297_at | -0.43 | -3.52 | -0.83 | 0.88 | -0.96 | 2.15 | -0.80 | 0.57 | Casp12 / LOC100044205 | caspase 12 / hypothetical protein LOC100044205 | proteolysis / proteolysis / apoptosis / induction of apoptosis / induction of apoptosis / virus-infected cell apoptosis / unfolded protein response / regulation of apoptosis | protein binding / protein binding / peptidase activity / cysteine-type peptidase activity / hydrolase activity / caspase activity / caspase activity | intracellular / endoplasmic reticulum |
| 1449591_at | 1.30 | 10.39 | 0.40 | -0.62 | -0.15 | -5.19 | -0.01 | -6.09 | Casp4 / LOC100044206 | caspase 4, apoptosis-related cysteine peptidase / hypothetical protein LOC100044206 | proteolysis / proteolysis / apoptosis / induction of apoptosis / induction of apoptosis / regulation of apoptosis | protein binding / peptidase activity / cysteine-type peptidase activity / hydrolase activity / caspase activity / caspase activity | intracellular |
| 1415995_at | -0.66 | 2.07 | -0.26 | -4.05 | -0.35 | -2.54 | -0.23 | -4.27 | Casp6 | caspase 6 | proteolysis / proteolysis / apoptosis / induction of apoptosis / induction of apoptosis / regulation of apoptosis | peptidase activity / cysteine-type peptidase activity / hydrolase activity / caspase activity / caspase activity | cytoplasm |
| 1451413_at | -0.58 | 1.11 | -0.66 | 2.37 | -0.25 | -4.11 | -0.16 | -5.12 | Cast | calpastatin | protein catabolic process | endopeptidase inhibitor activity / cysteine protease inhibitor activity / calpain inhibitor activity / calpain inhibitor activity | --- |
| 1416429_a_at | -0.66 | 1.15 | -0.26 | -4.30 | -0.10 | -5.98 | -0.04 | -6.05 | Cat | catalase | response to oxidative stress / hydrogen peroxide catabolic process / hydrogen peroxide catabolic process / oxidation reduction | catalase activity / catalase activity / peroxidase activity / iron ion binding / oxidoreductase activity / oxidoreductase activity, acting on peroxide as acceptor / metal ion binding | mitochondrion / peroxisome / peroxisomal membrane |
| 1416430_at | -0.64 | 2.68 | 0.00 | -6.28 | -0.07 | -6.07 | -0.02 | -6.07 | Cat | catalase | response to oxidative stress / hydrogen peroxide catabolic process / hydrogen peroxide catabolic process / oxidation reduction | catalase activity / catalase activity / peroxidase activity / iron ion binding / oxidoreductase activity / oxidoreductase activity, acting on peroxide as acceptor / metal ion binding | mitochondrion / peroxisome / peroxisomal membrane |
| 1424533_a_at | 0.61 | -0.41 | 0.55 | -1.20 | 0.72 | 0.92 | 0.26 | -4.55 | Ccdc137 | coiled-coil domain containing 137 | --- | --- | --- |
| 1452414_s_at | 0.49 | -1.86 | 0.37 | -3.31 | 0.77 | 1.66 | 0.38 | -3.10 | Ccdc86 | coiled-coil domain containing 86 | --- | --- | nucleus / nucleus |
| 1454197_a_at | 0.60 | 0.30 | 0.55 | -0.40 | 0.88 | 3.68 | 0.50 | -1.04 | Ccdc86 | coiled-coil domain containing 86 | --- | --- | nucleus / nucleus |
| 1420380_at | 2.38 | 7.30 | 1.05 | -0.30 | 0.94 | -1.16 | 0.67 | -3.02 | Ccl2 | chemokine (C-C motif) ligand 2 | positive regulation of endothelial cell proliferation / cellular calcium ion homeostasis / anti-apoptosis / chemotaxis / inflammatory response / immune response / signal transduction / transforming growth factor beta receptor signaling pathway / cytokine and chemokine mediated signaling pathway / vascular endothelial growth factor receptor signaling pathway | G-protein-coupled receptor binding / cytokine activity / protein binding / chemokine activity / CCR2 chemokine receptor binding | extracellular region / extracellular space / extracellular space / cytoplasm / cell soma |
| 1422029_at | 1.24 | 2.78 | -0.07 | -6.21 | -0.36 | -4.80 | -0.58 | -2.75 | Ccl20 | chemokine (C-C motif) ligand 20 | chemotaxis / inflammatory response / immune response / signal transduction | cytokine activity / cytokine activity / protein binding / chemokine activity | extracellular region / extracellular space / extracellular space |
| 1418126_at | 1.25 | 2.68 | 0.94 | 0.25 | 0.38 | -4.67 | -0.02 | -6.09 | Ccl5 | chemokine (C-C motif) ligand 5 | chemotaxis / inflammatory response / immune response / signal transduction | cytokine activity / chemokine activity / chemoattractant activity | extracellular region / extracellular space / extracellular space |
| 1450920_at | -0.66 | 2.04 | -0.36 | -2.42 | -0.06 | -6.18 | -0.15 | -5.23 | Ccnb2 | cyclin B2 | cell cycle / mitosis / cell division / regulation of cell cycle | protein binding / cyclin-dependent protein kinase regulator activity | nucleus / microtubule cytoskeleton |
| 1415907_at | -0.73 | 0.48 | -0.22 | -5.28 | -0.33 | -4.21 | -0.05 | -6.03 | Ccnd3 | cyclin D3 | cell cycle / signal transduction / T cell proliferation / cell division / regulation of cell cycle | cyclin-dependent protein kinase activity / protein binding / cyclin-dependent protein kinase regulator activity | nucleus / cytoplasm |
| 1448334_a_at | -0.20 | -5.47 | -0.68 | 0.52 | -0.24 | -4.97 | -0.36 | -3.38 | Ccni | cyclin I | regulation of cell cycle | cyclin-dependent protein kinase regulator activity | --- |
| 1435445_at | -0.44 | -3.24 | -0.63 | -0.84 | -0.91 | 2.14 | -0.58 | -1.34 | Ccnt2 | cyclin T2 | regulation of cyclin-dependent protein kinase activity / cytokinesis / regulation of transcription, DNA-dependent / transcription from RNA polymerase II promoter / cell cycle | protein binding / cyclin-dependent protein kinase regulator activity | nucleus / nucleus |
| 1433741_at | -0.75 | 2.77 | -0.42 | -1.80 | -0.44 | -1.51 | -0.64 | 1.31 | Cd38 | CD38 antigen | metabolic process / positive regulation of B cell proliferation | catalytic activity / NAD+ nucleosidase activity / binding / hydrolase activity / hydrolase activity, acting on glycosyl bonds / phosphorus-oxygen lyase activity | membrane fraction / membrane / integral to membrane / integral to membrane |
| 1439221_s_at | 0.77 | 3.91 | 0.37 | -2.05 | -0.16 | -5.30 | -0.11 | -5.61 | Cd40 | CD40 antigen | immune response-regulating cell surface receptor signaling pathway / apoptosis / immune response / signal transduction / positive regulation of B cell proliferation / positive regulation of B cell proliferation / positive regulation of interleukin-12 production / B cell activation / positive regulation of isotype switching to IgG isotypes / regulation of immune response / regulation of immunoglobulin secretion | receptor activity / transmembrane receptor activity / protein binding | extracellular region / external side of plasma membrane / membrane / integral to membrane / integral to membrane |
| 1449473_s_at | 0.82 | 4.29 | 0.25 | -4.05 | -0.03 | -6.27 | 0.08 | -5.86 | Cd40 | CD40 antigen | immune response-regulating cell surface receptor signaling pathway / apoptosis / immune response / signal transduction / positive regulation of B cell proliferation / positive regulation of B cell proliferation / positive regulation of interleukin-12 production / B cell activation / positive regulation of isotype switching to IgG isotypes / regulation of immune response / regulation of immunoglobulin secretion | receptor activity / transmembrane receptor activity / protein binding | extracellular region / external side of plasma membrane / membrane / integral to membrane / integral to membrane |
| 1419554_at | -0.30 | -4.25 | -0.34 | -3.63 | -0.68 | 0.95 | -0.32 | -3.70 | Cd47 | CD47 antigen (Rh-related antigen, integrin-associated signal transducer) | cell adhesion / integrin-mediated signaling pathway / opsonization / positive regulation of phagocytosis | protein binding / protein binding | extracellular space / plasma membrane / integral to plasma membrane / membrane / integral to membrane |
| 1443184_at | 0.20 | -5.53 | -0.19 | -5.41 | -0.67 | 0.15 | -0.26 | -4.60 | Cdc14a / LOC100047731 | CDC14 cell division cycle 14 homolog A (S. cerevisiae) / hypothetical protein LOC100047731 | protein amino acid dephosphorylation / protein amino acid dephosphorylation / cell cycle / dephosphorylation / cell division | phosphoprotein phosphatase activity / phosphoprotein phosphatase activity / protein tyrosine phosphatase activity / protein tyrosine phosphatase activity / protein tyrosine/serine/threonine phosphatase activity / protein tyrosine/serine/threonine phosphatase activity / hydrolase activity / phosphoric monoester hydrolase activity | nucleus / centrosome / spindle |
| 1422642_at | 0.66 | 2.73 | 0.52 | 0.69 | 0.33 | -2.61 | 0.19 | -4.55 | Cdc42ep3 | CDC42 effector protein (Rho GTPase binding) 3 | regulation of cell shape | protein binding | cytoplasm / cytoskeleton / endomembrane system / actin cytoskeleton / membrane |
| 1450700_at | 0.83 | 0.39 | 0.74 | -0.51 | 0.86 | 0.69 | 0.54 | -2.49 | Cdc42ep3 | CDC42 effector protein (Rho GTPase binding) 3 | regulation of cell shape | protein binding | cytoplasm / cytoskeleton / endomembrane system / actin cytoskeleton / membrane |
| 1449152_at | -0.70 | 1.68 | -0.44 | -1.92 | -0.26 | -4.43 | -0.18 | -5.14 | Cdkn2b / LOC100047564 | cyclin-dependent kinase inhibitor 2B (p15, inhibits CDK4) / similar to Cyclin-dependent kinase 4 inhibitor B (p14-INK4b) (p15-INK4b) | regulation of transcription, DNA-dependent / cell cycle / negative regulation of cell proliferation / negative regulation of cell cycle | DNA binding / transcription factor activity / cyclin-dependent protein kinase inhibitor activity / kinase activity | nucleus / cytoplasm |
| 1418071_s_at | 0.38 | -2.44 | 0.15 | -5.48 | 0.59 | 0.87 | 0.18 | -4.94 | Cdyl | chromodomain protein, Y chromosome-like | chromatin assembly or disassembly / transcription / regulation of transcription, DNA-dependent / metabolic process | chromatin binding / catalytic activity / histone acetyltransferase activity / acyltransferase activity / transferase activity | chromatin / nucleus |
| 1418901_at | 0.99 | 6.21 | 0.97 | 5.83 | 0.99 | 6.06 | 0.66 | 2.12 | Cebpb | CCAAT/enhancer binding protein (C/EBP), beta | embryonic placenta development / transcription / regulation of transcription, DNA-dependent / anti-apoptosis / induction of apoptosis / neuron differentiation / regulation of interleukin-6 biosynthetic process / fat cell differentiation / positive regulation of transcription / positive regulation of transcription from RNA polymerase II promoter | DNA binding / DNA binding / transcription factor activity / RNA polymerase II transcription factor activity, enhancer binding / protein binding / transcription activator activity / protein homodimerization activity / protein homodimerization activity / sequence-specific DNA binding / sequence-specific DNA binding / protein heterodimerization activity / protein heterodimerization activity / protein dimerization activity | nucleus / nucleus / cytoplasm |
| 1423233_at | 1.96 | 8.94 | 0.40 | -3.83 | 0.08 | -6.18 | 0.09 | -5.96 | Cebpd | CCAAT/enhancer binding protein (C/EBP), delta | transcription / regulation of transcription, DNA-dependent | DNA binding / DNA binding / transcription factor activity / protein binding / protein homodimerization activity / sequence-specific DNA binding / protein heterodimerization activity / protein dimerization activity | nucleus / nucleus |
| 1433561_at | -0.69 | 0.91 | -0.54 | -0.93 | -0.44 | -2.28 | -0.33 | -3.63 | Centb2 | centaurin, beta 2 | actin filament-based process / regulation of ARF GTPase activity | GTPase activator activity / ARF GTPase activator activity / ARF GTPase activator activity / zinc ion binding / metal ion binding | ruffle |
| 1425642_at | -1.00 | 2.98 | -0.87 | 1.71 | -0.43 | -3.16 | -0.17 | -5.53 | Cep290 | centrosomal protein 290 | transcription / regulation of transcription, DNA-dependent / transport / protein transport / protein transport / cell projection organization and biogenesis / hindbrain development / otic vesicle formation / eye photoreceptor cell development / pronephros development | protein binding / transcription activator activity / microtubule minus-end binding | gamma-tubulin complex / nucleus / nucleus / centrosome / centrosome / centrosome / cytosol / cilium / photoreceptor connecting cilium / cell projection |
| 1452983_at | 0.11 | -6.00 | 0.19 | -5.01 | 0.59 | 1.00 | 0.21 | -4.62 | Cep57 | centrosomal protein 57 | protein import into nucleus, translocation / spermatid development / fibroblast growth factor receptor signaling pathway | protein binding / fibroblast growth factor binding / protein homodimerization activity | nucleus / nucleus / cytoplasm / cytoplasm / centrosome / microtubule |
| 1452309_at | -0.25 | -4.83 | -0.46 | -2.06 | -0.93 | 3.63 | -0.56 | -0.58 | Cgnl1 | cingulin-like 1 | --- | motor activity | tight junction / actin cytoskeleton / myosin complex / cell junction / apical junction complex |
| 1451382_at | 3.25 | 10.04 | 2.30 | 6.42 | 1.98 | 5.00 | 1.33 | 1.28 | Chac1 | ChaC, cation transport regulator-like 1 (E. coli) | --- | --- | --- |
| 1438659_x_at | -0.11 | -6.12 | 0.23 | -4.91 | 0.66 | 0.47 | 0.65 | 0.44 | Chchd6 | coiled-coil-helix-coiled-coil-helix domain containing 6 | --- | --- | --- |
| 1417852_x_at | 1.50 | 4.88 | 0.12 | -6.08 | -0.41 | -4.30 | -0.14 | -5.83 | Clca1 | chloride channel calcium activated 1 | chloride transport | intracellular calcium activated chloride channel activity | integral to plasma membrane |
| 1417853_at | 0.93 | 1.27 | 0.30 | -4.94 | -0.30 | -4.97 | 0.03 | -6.08 | Clca1 | chloride channel calcium activated 1 | chloride transport | intracellular calcium activated chloride channel activity | integral to plasma membrane |
| 1460259_s_at | 1.45 | 2.56 | 0.03 | -6.27 | -0.25 | -5.74 | -0.15 | -5.89 | Clca1 / Clca2 | chloride channel calcium activated 1 / chloride channel calcium activated 2 | chloride transport / chloride transport / apoptosis | intracellular calcium activated chloride channel activity / intracellular calcium activated chloride channel activity | integral to plasma membrane / integral to plasma membrane |
| 1433486_at | -0.46 | -3.02 | -0.65 | -0.82 | -0.90 | 1.87 | -0.70 | -0.24 | Clcn3 | chloride channel 3 | transport / ion transport / chloride transport | ion channel activity / voltage-gated ion channel activity / voltage-gated chloride channel activity / chloride channel activity / protein binding / PDZ domain binding / chloride ion binding / protein heterodimerization activity | endosome / early endosome / late endosome / Golgi apparatus / vesicle membrane / vesicle membrane / membrane / integral to membrane / integral to membrane / apical plasma membrane / early endosome membrane / late endosome membrane |
| 1433487_at | -0.37 | -3.72 | -0.20 | -5.35 | -0.68 | 0.22 | -0.14 | -5.64 | Clcn3 | chloride channel 3 | transport / ion transport / chloride transport | ion channel activity / voltage-gated ion channel activity / voltage-gated chloride channel activity / chloride channel activity / protein binding / PDZ domain binding / chloride ion binding / protein heterodimerization activity | endosome / early endosome / late endosome / Golgi apparatus / vesicle membrane / vesicle membrane / membrane / integral to membrane / integral to membrane / apical plasma membrane / early endosome membrane / late endosome membrane |
| 1420804_s_at | 0.48 | -5.11 | 1.57 | 1.51 | 1.31 | 0.01 | 1.14 | -0.95 | Clec4d | C-type lectin domain family 4, member d | immune response | receptor activity / binding / sugar binding / sugar binding | membrane / integral to membrane / integral to membrane |
| 1417551_at | 0.39 | -1.62 | 0.58 | 1.48 | 0.73 | 3.68 | 0.76 | 3.79 | Cln3 | ceroid lipofuscinosis, neuronal 3, juvenile (Batten, Spielmeyer-Vogt disease) | autophagic vacuole fusion / regulation of action potential / globoside metabolic process / amino acid metabolic process / ceramide metabolic process / glucosylceramide metabolic process / galactosylceramide metabolic process / sphingomyelin metabolic process / amino acid transport / receptor-mediated endocytosis / lysosome organization and biogenesis / lysosomal lumen acidification / associative learning / regulation of gene expression / arginine transport / membrane organization and biogenesis / macroautophagy / negative regulation of macroautophagy / protein processing / ionotropic glutamate receptor signaling pathway / neurotransmitter metabolic process / amyloid precursor protein catabolic process / negative regulation of apoptosis / negative regulation of catalytic activity / negative regulation of neuron apoptosis / negative regulation of proteolysis / neuromuscular process controlling balance / regulation of intracellular pH / cytosolic calcium ion homeostasis | protein binding / protein binding / calcium-dependent protein binding | Golgi membrane / membrane fraction / nucleus / cytoplasm / lysosome / lysosome / lysosome / lysosomal membrane / early endosome / early endosome / late endosome / autophagic vacuole / endoplasmic reticulum / Golgi apparatus / Golgi stack / trans-Golgi network / plasma membrane / caveola / synaptic vesicle / membrane / integral to membrane / integral to membrane / integral to membrane / integral to endoplasmic reticulum membrane / neuron projection / cytoplasmic vesicle part / membrane raft |
| 1448456_at | 0.12 | -5.88 | 0.27 | -3.88 | 0.58 | 0.87 | 0.26 | -3.95 | Cln8 | ceroid-lipofuscinosis, neuronal 8 | age-dependent response to oxidative stress / phospholipid metabolic process / phospholipid metabolic process / phospholipid metabolic process / ceramide metabolic process / mitochondrial membrane organization and biogenesis / lysosome organization and biogenesis / nervous system development / nervous system development / visual perception / adult walking behavior / cholesterol metabolic process / cholesterol metabolic process / associative learning / adult locomotory behavior / regulation of cell size / spinal cord motor neuron differentiation / social behavior / negative regulation of apoptosis / cellular protein catabolic process / cellular macromolecule catabolic process / cellular protein metabolic process / photoreceptor cell maintenance / musculoskeletal movement / neuromuscular process controlling posture / neuromuscular process controlling balance / negative regulation of transferase activity / glutamate uptake during transmission of nerve impulse / retina development in camera-type eye / neurofilament cytoskeleton organization and biogenesis | --- | endoplasmic reticulum / endoplasmic reticulum / endoplasmic reticulum membrane / ER-Golgi intermediate compartment / membrane / integral to membrane / integral to membrane / ER-Golgi intermediate compartment membrane |
| 1459992_x_at | 0.44 | -0.87 | 0.21 | -4.46 | 0.60 | 1.87 | 0.34 | -2.28 | Cln8 | ceroid-lipofuscinosis, neuronal 8 | age-dependent response to oxidative stress / phospholipid metabolic process / phospholipid metabolic process / phospholipid metabolic process / ceramide metabolic process / mitochondrial membrane organization and biogenesis / lysosome organization and biogenesis / nervous system development / nervous system development / visual perception / adult walking behavior / cholesterol metabolic process / cholesterol metabolic process / associative learning / adult locomotory behavior / regulation of cell size / spinal cord motor neuron differentiation / social behavior / negative regulation of apoptosis / cellular protein catabolic process / cellular macromolecule catabolic process / cellular protein metabolic process / photoreceptor cell maintenance / musculoskeletal movement / neuromuscular process controlling posture / neuromuscular process controlling balance / negative regulation of transferase activity / glutamate uptake during transmission of nerve impulse / retina development in camera-type eye / neurofilament cytoskeleton organization and biogenesis | --- | endoplasmic reticulum / endoplasmic reticulum / endoplasmic reticulum membrane / ER-Golgi intermediate compartment / membrane / integral to membrane / integral to membrane / ER-Golgi intermediate compartment membrane |
| 1450981_at | -0.80 | 6.25 | -0.26 | -2.99 | 0.08 | -5.90 | 0.07 | -5.75 | Cnn2 | calponin 2 | actomyosin structure organization and biogenesis | actin binding / calmodulin binding | --- |
| 1420383_a_at | 0.17 | -5.13 | 0.28 | -3.27 | 0.58 | 1.81 | 0.41 | -1.01 | Col4a3bp | collagen, type IV, alpha 3 (Goodpasture antigen) binding protein | transport / lipid transport | kinase activity | cytoplasm / endoplasmic reticulum / Golgi apparatus |
| 1418440_at | -0.13 | -6.09 | -0.51 | -2.40 | -0.96 | 2.38 | -0.51 | -2.25 | Col8a1 | collagen, type VIII, alpha 1 | phosphate transport / cell adhesion / camera-type eye morphogenesis / epithelial cell proliferation | structural molecule activity / protein binding / extracellular matrix structural constituent conferring tensile strength | extracellular region / proteinaceous extracellular matrix / collagen / basement membrane / extracellular space / cytoplasm |
| 1447819_x_at | -0.02 | -6.44 | -0.16 | -5.54 | -0.69 | 1.37 | -0.28 | -4.04 | Col8a1 | collagen, type VIII, alpha 1 | phosphate transport / cell adhesion / camera-type eye morphogenesis / epithelial cell proliferation | structural molecule activity / protein binding / extracellular matrix structural constituent conferring tensile strength | extracellular region / proteinaceous extracellular matrix / collagen / basement membrane / extracellular space / cytoplasm |
| 1455627_at | -0.29 | -2.51 | -0.60 | 3.33 | -0.85 | 6.72 | -0.68 | 4.32 | Col8a1 | collagen, type VIII, alpha 1 | phosphate transport / cell adhesion / camera-type eye morphogenesis / epithelial cell proliferation | structural molecule activity / protein binding / extracellular matrix structural constituent conferring tensile strength | extracellular region / proteinaceous extracellular matrix / collagen / basement membrane / extracellular space / cytoplasm |
| 1452102_at | -0.29 | -2.91 | -0.19 | -4.53 | -0.30 | -2.64 | -0.65 | 3.06 | Copb2 | coatomer protein complex, subunit beta 2 (beta prime) | protein complex assembly / transport / intracellular protein transport / intra-Golgi vesicle-mediated transport / protein transport / vesicle-mediated transport | structural molecule activity / protein binding / protein transporter activity | Golgi membrane / cytoplasm / Golgi apparatus / Golgi apparatus / Golgi apparatus / membrane / membrane coat / COPI vesicle coat / COPI vesicle coat / cytoplasmic vesicle |
| 1437721_at | 0.13 | -5.92 | 0.73 | 2.02 | 0.09 | -6.07 | -0.16 | -5.29 | Coro1c | coronin, actin binding protein 1C | actin cortical patch assembly / phagocytosis / actin cytoskeleton organization and biogenesis | actin binding | cytoskeleton |
| 1455009_at | -0.37 | -2.04 | -0.59 | 1.62 | -0.52 | 0.53 | -0.58 | 1.46 | Cpd | carboxypeptidase D | proteolysis | carboxypeptidase activity / metallocarboxypeptidase activity / carboxypeptidase A activity / carboxypeptidase D activity / peptidase activity / metallopeptidase activity / zinc ion binding / metallocarboxypeptidase D activity / hydrolase activity / metal ion binding | extracellular space / nucleus / membrane / integral to membrane / integral to membrane |
| 1428234_at | -0.49 | -1.32 | -0.61 | 0.51 | 0.11 | -5.93 | -0.07 | -5.95 | Cpsf6 | cleavage and polyadenylation specific factor 6 | regulation of transcription, DNA-dependent / mRNA processing | nucleotide binding / nucleic acid binding / DNA binding / RNA binding / protein binding | nucleus / nucleus / mRNA cleavage factor complex / paraspeckles |
| 1452381_at | -0.60 | -3.43 | -0.81 | -1.59 | -1.16 | 1.16 | -0.87 | -1.05 | Creb3l2 | cAMP responsive element binding protein 3-like 2 | transcription / regulation of transcription, DNA-dependent / response to unfolded protein | DNA binding / transcription factor activity / sequence-specific DNA binding / protein dimerization activity | nucleus / endoplasmic reticulum / membrane / integral to membrane |
| 1415948_at | 0.11 | -6.06 | 0.63 | 0.95 | 0.88 | 3.87 | 0.75 | 2.35 | Creg1 | cellular repressor of E1A-stimulated genes 1 | regulation of cell growth / regulation of transcription, DNA-dependent | transcription factor binding | extracellular region / transcription factor complex |
| 1425154_a_at | 0.62 | 1.48 | 0.28 | -3.63 | 0.21 | -4.67 | 0.11 | -5.66 | Csf1 | colony stimulating factor 1 (macrophage) | positive regulation of cell-matrix adhesion / positive regulation of cell proliferation / cellular process / regulation of ossification / positive regulation of cell migration / positive regulation of multicellular organism growth / positive regulation of odontogenesis of dentine-containing teeth / positive regulation of macrophage differentiation / positive regulation of monocyte differentiation / positive regulation of osteoclast differentiation / positive regulation of osteoclast differentiation / positive regulation of osteoclast differentiation / positive regulation of protein kinase activity / positive regulation of Ras protein signal transduction / homeostasis of number of cells within a tissue | cytokine activity / cytokine activity / protein binding / growth factor activity | extracellular region / extracellular space / plasma membrane / membrane / integral to membrane |
| 1425155_x_at | 1.43 | 6.33 | 0.79 | 0.71 | 0.40 | -3.66 | 0.41 | -3.35 | Csf1 | colony stimulating factor 1 (macrophage) | positive regulation of cell-matrix adhesion / positive regulation of cell proliferation / cellular process / regulation of ossification / positive regulation of cell migration / positive regulation of multicellular organism growth / positive regulation of odontogenesis of dentine-containing teeth / positive regulation of macrophage differentiation / positive regulation of monocyte differentiation / positive regulation of osteoclast differentiation / positive regulation of osteoclast differentiation / positive regulation of osteoclast differentiation / positive regulation of protein kinase activity / positive regulation of Ras protein signal transduction / homeostasis of number of cells within a tissue | cytokine activity / cytokine activity / protein binding / growth factor activity | extracellular region / extracellular space / plasma membrane / membrane / integral to membrane |
| 1420340_at | 0.78 | 0.36 | 0.01 | -6.28 | 0.19 | -5.62 | 0.02 | -6.09 | Cspp1 | centrosome and spindle pole associated protein 1 | --- | --- | --- |
| 1452697_at | 0.60 | 0.13 | 0.55 | -0.56 | 0.55 | -0.57 | 0.20 | -4.95 | Ctdp1 | CTD (carboxy-terminal domain, RNA polymerase II, polypeptide A) phosphatase, subunit 1 | --- | phosphoprotein phosphatase activity / protein binding / hydrolase activity | intracellular / nucleus |
| 1420930_s_at | -0.56 | -0.46 | -0.62 | 0.50 | -0.60 | 0.23 | -0.52 | -0.84 | Ctnnal1 | catenin (cadherin associated protein), alpha-like 1 | cell adhesion / Rho protein signal transduction | structural molecule activity / cadherin binding | cytoplasm / cytosol / cytoskeleton / plasma membrane / actin cytoskeleton / membrane |
| 1435435_at | -1.81 | 5.64 | -1.21 | 1.74 | -0.81 | -1.43 | -0.99 | 0.08 | Cttnbp2 | cortactin binding protein 2 | actin cytoskeleton organization and biogenesis | cytoskeletal regulatory protein binding | actin cytoskeleton |
| 1415803_at | -0.02 | -6.44 | -0.94 | 1.00 | -0.58 | -2.53 | -0.34 | -4.56 | Cx3cl1 | chemokine (C-X3-C motif) ligand 1 | chemotaxis / immune response / cell adhesion / signal transduction / positive regulation of inflammatory response / positive regulation of calcium-independent cell-cell adhesion | cytokine activity / protein binding / chemokine activity | extracellular region / extracellular space / plasma membrane / cell surface / membrane / integral to membrane |
| 1419209_at | 3.00 | 11.17 | 1.79 | 5.93 | 1.01 | 0.68 | 0.33 | -4.89 | Cxcl1 | chemokine (C-X-C motif) ligand 1 | inflammatory response / immune response / cellular process / regulation of cell cycle | cytokine activity / chemokine activity / growth factor activity | extracellular region / extracellular space / extracellular space / extracellular space / intracellular |
| 1441855_x_at | 2.48 | 9.69 | 1.52 | 4.70 | 0.85 | -0.39 | 0.22 | -5.50 | Cxcl1 | chemokine (C-X-C motif) ligand 1 | inflammatory response / immune response / cellular process / regulation of cell cycle | cytokine activity / chemokine activity / growth factor activity | extracellular region / extracellular space / extracellular space / extracellular space / intracellular |
| 1457644_s_at | 2.84 | 11.91 | 1.62 | 6.29 | 1.00 | 1.77 | 0.31 | -4.72 | Cxcl1 | chemokine (C-X-C motif) ligand 1 | inflammatory response / immune response / cellular process / regulation of cell cycle | cytokine activity / chemokine activity / growth factor activity | extracellular region / extracellular space / extracellular space / extracellular space / intracellular |
| 1418930_at | 1.81 | 4.68 | 0.22 | -5.83 | 0.61 | -3.52 | 0.29 | -5.37 | Cxcl10 / LOC100045000 | chemokine (C-X-C motif) ligand 10 / similar to Small inducible cytokine B10 precursor (CXCL10) (Interferon-gamma-induced protein CRG-2) (Gamma-IP10) (IP-10) (C7) | chemotaxis / inflammatory response / immune response / signal transduction / positive regulation of cell proliferation / protein secretion / positive regulation of cell migration | cytokine activity / chemokine activity | extracellular region / extracellular space |
| 1419697_at | 0.41 | -4.45 | -0.84 | -0.35 | -0.55 | -3.07 | -0.96 | 0.72 | Cxcl11 / LOC630447 | chemokine (C-X-C motif) ligand 11 / similar to Small inducible cytokine B11 precursor (CXCL11) (Interferon-inducible T-cell alpha chemoattractant) (I-TAC) | chemotaxis / inflammatory response / immune response | cytokine activity / chemokine activity / chemokine activity | extracellular region / extracellular space / extracellular space |
| 1421404_at | -0.81 | -4.00 | -1.57 | 0.09 | -1.82 | 1.28 | -1.11 | -2.21 | Cxcl15 | chemokine (C-X-C motif) ligand 15 | chemotaxis / chemotaxis / inflammatory response / inflammatory response / immune response / signal transduction / hemopoiesis / neutrophil chemotaxis | cytokine activity / chemokine activity / chemokine activity | extracellular region / extracellular region / extracellular space / extracellular space |
| 1456428_at | -0.91 | -3.43 | -1.52 | -0.04 | -1.56 | 0.18 | -1.14 | -1.98 | Cxcl15 | chemokine (C-X-C motif) ligand 15 | chemotaxis / chemotaxis / inflammatory response / inflammatory response / immune response / signal transduction / hemopoiesis / neutrophil chemotaxis | cytokine activity / chemokine activity / chemokine activity | extracellular region / extracellular region / extracellular space / extracellular space |
| 1418718_at | 1.48 | 9.69 | 0.59 | 0.76 | 0.41 | -1.91 | 0.53 | -0.04 | Cxcl16 | chemokine (C-X-C motif) ligand 16 | chemotaxis / immune response | low-density lipoprotein receptor activity / scavenger receptor activity / cytokine activity / chemokine activity | extracellular region / extracellular space / membrane / integral to membrane |
| 1449195_s_at | 1.51 | 8.13 | 0.69 | 0.66 | 0.52 | -1.54 | 0.57 | -0.87 | Cxcl16 | chemokine (C-X-C motif) ligand 16 | chemotaxis / immune response | low-density lipoprotein receptor activity / scavenger receptor activity / cytokine activity / chemokine activity | extracellular region / extracellular space / membrane / integral to membrane |
| 1449984_at | 3.91 | 11.00 | 2.25 | 5.41 | 1.88 | 3.73 | 0.68 | -3.48 | Cxcl2 | chemokine (C-X-C motif) ligand 2 | chemotaxis / inflammatory response / immune response / signal transduction / neutrophil chemotaxis | cytokine activity / chemokine activity | extracellular region / extracellular space / extracellular space |
| 1438148_at | 1.09 | 6.83 | 0.19 | -4.96 | -0.02 | -6.30 | 0.03 | -6.06 | Cxcl3 | chemokine (C-X-C motif) ligand 3 | immune response | cytokine activity / chemokine activity | extracellular region |
| 1419728_at | 2.19 | 7.86 | 0.96 | 0.06 | -0.08 | -6.23 | -0.33 | -4.97 | Cxcl5 | chemokine (C-X-C motif) ligand 5 | chemotaxis / inflammatory response / immune response / signal transduction | cytokine activity / chemokine activity | extracellular region / extracellular space / extracellular space |
| 1424048_a_at | 0.63 | 1.53 | 1.00 | 5.89 | 0.71 | 2.67 | 0.58 | 0.90 | Cyb5r1 | cytochrome b5 reductase 1 | steroid biosynthetic process / lipid biosynthetic process / sterol biosynthetic process | cytochrome-b5 reductase activity / electron carrier activity / oxidoreductase activity / oxidoreductase activity | membrane / integral to membrane |
| 1434245_a_at | -0.62 | 0.53 | -0.28 | -4.10 | -0.03 | -6.29 | -0.02 | -6.08 | Cybasc3 | cytochrome b, ascorbate dependent 3 | transport / oxidation reduction | iron ion binding / oxidoreductase activity / metal ion binding | lysosome / lysosomal membrane / endosome / late endosome / membrane / integral to membrane |
| 1428347_at | -0.70 | 1.31 | -0.30 | -3.97 | -0.02 | -6.29 | -0.34 | -3.41 | Cyfip2 | cytoplasmic FMR1 interacting protein 2 | apoptosis / apoptosis / cell adhesion / cell-cell adhesion | protein binding / protein binding / protein binding | cytoplasm / cytoplasm / membrane / integral to membrane / synaptosome / synaptosome / cell junction / synapse / perinuclear region of cytoplasm |
| 1428283_at | -1.20 | 3.12 | -0.92 | 0.75 | -1.04 | 1.79 | -0.57 | -2.51 | Cyp2s1 | cytochrome P450, family 2, subfamily s, polypeptide 1 | oxidation reduction | monooxygenase activity / iron ion binding / electron carrier activity / oxidoreductase activity / oxidoreductase activity, acting on paired donors, with incorporation or reduction of molecular oxygen, reduced flavin or flavoprotein as one donor, and incorporation of one atom of oxygen / heme binding / metal ion binding / unspecific monooxygenase activity | endoplasmic reticulum / endoplasmic reticulum membrane / microsome / membrane / integral to membrane |
| 1422533_at | -0.42 | -4.52 | -1.00 | 0.69 | -1.18 | 2.13 | -0.37 | -4.56 | Cyp51 | cytochrome P450, family 51 | proteolysis / cholesterol biosynthetic process / oxidation reduction | trypsin activity / monooxygenase activity / iron ion binding / methyltransferase activity / sterol 14-demethylase activity / electron carrier activity / heme binding / metal ion binding | extracellular space / integral to membrane |
| 1450646_at | -0.18 | -5.49 | -0.63 | 0.63 | -1.04 | 5.22 | -0.64 | 0.77 | Cyp51 | cytochrome P450, family 51 | proteolysis / cholesterol biosynthetic process / oxidation reduction | trypsin activity / monooxygenase activity / iron ion binding / methyltransferase activity / sterol 14-demethylase activity / electron carrier activity / heme binding / metal ion binding | extracellular space / integral to membrane |
| 1418944_at | -1.34 | 2.92 | -0.73 | -1.88 | -0.27 | -5.51 | -0.41 | -4.42 | Cysltr1 | cysteinyl leukotriene receptor 1 | signal transduction / cell surface receptor linked signal transduction / G-protein coupled receptor protein signaling pathway | rhodopsin-like receptor activity / signal transducer activity / receptor activity / G-protein coupled receptor activity / leukotriene receptor activity / leukotriene receptor activity | plasma membrane / integral to plasma membrane / membrane / integral to membrane / integral to membrane |
| 1449282_at | -1.00 | 1.72 | -0.30 | -4.93 | -0.52 | -2.91 | -0.49 | -3.10 | Cysltr1 | cysteinyl leukotriene receptor 1 | signal transduction / cell surface receptor linked signal transduction / G-protein coupled receptor protein signaling pathway | rhodopsin-like receptor activity / signal transducer activity / receptor activity / G-protein coupled receptor activity / leukotriene receptor activity / leukotriene receptor activity | plasma membrane / integral to plasma membrane / membrane / integral to membrane / integral to membrane |
| 1455340_at | -0.82 | 3.06 | -0.69 | 1.40 | -0.28 | -4.16 | -0.59 | 0.13 | D030011O10Rik | RIKEN cDNA D030011O10 gene | --- | --- | membrane / integral to membrane |
| 1456422_at | -0.69 | 1.49 | -0.15 | -5.62 | -0.09 | -6.07 | -0.05 | -6.03 | D030011O10Rik | RIKEN cDNA D030011O10 gene | --- | --- | membrane / integral to membrane |
| 1436736_x_at | -1.47 | 4.29 | -0.71 | -1.79 | 0.16 | -5.96 | 0.16 | -5.78 | D0H4S114 | DNA segment, human D4S114 | regulation of transforming growth factor beta receptor signaling pathway | protein binding | --- |
| 1450839_at | -0.85 | 4.32 | -0.12 | -5.75 | 0.23 | -4.58 | -0.05 | -6.01 | D0H4S114 | DNA segment, human D4S114 | regulation of transforming growth factor beta receptor signaling pathway | protein binding | --- |
| 1455259_a_at | 0.16 | -5.94 | 0.20 | -5.52 | 0.74 | 0.10 | 0.58 | -1.60 | D530033C11Rik | RIKEN cDNA D530033C11 gene | --- | --- | --- |
| 1437085_at | -0.64 | -0.30 | -0.68 | 0.22 | -0.59 | -0.84 | -0.34 | -3.74 | D630039A03Rik | RIKEN cDNA D630039A03 gene | --- | --- | --- |
| 1441506_at | -0.25 | -6.19 | -0.91 | -3.53 | -1.97 | 1.61 | -0.58 | -4.86 | Dcn | decorin | --- | protein binding | extracellular region / proteinaceous extracellular matrix / extracellular space |
| 1426215_at | -1.13 | 2.47 | -0.61 | -2.20 | 0.09 | -6.19 | -0.23 | -5.33 | Ddc | dopa decarboxylase | amino acid and derivative metabolic process / carboxylic acid metabolic process / catecholamine biosynthetic process | catalytic activity / aromatic-L-amino-acid decarboxylase activity / lyase activity / carboxy-lyase activity / pyridoxal phosphate binding | --- |
| 1417516_at | 2.38 | 7.99 | 2.06 | 6.32 | 1.33 | 2.20 | 1.03 | 0.12 | Ddit3 | DNA-damage inducible transcript 3 | response to amphetamine / transcription / regulation of transcription, DNA-dependent / regulation of transcription, DNA-dependent / response to oxidative stress / ER overload response / ER overload response / cell cycle / cell cycle arrest / aging / response to nutrient / regulation of cell redox homeostasis / regulation of cell redox homeostasis / unfolded protein response / negative regulation of CREB transcription factor activity / response to drug / response to hydrogen peroxide / mRNA transcription from RNA polymerase II promoter / mRNA transcription from RNA polymerase II promoter / regulation of apoptosis / positive regulation of apoptosis / positive regulation of transcription / embryonic organ development | DNA binding / DNA binding / DNA binding / transcription factor activity / transcription factor activity / protein binding / sequence-specific DNA binding / protein dimerization activity | nucleus / nucleus / nucleus / cytoplasm |
| 1444139_at | 0.05 | -6.39 | 0.39 | -3.26 | 0.70 | 0.51 | 0.35 | -3.67 | Ddit4l | DNA-damage-inducible transcript 4-like | negative regulation of signal transduction | --- | cytoplasm / cytoplasm |
| 1451751_at | 0.25 | -5.30 | 0.29 | -4.81 | 0.77 | 0.31 | 0.40 | -3.61 | Ddit4l | DNA-damage-inducible transcript 4-like | negative regulation of signal transduction | --- | cytoplasm / cytoplasm |
| 1455688_at | 0.01 | -6.45 | -0.12 | -5.83 | -0.65 | 0.70 | -0.33 | -3.56 | Ddr2 | discoidin domain receptor family, member 2 | protein amino acid phosphorylation / cell adhesion / transmembrane receptor protein tyrosine kinase signaling pathway / transmembrane receptor protein tyrosine kinase signaling pathway / positive regulation of cell proliferation | nucleotide binding / protein kinase activity / protein tyrosine kinase activity / transmembrane receptor protein tyrosine kinase activity / receptor activity / ATP binding / kinase activity / transferase activity | membrane / integral to membrane / integral to membrane |
| 1428563_at | 0.02 | -6.44 | 0.16 | -6.01 | 1.05 | 0.15 | 0.28 | -5.35 | Ddx10 | DEAD (Asp-Glu-Ala-Asp) box polypeptide 10 | --- | nucleotide binding / nucleic acid binding / RNA binding / helicase activity / ATP binding / ATP-dependent helicase activity / hydrolase activity | --- |
| 1416751_a_at | 0.40 | -2.07 | 0.58 | 0.65 | 0.58 | 0.71 | 0.47 | -0.89 | Ddx20 | DEAD (Asp-Glu-Ala-Asp) box polypeptide 20 | negative regulation of transcription from RNA polymerase II promoter / mRNA processing / induction of apoptosis / RNA splicing / positive regulation of apoptosis | nucleotide binding / nucleic acid binding / DNA binding / helicase activity / protein binding / ATP binding / ATP-dependent helicase activity / transcription repressor activity / hydrolase activity | nucleus / spliceosome / cytoplasm / transcriptional repressor complex |
| 1436562_at | -0.52 | 0.63 | -0.63 | 2.42 | -0.77 | 4.32 | -0.74 | 3.64 | Ddx58 | DEAD (Asp-Glu-Ala-Asp) box polypeptide 58 | immune response / response to virus / regulation of apoptosis / innate immune response | nucleotide binding / nucleic acid binding / RNA binding / helicase activity / protein binding / ATP binding / ATP binding / ATP-dependent helicase activity / ATP-dependent helicase activity / hydrolase activity | intracellular / cytoplasm |
| 1456890_at | -0.49 | 0.44 | -0.76 | 4.31 | -0.62 | 2.44 | -0.63 | 2.51 | Ddx58 | DEAD (Asp-Glu-Ala-Asp) box polypeptide 58 | immune response / response to virus / regulation of apoptosis / innate immune response | nucleotide binding / nucleic acid binding / RNA binding / helicase activity / protein binding / ATP binding / ATP binding / ATP-dependent helicase activity / ATP-dependent helicase activity / hydrolase activity | intracellular / cytoplasm |
| 1449443_at | -0.59 | 0.05 | -0.27 | -4.33 | -0.35 | -3.17 | -0.26 | -4.29 | Decr1 | 2,4-dienoyl CoA reductase 1, mitochondrial | metabolic process / metabolic process | catalytic activity / binding / 2,4-dienoyl-CoA reductase (NADPH) activity / 2,4-dienoyl-CoA reductase (NADPH) activity / oxidoreductase activity / oxidoreductase activity | mitochondrion / mitochondrion / mitochondrion |
| 1436569_at | -0.91 | 2.15 | -0.66 | -0.47 | -0.77 | 0.77 | -0.73 | 0.34 | Depdc2 | DEP domain containing 2 | G-protein coupled receptor protein signaling pathway / intracellular signaling cascade / regulation of Rho protein signal transduction | guanyl-nucleotide exchange factor activity / Rho guanyl-nucleotide exchange factor activity / protein binding / Rac GTPase activator activity / Rac guanyl-nucleotide exchange factor activity | intracellular |
| 1428622_at | -0.99 | 1.36 | -0.81 | -0.19 | -0.47 | -3.51 | -0.43 | -3.77 | Depdc6 | DEP domain containing 6 | intracellular signaling cascade / intracellular signaling cascade | protein binding / protein binding | --- |
| 1443579_s_at | -1.30 | 4.82 | -0.79 | 0.31 | -0.34 | -4.49 | -0.76 | 0.00 | Depdc6 | DEP domain containing 6 | intracellular signaling cascade / intracellular signaling cascade | protein binding / protein binding | --- |
| 1451348_at | -1.32 | 2.41 | -1.17 | 1.32 | -0.57 | -3.45 | -0.74 | -1.94 | Depdc6 | DEP domain containing 6 | intracellular signaling cascade / intracellular signaling cascade | protein binding / protein binding | --- |
| 1424303_at | 0.38 | -3.73 | 0.54 | -1.70 | 0.97 | 2.99 | 0.50 | -2.12 | Depdc7 | DEP domain containing 7 | intracellular signaling cascade | --- | --- |
| 1417903_at | -0.75 | 0.82 | -0.36 | -3.78 | -0.25 | -4.98 | -0.07 | -5.98 | Dfna5h | deafness, autosomal dominant 5 homolog (human) | sensory perception of sound / inner ear receptor cell differentiation | --- | --- |
| 1422678_at | 0.45 | -1.37 | 0.78 | 3.26 | 0.64 | 1.54 | 0.30 | -3.42 | Dgat2 | diacylglycerol O-acyltransferase 2 | glycerol metabolic process / lipid metabolic process / lipid biosynthetic process / triacylglycerol biosynthetic process / triacylglycerol biosynthetic process / triacylglycerol biosynthetic process | 2-acylglycerol O-acyltransferase activity / diacylglycerol O-acyltransferase activity / diacylglycerol O-acyltransferase activity / diacylglycerol O-acyltransferase activity / acyltransferase activity / transferase activity | membrane fraction / membrane fraction / endoplasmic reticulum / endoplasmic reticulum membrane / membrane / integral to membrane / integral to membrane / integral to membrane |
| 1430750_at | -0.02 | -6.43 | 0.20 | -5.20 | 0.63 | 0.56 | 0.10 | -5.83 | Dhfr | dihydrofolate reductase | glycine biosynthetic process / one-carbon compound metabolic process / nucleotide biosynthetic process | dihydrofolate reductase activity / dihydrofolate reductase activity / oxidoreductase activity | --- |
| 1442003_at | -0.77 | 1.18 | -0.44 | -2.78 | -0.11 | -6.04 | -0.33 | -3.98 | Diap2 | diaphanous homolog 2 (Drosophila) | multicellular organismal development / cellular component organization and biogenesis / actin cytoskeleton organization and biogenesis / actin filament polymerization / cell differentiation / oogenesis | actin binding / actin binding / Rho GTPase binding | --- |
| 1416918_at | -0.60 | 0.03 | -0.20 | -5.12 | -0.31 | -3.85 | -0.22 | -4.74 | Dlg3 | discs, large homolog 3 (Drosophila) | --- | protein binding / protein binding | cytoplasm |
| 1449470_at | -0.19 | -5.31 | -0.69 | 1.76 | -0.30 | -3.73 | -0.37 | -2.67 | Dlx1 | distal-less homeobox 1 | regulation of transcription, DNA-dependent / multicellular organismal development / cell differentiation / negative regulation of neuron apoptosis / regulation of transcription | DNA binding / DNA binding / chromatin binding / transcription factor activity / sequence-specific DNA binding | nucleus / nucleus / transcription factor complex |
| 1448665_at | -0.78 | 1.52 | -0.98 | 3.59 | -0.47 | -2.32 | -0.72 | 0.79 | Dmd | dystrophin, muscular dystrophy | muscle development / skeletal muscle development / skeletal muscle development / neurotransmitter receptor metabolic process / muscle maintenance | actin binding / structural molecule activity / calcium ion binding / protein binding / protein binding / zinc ion binding / metal ion binding | insoluble fraction / cytoplasm / microsome / cytoskeleton / plasma membrane / dystrophin-associated glycoprotein complex / membrane / Z disc / cell-substrate junction / sarcolemma / membrane raft / synapse |
| 1442894_at | -0.30 | -4.15 | -0.52 | -1.01 | -0.78 | 2.23 | -0.29 | -4.04 | Dnahc6 | dynein, axonemal, heavy chain 6 | microtubule-based movement | microtubule motor activity | axonemal dynein complex / dynein complex |
| 1417191_at | 0.68 | 0.38 | 0.38 | -3.37 | 0.22 | -5.14 | 0.06 | -6.00 | Dnajb9 | DnaJ (Hsp40) homolog, subfamily B, member 9 | protein folding | chaperone regulator activity / heat shock protein binding / unfolded protein binding | extracellular space / nucleus / nucleolus / cytoplasm / endoplasmic reticulum |
| 1427881_at | 0.22 | -5.10 | 0.49 | -1.33 | 0.62 | 0.41 | 0.26 | -4.35 | Dnttip2 | deoxynucleotidyltransferase, terminal, interacting protein 2 | transcription / regulation of transcription, DNA-dependent | --- | nucleus |
| 1441462_at | -0.04 | -6.43 | -0.58 | -3.20 | -1.01 | 0.40 | -0.38 | -4.65 | Dock4 | dedicator of cytokinesis 4 | --- | guanyl-nucleotide exchange factor activity | endomembrane system / membrane / stereocilium / stereocilium bundle |
| 1450931_at | -0.22 | -5.29 | -0.36 | -3.54 | -0.53 | -1.33 | -0.71 | 0.88 | Dock9 | dedicator of cytokinesis 9 | --- | guanyl-nucleotide exchange factor activity / Rho guanyl-nucleotide exchange factor activity / GTP binding / Rho GTPase binding / GTPase binding | endomembrane system / membrane |
| 1450932_s_at | -0.11 | -6.06 | -0.15 | -5.58 | -0.46 | -1.51 | -0.78 | 2.67 | Dock9 | dedicator of cytokinesis 9 | --- | guanyl-nucleotide exchange factor activity / Rho guanyl-nucleotide exchange factor activity / GTP binding / Rho GTPase binding / GTPase binding | endomembrane system / membrane |
| 1452647_a_at | 0.63 | -1.09 | 0.54 | -2.04 | 1.02 | 3.00 | 0.64 | -0.82 | Dph2 | DPH2 homolog (S. cerevisiae) | --- | protein binding | --- |
| 1435680_a_at | 0.35 | -5.02 | 0.55 | -3.25 | 0.86 | -0.52 | 0.93 | 0.12 | Dpp7 | dipeptidylpeptidase 7 | proteolysis | aminopeptidase activity / serine-type endopeptidase activity / protein binding / peptidase activity / serine-type peptidase activity / hydrolase activity | extracellular space / lysosome |
| 1436479_a_at | 0.86 | 1.75 | 0.98 | 2.88 | 0.83 | 1.38 | 1.19 | 4.52 | Dpp7 | dipeptidylpeptidase 7 | proteolysis | aminopeptidase activity / serine-type endopeptidase activity / protein binding / peptidase activity / serine-type peptidase activity / hydrolase activity | extracellular space / lysosome |
| 1436480_at | 0.70 | 1.94 | 0.44 | -1.62 | 0.47 | -1.25 | 0.30 | -3.62 | Dpp7 | dipeptidylpeptidase 7 | proteolysis | aminopeptidase activity / serine-type endopeptidase activity / protein binding / peptidase activity / serine-type peptidase activity / hydrolase activity | extracellular space / lysosome |
| 1416019_at | 0.13 | -5.90 | 0.46 | -1.50 | 0.83 | 3.37 | 0.43 | -1.86 | Dr1 | down-regulator of transcription 1 | transcription / regulation of transcription, DNA-dependent | DNA binding / DNA binding / sequence-specific DNA binding | intracellular / nucleus / nucleus |
| 1422278_at | -0.81 | 0.42 | -0.63 | -1.46 | -0.40 | -3.88 | -0.02 | -6.09 | Drd3 | dopamine receptor 3 | response to amphetamine / regulation of blood volume by renin-angiotensin / regulation of blood volume by renin-angiotensin / signal transduction / G-protein coupled receptor protein signaling pathway / dopamine receptor, adenylate cyclase activating pathway / locomotory behavior / locomotory behavior / visual learning / regulation of dopamine secretion / regulation of lipid metabolic process / circadian regulation of gene expression / regulation of multicellular organism growth / response to cocaine / response to morphine / response to ethanol / negative regulation of blood pressure / behavioral response to cocaine / behavioral response to cocaine / negative regulation of oligodendrocyte differentiation / musculoskeletal movement, spinal reflex action / negative regulation of protein kinase B signaling cascade / negative regulation of dopamine receptor signaling pathway / positive regulation of dopamine receptor signaling pathway | rhodopsin-like receptor activity / dopamine D3 receptor activity / signal transducer activity / receptor activity / G-protein coupled receptor activity / dopamine receptor activity / protein binding | membrane fraction / plasma membrane / plasma membrane / membrane / integral to membrane / integral to membrane |
| 1436545_at | 0.65 | 1.61 | 0.78 | 3.24 | 0.42 | -1.73 | 0.40 | -1.90 | Dtx4 | deltex 4 homolog (Drosophila) | Notch signaling pathway | protein binding / zinc ion binding / metal ion binding | cytoplasm |
| 1453252_at | 0.20 | -6.03 | 0.05 | -6.26 | 1.08 | 0.48 | 0.34 | -5.03 | Dus4l | dihydrouridine synthase 4-like (S. cerevisiae) | tryptophan metabolic process / tRNA processing / metabolic process | catalytic activity / indole-3-glycerol-phosphate synthase activity / oxidoreductase activity / tRNA dihydrouridine synthase activity / FAD binding | --- |
| 1448830_at | 0.72 | 2.46 | 0.44 | -1.44 | 0.23 | -4.65 | 0.55 | 0.16 | Dusp1 | dual specificity phosphatase 1 | protein amino acid dephosphorylation / cell cycle / intracellular signaling cascade / dephosphorylation | phosphoprotein phosphatase activity / protein tyrosine phosphatase activity / protein binding / protein tyrosine/serine/threonine phosphatase activity / hydrolase activity / phosphoric monoester hydrolase activity / MAP kinase tyrosine/serine/threonine phosphatase activity | --- |
| 1428834_at | 0.78 | -0.10 | 0.89 | 1.06 | 0.45 | -3.48 | 0.35 | -4.24 | Dusp4 | dual specificity phosphatase 4 | protein amino acid dephosphorylation / protein amino acid dephosphorylation / dephosphorylation | phosphoprotein phosphatase activity / phosphoprotein phosphatase activity / protein tyrosine phosphatase activity / protein tyrosine/serine/threonine phosphatase activity / protein tyrosine/serine/threonine phosphatase activity / hydrolase activity / phosphoric monoester hydrolase activity / MAP kinase tyrosine/serine/threonine phosphatase activity | nucleus |
| 1423736_a_at | -0.68 | 0.45 | -0.28 | -4.56 | -0.01 | -6.31 | -0.02 | -6.09 | Dym | dymeclin | --- | --- | membrane / integral to membrane |
| 1459854_s_at | -0.41 | -0.90 | -0.60 | 2.16 | -0.18 | -4.81 | -0.18 | -4.66 | Dynlt3 | dynein light chain Tctex-type 3 | --- | motor activity | microtubule / dynein complex |
| 1444086_at | -0.40 | -4.00 | -1.07 | 2.84 | -0.67 | -1.02 | -0.39 | -3.84 | E030049G20Rik | RIKEN cDNA E030049G20 gene | --- | --- | --- |
| 1443541_at | -0.68 | 1.75 | -0.23 | -4.69 | -0.41 | -2.17 | -0.41 | -2.00 | EG381438 | Hypothetical protein LOC100041338 | --- | --- | --- |
| 1457365_at | 0.07 | -6.34 | 0.21 | -5.40 | 0.42 | -3.26 | 0.75 | 0.54 | EG628262 | Predicted gene, EG628262 | --- | --- | --- |
| 1427682_a_at | -0.50 | -5.42 | -1.73 | 0.80 | -1.97 | 1.93 | -1.59 | 0.16 | Egr2 | early growth response 2 | transcription / regulation of transcription, DNA-dependent / regulation of transcription, DNA-dependent / rhythmic behavior / rhythmic behavior / Schwann cell differentiation / myelination / regulation of transcription | nucleic acid binding / DNA binding / transcription factor activity / zinc ion binding / transcription regulator activity / metal ion binding | intracellular / nucleus / nucleus / nucleus |
| 1427683_at | -0.55 | -4.67 | -1.44 | 1.11 | -1.82 | 3.22 | -1.73 | 2.61 | Egr2 | early growth response 2 | transcription / regulation of transcription, DNA-dependent / regulation of transcription, DNA-dependent / rhythmic behavior / rhythmic behavior / Schwann cell differentiation / myelination / regulation of transcription | nucleic acid binding / DNA binding / transcription factor activity / zinc ion binding / transcription regulator activity / metal ion binding | intracellular / nucleus / nucleus / nucleus |
| 1416614_at | -0.37 | -3.01 | -0.42 | -2.10 | -0.13 | -5.76 | -0.63 | 0.81 | Eid1 | EP300 interacting inhibitor of differentiation 1 | negative regulation of transcription from RNA polymerase II promoter / transcription / regulation of transcription, DNA-dependent / cell cycle / cell differentiation / cell differentiation | transcription corepressor activity / protein binding / protein binding / specific transcriptional repressor activity / specific transcriptional repressor activity / histone acetyltransferase regulator activity / histone acetyltransferase binding | nucleus / nucleus / cytoplasm |
| 1434524_at | 0.02 | -6.43 | 0.20 | -5.34 | 0.69 | 0.72 | 0.25 | -4.65 | Eif2b3 | eukaryotic translation initiation factor 2B, subunit 3 | translational initiation / biosynthetic process / oligodendrocyte development / cellular response to stimulus | translation initiation factor activity / translation initiation factor activity / guanyl-nucleotide exchange factor activity / guanyl-nucleotide exchange factor activity / protein binding / protein binding / translation factor activity, nucleic acid binding / acyltransferase activity / transferase activity / nucleotidyltransferase activity | cytoplasm / eukaryotic translation initiation factor 2B complex / eukaryotic translation initiation factor 2B complex |
| 1417403_at | -0.39 | -2.54 | -0.47 | -1.24 | -0.65 | 1.40 | -0.35 | -2.83 | Elovl6 | ELOVL family member 6, elongation of long chain fatty acids (yeast) | fatty acid biosynthetic process / lipid biosynthetic process / fatty acid elongation | transferase activity, transferring groups other than amino-acyl groups | mitochondrion / endoplasmic reticulum / endoplasmic reticulum membrane / membrane / integral to membrane / integral to membrane / integral to endoplasmic reticulum membrane |
| 1417404_at | -0.46 | -1.82 | -0.45 | -1.78 | -0.64 | 0.85 | -0.46 | -1.59 | Elovl6 | ELOVL family member 6, elongation of long chain fatty acids (yeast) | fatty acid biosynthetic process / lipid biosynthetic process / fatty acid elongation | transferase activity, transferring groups other than amino-acyl groups | mitochondrion / endoplasmic reticulum / endoplasmic reticulum membrane / membrane / integral to membrane / integral to membrane / integral to endoplasmic reticulum membrane |
| 1448649_at | -0.80 | -3.17 | -1.14 | -0.82 | -1.41 | 0.80 | -0.98 | -1.76 | Enpep | glutamyl aminopeptidase | angiogenesis / proteolysis / cell proliferation / cell proliferation / cell migration / cell migration | aminopeptidase activity / membrane alanyl aminopeptidase activity / glutamyl aminopeptidase activity / glutamyl aminopeptidase activity / calcium ion binding / peptidase activity / metallopeptidase activity / zinc ion binding / hydrolase activity / metal ion binding | plasma membrane / brush border / external side of plasma membrane / membrane / integral to membrane / apical plasma membrane / cytoplasmic vesicle / apical part of cell |
| 1448136_at | 0.68 | -1.51 | 0.49 | -3.30 | -0.93 | 1.01 | -0.90 | 0.75 | Enpp2 | ectonucleotide pyrophosphatase/phosphodiesterase 2 | chemotaxis / metabolic process / lipid catabolic process | nucleic acid binding / catalytic activity / endonuclease activity / phosphodiesterase I activity / nucleotide diphosphatase activity / hydrolase activity / phosphoric monoester hydrolase activity / metal ion binding / alkylglycerophosphoethanolamine phosphodiesterase activity | extracellular region / extracellular space / integral to plasma membrane |
| 1444178_at | -0.36 | -2.46 | -0.04 | -6.21 | -0.66 | 2.26 | -0.14 | -5.36 | ENSMUSG00000052976 | predicted gene, ENSMUSG00000052976 | --- | --- | --- |
| 1435740_at | -0.42 | -2.34 | -0.42 | -2.20 | -0.56 | -0.27 | -0.85 | 3.20 | ENSMUSG00000072684 | predicted gene, ENSMUSG00000072684 | --- | --- | --- |
| 1435436_at | -0.95 | -2.65 | -1.31 | -0.46 | -1.58 | 1.05 | -1.17 | -1.20 | Epas1 | endothelial PAS domain protein 1 | two-component signal transduction system (phosphorelay) / angiogenesis / angiogenesis / response to hypoxia / response to hypoxia / embryonic placenta development / blood vessel remodeling / regulation of heart rate / transcription / regulation of transcription, DNA-dependent / response to oxidative stress / response to oxidative stress / mitochondrion organization and biogenesis / signal transduction / multicellular organismal development / visual perception / hemopoiesis / cell differentiation / cell differentiation / erythrocyte differentiation / lung development / norepinephrine metabolic process / surfactant homeostasis / regulation of transcription from RNA polymerase II promoter in response to oxidative stress / regulation of transcription from RNA polymerase II promoter in response to oxidative stress / regulation of transcription from RNA polymerase II promoter in response to oxidative stress / regulation of transcription / positive regulation of transcription from RNA polymerase II promoter / positive regulation of transcription from RNA polymerase II promoter / positive regulation of transcription from RNA polymerase II promoter / positive regulation of transcription from RNA polymerase II promoter / cell maturation | two-component sensor activity / DNA binding / DNA binding / DNA binding / DNA binding / transcription factor activity / transcription factor activity / signal transducer activity / protein binding / transcription factor binding / transcription regulator activity / histone acetyltransferase binding / protein heterodimerization activity | nucleus / nucleus / transcription factor complex / transcription factor complex / cytoplasm |
| 1433490_s_at | -0.40 | -3.16 | -0.71 | 0.84 | -0.73 | 1.01 | -0.56 | -0.92 | Epb4.1l2 | erythrocyte protein band 4.1-like 2 | regulation of cell shape / actin cytoskeleton organization and biogenesis / cortical actin cytoskeleton organization and biogenesis | actin binding / actin binding / structural molecule activity / binding / cytoskeletal protein binding / spectrin binding | cytoplasm / cytoskeleton / actin cytoskeleton / extrinsic to membrane |
| 1433491_at | -0.81 | -0.46 | -0.91 | 0.58 | -0.65 | -1.94 | -0.84 | -0.03 | Epb4.1l2 | erythrocyte protein band 4.1-like 2 | regulation of cell shape / actin cytoskeleton organization and biogenesis / cortical actin cytoskeleton organization and biogenesis | actin binding / actin binding / structural molecule activity / binding / cytoskeletal protein binding / spectrin binding | cytoplasm / cytoskeleton / actin cytoskeleton / extrinsic to membrane |
| 1451991_at | -0.47 | -3.25 | -0.51 | -2.59 | -0.84 | 0.83 | -0.45 | -3.16 | Epha7 | Eph receptor A7 | protein amino acid phosphorylation / transmembrane receptor protein tyrosine kinase signaling pathway / phosphorylation / retinal ganglion cell axon guidance / positive regulation of neuron apoptosis / branching morphogenesis of a nerve | nucleotide binding / protein kinase activity / protein tyrosine kinase activity / protein tyrosine kinase activity / transmembrane receptor protein tyrosine kinase activity / receptor activity / ephrin receptor activity / protein binding / ATP binding / kinase activity / transferase activity / chemorepellent activity | extracellular space / membrane / integral to membrane / integral to membrane |
| 1421240_at | 0.64 | 1.35 | 0.01 | -6.28 | -0.05 | -6.23 | -0.07 | -5.91 | Ern1 | endoplasmic reticulum (ER) to nucleus signalling 1 | transcription / regulation of transcription, DNA-dependent / mRNA processing / protein amino acid phosphorylation / protein amino acid phosphorylation / apoptosis / induction of apoptosis / response to stress / response to unfolded protein / activation of signaling protein activity involved in unfolded protein response / cell cycle arrest / unfolded protein response / protein amino acid autophosphorylation / oxidation reduction | nucleotide binding / magnesium ion binding / magnesium ion binding / catalytic activity / endonuclease activity / endoribonuclease activity / protein kinase activity / protein serine/threonine kinase activity / protein serine/threonine kinase activity / ATP binding / ATP binding / kinase activity / transferase activity / hydrolase activity / endoribonuclease activity, producing 5'-phosphomonoesters / metal ion binding | endoplasmic reticulum / endoplasmic reticulum membrane / endoplasmic reticulum membrane / membrane / integral to membrane / integral to endoplasmic reticulum membrane |
| 1419029_at | -0.11 | -6.32 | -0.27 | -5.50 | -1.18 | 1.52 | -0.96 | -0.13 | Ero1l | ERO1-like (S. cerevisiae) | protein folding / protein folding / protein thiol-disulfide exchange / transport / unfolded protein response / oxidation reduction | protein binding / electron carrier activity / oxidoreductase activity / oxidoreductase activity / oxidoreductase activity, acting on sulfur group of donors, disulfide as acceptor / FAD binding | endoplasmic reticulum / endoplasmic reticulum membrane / membrane / integral to endoplasmic reticulum membrane |
| 1419030_at | -0.17 | -6.20 | -0.35 | -5.28 | -1.39 | 1.89 | -1.05 | -0.36 | Ero1l | ERO1-like (S. cerevisiae) | protein folding / protein folding / protein thiol-disulfide exchange / transport / unfolded protein response / oxidation reduction | protein binding / electron carrier activity / oxidoreductase activity / oxidoreductase activity / oxidoreductase activity, acting on sulfur group of donors, disulfide as acceptor / FAD binding | endoplasmic reticulum / endoplasmic reticulum membrane / membrane / integral to endoplasmic reticulum membrane |
| 1449324_at | -0.42 | -5.28 | -0.56 | -4.34 | -1.53 | 1.97 | -0.94 | -1.71 | Ero1l | ERO1-like (S. cerevisiae) | protein folding / protein folding / protein thiol-disulfide exchange / transport / unfolded protein response / oxidation reduction | protein binding / electron carrier activity / oxidoreductase activity / oxidoreductase activity / oxidoreductase activity, acting on sulfur group of donors, disulfide as acceptor / FAD binding | endoplasmic reticulum / endoplasmic reticulum membrane / membrane / integral to endoplasmic reticulum membrane |
| 1425705_a_at | 0.70 | 2.75 | 0.69 | 2.58 | 0.27 | -3.81 | -0.04 | -6.02 | Ero1lb | ERO1-like beta (S. cerevisiae) | protein thiol-disulfide exchange / transport / oxidation reduction | protein binding / electron carrier activity / oxidoreductase activity / oxidoreductase activity, acting on sulfur group of donors, disulfide as acceptor / FAD binding | endoplasmic reticulum / endoplasmic reticulum membrane / membrane |
| 1434714_at | 1.07 | 5.91 | 1.05 | 5.60 | 0.55 | -0.26 | 0.06 | -5.98 | Ero1lb | ERO1-like beta (S. cerevisiae) | protein thiol-disulfide exchange / transport / oxidation reduction | protein binding / electron carrier activity / oxidoreductase activity / oxidoreductase activity, acting on sulfur group of donors, disulfide as acceptor / FAD binding | endoplasmic reticulum / endoplasmic reticulum membrane / membrane |
| 1417825_at | 0.27 | -3.73 | 0.55 | 1.18 | 0.83 | 4.92 | 0.63 | 2.24 | Esd | esterase D/formylglutathione hydrolase | --- | catalytic activity / carboxylesterase activity / carboxylesterase activity / hydrolase activity / hydrolase activity / hydrolase activity, acting on ester bonds / S-formylglutathione hydrolase activity | cytoplasm / cytoplasmic membrane-bounded vesicle / cytoplasmic vesicle |
| 1438488_at | -0.01 | -6.44 | 0.62 | 1.94 | 0.83 | 4.69 | 0.46 | -0.43 | Esd | esterase D/formylglutathione hydrolase | --- | catalytic activity / carboxylesterase activity / carboxylesterase activity / hydrolase activity / hydrolase activity / hydrolase activity, acting on ester bonds / S-formylglutathione hydrolase activity | cytoplasm / cytoplasmic membrane-bounded vesicle / cytoplasmic vesicle |
| 1441604_at | 1.13 | 0.53 | 0.68 | -2.85 | 0.42 | -4.77 | 0.33 | -5.15 | Esd | Esterase D/formylglutathione hydrolase | --- | catalytic activity / carboxylesterase activity / carboxylesterase activity / hydrolase activity / hydrolase activity / hydrolase activity, acting on ester bonds / S-formylglutathione hydrolase activity | cytoplasm / cytoplasmic membrane-bounded vesicle / cytoplasmic vesicle |
| 1435663_at | -0.68 | 1.18 | -0.14 | -5.73 | 0.05 | -6.24 | -0.07 | -5.94 | Esr1 | estrogen receptor 1 (alpha) | transcription / regulation of transcription, DNA-dependent / cell growth / negative regulation of mitosis | DNA binding / transcription factor activity / steroid hormone receptor activity / receptor activity / ligand-dependent nuclear receptor activity / ligand-dependent nuclear receptor activity / steroid binding / protein binding / protein binding / zinc ion binding / lipid binding / transcription regulator activity / sequence-specific DNA binding / metal ion binding | nucleus / nucleus / cytoplasm |
| 1460591_at | -0.92 | 1.83 | -0.55 | -1.99 | -0.16 | -5.81 | -0.36 | -4.01 | Esr1 | estrogen receptor 1 (alpha) | transcription / regulation of transcription, DNA-dependent / cell growth / negative regulation of mitosis | DNA binding / transcription factor activity / steroid hormone receptor activity / receptor activity / ligand-dependent nuclear receptor activity / ligand-dependent nuclear receptor activity / steroid binding / protein binding / protein binding / zinc ion binding / lipid binding / transcription regulator activity / sequence-specific DNA binding / metal ion binding | nucleus / nucleus / cytoplasm |
| 1419031_at | -0.72 | -0.04 | -1.01 | 2.97 | -0.82 | 1.10 | -0.49 | -2.43 | Fads2 | fatty acid desaturase 2 | lipid metabolic process / fatty acid biosynthetic process / transport / lipid biosynthetic process / oxidation reduction | stearoyl-CoA 9-desaturase activity / iron ion binding / oxidoreductase activity / oxidoreductase activity, acting on paired donors, with oxidation of a pair of donors resulting in the reduction of molecular oxygen to two molecules of water / heme binding / transition metal ion binding | membrane fraction / endoplasmic reticulum / endoplasmic reticulum membrane / membrane / integral to membrane / integral to membrane |
| 1449325_at | -0.60 | 0.39 | -0.68 | 1.51 | -0.43 | -1.96 | -0.33 | -3.32 | Fads2 | fatty acid desaturase 2 | lipid metabolic process / fatty acid biosynthetic process / transport / lipid biosynthetic process / oxidation reduction | stearoyl-CoA 9-desaturase activity / iron ion binding / oxidoreductase activity / oxidoreductase activity, acting on paired donors, with oxidation of a pair of donors resulting in the reduction of molecular oxygen to two molecules of water / heme binding / transition metal ion binding | membrane fraction / endoplasmic reticulum / endoplasmic reticulum membrane / membrane / integral to membrane / integral to membrane |
| 1426870_at | 0.97 | 1.80 | 0.65 | -1.32 | 0.52 | -2.73 | 0.20 | -5.40 | Fbxo33 | F-box protein 33 | ubiquitin cycle | --- | --- |
| 1426871_at | 1.16 | 4.43 | 0.94 | 2.41 | 0.56 | -1.64 | 0.46 | -2.68 | Fbxo33 | F-box protein 33 | ubiquitin cycle | --- | --- |
| 1451524_at | 0.14 | -5.95 | 0.02 | -6.27 | 0.67 | 0.41 | 0.38 | -3.08 | Fbxw2 | F-box and WD-40 domain protein 2 | ubiquitin cycle / Wnt receptor signaling pathway | --- | nucleus / cytoplasm |
| 1437200_at | -0.58 | 0.26 | -0.37 | -2.79 | -0.29 | -3.98 | -0.18 | -5.06 | Fcho2 | FCH domain only 2 | --- | --- | --- |
| 1418698_a_at | -0.73 | 3.33 | -0.47 | -0.45 | -0.28 | -3.50 | -0.13 | -5.42 | Fech | ferrochelatase | porphyrin biosynthetic process / porphyrin biosynthetic process / heme biosynthetic process / heme biosynthetic process / detection of UV | ferrochelatase activity / ferrochelatase activity / iron ion binding / lyase activity / metal ion binding / iron-sulfur cluster binding / 2 iron, 2 sulfur cluster binding | cytoplasm / mitochondrion / mitochondrion / mitochondrial inner membrane / mitochondrial inner membrane / membrane |
| 1449181_at | -0.78 | 4.14 | -0.32 | -2.82 | -0.37 | -2.03 | -0.20 | -4.49 | Fech | ferrochelatase | porphyrin biosynthetic process / porphyrin biosynthetic process / heme biosynthetic process / heme biosynthetic process / detection of UV | ferrochelatase activity / ferrochelatase activity / iron ion binding / lyase activity / metal ion binding / iron-sulfur cluster binding / 2 iron, 2 sulfur cluster binding | cytoplasm / mitochondrion / mitochondrion / mitochondrial inner membrane / mitochondrial inner membrane / membrane |
| 1434180_at | -1.04 | 6.42 | -0.39 | -2.00 | -0.52 | 0.03 | -0.11 | -5.61 | Fermt2 | fermitin family homolog 2 (Drosophila) | cell adhesion / regulation of cell shape | binding / protein binding | stress fiber / cytoplasm / cytoskeleton / filamentous actin |
| 1428079_at | -0.26 | -5.02 | -0.53 | -1.92 | -0.97 | 2.97 | -0.50 | -2.12 | Fgb | fibrinogen, B beta polypeptide | signal transduction / blood coagulation / blood coagulation / platelet activation / protein polymerization | receptor binding / protein binding, bridging | extracellular region / fibrinogen complex / extracellular space |
| 1450869_at | -0.85 | 3.33 | -0.49 | -1.24 | -0.28 | -4.16 | -0.34 | -3.22 | Fgf1 | fibroblast growth factor 1 | angiogenesis / angiogenesis / induction of an organ / signal transduction / multicellular organismal development / cell proliferation / cell proliferation / cell differentiation / lung development / positive regulation of epithelial cell proliferation | fibroblast growth factor receptor binding / growth factor activity / growth factor activity / heparin binding / heparin binding | proteinaceous extracellular matrix / extracellular space |
| 1453006_at | 0.06 | -6.32 | 0.03 | -6.25 | 0.72 | 2.84 | 0.24 | -4.09 | Fgfbp3 | fibroblast growth factor binding protein 3 | --- | oxidoreductase activity / growth factor binding | extracellular region |
| 1418596_at | -0.41 | -3.16 | -0.68 | 0.28 | -0.92 | 2.88 | -0.53 | -1.43 | Fgfr4 | fibroblast growth factor receptor 4 | induction of an organ / protein amino acid phosphorylation / signal transduction / lung development | nucleotide binding / protein kinase activity / protein tyrosine kinase activity / transmembrane receptor protein tyrosine kinase activity / receptor activity / fibroblast growth factor receptor activity / ATP binding / kinase activity / transferase activity | extracellular space / membrane / integral to membrane |
| 1439190_at | -0.12 | -6.32 | -0.50 | -4.48 | -1.26 | 0.87 | -0.86 | -1.87 | Fhad1 | forkhead-associated (FHA) phosphopeptide binding domain 1 | --- | --- | --- |
| 1433481_at | -0.90 | -0.43 | -0.90 | -0.34 | -1.26 | 2.44 | -0.89 | -0.38 | Fkbp14 | FK506 binding protein 14 | protein folding | peptidyl-prolyl cis-trans isomerase activity / calcium ion binding / isomerase activity | endoplasmic reticulum / endoplasmic reticulum lumen |
| 1416803_at | -0.28 | -4.15 | -0.39 | -2.51 | -0.49 | -1.03 | -0.62 | 0.88 | Fkbp7 | FK506 binding protein 7 | protein folding | peptidyl-prolyl cis-trans isomerase activity / calcium ion binding / calcium ion binding / protein binding / isomerase activity | extracellular space / endoplasmic reticulum / endoplasmic reticulum / endoplasmic reticulum lumen |
| 1455096_at | -0.46 | -2.64 | -0.87 | 2.21 | -0.63 | -0.38 | -0.65 | -0.15 | Flrt2 | fibronectin leucine rich transmembrane protein 2 | --- | protein binding | integral to membrane |
| 1429310_at | 0.76 | -0.44 | 0.85 | 0.55 | 0.77 | -0.27 | 0.80 | 0.09 | Flrt3 | fibronectin leucine rich transmembrane protein 3 | --- | protein binding | integral to membrane |
| 1424123_at | -0.09 | -6.32 | -0.62 | -1.60 | -0.81 | 0.37 | -0.43 | -3.44 | Flvcr2 | feline leukemia virus subgroup C cellular receptor family, member 2 | transport | receptor activity / transporter activity | plasma membrane / membrane / integral to membrane |
| 1435459_at | -1.16 | 3.51 | -0.79 | 0.09 | -0.66 | -1.22 | -0.70 | -0.73 | Fmo2 | flavin containing monooxygenase 2 | oxygen and reactive oxygen species metabolic process / oxidation reduction | magnesium ion binding / magnesium ion binding / monooxygenase activity / monooxygenase activity / flavin-containing monooxygenase activity / flavin-containing monooxygenase activity / oxidoreductase activity / FAD binding / NADP binding | extracellular space / endoplasmic reticulum / endoplasmic reticulum membrane / microsome / microsome / membrane / integral to membrane / integral to membrane / intrinsic to endoplasmic reticulum membrane |
| 1426903_at | -0.02 | -6.44 | -0.50 | -2.29 | -0.68 | -0.27 | -0.78 | 0.88 | Fndc3a | fibronectin type III domain containing 3a | spermatid development / fertilization / cell-cell adhesion | --- | acrosome / cytosol / vesicle membrane / cytoplasmic vesicle / vesicular fraction |
| 1439189_at | 0.72 | 3.68 | 0.63 | 2.37 | 0.78 | 4.43 | 0.41 | -1.07 | Fnip2 | folliculin interacting protein 2 | --- | --- | --- |
| 1422134_at | 0.97 | 1.09 | 0.70 | -1.39 | 0.63 | -2.07 | 0.30 | -4.90 | Fosb | FBJ osteosarcoma oncogene B | regulation of transcription, DNA-dependent / cellular process / regulation of cell cycle | DNA binding / DNA binding / transcription factor activity / sequence-specific DNA binding / protein dimerization activity | intracellular / nucleus |
| 1417487_at | 0.82 | 1.68 | 0.59 | -0.94 | 0.93 | 2.79 | 0.35 | -3.71 | Fosl1 | fos-like antigen 1 | regulation of transcription, DNA-dependent / positive regulation of transcription from RNA polymerase II promoter, mitotic | DNA binding / transcription factor activity / transcription activator activity / sequence-specific DNA binding / protein dimerization activity | nucleus |
| 1422833_at | -0.76 | 1.95 | -0.29 | -4.22 | -0.47 | -1.71 | -0.24 | -4.61 | Foxa2 | forkhead box A2 | ectoderm formation / transcription / regulation of transcription, DNA-dependent / regulation of transcription, DNA-dependent / pattern specification process / anterior/posterior pattern formation / dorsal/ventral pattern formation / cell differentiation in hindbrain / cell differentiation in hindbrain / lung development / epithelial cell differentiation / endocrine pancreas development / negative regulation of neuron differentiation / positive regulation of neuron differentiation / positive regulation of transcription from RNA polymerase II promoter / positive regulation of transcription from RNA polymerase II promoter / cell development / anatomical structure formation / neuron fate specification / branching morphogenesis of a tube | DNA binding / DNA binding / transcription factor activity / transcription factor activity / RNA polymerase II transcription factor activity, enhancer binding / sequence-specific DNA binding | nucleus / nucleus |
| 1440108_at | -0.43 | -3.27 | -0.21 | -5.39 | -0.91 | 2.23 | -0.86 | 1.73 | Foxp2 | forkhead box P2 | negative regulation of transcription from RNA polymerase II promoter / positive regulation of mesenchymal cell proliferation / transcription / regulation of transcription, DNA-dependent / skeletal muscle development / embryonic development / post-embryonic development / negative regulation of transcription / cerebellum development / caudate nucleus development / putamen development / lung development / growth / vocal learning / camera-type eye development / alveolus development / smooth muscle development / positive regulation of epithelial cell proliferation / righting reflex | nucleic acid binding / DNA binding / DNA binding / DNA binding / transcription factor activity / transcription factor activity / protein binding / zinc ion binding / transcription repressor activity / specific transcriptional repressor activity / protein homodimerization activity / protein homodimerization activity / sequence-specific DNA binding / metal ion binding / protein heterodimerization activity | intracellular / nucleus |
| 1436413_at | 0.02 | -6.43 | -0.36 | -2.50 | -0.35 | -2.61 | -0.72 | 2.73 | Frk | fyn-related kinase | protein amino acid phosphorylation / protein amino acid phosphorylation / intracellular signaling cascade / negative regulation of cell proliferation / regulation of cell cycle | nucleotide binding / protein kinase activity / protein kinase activity / protein tyrosine kinase activity / non-membrane spanning protein tyrosine kinase activity / non-membrane spanning protein tyrosine kinase activity / protein binding / ATP binding / kinase activity / transferase activity | extracellular space / intracellular / nucleus / cytoplasm |
| 1419301_at | -0.63 | -4.09 | -0.68 | -3.60 | -1.25 | 0.15 | -0.44 | -4.86 | Fzd4 | frizzled homolog 4 (Drosophila) | signal transduction / cell surface receptor linked signal transduction / G-protein coupled receptor protein signaling pathway / multicellular organismal development / Wnt receptor signaling pathway | signal transducer activity / receptor activity / non-G-protein coupled 7TM receptor activity / G-protein coupled receptor activity / protein binding | extracellular space / membrane / integral to membrane / integral to membrane |
| 1422327_s_at | -0.67 | 0.29 | -0.34 | -3.86 | 0.19 | -5.42 | -0.06 | -5.99 | G6pd2 / G6pdx | glucose-6-phosphate dehydrogenase 2 / glucose-6-phosphate dehydrogenase X-linked | cytokine production / angiotensin mediated vasoconstriction involved in regulation of systemic arterial blood pressure / vasodilation by angiotensin involved in regulation of systemic arterial blood pressure / carbohydrate metabolic process / glucose metabolic process / NADP biosynthetic process / NADP biosynthetic process / glutathione metabolic process / response to oxidative stress / metabolic process / pentose biosynthetic process / pentose biosynthetic process / interleukin-10 production / interleukin-12 production / regulation of multicellular organism growth / erythrocyte development | catalytic activity / glucose-6-phosphate dehydrogenase activity / glucose-6-phosphate dehydrogenase activity / glucose-6-phosphate dehydrogenase activity / binding / oxidoreductase activity / protein homodimerization activity | --- |
| 1419761_a_at | 0.73 | 0.59 | 0.04 | -6.25 | 0.00 | -6.31 | -0.08 | -5.96 | Gabpb1 | GA repeat binding protein, beta 1 | transcription / regulation of transcription, DNA-dependent | DNA binding / transcription factor activity | nucleus |
| 1449519_at | 0.77 | -0.46 | 0.41 | -4.02 | 0.88 | 0.64 | 0.34 | -4.49 | Gadd45a | growth arrest and DNA-damage-inducible 45 alpha | G2/M transition of mitotic cell cycle / negative regulation of protein kinase activity / response to DNA damage stimulus / cell cycle / cell cycle arrest / centrosome cycle / regulation of cell cycle | protein binding | nucleus |
| 1449773_s_at | 1.82 | 5.98 | 1.64 | 4.82 | 1.57 | 4.43 | 0.88 | -0.56 | Gadd45b | growth arrest and DNA-damage-inducible 45 beta | activation of MAPKK activity / negative regulation of protein kinase activity / apoptosis / multicellular organismal development / cell differentiation / regulation of cell cycle | protein binding | nucleus |
| 1450971_at | 1.67 | 4.91 | 1.47 | 3.59 | 1.59 | 4.36 | 0.82 | -1.18 | Gadd45b | growth arrest and DNA-damage-inducible 45 beta | activation of MAPKK activity / negative regulation of protein kinase activity / apoptosis / multicellular organismal development / cell differentiation / regulation of cell cycle | protein binding | nucleus |
| 1453851_a_at | 1.27 | 1.52 | 1.40 | 2.44 | 1.11 | 0.40 | 0.56 | -3.67 | Gadd45g | growth arrest and DNA-damage-inducible 45 gamma | activation of MAPKK activity / negative regulation of protein kinase activity / apoptosis / multicellular organismal development / cell differentiation / interferon-gamma biosynthetic process / T-helper 1 cell differentiation / regulation of cell cycle | protein binding | nucleus |
| 1434754_at | 0.92 | 7.10 | 0.68 | 3.89 | 0.25 | -3.46 | 0.32 | -2.01 | Garnl4 | GTPase activating RANGAP domain-like 4 | regulation of small GTPase mediated signal transduction | GTPase activator activity | intracellular / cytoplasm |
| 1418240_at | 1.52 | 4.22 | 0.17 | -5.93 | 0.13 | -6.12 | -0.08 | -6.03 | Gbp2 | guanylate nucleotide binding protein 2 | immune response / immune response | nucleotide binding / GTPase activity / GTPase activity / GTP binding / GTP binding | plasma membrane / membrane |
| 1435906_x_at | 1.43 | 3.81 | 0.44 | -4.20 | 0.27 | -5.44 | 0.19 | -5.65 | Gbp2 | guanylate nucleotide binding protein 2 | immune response / immune response | nucleotide binding / GTPase activity / GTPase activity / GTP binding / GTP binding | plasma membrane / membrane |
| 1418392_a_at | 0.78 | 3.89 | 0.39 | -1.82 | 0.29 | -3.52 | 0.04 | -6.02 | Gbp3 | guanylate nucleotide binding protein 3 | immune response / immune response | nucleotide binding / GTPase activity / GTPase activity / GTP binding / GTP binding | cytoplasm / cytosol |
| 1424698_s_at | -0.86 | 3.95 | -0.86 | 3.91 | -0.40 | -2.24 | -0.07 | -5.94 | Gca | grancalcin | --- | calcium ion binding / calcium ion binding | cytoplasm / cytosol / membrane |
| 1424296_at | 0.23 | -5.31 | 0.71 | 0.22 | 0.88 | 2.00 | 0.50 | -2.20 | Gclc | glutamate-cysteine ligase, catalytic subunit | cysteine metabolic process / cysteine metabolic process / glutamate metabolic process / glutamate metabolic process / glutathione metabolic process / glutathione biosynthetic process / glutathione biosynthetic process / glutathione biosynthetic process / glutathione biosynthetic process / anti-apoptosis / anti-apoptosis / response to oxidative stress / response to oxidative stress / response to heat / response to heat / response to xenobiotic stimulus / response to hormone stimulus / response to hormone stimulus / negative regulation of transcription / negative regulation of transcription / L-ascorbic acid metabolic process / regulation of cell redox homeostasis / regulation of cell redox homeostasis / negative regulation of protein ubiquitination / positive regulation of proteasomal ubiquitin-dependent protein catabolic process / negative regulation of apoptosis / response to arsenic / regulation of blood vessel size / regulation of blood vessel size / regulation of mitochondrial depolarization | magnesium ion binding / magnesium ion binding / glutamate-cysteine ligase activity / glutamate-cysteine ligase activity / glutamate-cysteine ligase activity / glutamate-cysteine ligase activity / glutamate binding / glutamate binding / ligase activity / ADP binding / ADP binding / protein heterodimerization activity / coenzyme binding / coenzyme binding | cytosol / glutamate-cysteine ligase complex |
| 1455959_s_at | 0.44 | -2.47 | 0.64 | 0.24 | 0.85 | 2.61 | 0.47 | -1.96 | Gclc | Glutamate-cysteine ligase, catalytic subunit | cysteine metabolic process / cysteine metabolic process / glutamate metabolic process / glutamate metabolic process / glutathione metabolic process / glutathione biosynthetic process / glutathione biosynthetic process / glutathione biosynthetic process / glutathione biosynthetic process / anti-apoptosis / anti-apoptosis / response to oxidative stress / response to oxidative stress / response to heat / response to heat / response to xenobiotic stimulus / response to hormone stimulus / response to hormone stimulus / negative regulation of transcription / negative regulation of transcription / L-ascorbic acid metabolic process / regulation of cell redox homeostasis / regulation of cell redox homeostasis / negative regulation of protein ubiquitination / positive regulation of proteasomal ubiquitin-dependent protein catabolic process / negative regulation of apoptosis / response to arsenic / regulation of blood vessel size / regulation of blood vessel size / regulation of mitochondrial depolarization | magnesium ion binding / magnesium ion binding / glutamate-cysteine ligase activity / glutamate-cysteine ligase activity / glutamate-cysteine ligase activity / glutamate-cysteine ligase activity / glutamate binding / glutamate binding / ligase activity / ADP binding / ADP binding / protein heterodimerization activity / coenzyme binding / coenzyme binding | cytosol / glutamate-cysteine ligase complex |
| 1418627_at | -0.14 | -6.13 | 1.12 | 2.93 | 1.33 | 4.51 | 1.02 | 1.99 | Gclm | glutamate-cysteine ligase , modifier subunit | cysteine metabolic process / glutamate metabolic process / glutamate metabolic process / glutathione metabolic process / glutathione biosynthetic process / glutathione biosynthetic process / glutathione biosynthetic process / glutathione biosynthetic process / response to oxidative stress / response to oxidative stress / response to oxidative stress / positive regulation of glutamate-cysteine ligase activity / response to drug / response to drug / negative regulation of apoptosis / negative regulation of neuron apoptosis / regulation of blood vessel size / regulation of blood vessel size / response to nitrosative stress / regulation of mitochondrial depolarization | glutamate-cysteine ligase activity / glutamate-cysteine ligase activity / protein binding / oxidoreductase activity / ligase activity / glutamate-cysteine ligase catalytic subunit binding / glutamate-cysteine ligase catalytic subunit binding / glutamate-cysteine ligase catalytic subunit binding / protein heterodimerization activity | soluble fraction / soluble fraction / glutamate-cysteine ligase complex |
| 1418949_at | 1.46 | 4.14 | 1.46 | 4.07 | 1.23 | 2.50 | 0.81 | -0.90 | Gdf15 | growth differentiation factor 15 | --- | cytokine activity / growth factor activity | extracellular region / extracellular space / extracellular space |
| 1418753_at | -0.45 | -0.71 | -0.73 | 3.36 | -0.71 | 3.14 | -0.47 | -0.38 | Gfpt2 | glutamine fructose-6-phosphate transaminase 2 | carbohydrate metabolic process / glutamine metabolic process / glutamine metabolic process / metabolic process / carbohydrate biosynthetic process | glutamine-fructose-6-phosphate transaminase (isomerizing) activity / glutamine-fructose-6-phosphate transaminase (isomerizing) activity / sugar binding / transaminase activity / transferase activity | cytoplasm |
| 1416592_at | -0.76 | 0.30 | -0.26 | -5.08 | -0.42 | -3.46 | -0.41 | -3.42 | Glrx | glutaredoxin | transport / cell redox homeostasis / oxidation reduction | electron carrier activity / protein disulfide oxidoreductase activity / transferase activity / protein-disulfide reductase (glutathione) activity | cytoplasm |
| 1428270_at | -0.61 | 2.47 | -0.31 | -2.48 | -0.17 | -4.93 | -0.11 | -5.47 | Glt8d1 | glycosyltransferase 8 domain containing 1 | carbohydrate biosynthetic process | transferase activity / transferase activity, transferring glycosyl groups / transferase activity, transferring hexosyl groups | membrane / integral to membrane |
| 1455558_at | -0.69 | 1.54 | -0.32 | -3.61 | -0.37 | -2.85 | -0.33 | -3.31 | Gm114 | gene model 114, (NCBI) | --- | --- | --- |
| 1435188_at | 0.83 | 0.96 | 0.41 | -3.54 | 0.46 | -3.04 | 0.39 | -3.61 | Gm129 | gene model 129, (NCBI) | --- | --- | mitochondrial small ribosomal subunit |
| 1436348_at | 0.66 | 2.35 | 0.65 | 2.26 | 0.43 | -1.18 | 0.35 | -2.32 | Gm505 | Gene model 505, (NCBI) | --- | --- | --- |
| 1454921_at | 0.22 | -5.12 | 0.20 | -5.12 | 0.23 | -4.84 | 0.61 | 0.33 | Gm561 | gene model 561, (NCBI) | --- | --- | --- |
| 1419316_s_at | -0.04 | -6.38 | 0.45 | -0.65 | 0.71 | 3.32 | 0.52 | 0.47 | Gnb1l | guanine nucleotide binding protein (G protein), beta polypeptide 1-like | social behavior | --- | --- |
| 1448942_at | -0.70 | 1.45 | -0.43 | -2.11 | -0.60 | 0.22 | -0.24 | -4.56 | Gng11 | guanine nucleotide binding protein (G protein), gamma 11 | signal transduction / signal transduction / G-protein coupled receptor protein signaling pathway / G-protein coupled receptor protein signaling pathway | GTPase activity / signal transducer activity / signal transducer activity | heterotrimeric G-protein complex / heterotrimeric G-protein complex / plasma membrane / membrane |
| 1448163_at | 0.16 | -5.01 | 0.59 | 3.02 | 0.77 | 5.45 | 0.60 | 2.99 | Gnpda1 | glucosamine-6-phosphate deaminase 1 | carbohydrate metabolic process / fructose 6-phosphate metabolic process / glucosamine metabolic process / glucosamine catabolic process / N-acetylglucosamine metabolic process / generation of precursor metabolites and energy / acrosome reaction / fructose biosynthetic process | glucosamine-6-phosphate deaminase activity / glucosamine-6-phosphate deaminase activity / hydrolase activity | cytoplasm / cytoplasm |
| 1455065_x_at | 0.35 | -2.91 | 0.74 | 2.75 | 0.98 | 5.38 | 0.88 | 4.13 | Gnpda1 | glucosamine-6-phosphate deaminase 1 | carbohydrate metabolic process / fructose 6-phosphate metabolic process / glucosamine metabolic process / glucosamine catabolic process / N-acetylglucosamine metabolic process / generation of precursor metabolites and energy / acrosome reaction / fructose biosynthetic process | glucosamine-6-phosphate deaminase activity / glucosamine-6-phosphate deaminase activity / hydrolase activity | cytoplasm / cytoplasm |
| 1450153_at | -0.62 | 0.11 | -0.14 | -5.73 | 0.06 | -6.22 | -0.25 | -4.50 | Gopc | golgi associated PDZ and coiled-coil motif containing | transport / autophagy / protein transport / protein homooligomerization | small GTPase regulator activity / protein binding / protein binding / protein binding / cystic fibrosis transmembrane conductance regulator binding / syntaxin-6 binding | Golgi membrane / membrane fraction / cytoplasm / cytoplasm / Golgi apparatus / Golgi apparatus / membrane / synaptosome / cell junction / trans-Golgi network transport vesicle / cell projection / synapse / postsynaptic membrane |
| 1419499_at | -1.01 | 5.55 | -0.79 | 3.01 | -0.64 | 1.11 | -0.51 | -0.54 | Gpam | glycerol-3-phosphate acyltransferase, mitochondrial | fatty acid metabolic process / metabolic process / phospholipid biosynthetic process / positive regulation of multicellular organism growth | glycerol-3-phosphate O-acyltransferase activity / glycerol-3-phosphate O-acyltransferase activity / acyltransferase activity / transferase activity | mitochondrion / mitochondrion / mitochondrial outer membrane / mitochondrial inner membrane / membrane / integral to membrane |
| 1439345_at | 0.32 | -5.33 | 1.34 | 3.00 | 0.90 | -0.38 | 1.05 | 0.87 | Gpnmb | glycoprotein (transmembrane) nmb | osteoblast differentiation / cell adhesion / metabolic process / bone mineralization | integrin binding / heparin binding | integral to plasma membrane / endomembrane system / membrane / integral to membrane / cytoplasmic membrane-bounded vesicle / cytoplasmic vesicle membrane / cytoplasmic vesicle / melanosome |
| 1448303_at | 0.13 | -6.11 | 0.48 | -2.74 | 0.84 | 1.13 | 0.82 | 0.99 | Gpnmb | glycoprotein (transmembrane) nmb | osteoblast differentiation / regulation of transcription, DNA-dependent / regulation of transcription, DNA-dependent / regulation of transcription, DNA-dependent / cell adhesion / nervous system development / metabolic process / bone mineralization / response to drug | DNA binding / DNA binding / transcription factor activity / transcription factor activity / transcription factor activity / integrin binding / heparin binding / sequence-specific DNA binding / protein dimerization activity | nucleus / nucleus / nucleus / transcription factor complex / integral to plasma membrane / endomembrane system / membrane / integral to membrane / cytoplasmic membrane-bounded vesicle / cytoplasmic vesicle membrane / cytoplasmic vesicle / melanosome |
| 1418379_s_at | -0.52 | -2.75 | -0.78 | 0.14 | -0.61 | -1.63 | -0.33 | -4.42 | Gpr124 | G protein-coupled receptor 124 | signal transduction / G-protein coupled receptor protein signaling pathway / neuropeptide signaling pathway | signal transducer activity / receptor activity / G-protein coupled receptor activity / protein binding | plasma membrane / plasma membrane / membrane / integral to membrane |
| 1450881_s_at | 1.93 | 9.54 | 1.94 | 9.14 | 1.52 | 6.95 | 1.07 | 3.46 | Gpr137b | G protein-coupled receptor 137B | --- | --- | membrane / integral to membrane / integral to membrane |
| 1429775_a_at | 1.61 | 6.40 | 2.18 | 9.02 | 2.09 | 8.69 | 1.23 | 3.46 | Gpr137b / Gpr137b-ps / LOC100044979 | G protein-coupled receptor 137B / G protein-coupled receptor 137B, pseudogene / similar to Gpr137b protein | --- | --- | membrane / integral to membrane / integral to membrane |
| 1439255_s_at | 1.85 | 8.33 | 2.16 | 9.39 | 2.22 | 9.72 | 1.32 | 4.57 | Gpr137b / Gpr137b-ps / LOC100044979 | G protein-coupled receptor 137B / G protein-coupled receptor 137B, pseudogene / similar to Gpr137b protein | --- | --- | membrane / integral to membrane / integral to membrane |
| 1450882_s_at | 1.64 | 7.13 | 1.90 | 8.22 | 2.15 | 9.44 | 1.44 | 5.31 | Gpr137b / Gpr137b-ps / LOC100044979 | G protein-coupled receptor 137B / G protein-coupled receptor 137B, pseudogene / similar to Gpr137b protein | --- | --- | membrane / integral to membrane / integral to membrane |
| 1449670_x_at | 1.38 | 5.62 | 1.55 | 6.57 | 1.56 | 6.70 | 0.58 | -1.73 | Gpr137b / LOC100044979 | G protein-coupled receptor 137B / similar to Gpr137b protein | --- | --- | membrane / integral to membrane / integral to membrane |
| 1439256_x_at | 1.70 | 7.82 | 1.83 | 8.20 | 1.75 | 7.89 | 1.11 | 3.37 | Gpr137b-ps | G protein-coupled receptor 137B, pseudogene | --- | --- | membrane / integral to membrane / integral to membrane |
| 1452353_at | -1.05 | 8.82 | -0.86 | 6.59 | -0.89 | 6.98 | -0.74 | 4.83 | Gpr155 | G protein-coupled receptor 155 | intracellular signaling cascade | receptor activity | integral to membrane |
| 1420364_at | -0.62 | 3.65 | -0.69 | 4.63 | -0.38 | -0.67 | -0.32 | -1.65 | Gpr87 | G protein-coupled receptor 87 | signal transduction / G-protein coupled receptor protein signaling pathway | rhodopsin-like receptor activity / signal transducer activity / receptor activity / G-protein coupled receptor activity / purinergic nucleotide receptor activity, G-protein coupled | plasma membrane / membrane / integral to membrane / integral to membrane |
| 1438385_s_at | 0.65 | 1.92 | 0.77 | 3.45 | 0.70 | 2.49 | 0.39 | -1.87 | Gpt2 | glutamic pyruvate transaminase (alanine aminotransferase) 2 | biosynthetic process | catalytic activity / alanine transaminase activity / transaminase activity / transferase activity / transferase activity, transferring nitrogenous groups / 1-aminocyclopropane-1-carboxylate synthase activity / pyridoxal phosphate binding | --- |
| 1460206_at | 0.68 | 1.02 | 0.37 | -3.09 | 0.60 | 0.01 | 0.14 | -5.56 | Grasp | GRP1 (general receptor for phosphoinositides 1)-associated scaffold protein | intracellular protein transport / signal transduction / intracellular signaling cascade | protein binding / protein binding / protein binding | nucleus / nuclear envelope lumen / cytoplasm / cytoplasm / plasma membrane / plasma membrane / plasma membrane / membrane |
| 1448227_at | -0.69 | 0.86 | -0.54 | -1.08 | -0.25 | -4.78 | -0.02 | -6.08 | Grb7 | growth factor receptor bound protein 7 | signal transduction / signal transduction | receptor activity / SH3/SH2 adaptor activity / protein binding | cytosol |
| 1425891_a_at | -0.74 | 1.66 | -0.40 | -2.73 | -0.15 | -5.64 | -0.17 | -5.34 | Grtp1 | GH regulated TBC protein 1 | regulation of Rab GTPase activity | GTPase activator activity / Rab GTPase activator activity / hormone activity | extracellular region / intracellular |
| 1452171_at | 0.34 | -3.94 | 0.13 | -5.85 | 0.65 | 0.14 | 0.28 | -4.26 | Grwd1 | glutamate-rich WD repeat containing 1 | --- | --- | nucleus |
| 1415812_at | 0.33 | -5.55 | 0.82 | -2.05 | 1.85 | 4.65 | 1.05 | -0.31 | Gsn | gelsolin | vesicle-mediated transport / actin filament polymerization / actin filament severing / actin filament severing / barbed-end actin filament capping / barbed-end actin filament capping | actin binding / structural molecule activity / calcium ion binding / calcium ion binding | extracellular region / extracellular region / extracellular space / cytoplasm / cytoplasm / cytosol / cytoskeleton / actin cytoskeleton / lamellipodium |
| 1436991_x_at | 0.51 | -4.91 | 0.83 | -2.81 | 2.14 | 4.65 | 1.25 | -0.12 | Gsn | gelsolin | vesicle-mediated transport / actin filament polymerization / actin filament severing / actin filament severing / barbed-end actin filament capping / barbed-end actin filament capping | actin binding / structural molecule activity / calcium ion binding / calcium ion binding | extracellular region / extracellular region / extracellular space / cytoplasm / cytoplasm / cytosol / cytoskeleton / actin cytoskeleton / lamellipodium |
| 1437171_x_at | 0.38 | -5.78 | 0.81 | -3.68 | 2.15 | 3.20 | 1.15 | -1.72 | Gsn | gelsolin | vesicle-mediated transport / actin filament polymerization / actin filament severing / actin filament severing / barbed-end actin filament capping / barbed-end actin filament capping | actin binding / structural molecule activity / calcium ion binding / calcium ion binding | extracellular region / extracellular region / extracellular space / cytoplasm / cytoplasm / cytosol / cytoskeleton / actin cytoskeleton / lamellipodium |
| 1456312_x_at | 0.47 | -5.38 | 0.88 | -3.19 | 1.89 | 2.23 | 1.00 | -2.45 | Gsn | gelsolin | vesicle-mediated transport / actin filament polymerization / actin filament severing / actin filament severing / barbed-end actin filament capping / barbed-end actin filament capping | actin binding / structural molecule activity / calcium ion binding / calcium ion binding | extracellular region / extracellular region / extracellular space / cytoplasm / cytoplasm / cytosol / cytoskeleton / actin cytoskeleton / lamellipodium |
| 1456569_x_at | 0.27 | -4.67 | 0.32 | -3.93 | 0.77 | 1.72 | 0.56 | -0.78 | Gsn | gelsolin | vesicle-mediated transport / actin filament polymerization / actin filament severing / actin filament severing / barbed-end actin filament capping / barbed-end actin filament capping | actin binding / structural molecule activity / calcium ion binding / calcium ion binding | extracellular region / extracellular region / extracellular space / cytoplasm / cytoplasm / cytosol / cytoskeleton / actin cytoskeleton / lamellipodium |
| 1423436_at | 0.01 | -6.45 | 0.31 | -4.31 | 0.81 | 1.57 | 0.88 | 2.20 | Gsta3 | glutathione S-transferase, alpha 3 | metabolic process | glutathione transferase activity / transferase activity | cytoplasm |
| 1416368_at | -0.26 | -5.24 | 0.55 | -2.08 | 0.80 | 0.61 | 0.99 | 2.39 | Gsta4 | glutathione S-transferase, alpha 4 | metabolic process | glutathione transferase activity / glutathione transferase activity / glutathione transferase activity / transferase activity | cytoplasm |
| 1419072_at | -0.89 | 1.14 | -0.61 | -1.67 | -0.43 | -3.60 | -0.51 | -2.65 | Gstm7 | glutathione S-transferase, mu 7 | metabolic process / metabolic process | glutathione transferase activity / glutathione transferase activity / transferase activity | --- |
| 1453708_a_at | 0.05 | -6.40 | 0.77 | -0.38 | 0.93 | 1.13 | 0.94 | 1.15 | Gsto2 | glutathione S-transferase omega 2 | metabolic process | glutathione transferase activity / transferase activity | cytoplasm |
| 1417883_at | 0.16 | -5.79 | 0.15 | -5.72 | 0.06 | -6.21 | 0.67 | 0.20 | Gstt2 | glutathione S-transferase, theta 2 | glutathione metabolic process / glutathione metabolic process | glutathione transferase activity / glutathione transferase activity / transferase activity | nucleus / cytoplasm |
| 1433511_at | -0.57 | 0.74 | -0.63 | 1.69 | -0.18 | -5.05 | -0.23 | -4.21 | Gtf2a1 | General transcription factor II A, 1 | transcription / regulation of transcription, DNA-dependent / transcription initiation from RNA polymerase II promoter | RNA polymerase II transcription factor activity | nucleus / transcription factor TFIIA complex |
| 1448884_at | -0.58 | 0.13 | -0.33 | -3.36 | -0.64 | 1.09 | -0.38 | -2.49 | Gtf2e2 | general transcription factor II E, polypeptide 2 (beta subunit) | transcription / regulation of transcription, DNA-dependent / transcription initiation from RNA polymerase II promoter / transcription initiation from RNA polymerase II promoter | DNA binding / RNA polymerase II transcription factor activity / RNA polymerase II transcription factor activity / translation initiation factor activity / protein binding / protein binding | nucleus / transcription factor TFIIE complex / transcription factor TFIIE complex |
| 1416969_at | 0.20 | -5.28 | 0.21 | -5.03 | 0.74 | 1.88 | 0.40 | -2.52 | Gtse1 | G two S phase expressed protein 1 | --- | --- | cytoplasm / microtubule |
| 1449875_s_at | -0.38 | -2.68 | -0.63 | 1.02 | -0.32 | -3.39 | -0.22 | -4.66 | H2-T10 / H2-T17 / H2-T22 / H2-T9 | histocompatibility 2, T region locus 10 / histocompatibility 2, T region locus 17 / histocompatibility 2, T region locus 22 / histocompatibility 2, T region locus 9 | defense response / immune response / antigen processing and presentation | --- | membrane / integral to membrane / MHC class I protein complex |
| 1420420_at | -0.24 | -4.32 | -0.12 | -5.69 | -0.52 | 0.38 | -0.83 | 4.41 | Hao1 | hydroxyacid oxidase 1, liver | metabolic process / oxidative photosynthetic carbon pathway | catalytic activity / (S)-2-hydroxy-acid oxidase activity / electron carrier activity / FMN binding / oxidoreductase activity | peroxisome / peroxisome |
| 1439815_at | -0.69 | 3.38 | -0.18 | -4.87 | -0.20 | -4.61 | -0.23 | -3.91 | Heatr5b | HEAT repeat containing 5B | --- | binding | --- |
| 1435626_a_at | 0.95 | 0.67 | 0.28 | -5.27 | -0.22 | -5.68 | 0.10 | -5.97 | Herpud1 | homocysteine-inducible, endoplasmic reticulum stress-inducible, ubiquitin-like domain member 1 | protein modification process / response to stress / response to unfolded protein / response to unfolded protein | --- | endoplasmic reticulum / endoplasmic reticulum membrane / endoplasmic reticulum membrane / membrane / integral to membrane / integral to membrane |
| 1448185_at | 1.05 | 1.04 | 0.40 | -4.52 | -0.05 | -6.28 | 0.01 | -6.09 | Herpud1 | homocysteine-inducible, endoplasmic reticulum stress-inducible, ubiquitin-like domain member 1 | protein modification process / response to stress / response to unfolded protein / response to unfolded protein | --- | endoplasmic reticulum / endoplasmic reticulum membrane / endoplasmic reticulum membrane / membrane / integral to membrane / integral to membrane |
| 1449024_a_at | 0.14 | -5.30 | 0.28 | -2.86 | 0.42 | -0.11 | 0.58 | 2.57 | Hexa | hexosaminidase A | skeletal development / carbohydrate metabolic process / ganglioside catabolic process / lysosome organization and biogenesis / lysosome organization and biogenesis / sensory perception of sound / locomotory behavior / adult walking behavior / metabolic process / sequestering of lipid / sexual reproduction / glycosaminoglycan metabolic process / myelination / neuromuscular process controlling posture / neuromuscular process controlling balance | catalytic activity / beta-N-acetylhexosaminidase activity / beta-N-acetylhexosaminidase activity / beta-N-acetylhexosaminidase activity / beta-N-acetylhexosaminidase activity / hydrolase activity / hydrolase activity, acting on glycosyl bonds / cation binding / protein heterodimerization activity / protein heterodimerization activity | lysosome / lysosome / membrane |
| 1429025_a_at | -0.70 | 0.28 | -0.60 | -0.75 | -0.34 | -4.01 | -0.13 | -5.72 | Hexim2 / LOC100044959 | hexamthylene bis-acetamide inducible 2 / similar to hexamthylene bis-acetamide inducible 2 | negative regulation of transcription from RNA polymerase II promoter / transcription / regulation of transcription, DNA-dependent / negative regulation of cyclin-dependent protein kinase activity | cyclin-dependent protein kinase inhibitor activity / protein binding / transcription repressor activity / snRNA binding | nucleus |
| 1418405_at | -0.35 | -5.83 | -0.52 | -5.02 | -1.53 | 0.44 | -0.91 | -2.94 | Hgfac | hepatocyte growth factor activator | proteolysis | catalytic activity / serine-type endopeptidase activity / protein binding / peptidase activity / hydrolase activity | extracellular region / extracellular space |
| 1447854_s_at | -0.50 | -2.83 | -0.60 | -1.64 | -0.73 | -0.26 | -0.78 | 0.25 | Hist2h2be | histone cluster 2, H2be | nucleosome assembly | DNA binding | nucleosome / nucleus / chromosome |
| 1455095_at | -0.66 | -0.38 | -0.76 | 0.83 | -0.46 | -2.74 | -0.88 | 2.06 | Hist2h2be | histone cluster 2, H2be | nucleosome assembly | DNA binding | nucleosome / nucleus / chromosome |
| 1426475_at | -0.61 | 2.14 | -0.14 | -5.37 | 0.10 | -5.83 | 0.15 | -5.18 | Hmbs | hydroxymethylbilane synthase | porphyrin biosynthetic process / heme biosynthetic process / tetrapyrrole biosynthetic process | hydroxymethylbilane synthase activity / transferase activity | --- |
| 1427229_at | -0.60 | -2.62 | -0.97 | 0.86 | -0.78 | -0.83 | -0.42 | -4.03 | Hmgcr | 3-hydroxy-3-methylglutaryl-Coenzyme A reductase | steroid biosynthetic process / cholesterol biosynthetic process / cholesterol biosynthetic process / isoprenoid biosynthetic process / visual learning / lipid biosynthetic process / embryonic development / coenzyme A metabolic process / sterol biosynthetic process / negative regulation of MAP kinase activity | hydroxymethylglutaryl-CoA reductase (NADPH) activity / hydroxymethylglutaryl-CoA reductase (NADPH) activity / oxidoreductase activity / oxidoreductase activity, acting on the CH-OH group of donors, NAD or NADP as acceptor / hydroxymethylglutaryl-CoA reductase activity / protein homodimerization activity / NADP binding / coenzyme binding | peroxisome / peroxisomal membrane / endoplasmic reticulum / endoplasmic reticulum membrane / microsome / membrane / integral to membrane |
| 1441536_at | -0.65 | -4.22 | -1.08 | -1.45 | -1.73 | 2.29 | -1.18 | -0.83 | Hmgcs1 | 3-hydroxy-3-methylglutaryl-Coenzyme A synthase 1 | acetyl-CoA metabolic process / steroid biosynthetic process / cholesterol biosynthetic process / metabolic process / isoprenoid biosynthetic process / lipid biosynthetic process / sterol biosynthetic process | catalytic activity / hydroxymethylglutaryl-CoA synthase activity / hydroxymethylglutaryl-CoA synthase activity / transferase activity | cytoplasm |
| 1433444_at | -0.18 | -5.53 | -0.50 | -1.40 | -0.76 | 1.92 | -0.55 | -0.62 | Hmgcs1 / LOC100040592 | 3-hydroxy-3-methylglutaryl-Coenzyme A synthase 1 / similar to Hmgcs1 protein | acetyl-CoA metabolic process / steroid biosynthetic process / cholesterol biosynthetic process / metabolic process / isoprenoid biosynthetic process / lipid biosynthetic process / sterol biosynthetic process | catalytic activity / hydroxymethylglutaryl-CoA synthase activity / hydroxymethylglutaryl-CoA synthase activity / transferase activity | cytoplasm |
| 1433445_x_at | -0.13 | -5.95 | -0.50 | -1.34 | -0.75 | 1.89 | -0.47 | -1.67 | Hmgcs1 / LOC100040592 | 3-hydroxy-3-methylglutaryl-Coenzyme A synthase 1 / similar to Hmgcs1 protein | acetyl-CoA metabolic process / steroid biosynthetic process / cholesterol biosynthetic process / metabolic process / isoprenoid biosynthetic process / lipid biosynthetic process / sterol biosynthetic process | catalytic activity / hydroxymethylglutaryl-CoA synthase activity / hydroxymethylglutaryl-CoA synthase activity / transferase activity | cytoplasm |
| 1433446_at | -0.41 | -3.97 | -0.50 | -2.92 | -0.85 | 0.59 | -0.71 | -0.69 | Hmgcs1 / LOC100040592 | 3-hydroxy-3-methylglutaryl-Coenzyme A synthase 1 / similar to Hmgcs1 protein | acetyl-CoA metabolic process / steroid biosynthetic process / cholesterol biosynthetic process / metabolic process / isoprenoid biosynthetic process / lipid biosynthetic process / sterol biosynthetic process | catalytic activity / hydroxymethylglutaryl-CoA synthase activity / hydroxymethylglutaryl-CoA synthase activity / transferase activity | cytoplasm |
| 1448239_at | 0.75 | -5.45 | 0.64 | -5.53 | 2.56 | 0.63 | 0.75 | -5.10 | Hmox1 | heme oxygenase (decycling) 1 | heme oxidation / response to stimulus | heme oxygenase (decyclizing) activity / iron ion binding / oxidoreductase activity / metal ion binding | endoplasmic reticulum / microsome / integral to membrane |
| 1455120_at | 0.32 | -4.27 | 0.36 | -3.72 | 1.14 | 4.83 | 0.64 | -0.26 | Hpdl | 4-hydroxyphenylpyruvate dioxygenase-like | aromatic amino acid family metabolic process | 4-hydroxyphenylpyruvate dioxygenase activity | --- |
| 1419905_s_at | -1.35 | 0.55 | -0.10 | -6.21 | 0.55 | -4.53 | 0.32 | -5.46 | Hpgd | hydroxyprostaglandin dehydrogenase 15 (NAD) | lipid metabolic process / fatty acid metabolic process / prostaglandin metabolic process / prostaglandin metabolic process / transforming growth factor beta receptor signaling pathway / female pregnancy / parturition / metabolic process / metabolic process / negative regulation of cell cycle | catalytic activity / catalytic activity / catalytic activity / prostaglandin-D synthase activity / prostaglandin E receptor activity / binding / electron carrier activity / 15-hydroxyprostaglandin dehydrogenase (NAD+) activity / 15-hydroxyprostaglandin dehydrogenase (NAD+) activity / 15-hydroxyprostaglandin dehydrogenase (NAD+) activity / oxidoreductase activity / NAD binding | cytoplasm |
| 1425362_at | 0.06 | -6.40 | -0.08 | -6.18 | -0.90 | 0.43 | -0.05 | -6.06 | Hrbl | HIV-1 Rev binding protein-like | regulation of ARF GTPase activity | ARF GTPase activator activity / zinc ion binding / metal ion binding | --- |
| 1428326_s_at | -0.39 | -1.73 | -0.58 | 1.52 | -0.47 | -0.20 | -0.40 | -1.33 | Hrsp12 | heat-responsive protein 12 | --- | nuclease activity / endonuclease activity / hydrolase activity | --- |
| 1423450_a_at | -0.41 | -4.23 | -0.64 | -1.80 | -0.93 | 0.88 | -0.62 | -1.95 | Hs3st1 | heparan sulfate (glucosamine) 3-O-sulfotransferase 1 | --- | sulfotransferase activity / sulfotransferase activity / heparin-glucosamine 3-O-sulfotransferase activity / transferase activity | extracellular space / Golgi apparatus / Golgi lumen |
| 1427126_at | 0.30 | -5.23 | 1.47 | 4.83 | 0.45 | -3.86 | 0.81 | -0.38 | Hspa1b | heat shock protein 1B | telomere maintenance / DNA repair / anti-apoptosis / response to stress / response to heat / negative regulation of caspase activity | nucleotide binding / protein binding / ATP binding | --- |
| 1427127_x_at | 0.11 | -6.42 | 2.44 | 1.00 | 0.54 | -5.66 | 1.49 | -2.42 | Hspa1b | heat shock protein 1B | telomere maintenance / DNA repair / anti-apoptosis / response to stress / response to heat / negative regulation of caspase activity | nucleotide binding / protein binding / ATP binding | --- |
| 1452318_a_at | 0.40 | -5.23 | 1.34 | 1.37 | 0.51 | -4.47 | 0.95 | -1.28 | Hspa1b | heat shock protein 1B | telomere maintenance / DNA repair / anti-apoptosis / response to stress / response to heat / negative regulation of caspase activity | nucleotide binding / protein binding / ATP binding | --- |
| 1443183_at | 0.90 | 0.01 | 0.20 | -5.74 | 0.37 | -4.67 | -0.45 | -3.88 | Huwe1 | HECT, UBA and WWE domain containing 1 | protein polyubiquitination / protein modification process / ubiquitin cycle / histone ubiquitination / mRNA transport | DNA binding / DNA binding / ubiquitin-protein ligase activity / ubiquitin-protein ligase activity / binding / ligase activity | intracellular / nucleus / nucleus / cytoplasm |
| 1460106_at | -0.69 | -1.04 | -0.65 | -1.36 | -0.81 | 0.29 | -0.66 | -1.19 | Hydin | hydrocephalus inducing | multicellular organismal development | --- | --- |
| 1424067_at | 0.79 | 4.57 | -0.08 | -5.98 | -0.22 | -4.37 | 0.00 | -6.10 | Icam1 | intercellular adhesion molecule 1 | cell adhesion / cell adhesion / leukocyte adhesion / cell-cell adhesion / regulation of cell adhesion / cell adhesion mediated by integrin | protein binding | nucleus / external side of plasma membrane / membrane / integral to membrane |
| 1453596_at | -0.44 | -3.49 | -0.03 | -6.27 | -0.83 | 0.84 | -0.26 | -4.94 | Id2 | inhibitor of DNA binding 2 | negative regulation of transcription from RNA polymerase II promoter / multicellular organismal development / heart development / negative regulation of transcription / enucleate erythrocyte differentiation / negative regulation of transcription factor activity / regulation of transcription / positive regulation of macrophage differentiation / lymph node development | protein binding / protein binding / transcription repressor activity / transcription regulator activity | nucleus / nucleus / cytoplasm |
| 1419821_s_at | -0.61 | 1.88 | -0.50 | 0.32 | -0.30 | -3.10 | -0.29 | -3.19 | Idh1 | isocitrate dehydrogenase 1 (NADP+), soluble | glyoxylate cycle / tricarboxylic acid cycle / glutathione metabolic process / response to oxidative stress / metabolic process | magnesium ion binding / isocitrate dehydrogenase (NADP+) activity / isocitrate dehydrogenase (NADP+) activity / oxidoreductase activity / oxidoreductase activity, acting on the CH-OH group of donors, NAD or NADP as acceptor / manganese ion binding / metal ion binding | cytoplasm / cytosol |
| 1423804_a_at | -0.30 | -4.78 | -0.85 | 1.56 | -0.94 | 2.39 | -0.26 | -4.84 | Idi1 | isopentenyl-diphosphate delta isomerase | steroid biosynthetic process / steroid biosynthetic process / cholesterol biosynthetic process / cholesterol biosynthetic process / isoprenoid biosynthetic process / isoprenoid biosynthetic process / lipid biosynthetic process / carotenoid biosynthetic process / sterol biosynthetic process / sterol biosynthetic process | magnesium ion binding / magnesium ion binding / isopentenyl-diphosphate delta-isomerase activity / isopentenyl-diphosphate delta-isomerase activity / hydrolase activity / isomerase activity / isomerase activity / metal ion binding | peroxisome / peroxisome |
| 1451122_at | -0.21 | -5.51 | -0.68 | -0.03 | -0.77 | 0.94 | -0.31 | -4.26 | Idi1 | isopentenyl-diphosphate delta isomerase | steroid biosynthetic process / steroid biosynthetic process / cholesterol biosynthetic process / cholesterol biosynthetic process / isoprenoid biosynthetic process / isoprenoid biosynthetic process / lipid biosynthetic process / carotenoid biosynthetic process / sterol biosynthetic process / sterol biosynthetic process | magnesium ion binding / magnesium ion binding / isopentenyl-diphosphate delta-isomerase activity / isopentenyl-diphosphate delta-isomerase activity / hydrolase activity / isomerase activity / isomerase activity / metal ion binding | peroxisome / peroxisome |
| 1417612_at | 0.71 | -0.81 | 0.78 | -0.07 | 0.95 | 1.54 | 0.33 | -4.45 | Ier5 | immediate early response 5 | --- | --- | --- |
| 1452231_x_at | -0.57 | -2.42 | -0.81 | 0.09 | -0.50 | -3.06 | -0.42 | -3.74 | Ifi203 | interferon activated gene 203 | --- | protein binding | nucleus |
| 1419603_at | -0.61 | -1.50 | -0.87 | 1.37 | -0.64 | -1.01 | -0.34 | -4.19 | Ifi204 | interferon activated gene 204 | transcription / regulation of transcription, DNA-dependent / regulation of transcription from RNA polymerase II promoter / DNA damage response, signal transduction by p53 class mediator resulting in induction of apoptosis / positive regulation of osteoblast differentiation | DNA binding / transcription cofactor activity / protein binding / protein binding / protein binding | nucleus / nucleoplasm / nucleolus / cytoplasm |
| 1452349_x_at | -0.51 | -1.69 | -0.70 | 0.79 | -0.45 | -2.41 | -0.31 | -4.00 | Ifi205 / Mnda | interferon activated gene 205 / myeloid cell nuclear differentiation antigen | transcription / regulation of transcription, DNA-dependent | DNA binding / protein binding | nucleus / cytoplasm |
| 1422476_at | 0.32 | -3.37 | 0.36 | -2.63 | 0.37 | -2.48 | 0.61 | 1.05 | Ifi30 | interferon gamma inducible protein 30 | antigen processing and presentation of exogenous peptide antigen via MHC class II | disulfide oxidoreductase activity | extracellular space / lysosome |
| 1418293_at | -0.33 | -4.27 | 0.16 | -5.68 | 0.69 | 0.11 | 0.75 | 0.81 | Ifit2 | interferon-induced protein with tetratricopeptide repeats 2 | --- | binding | --- |
| 1416067_at | 1.23 | 3.99 | 0.55 | -2.40 | 0.57 | -2.28 | 0.38 | -3.98 | Ifrd1 | interferon-related developmental regulator 1 | multicellular organismal development / adult somatic muscle development / cell differentiation / muscle cell differentiation | binding | nucleus |
| 1418918_at | -0.54 | -2.49 | -0.80 | 0.38 | -1.07 | 2.94 | -0.55 | -2.14 | Igfbp1 | insulin-like growth factor binding protein 1 | regulation of cell growth | insulin-like growth factor binding / insulin-like growth factor binding / growth factor binding | extracellular region / extracellular space / extracellular space |
| 1423062_at | -0.40 | -2.63 | -0.52 | -0.71 | -0.83 | 3.16 | -0.71 | 1.66 | Igfbp3 | insulin-like growth factor binding protein 3 | regulation of cell growth / negative regulation of protein amino acid phosphorylation / protein amino acid phosphorylation / positive regulation of apoptosis / positive regulation of myoblast differentiation | insulin-like growth factor binding / insulin-like growth factor binding / protein tyrosine phosphatase activator activity / growth factor binding | extracellular region / extracellular space / extracellular space / nucleus |
| 1458268_s_at | -0.61 | 0.40 | -0.51 | -0.92 | -1.07 | 5.57 | -0.84 | 3.12 | Igfbp3 | insulin-like growth factor binding protein 3 | regulation of cell growth / negative regulation of protein amino acid phosphorylation / protein amino acid phosphorylation / positive regulation of apoptosis / positive regulation of myoblast differentiation | insulin-like growth factor binding / insulin-like growth factor binding / protein tyrosine phosphatase activator activity / growth factor binding | extracellular region / extracellular space / extracellular space / nucleus |
| 1421016_at | -0.05 | -6.38 | 0.13 | -5.76 | 0.65 | 0.85 | 0.38 | -2.65 | Ighmbp2 | immunoglobulin mu binding protein 2 | transcription / regulation of transcription, DNA-dependent | nucleotide binding / nucleic acid binding / DNA binding / helicase activity / protein binding / ATP binding / zinc ion binding / hydrolase activity / nucleoside-triphosphatase activity / metal ion binding | nucleus / nucleus / cytoplasm / axon / cell soma |
| 1419043_a_at | -0.78 | 0.79 | -0.56 | -1.58 | -0.55 | -1.79 | -0.43 | -3.04 | Iigp1 / LOC100044196 | interferon inducible GTPase 1 / hypothetical protein LOC100044196 | cytokine and chemokine mediated signaling pathway | nucleotide binding / GTPase activity / GTPase activity / GTP binding / GTP binding / hydrolase activity / GDP binding / identical protein binding | nucleus / nuclear envelope / cytoplasm / endoplasmic reticulum / endoplasmic reticulum membrane / Golgi apparatus / membrane |
| 1421208_at | 0.00 | -6.45 | 0.19 | -5.06 | 0.69 | 2.09 | 0.30 | -3.54 | Ikbkg | inhibitor of kappaB kinase gamma | B cell homeostasis / transcription / regulation of transcription, DNA-dependent / activation of NF-kappaB-inducing kinase activity | protein binding / kinase activity | nucleus / cytoplasm / cytoplasm |
| 1421209_s_at | -0.03 | -6.42 | 0.38 | -3.32 | 1.04 | 4.21 | 0.62 | -0.35 | Ikbkg | inhibitor of kappaB kinase gamma | B cell homeostasis / transcription / regulation of transcription, DNA-dependent / activation of NF-kappaB-inducing kinase activity | protein binding / kinase activity | nucleus / cytoplasm / cytoplasm |
| 1435646_at | -0.15 | -5.53 | 0.25 | -4.06 | 0.96 | 5.96 | 0.40 | -1.58 | Ikbkg | inhibitor of kappaB kinase gamma | B cell homeostasis / transcription / regulation of transcription, DNA-dependent / activation of NF-kappaB-inducing kinase activity | protein binding / kinase activity | nucleus / cytoplasm / cytoplasm |
| 1435647_at | -0.15 | -5.96 | 0.47 | -2.62 | 0.96 | 2.86 | 0.39 | -3.40 | Ikbkg | inhibitor of kappaB kinase gamma | B cell homeostasis / transcription / regulation of transcription, DNA-dependent / activation of NF-kappaB-inducing kinase activity | protein binding / kinase activity | nucleus / cytoplasm / cytoplasm |
| 1454690_at | 0.02 | -6.42 | 0.41 | -1.32 | 0.65 | 2.39 | 0.25 | -3.86 | Ikbkg | inhibitor of kappaB kinase gamma | B cell homeostasis / transcription / regulation of transcription, DNA-dependent / activation of NF-kappaB-inducing kinase activity | protein binding / kinase activity | nucleus / cytoplasm / cytoplasm |
| 1437542_at | -0.75 | -0.97 | -0.31 | -4.97 | -0.76 | -0.84 | -0.88 | 0.30 | Ikzf2 | IKAROS family zinc finger 2 | transcription / regulation of transcription, DNA-dependent | nucleic acid binding / DNA binding / transcription factor activity / zinc ion binding / metal ion binding | intracellular / nucleus / transcription factor complex |
| 1427164_at | -0.06 | -6.38 | -0.72 | -0.15 | -0.97 | 2.33 | -0.68 | -0.54 | Il13ra1 | interleukin 13 receptor, alpha 1 | cell surface receptor linked signal transduction | receptor activity / hematopoietin/interferon-class (D200-domain) cytokine receptor activity / interleukin receptor activity | extracellular space / integral to plasma membrane / membrane / integral to membrane / integral to membrane |
| 1427165_at | -0.41 | -3.02 | -0.65 | 0.21 | -0.80 | 2.02 | -0.67 | 0.44 | Il13ra1 | interleukin 13 receptor, alpha 1 | cell surface receptor linked signal transduction | receptor activity / hematopoietin/interferon-class (D200-domain) cytokine receptor activity / interleukin receptor activity | extracellular space / integral to plasma membrane / membrane / integral to membrane / integral to membrane |
| 1454783_at | -0.34 | -5.65 | -1.07 | -1.08 | -1.30 | 0.42 | -0.83 | -2.55 | Il13ra1 | interleukin 13 receptor, alpha 1 | cell surface receptor linked signal transduction | receptor activity / hematopoietin/interferon-class (D200-domain) cytokine receptor activity / interleukin receptor activity | extracellular space / integral to plasma membrane / membrane / integral to membrane / integral to membrane |
| 1448950_at | 0.17 | -5.88 | -0.31 | -4.55 | -0.79 | 0.75 | -0.27 | -4.72 | Il1r1 | interleukin 1 receptor, type I | signal transduction / cytokine and chemokine mediated signaling pathway / innate immune response | signal transducer activity / receptor activity / transmembrane receptor activity / interleukin-1 receptor activity / interleukin-1 receptor activity / interleukin-1, Type I, activating receptor activity / protein binding | extracellular space / integral to plasma membrane / membrane / integral to membrane / integral to membrane / intrinsic to membrane |
| 1425663_at | 0.97 | 6.56 | 0.76 | 3.95 | 0.53 | 0.72 | 0.09 | -5.76 | Il1rn | interleukin 1 receptor antagonist | lipid metabolic process / inflammatory response / immune response / cell surface receptor linked signal transduction / insulin secretion | receptor activity / interleukin-1 receptor binding / interleukin-1 receptor antagonist activity | extracellular region / cytoplasm / integral to plasma membrane |
| 1451798_at | 2.33 | 5.85 | 1.62 | 2.25 | 1.77 | 3.06 | 0.59 | -4.09 | Il1rn | interleukin 1 receptor antagonist | lipid metabolic process / inflammatory response / immune response / cell surface receptor linked signal transduction / insulin secretion | receptor activity / interleukin-1 receptor binding / interleukin-1 receptor antagonist activity | extracellular region / cytoplasm / integral to plasma membrane |
| 1436861_at | -0.87 | 3.19 | -0.09 | -6.07 | 0.40 | -2.76 | -0.11 | -5.76 | Il7 | interleukin 7 | T cell lineage commitment / anti-apoptosis / immune response / regulation of gene expression / regulation of gene expression / negative regulation of cell growth / positive regulation of B cell proliferation / negative regulation of apoptosis / negative regulation of catalytic activity / bone resorption / positive regulation of T cell differentiation / positive regulation of organ growth / positive regulation of organ growth / homeostasis of number of cells within a tissue | cytokine activity / cytokine activity / hematopoietin/interferon-class (D200-domain) cytokine receptor binding / interleukin-7 receptor binding / growth factor activity / growth factor activity | extracellular region / extracellular space / extracellular space |
| 1415911_at | 0.43 | -1.94 | 0.36 | -2.82 | 0.59 | 0.49 | 0.41 | -2.05 | Impact | imprinted and ancient | negative regulation of protein amino acid phosphorylation / regulation of translational initiation | protein binding | --- |
| 1442073_at | -0.64 | 1.62 | -0.54 | 0.20 | -0.70 | 2.43 | -0.45 | -1.17 | Inpp1 | inositol polyphosphate-1-phosphatase | --- | magnesium ion binding / inositol or phosphatidylinositol phosphatase activity / inositol-1,4-bisphosphate 1-phosphatase activity / hydrolase activity / lithium ion binding / metal ion binding | --- |
| 1456259_at | -0.61 | 0.16 | -0.43 | -2.28 | -0.05 | -6.22 | -0.20 | -5.02 | Ints10 | integrator complex subunit 10 | --- | --- | nucleus |
| 1453312_at | -0.37 | -3.22 | -0.73 | 1.70 | -0.16 | -5.59 | -0.34 | -3.30 | Iqwd1 | IQ motif and WD repeats 1 | --- | DNA binding | nucleus / nucleus |
| 1418825_at | -0.85 | 0.52 | -0.34 | -4.52 | -0.06 | -6.24 | -0.42 | -3.65 | Irgm | immunity-related GTPase family, M | autophagy / defense response / immune response / innate immune response | nucleotide binding / GTP binding / hydrolase activity | Golgi membrane / cytoplasm / endoplasmic reticulum / Golgi apparatus / plasma membrane / membrane / cytoplasmic vesicle |
| 1423104_at | -0.83 | 4.60 | -0.40 | -1.69 | -0.23 | -4.39 | -0.31 | -2.92 | Irs1 | insulin receptor substrate 1 | positive regulation of mesenchymal cell proliferation / signal transduction / insulin receptor signaling pathway / insulin receptor signaling pathway / insulin secretion / positive regulation of cell migration / mammary gland development / response to peptide hormone stimulus / protein kinase B signaling cascade / positive regulation of phosphoinositide 3-kinase activity / insulin-like growth factor receptor signaling pathway / insulin-like growth factor receptor signaling pathway | signal transducer activity / receptor activity / insulin receptor binding / insulin receptor binding / insulin-like growth factor receptor binding / insulin-like growth factor receptor binding / protein binding / protein binding / protein kinase binding / SH2 domain binding / SH2 domain binding / phosphoinositide 3-kinase binding / phosphoinositide 3-kinase binding | nucleus / cytoplasm / microsome |
| 1423957_at | 0.64 | 0.27 | 0.38 | -3.15 | 0.75 | 1.67 | 0.22 | -4.88 | Isg20l1 | interferon stimulated exonuclease gene 20-like 1 | apoptosis / response to DNA damage stimulus | nucleic acid binding / nuclease activity / exonuclease activity / hydrolase activity | intracellular / nucleus |
| 1450506_a_at | 0.28 | -5.27 | 0.25 | -5.31 | 0.91 | 1.07 | 0.23 | -5.29 | Isg20l1 | interferon stimulated exonuclease gene 20-like 1 | apoptosis / response to DNA damage stimulus | nucleic acid binding / nuclease activity / exonuclease activity / hydrolase activity | intracellular / nucleus |
| 1451186_at | 0.64 | 2.30 | 0.35 | -2.25 | 0.36 | -2.23 | 0.30 | -3.01 | Isg20l1 | interferon stimulated exonuclease gene 20-like 1 | apoptosis / response to DNA damage stimulus | nucleic acid binding / nuclease activity / exonuclease activity / hydrolase activity | intracellular / nucleus |
| 1435637_at | -0.60 | 1.63 | -0.51 | 0.31 | -0.35 | -2.32 | -0.09 | -5.73 | Itfg1 | integrin alpha FG-GAP repeat containing 1 | --- | protein binding | extracellular region / membrane / integral to membrane / integral to membrane |
| 1422157_a_at | 0.18 | -5.54 | 0.47 | -1.84 | 0.71 | 1.28 | 0.77 | 1.91 | Itgb1bp1 | integrin beta 1 binding protein 1 | cell-matrix adhesion / integrin-mediated signaling pathway / regulation of cell adhesion | integrin binding / protein binding / protein C-terminus binding | ruffle / cytosol / lamellipodium |
| 1422983_at | 1.06 | 0.94 | 0.89 | -0.43 | 0.39 | -4.68 | 0.50 | -3.68 | Itgb6 | integrin beta 6 | inflammatory response / cell adhesion / cell-matrix adhesion / integrin-mediated signaling pathway / multicellular organismal development | receptor activity / integrin binding / binding / protein binding | integrin complex / membrane / integral to membrane |
| 1417618_at | -0.36 | -4.42 | -0.45 | -3.48 | -0.99 | 2.02 | -0.82 | 0.37 | Itih2 | inter-alpha trypsin inhibitor, heavy chain 2 | hyaluronan metabolic process | endopeptidase inhibitor activity / serine-type endopeptidase inhibitor activity | extracellular region / extracellular space |
| 1429159_at | -0.42 | -4.41 | -0.66 | -2.13 | -1.24 | 2.86 | -0.68 | -1.80 | Itih5 | inter-alpha (globulin) inhibitor H5 | hyaluronan metabolic process | serine-type endopeptidase inhibitor activity | extracellular region / extracellular space |
| 1420961_a_at | 0.76 | 0.39 | -0.08 | -6.16 | -0.13 | -5.95 | 0.10 | -5.91 | Ivns1abp | influenza virus NS1A binding protein | --- | protein binding | nucleus / cytoplasm / cytoskeleton |
| 1421065_at | -0.50 | -2.96 | -0.68 | -0.98 | -0.95 | 1.73 | -0.65 | -1.19 | Jak2 | Janus kinase 2 | protein amino acid phosphorylation / apoptosis / apoptosis / enzyme linked receptor protein signaling pathway / protein kinase cascade / JAK-STAT cascade / JAK-STAT cascade / tyrosine phosphorylation of STAT protein / tyrosine phosphorylation of STAT protein / STAT protein nuclear translocation / negative regulation of cell proliferation / peptidyl-tyrosine phosphorylation / cytokine and chemokine mediated signaling pathway / myeloid cell differentiation / cell differentiation / negative regulation of DNA binding | nucleotide binding / protein kinase activity / protein tyrosine kinase activity / protein tyrosine kinase activity / non-membrane spanning protein tyrosine kinase activity / Janus kinase activity / Janus kinase activity / Janus kinase activity / receptor binding / protein binding / protein binding / ATP binding / kinase activity / transferase activity / SH2 domain binding | cytoplasm / cytoskeleton / caveola / membrane / membrane raft |
| 1421066_at | -0.36 | -3.15 | -0.56 | -0.20 | -0.78 | 2.63 | -0.54 | -0.41 | Jak2 | Janus kinase 2 | protein amino acid phosphorylation / apoptosis / apoptosis / enzyme linked receptor protein signaling pathway / protein kinase cascade / JAK-STAT cascade / JAK-STAT cascade / tyrosine phosphorylation of STAT protein / tyrosine phosphorylation of STAT protein / STAT protein nuclear translocation / negative regulation of cell proliferation / peptidyl-tyrosine phosphorylation / cytokine and chemokine mediated signaling pathway / myeloid cell differentiation / cell differentiation / negative regulation of DNA binding | nucleotide binding / protein kinase activity / protein tyrosine kinase activity / protein tyrosine kinase activity / non-membrane spanning protein tyrosine kinase activity / Janus kinase activity / Janus kinase activity / Janus kinase activity / receptor binding / protein binding / protein binding / ATP binding / kinase activity / transferase activity / SH2 domain binding | cytoplasm / cytoskeleton / caveola / membrane / membrane raft |
| 1449117_at | 0.18 | -5.22 | 0.31 | -3.28 | 0.61 | 1.30 | 0.40 | -1.75 | Jund | Jun proto-oncogene related gene d | osteoblast development / transcription / regulation of transcription, DNA-dependent / cellular process / regulation of transcription / positive regulation of osteoblast differentiation / regulation of cell cycle | DNA binding / DNA binding / transcription factor activity / sequence-specific DNA binding / protein dimerization activity | intracellular / nucleus |
| 1433902_at | 0.18 | -5.91 | 0.28 | -5.00 | 0.80 | 0.14 | 0.18 | -5.54 | Kbtbd8 | kelch repeat and BTB (POZ) domain containing 8 | --- | protein binding | --- |
| 1449158_at | -0.64 | -3.45 | -0.81 | -2.08 | -1.30 | 1.43 | -0.97 | -0.81 | Kcnk2 | potassium channel, subfamily K, member 2 | transport / ion transport / potassium ion transport / potassium ion transport / G-protein coupled receptor protein signaling pathway / stabilization of membrane potential | ion channel activity / voltage-gated ion channel activity / voltage-gated potassium channel activity / potassium channel activity / potassium ion binding | integral to plasma membrane / voltage-gated potassium channel complex / membrane / integral to membrane / integral to membrane |
| 1421852_at | -0.79 | 1.46 | -0.69 | 0.31 | -0.75 | 0.99 | -0.35 | -3.62 | Kcnk5 | potassium channel, subfamily K, member 5 | transport / ion transport / potassium ion transport | ion channel activity / potassium channel activity | integral to plasma membrane / membrane / membrane / integral to membrane |
| 1434881_s_at | -0.04 | -6.38 | -0.85 | 4.85 | -0.95 | 6.02 | -1.02 | 6.22 | Kctd12 | potassium channel tetramerisation domain containing 12 | potassium ion transport | voltage-gated potassium channel activity / protein binding | voltage-gated potassium channel complex / membrane |
| 1435339_at | 0.49 | -1.16 | 0.70 | 1.83 | 0.66 | 1.32 | 0.23 | -4.55 | Kctd15 | potassium channel tetramerisation domain containing 15 | potassium ion transport | ion channel activity / voltage-gated potassium channel activity / protein binding | voltage-gated potassium channel complex / membrane |
| 1416324_s_at | 0.12 | -5.91 | 0.11 | -5.81 | 0.77 | 3.51 | 0.23 | -4.29 | Kctd20 | potassium channel tetramerisation domain containing 20 | potassium ion transport / potassium ion transport | voltage-gated potassium channel activity / voltage-gated potassium channel activity / protein binding / protein binding / identical protein binding | voltage-gated potassium channel complex / voltage-gated potassium channel complex / membrane / membrane |
| 1453317_a_at | -0.93 | 4.55 | -0.62 | 0.86 | -0.14 | -5.65 | -0.34 | -3.15 | Khdrbs3 | KH domain containing, RNA binding, signal transduction associated 3 | transcription / regulation of transcription, DNA-dependent | RNA binding / protein binding | nucleus |
| 1429062_at | -0.82 | 4.13 | -0.23 | -4.39 | 0.23 | -4.45 | -0.22 | -4.38 | Kif16b | kinesin family member 16B | microtubule-based process / microtubule-based movement / cell communication | nucleotide binding / motor activity / motor activity / microtubule motor activity / protein binding / ATP binding / ATP binding / phosphoinositide binding | kinesin complex / microtubule / microtubule associated complex / microtubule associated complex |
| 1429063_s_at | -0.69 | 0.02 | -0.34 | -4.08 | 0.17 | -5.67 | -0.11 | -5.82 | Kif16b | kinesin family member 16B | microtubule-based process / microtubule-based movement / cell communication | nucleotide binding / motor activity / motor activity / microtubule motor activity / protein binding / ATP binding / ATP binding / phosphoinositide binding | kinesin complex / microtubule / microtubule associated complex / microtubule associated complex |
| 1450074_at | 0.00 | -6.45 | 0.17 | -5.38 | 0.66 | 1.30 | 0.14 | -5.48 | Kif3b | kinesin family member 3B | microtubule-based process / microtubule-based movement | nucleotide binding / motor activity / microtubule motor activity / protein binding / ATP binding | kinesin complex / microtubule / microtubule associated complex |
| 1422945_a_at | -0.60 | -1.41 | -0.99 | 2.70 | -0.73 | 0.09 | -0.68 | -0.37 | Kif5c | kinesin family member 5C | microtubule-based process / microtubule-based movement / motor axon guidance | nucleotide binding / motor activity / microtubule motor activity / microtubule motor activity / protein binding / ATP binding | cytoplasm / kinesin complex / kinesin complex / microtubule / microtubule associated complex / ciliary rootlet / neuron projection |
| 1455266_at | -0.69 | 1.38 | -0.89 | 3.63 | -0.83 | 3.00 | -0.76 | 2.16 | Kif5c | kinesin family member 5C | microtubule-based process / microtubule-based movement / motor axon guidance | nucleotide binding / motor activity / microtubule motor activity / microtubule motor activity / protein binding / ATP binding | cytoplasm / kinesin complex / kinesin complex / microtubule / microtubule associated complex / ciliary rootlet / neuron projection |
| 1415854_at | 0.54 | -4.48 | 0.70 | -3.29 | 1.33 | 1.01 | 0.75 | -2.82 | Kitl | kit ligand | neural crest cell migration / positive regulation of myeloid leukocyte differentiation / cell adhesion / germ cell development / positive regulation of cell proliferation / positive regulation of cell proliferation / negative regulation of apoptosis / positive regulation of MAP kinase activity / positive regulation of Ras protein signal transduction / positive regulation of peptidyl-tyrosine phosphorylation | stem cell factor receptor binding / stem cell factor receptor binding / protein binding / protein binding / growth factor activity | extracellular region / extracellular space / plasma membrane / plasma membrane / membrane / integral to membrane / integral to membrane |
| 1437241_at | 0.56 | -2.21 | 0.29 | -4.83 | 0.78 | 0.17 | 0.43 | -3.33 | Klf11 | Kruppel-like factor 11 | transcription / regulation of transcription, DNA-dependent / hemopoiesis / negative regulation of transcription, DNA-dependent | nucleic acid binding / DNA binding / DNA binding / transcription factor activity / zinc ion binding / metal ion binding | intracellular / nucleus / nucleus |
| 1417394_at | 0.72 | 2.24 | 0.59 | 0.61 | 0.79 | 3.13 | 0.92 | 4.29 | Klf4 | Kruppel-like factor 4 (gut) | transcription / regulation of transcription, DNA-dependent / negative regulation of cell proliferation / epidermal cell differentiation / stem cell maintenance / cell differentiation / post-embryonic camera-type eye development / negative regulation of transcription, DNA-dependent / negative regulation of transcription, DNA-dependent / positive regulation of transcription from RNA polymerase II promoter / epidermis morphogenesis | nucleic acid binding / DNA binding / DNA binding / transcription factor activity / zinc ion binding / transcription repressor activity / transcription repressor activity / sequence-specific DNA binding / metal ion binding | intracellular / nucleus / nucleus |
| 1417395_at | 0.51 | -0.86 | 0.75 | 2.27 | 0.80 | 2.91 | 0.52 | -0.60 | Klf4 | Kruppel-like factor 4 (gut) | transcription / regulation of transcription, DNA-dependent / negative regulation of cell proliferation / epidermal cell differentiation / stem cell maintenance / cell differentiation / post-embryonic camera-type eye development / negative regulation of transcription, DNA-dependent / negative regulation of transcription, DNA-dependent / positive regulation of transcription from RNA polymerase II promoter / epidermis morphogenesis | nucleic acid binding / DNA binding / DNA binding / transcription factor activity / zinc ion binding / transcription repressor activity / transcription repressor activity / sequence-specific DNA binding / metal ion binding | intracellular / nucleus / nucleus |
| 1447448_s_at | 0.80 | 1.32 | 0.63 | -0.52 | 0.67 | -0.10 | 0.52 | -1.72 | Klf6 | Kruppel-like factor 6 | transcription / regulation of transcription, DNA-dependent / cytokine and chemokine mediated signaling pathway | nucleic acid binding / DNA binding / DNA binding / zinc ion binding / metal ion binding | intracellular / nucleus / nucleus / cytoplasm |
| 1428081_at | 0.67 | 3.99 | 0.41 | -0.47 | 0.22 | -3.95 | 0.14 | -5.13 | Klhl21 | kelch-like 21 (Drosophila) | --- | protein binding | --- |
| 1435743_at | -0.66 | 3.45 | -0.36 | -1.46 | -0.04 | -6.21 | 0.03 | -6.04 | Klhl23 | kelch-like 23 (Drosophila) | --- | protein binding | --- |
| 1424096_at | -0.24 | -4.95 | 0.20 | -5.27 | 0.64 | 0.38 | 0.51 | -1.32 | Krt5 | keratin 5 | cell morphogenesis / cytoskeleton organization and biogenesis / epidermis development | structural molecule activity / structural constituent of cytoskeleton / protein binding | intermediate filament |
| 1424114_s_at | -0.25 | -4.57 | -0.52 | -0.51 | -0.74 | 2.41 | -0.80 | 3.00 | Lamb1-1 | laminin B1 subunit 1 | cell adhesion / negative regulation of cell adhesion / embryo implantation / learning and/or memory / positive regulation of cell migration / neurite development / odontogenesis | extracellular matrix structural constituent / extracellular matrix structural constituent / protein binding / protein binding / enzyme binding | extracellular region / proteinaceous extracellular matrix / proteinaceous extracellular matrix / basement membrane / basement membrane / basement membrane / basal lamina / laminin-1 complex / laminin-2 complex / laminin-8 complex / laminin-10 complex / laminin-10 complex / perinuclear region of cytoplasm |
| 1421279_at | 0.52 | -2.53 | 0.79 | 0.54 | 1.12 | 3.67 | 0.58 | -1.68 | Lamc2 | laminin, gamma 2 | cell adhesion | extracellular matrix structural constituent / protein binding / protein binding / heparin binding / heparin binding | extracellular region / proteinaceous extracellular matrix / basement membrane / basement membrane / basal lamina / extracellular space / extracellular matrix |
| 1420550_at | 0.35 | -3.22 | 0.58 | 0.17 | 0.23 | -4.78 | 0.38 | -2.65 | Lce1f | late cornified envelope 1F | --- | protein binding | --- |
| 1456712_at | -0.08 | -6.34 | -0.83 | 0.30 | -0.60 | -2.06 | -0.46 | -3.34 | Lcorl | ligand dependent nuclear receptor corepressor-like | transcription / regulation of transcription, DNA-dependent / transcription from RNA polymerase II promoter | DNA binding / RNA polymerase II transcription factor activity | nucleus / nucleus |
| 1421821_at | -0.63 | -2.73 | -1.02 | 0.78 | -0.94 | 0.07 | -0.42 | -4.21 | Ldlr | low density lipoprotein receptor | lipid metabolic process / lipid metabolic process / transport / lipid transport / endocytosis / steroid metabolic process / cholesterol metabolic process / cholesterol metabolic process / cholesterol transport / lipoprotein metabolic process / lipoprotein catabolic process / lipoprotein catabolic process / cholesterol homeostasis / cholesterol homeostasis | receptor activity / low-density lipoprotein receptor activity / low-density lipoprotein receptor activity / lipid transporter activity / calcium ion binding / protein binding / low-density lipoprotein binding / very-low-density lipoprotein receptor activity | extracellular space / endosome / coated pit / membrane / integral to membrane / integral to membrane |
| 1431947_at | -0.48 | -3.30 | -0.48 | -3.19 | -0.80 | 0.06 | -0.19 | -5.50 | Ldlr | low density lipoprotein receptor | lipid metabolic process / lipid metabolic process / transport / lipid transport / endocytosis / steroid metabolic process / cholesterol metabolic process / cholesterol metabolic process / cholesterol transport / lipoprotein metabolic process / lipoprotein catabolic process / lipoprotein catabolic process / cholesterol homeostasis / cholesterol homeostasis | receptor activity / low-density lipoprotein receptor activity / low-density lipoprotein receptor activity / lipid transporter activity / calcium ion binding / protein binding / low-density lipoprotein binding / very-low-density lipoprotein receptor activity | extracellular space / endosome / coated pit / membrane / integral to membrane / integral to membrane |
| 1459403_at | -0.90 | 0.44 | -0.89 | 0.37 | -1.03 | 1.62 | -0.27 | -5.09 | Ldlr | low density lipoprotein receptor | lipid metabolic process / lipid metabolic process / transport / lipid transport / endocytosis / steroid metabolic process / cholesterol metabolic process / cholesterol metabolic process / cholesterol transport / lipoprotein metabolic process / lipoprotein catabolic process / lipoprotein catabolic process / cholesterol homeostasis / cholesterol homeostasis | receptor activity / low-density lipoprotein receptor activity / low-density lipoprotein receptor activity / lipid transporter activity / calcium ion binding / protein binding / low-density lipoprotein binding / very-low-density lipoprotein receptor activity | extracellular space / endosome / coated pit / membrane / integral to membrane / integral to membrane |
| 1450988_at | -0.62 | 1.87 | -0.67 | 2.48 | -0.49 | -0.11 | -0.33 | -2.63 | Lgr5 | leucine rich repeat containing G protein coupled receptor 5 | signal transduction / G-protein coupled receptor protein signaling pathway | rhodopsin-like receptor activity / signal transducer activity / receptor activity / G-protein coupled receptor activity / protein binding / protein-hormone receptor activity | extracellular space / plasma membrane / membrane / integral to membrane / integral to membrane |
| 1434129_s_at | 1.33 | 1.67 | 0.47 | -4.55 | 0.29 | -5.59 | 0.14 | -5.93 | Lhfpl2 | lipoma HMGIC fusion partner-like 2 | metabolic process | transferase activity | membrane / integral to membrane / integral to membrane |
| 1434130_at | 0.59 | 1.58 | 0.14 | -5.45 | 0.06 | -6.14 | 0.06 | -5.91 | Lhfpl2 | lipoma HMGIC fusion partner-like 2 | metabolic process | transferase activity | membrane / integral to membrane / integral to membrane |
| 1421207_at | 1.05 | 3.27 | 0.53 | -2.19 | 0.21 | -5.43 | 0.24 | -4.99 | Lif | leukemia inhibitory factor | immune response / embryo implantation / embryo implantation / positive regulation of cell proliferation / positive regulation of cell proliferation / stem cell maintenance / stem cell maintenance / positive regulation of peptidyl-serine phosphorylation / tyrosine phosphorylation of Stat3 protein / positive regulation of MAPKKK cascade / positive regulation of macrophage differentiation / negative regulation of meiosis / positive regulation of transcription from RNA polymerase II promoter / decidualization / decidualization / negative regulation of hormone secretion / muscle morphogenesis / neuron development / positive regulation of peptidyl-tyrosine phosphorylation / maternal process involved in pregnancy | cytokine activity / cytokine activity / cytokine activity / leukemia inhibitory factor receptor binding / leukemia inhibitory factor receptor binding / growth factor activity / growth factor activity / growth factor activity | extracellular region / extracellular space / extracellular space |
| 1454984_at | -0.52 | -2.78 | -0.68 | -0.87 | -0.81 | 0.43 | -0.61 | -1.56 | Lifr | leukemia inhibitory factor receptor | positive regulation of cell proliferation / positive regulation of cell proliferation / cytokine and chemokine mediated signaling pathway | receptor activity / hematopoietin/interferon-class (D200-domain) cytokine receptor activity / leukemia inhibitory factor receptor activity / protein binding | extracellular region / plasma membrane / membrane / integral to membrane / integral to membrane |
| 1424408_at | 0.48 | -3.13 | 0.91 | 1.46 | 0.37 | -4.20 | 0.57 | -1.89 | Lims2 | LIM and senescent cell antigen like domains 2 | --- | zinc ion binding / metal ion binding | focal adhesion / cell junction |
| 1451849_a_at | -0.09 | -6.22 | -0.21 | -5.19 | 0.63 | 0.04 | -0.03 | -6.07 | Lmnb2 | lamin B2 | --- | structural molecule activity | nucleus / nuclear inner membrane / lamin filament / intermediate filament / membrane |
| 1418478_at | 0.04 | -6.39 | 0.71 | 1.80 | 0.04 | -6.26 | 0.01 | -6.09 | Lmo1 | LIM domain only 1 | multicellular organismal development / cell proliferation / cellular process | transcription factor activity / zinc ion binding / metal ion binding | nucleus / nucleus / nucleolus |
| 1436040_at | 1.07 | 0.72 | 1.02 | 0.33 | 1.09 | 0.87 | 0.54 | -3.48 | LOC100039864 | Hypothetical protein LOC100039864 | --- | --- | --- |
| 1458656_at | -0.26 | -5.02 | -0.40 | -3.39 | -0.69 | 0.09 | -0.05 | -6.03 | LOC100040174 / LOC100047186 | hypothetical protein LOC100040174 / hypothetical protein LOC100047186 | --- | --- | --- |
| 1431213_a_at | -0.43 | -5.83 | -0.67 | -4.87 | -1.81 | 0.30 | -1.15 | -2.67 | LOC433762 | hypothetical gene LOC433762 | DNA integration | nucleic acid binding / DNA binding / RNA-directed DNA polymerase activity / nuclease activity / endonuclease activity / ribonuclease H activity / structural molecule activity / transferase activity / nucleotidyltransferase activity / hydrolase activity / metal ion binding | viral capsid |
| 1446594_at | -0.75 | 2.01 | -0.03 | -6.26 | -0.05 | -6.23 | -0.08 | -5.92 | LOC676777 | similar to Eukaryotic translation initiation factor 3 subunit 2 (eIF-3 beta) (eIF3 p36) (eIF3i) (TGF-beta receptor-interacting protein 1) (TRIP-1) | --- | --- | --- |
| 1460178_at | -0.64 | 0.89 | -0.09 | -6.05 | -0.17 | -5.41 | -0.13 | -5.60 | Lonp2 | lon peptidase 2, peroxisomal | proteolysis / proteolysis / ATP-dependent proteolysis / peroxisome organization and biogenesis | nucleotide binding / ATP-dependent peptidase activity / serine-type endopeptidase activity / ATP binding / peptidase activity / hydrolase activity / nucleoside-triphosphatase activity | mitochondrion / peroxisome / peroxisomal matrix / peroxisomal matrix |
| 1455665_at | 0.67 | 2.11 | 0.74 | 3.06 | 0.42 | -1.55 | 0.49 | -0.42 | Lonrf1 / LOC631639 | LON peptidase N-terminal domain and ring finger 1 / similar to CG32369-PB, isoform B | ATP-dependent proteolysis / protein ubiquitination | ATP-dependent peptidase activity / ubiquitin-protein ligase activity / protein binding / zinc ion binding | ubiquitin ligase complex |
| 1429863_at | 0.53 | -0.16 | 0.89 | 4.50 | 0.02 | -6.30 | 0.16 | -5.26 | Lonrf3 | LON peptidase N-terminal domain and ring finger 3 | ATP-dependent proteolysis | ATP-dependent peptidase activity / binding / protein binding / zinc ion binding / metal ion binding | --- |
| 1434111_at | -0.15 | -6.03 | -0.55 | -2.31 | -0.80 | 0.27 | -0.65 | -1.22 | Lphn2 / LOC100048050 | latrophilin 2 / similar to calcium-independent alpha-latrotoxin receptor homolog 2 | signal transduction / G-protein coupled receptor protein signaling pathway / G-protein coupled receptor protein signaling pathway / neuropeptide signaling pathway | signal transducer activity / receptor activity / G-protein coupled receptor activity / G-protein coupled receptor activity / sugar binding / latrotoxin receptor activity / latrotoxin receptor activity | plasma membrane / membrane / membrane / integral to membrane / integral to membrane |
| 1434210_s_at | 0.71 | 3.97 | 0.10 | -5.79 | -0.08 | -5.98 | -0.21 | -4.15 | Lrig1 | leucine-rich repeats and immunoglobulin-like domains 1 | --- | protein binding | extracellular space / membrane / integral to membrane / integral to membrane |
| 1449893_a_at | 0.67 | 2.06 | -0.05 | -6.19 | -0.05 | -6.20 | -0.15 | -5.30 | Lrig1 | leucine-rich repeats and immunoglobulin-like domains 1 | --- | protein binding | extracellular space / membrane / integral to membrane / integral to membrane |
| 1438422_at | 0.13 | -5.98 | 0.31 | -3.94 | 0.62 | 0.13 | 0.20 | -5.11 | Lrrc20 | leucine rich repeat containing 20 | --- | protein binding | --- |
| 1433858_at | -0.13 | -6.14 | 0.02 | -6.27 | 1.10 | 2.89 | 0.59 | -1.94 | Lrrc28 | leucine rich repeat containing 28 | --- | protein binding | --- |
| 1437201_at | -0.92 | 2.72 | -0.53 | -1.73 | -0.33 | -4.16 | -0.46 | -2.49 | Lrrc4c | leucine rich repeat containing 4C | regulation of axonogenesis / regulation of axonogenesis | protein binding / protein binding / protein binding | membrane / membrane / integral to membrane |
| 1433505_a_at | 0.88 | 6.61 | 0.43 | -0.16 | 0.50 | 1.14 | 0.61 | 2.85 | Lrrc8d | leucine rich repeat containing 8D | --- | protein binding | membrane / integral to membrane / integral to membrane |
| 1437833_at | -0.38 | -5.56 | -0.61 | -4.25 | -1.37 | 0.46 | -0.53 | -4.53 | Ltbp3 | latent transforming growth factor beta binding protein 3 | skeletal development / transforming growth factor beta receptor signaling pathway / transforming growth factor beta receptor signaling pathway | binding / calcium ion binding / growth factor binding | extracellular region / extracellular region / proteinaceous extracellular matrix / extracellular space |
| 1428974_s_at | -0.09 | -6.24 | -0.33 | -3.96 | 0.68 | 0.25 | 0.00 | -6.10 | Lztfl1 | leucine zipper transcription factor-like 1 | --- | --- | --- |
| 1435828_at | -1.35 | 4.17 | -0.86 | 0.08 | -0.24 | -5.52 | -0.45 | -3.67 | Maf | avian musculoaponeurotic fibrosarcoma (v-maf) AS42 oncogene homolog | cytokine production / transcription / regulation of transcription, DNA-dependent / cellular process / regulation of chondrocyte differentiation / regulation of transcription / regulation of transcription / positive regulation of transcription from RNA polymerase II promoter / regulation of cell cycle | DNA binding / DNA binding / transcription factor activity / protein binding / sequence-specific DNA binding / protein dimerization activity | nucleus / nucleus / cytoplasm |
| 1447849_s_at | -1.61 | 7.12 | -1.22 | 4.23 | -0.92 | 1.63 | -1.01 | 2.39 | Maf | avian musculoaponeurotic fibrosarcoma (v-maf) AS42 oncogene homolog | cytokine production / transcription / regulation of transcription, DNA-dependent / cellular process / regulation of chondrocyte differentiation / regulation of transcription / regulation of transcription / positive regulation of transcription from RNA polymerase II promoter / regulation of cell cycle | DNA binding / DNA binding / transcription factor activity / protein binding / sequence-specific DNA binding / protein dimerization activity | nucleus / nucleus / cytoplasm |
| 1456060_at | -1.50 | 8.38 | -1.26 | 6.44 | -1.07 | 4.87 | -0.98 | 3.89 | Maf | avian musculoaponeurotic fibrosarcoma (v-maf) AS42 oncogene homolog | cytokine production / transcription / regulation of transcription, DNA-dependent / cellular process / regulation of chondrocyte differentiation / regulation of transcription / regulation of transcription / positive regulation of transcription from RNA polymerase II promoter / regulation of cell cycle | DNA binding / DNA binding / transcription factor activity / protein binding / sequence-specific DNA binding / protein dimerization activity | nucleus / nucleus / cytoplasm |
| 1418616_at | 0.85 | -0.54 | 0.53 | -3.36 | 1.00 | 0.80 | 0.54 | -3.09 | Mafk | v-maf musculoaponeurotic fibrosarcoma oncogene family, protein K (avian) | transcription / regulation of transcription, DNA-dependent / regulation of transcription, DNA-dependent / nervous system development / cellular process / regulation of transcription / regulation of cell cycle | DNA binding / DNA binding / transcription factor activity / transcription factor activity / protein binding / sequence-specific DNA binding / protein dimerization activity | intracellular / nucleus / nucleus |
| 1427042_at | -0.51 | 0.37 | -1.07 | 7.10 | -0.57 | 1.26 | -0.43 | -0.97 | Mal2 | mal, T-cell differentiation protein 2 | --- | protein binding | plasma membrane / membrane / integral to membrane / apical plasma membrane |
| 1417110_at | 1.12 | 4.62 | 0.52 | -1.83 | -0.19 | -5.46 | -0.07 | -5.97 | Man1a | mannosidase 1, alpha | metabolic process | mannosyl-oligosaccharide 1,2-alpha-mannosidase activity / calcium ion binding / hydrolase activity / hydrolase activity, acting on glycosyl bonds / metal ion binding | Golgi membrane / Golgi membrane / Golgi apparatus / membrane / integral to membrane / integral to membrane |
| 1428667_at | 0.14 | -6.12 | 0.54 | -2.66 | 0.94 | 1.21 | 0.64 | -1.58 | Maoa | monoamine oxidase A | catecholamine metabolic process / neurotransmitter catabolic process / dopamine catabolic process | protein binding / amine oxidase activity / electron carrier activity / oxidoreductase activity | mitochondrion / mitochondrion / mitochondrion / mitochondrial outer membrane / membrane / integral to membrane |
| 1426850_a_at | 0.21 | -5.57 | -0.08 | -6.14 | -0.40 | -3.60 | -0.74 | 0.22 | Map2k6 | mitogen-activated protein kinase kinase 6 | MAPKKK cascade / activation of MAPK activity / activation of MAPK activity / protein amino acid phosphorylation / protein amino acid phosphorylation / cardiac muscle contraction | nucleotide binding / protein kinase activity / protein serine/threonine kinase activity / protein tyrosine kinase activity / protein binding / protein binding / ATP binding / kinase activity / transferase activity | --- |
| 1424850_at | -0.35 | -2.49 | -0.77 | 3.83 | -0.37 | -2.05 | -0.21 | -4.39 | Map3k1 | mitogen-activated protein kinase kinase kinase 1 | protein amino acid phosphorylation / transforming growth factor beta receptor signaling pathway / JNK cascade / activation of JNK activity / apoptotic mitochondrial changes / regulation of cell migration / positive regulation of actin filament polymerization / wound healing / camera-type eye development | nucleotide binding / magnesium ion binding / protein kinase activity / protein kinase activity / protein serine/threonine kinase activity / MAP kinase kinase kinase activity / protein binding / protein binding / ATP binding / zinc ion binding / JUN kinase kinase activity / kinase activity / transferase activity / identical protein binding / metal ion binding | --- |
| 1438908_at | -0.28 | -4.94 | -0.79 | 0.80 | 0.09 | -6.15 | 0.00 | -6.10 | Map3k12 | mitogen-activated protein kinase kinase kinase 12 | protein amino acid phosphorylation / protein amino acid phosphorylation / histone phosphorylation / peptidyl-serine phosphorylation / peptidyl-threonine phosphorylation / protein amino acid autophosphorylation | nucleotide binding / magnesium ion binding / protein kinase activity / protein serine/threonine kinase activity / protein serine/threonine kinase activity / MAP kinase kinase kinase activity / protein tyrosine kinase activity / protein binding / ATP binding / kinase activity / transferase activity / protein kinase binding / protein homodimerization activity | cytoplasm / cytosol / plasma membrane / membrane |
| 1425985_s_at | -1.06 | 6.78 | -0.65 | 2.00 | -0.23 | -4.52 | -0.42 | -1.40 | Masp1 | mannan-binding lectin serine peptidase 1 | proteolysis / immune response / complement activation, classical pathway / innate immune response | catalytic activity / serine-type endopeptidase activity / calcium ion binding / sugar binding / peptidase activity / hydrolase activity | extracellular space |
| 1438602_s_at | -0.90 | 2.97 | -0.27 | -4.56 | -0.15 | -5.75 | -0.02 | -6.09 | Masp1 | mannan-binding lectin serine peptidase 1 | proteolysis / immune response / complement activation, classical pathway / innate immune response | catalytic activity / serine-type endopeptidase activity / calcium ion binding / sugar binding / peptidase activity / hydrolase activity | extracellular space |
| 1458508_at | -0.07 | -6.35 | 0.03 | -6.26 | -0.81 | 1.35 | -0.21 | -5.21 | Matr3 | matrin 3 | --- | nucleotide binding / nucleic acid binding / RNA binding / zinc ion binding / metal ion binding | intracellular / nucleus / nuclear matrix |
| 1425029_a_at | -0.64 | -1.50 | -0.36 | -4.28 | -0.86 | 0.80 | -0.47 | -3.14 | Mboat2 | membrane bound O-acyltransferase domain containing 2 | --- | acyltransferase activity / transferase activity | membrane / integral to membrane / integral to membrane |
| 1423960_at | -0.59 | 3.11 | -0.65 | 4.02 | -0.35 | -1.30 | -0.24 | -3.22 | Mboat5 | membrane bound O-acyltransferase domain containing 5 | phospholipid biosynthetic process | acyltransferase activity / transferase activity / 1-acylglycerophosphocholine O-acyltransferase activity | endoplasmic reticulum / membrane / integral to membrane |
| 1417227_at | -0.66 | 2.52 | -0.70 | 2.99 | -0.73 | 3.42 | -0.52 | 0.44 | Mccc1 | methylcrotonoyl-Coenzyme A carboxylase 1 (alpha) | metabolic process | nucleotide binding / catalytic activity / methylcrotonoyl-CoA carboxylase activity / ATP binding / biotin binding / ligase activity | mitochondrion / mitochondrion / mitochondrial inner membrane / mitochondrial matrix |
| 1457707_at | -0.26 | -5.10 | -0.76 | 0.82 | -0.46 | -2.66 | -0.42 | -3.11 | Mctp2 | multiple C2 domains, transmembrane 2 | --- | calcium ion binding / calcium ion binding / calcium-dependent phospholipid binding | membrane fraction / membrane / integral to membrane / integral to membrane |
| 1423605_a_at | 0.83 | 1.68 | 0.79 | 1.20 | 0.75 | 0.78 | 0.44 | -2.69 | Mdm2 | transformed mouse 3T3 cell double minute 2 | ubiquitin cycle / traversing start control point of mitotic cell cycle / protein ubiquitination / protein catabolic process | p53 binding / ubiquitin-protein ligase activity / protein binding / protein binding / zinc ion binding / ligase activity / metal ion binding | intracellular / nucleus / nucleus / nucleolus / cytoplasm / cytoplasm |
| 1427718_a_at | 0.83 | -0.13 | 1.16 | 2.81 | 0.97 | 1.26 | 0.48 | -3.36 | Mdm2 | transformed mouse 3T3 cell double minute 2 | ubiquitin cycle / traversing start control point of mitotic cell cycle / protein ubiquitination / protein catabolic process | p53 binding / ubiquitin-protein ligase activity / protein binding / protein binding / zinc ion binding / ligase activity / metal ion binding | intracellular / nucleus / nucleus / nucleolus / cytoplasm / cytoplasm |
| 1450992_a_at | -0.64 | 0.14 | -0.54 | -1.06 | -0.36 | -3.47 | -0.19 | -5.16 | Meis1 | Meis homeobox 1 | transcription / regulation of transcription, DNA-dependent / regulation of transcription, DNA-dependent / regulation of transcription from RNA polymerase II promoter / multicellular organismal development / regulation of transcription | DNA binding / DNA binding / transcription factor activity / RNA polymerase II transcription factor activity, enhancer binding / protein binding / sequence-specific DNA binding / sequence-specific DNA binding / sequence-specific DNA binding | nucleus / nucleus / transcription factor complex |
| 1418215_at | -0.46 | -2.68 | -0.44 | -2.88 | -0.92 | 2.61 | -0.59 | -0.98 | Mep1b | meprin 1 beta | proteolysis | meprin A activity / peptidase activity / metallopeptidase activity / zinc ion binding / astacin activity / hydrolase activity / metal ion binding | extracellular space / membrane fraction / membrane / integral to membrane / integral to membrane |
| 1422990_at | -0.65 | 0.42 | -0.34 | -3.61 | -0.13 | -5.82 | -0.13 | -5.64 | Met | met proto-oncogene | activation of MAPK activity / neuron migration / protein amino acid phosphorylation / multicellular organismal development / brain development / brain development / muscle development / muscle development / lactation / sperm motility / adult behavior / protein amino acid autophosphorylation / protein amino acid autophosphorylation / hepatocyte growth factor receptor signaling pathway / myoblast proliferation | nucleotide binding / protein kinase activity / protein tyrosine kinase activity / transmembrane receptor protein tyrosine kinase activity / receptor activity / hepatocyte growth factor receptor activity / protein binding / protein binding / ATP binding / kinase activity / transferase activity | extracellular space / membrane fraction / basal plasma membrane / basal plasma membrane / membrane / integral to membrane / integral to membrane / flagellum |
| 1435713_at | 0.28 | -4.35 | 0.30 | -3.87 | 0.61 | 0.42 | 0.17 | -5.23 | Mettl2 | methyltransferase like 2 | --- | methyltransferase activity / methyltransferase activity / S-adenosylmethionine-dependent methyltransferase activity / transferase activity | --- |
| 1452335_at | -0.60 | 0.47 | -0.20 | -5.11 | 0.02 | -6.30 | -0.01 | -6.09 | Mfsd8 | major facilitator superfamily domain containing 8 | transport | transporter activity | lysosome / lysosomal membrane / membrane / integral to membrane |
| 1459984_at | -0.59 | 0.06 | -0.17 | -5.45 | -0.39 | -2.72 | -0.23 | -4.59 | Mia3 | melanoma inhibitory activity 3 | --- | --- | membrane / integral to membrane |
| 1433755_at | 0.71 | 0.84 | 0.27 | -4.59 | 0.39 | -3.19 | -0.05 | -6.04 | Mier1 | mesoderm induction early response 1 homolog (Xenopus laevis | transcription / regulation of transcription, DNA-dependent | DNA binding | nucleus |
| 1434179_at | -0.18 | -5.61 | -0.28 | -4.45 | -0.15 | -5.69 | -0.64 | 0.29 | Mll3 | myeloid/lymphoid or mixed-lineage leukemia 3 | transcription / regulation of transcription, DNA-dependent / intracellular signaling cascade / chromatin modification | DNA binding / protein binding / methyltransferase activity / zinc ion binding / transferase activity / histone-lysine N-methyltransferase activity / metal ion binding | nucleus |
| 1431053_at | 0.06 | -6.38 | 0.09 | -6.10 | 0.71 | 0.18 | 0.33 | -4.05 | Mphosph9 | M-phase phosphoprotein 9 | --- | --- | --- |
| 1454158_at | 0.09 | -6.35 | -0.14 | -6.04 | 0.97 | 0.10 | 0.49 | -3.77 | Mpp7 | membrane protein, palmitoylated 7 (MAGUK p55 subfamily member 7) | --- | protein binding | tight junction / membrane / cell junction |
| 1424433_at | -0.82 | 2.53 | -0.32 | -3.83 | -0.23 | -4.88 | -0.40 | -2.67 | Msrb2 | methionine sulfoxide reductase B2 | protein repair | protein-methionine-R-oxide reductase activity / peptide-methionine-(S)-S-oxide reductase activity / zinc ion binding / zinc ion binding / oxidoreductase activity / metal ion binding | extracellular space / mitochondrion / mitochondrion |
| 1454997_at | -0.84 | 5.69 | -0.68 | 3.58 | -0.55 | 1.58 | -0.48 | 0.46 | Msrb3 | methionine sulfoxide reductase B3 | protein repair | protein-methionine-R-oxide reductase activity / peptide-methionine-(S)-S-oxide reductase activity / zinc ion binding / zinc ion binding / oxidoreductase activity / metal ion binding | mitochondrion / mitochondrion / mitochondrion / endoplasmic reticulum |
| 1422557_s_at | 0.49 | -3.85 | 1.15 | 1.90 | 0.31 | -5.20 | 0.27 | -5.23 | Mt1 | metallothionein 1 | cellular metal ion homeostasis / cellular zinc ion homeostasis / nitric oxide mediated signal transduction / detoxification of copper ion | copper ion binding / zinc ion binding / metal ion binding / metal ion binding | lysosome / cytosol |
| 1428942_at | 1.02 | -0.36 | 1.87 | 5.08 | 1.22 | 1.13 | 0.67 | -2.85 | Mt2 | metallothionein 2 | cellular zinc ion homeostasis / nitric oxide mediated signal transduction / detoxification of copper ion | zinc ion binding / metal ion binding | --- |
| 1421402_at | -0.70 | 2.03 | -0.05 | -6.18 | 0.11 | -5.88 | -0.06 | -5.97 | Mta3 | metastasis associated 3 | regulation of transcription, DNA-dependent | DNA binding / transcription factor activity / zinc ion binding / sequence-specific DNA binding / metal ion binding | nucleus / nucleus / cytoplasm / cytoplasm |
| 1419253_at | 1.26 | 1.78 | 0.78 | -1.85 | 1.09 | 0.56 | 0.74 | -2.08 | Mthfd2 | methylenetetrahydrofolate dehydrogenase (NAD+ dependent), methenyltetrahydrofolate cyclohydrolase | histidine biosynthetic process / purine nucleotide biosynthetic process / one-carbon compound metabolic process / metabolic process / amino acid biosynthetic process / methionine biosynthetic process / folic acid and derivative biosynthetic process | magnesium ion binding / catalytic activity / methenyltetrahydrofolate cyclohydrolase activity / methylenetetrahydrofolate dehydrogenase (NAD+) activity / methylenetetrahydrofolate dehydrogenase (NADP+) activity / binding / oxidoreductase activity / hydrolase activity | extracellular space / mitochondrion / mitochondrion |
| 1419254_at | 1.55 | 0.12 | 1.58 | 0.31 | 0.69 | -4.40 | 0.74 | -4.00 | Mthfd2 | methylenetetrahydrofolate dehydrogenase (NAD+ dependent), methenyltetrahydrofolate cyclohydrolase | histidine biosynthetic process / purine nucleotide biosynthetic process / one-carbon compound metabolic process / metabolic process / amino acid biosynthetic process / methionine biosynthetic process / folic acid and derivative biosynthetic process | magnesium ion binding / catalytic activity / methenyltetrahydrofolate cyclohydrolase activity / methylenetetrahydrofolate dehydrogenase (NAD+) activity / methylenetetrahydrofolate dehydrogenase (NADP+) activity / binding / oxidoreductase activity / hydrolase activity | extracellular space / mitochondrion / mitochondrion |
| 1419400_at | -0.62 | 1.54 | -0.56 | 0.67 | -0.40 | -1.71 | -0.23 | -4.25 | Mttp | microsomal triglyceride transfer protein | protein amino acid lipidation / lipid metabolic process / triacylglycerol metabolic process / transport / lipid transport / lipoprotein metabolic process / cholesterol homeostasis / lipoprotein transport | lipid transporter activity / lipid transporter activity / lipid binding | endoplasmic reticulum |
| 1436502_at | -0.82 | -0.36 | -1.13 | 2.43 | -0.64 | -2.01 | -0.37 | -4.32 | Mtus1 | mitochondrial tumor suppressor 1 | cell cycle | --- | nucleus / mitochondrion / Golgi apparatus / plasma membrane / membrane |
| 1454824_s_at | -0.79 | -1.04 | -1.02 | 1.03 | -0.83 | -0.60 | -0.98 | 0.66 | Mtus1 | mitochondrial tumor suppressor 1 | cell cycle | --- | nucleus / mitochondrion / Golgi apparatus / plasma membrane / membrane |
| 1448325_at | 1.45 | 2.11 | 1.78 | 4.04 | 1.28 | 1.06 | 1.05 | -0.51 | Myd116 | myeloid differentiation primary response gene 116 | regulation of translation / apoptosis / response to stress | --- | endoplasmic reticulum |
| 1425506_at | -0.49 | -1.21 | -0.74 | 2.22 | -0.71 | 1.93 | -0.73 | 2.01 | Mylk | myosin, light polypeptide kinase | protein amino acid phosphorylation / cytoskeleton organization and biogenesis | nucleotide binding / magnesium ion binding / protein kinase activity / protein serine/threonine kinase activity / myosin light chain kinase activity / calcium ion binding / calmodulin binding / ATP binding / kinase activity / transferase activity / metal ion binding | cytoplasm / cytoskeleton |
| 1452298_a_at | -0.35 | -1.95 | -0.66 | 3.31 | -0.27 | -3.31 | -0.16 | -4.91 | Myo5b | myosin Vb | cytoskeleton organization and biogenesis | nucleotide binding / motor activity / actin binding / calmodulin binding / ATP binding | cytoskeleton / myosin complex |
| 1416192_at | 0.27 | -2.89 | 0.40 | -0.14 | 0.62 | 3.60 | 0.29 | -2.23 | Napa | N-ethylmaleimide sensitive fusion protein attachment protein alpha | transport / intracellular protein transport / intracellular protein transport / brain development / protein transport / vesicle-mediated transport / neuron differentiation / apical protein localization | binding / protein binding / syntaxin binding | endoplasmic reticulum / Golgi apparatus / membrane |
| 1437872_at | -0.73 | 2.24 | -0.01 | -6.28 | -0.08 | -6.10 | -0.26 | -4.14 | Napepld | N-acyl phosphatidylethanolamine phospholipase D | lipid metabolic process / phospholipid catabolic process | zinc ion binding / hydrolase activity / metal ion binding | membrane |
| 1428666_at | 0.66 | 1.45 | 0.68 | 1.63 | 0.67 | 1.51 | 0.42 | -1.87 | Nars | asparaginyl-tRNA synthetase | translation / tRNA aminoacylation for protein translation / asparaginyl-tRNA aminoacylation / aspartyl-tRNA aminoacylation | nucleotide binding / nucleic acid binding / aminoacyl-tRNA ligase activity / aspartate-tRNA ligase activity / asparagine-tRNA ligase activity / protein binding / ATP binding / ligase activity | cytoplasm |
| 1431026_at | 0.16 | -5.93 | 0.44 | -3.07 | 0.82 | 1.16 | 0.54 | -1.87 | Nat12 | N-acetyltransferase 12 | metabolic process | N-acetyltransferase activity / acyltransferase activity / transferase activity | --- |
| 1456144_at | 0.72 | 0.22 | 0.17 | -5.64 | 0.04 | -6.27 | -0.29 | -4.54 | Nav3 | neuron navigator 3 | --- | nucleotide binding / nucleoside-triphosphatase activity | nucleus / nucleus / nuclear envelope / nuclear outer membrane / membrane |
| 1455694_at | 0.26 | -4.13 | 0.08 | -6.02 | 0.06 | -6.16 | 0.59 | 0.99 | Nbeal2 | neurobeachin-like 2 | --- | --- | --- |
| 1420808_at | -0.63 | 1.05 | -0.37 | -2.70 | -0.23 | -4.62 | -0.18 | -5.10 | Ncoa4 / EG627557 | nuclear receptor coactivator 4 / predicted gene, EG627557 | transmembrane receptor protein tyrosine kinase signaling pathway | receptor activity | --- |
| 1433720_s_at | 0.33 | -4.86 | 0.65 | -1.58 | 0.84 | 0.31 | 0.97 | 1.51 | Ndg2 | Nur77 downstream gene 2 | --- | --- | mitochondrion |
| 1436990_s_at | 0.65 | -1.85 | 1.34 | 4.32 | 2.04 | 8.34 | 1.66 | 5.98 | Ndg2 | Nur77 downstream gene 2 | --- | --- | mitochondrion |
| 1418068_at | 0.22 | -4.95 | 0.21 | -4.82 | 0.61 | 0.87 | 0.11 | -5.71 | Ndufa10 | NADH dehydrogenase (ubiquinone) 1 alpha subcomplex 10 | nucleobase, nucleoside, nucleotide and nucleic acid metabolic process / transport / oxidation reduction | ATP binding / phosphotransferase activity, alcohol group as acceptor | extracellular space / mitochondrion / mitochondrion / mitochondrial respiratory chain / mitochondrial matrix |
| 1418932_at | 0.49 | 0.64 | 0.61 | 2.54 | 0.37 | -1.55 | 0.20 | -4.26 | Nfil3 / LOC100046232 | nuclear factor, interleukin 3, regulated / similar to NFIL3/E4BP4 transcription factor | transcription / regulation of transcription, DNA-dependent / regulation of transcription, DNA-dependent / rhythmic process | DNA binding / transcription factor activity / sequence-specific DNA binding / protein dimerization activity | nucleus |
| 1421266_s_at | 0.94 | 4.49 | 0.42 | -2.15 | 0.29 | -3.97 | 0.29 | -3.79 | Nfkbib | nuclear factor of kappa light polypeptide gene enhancer in B-cells inhibitor, beta | --- | --- | nucleus / cytoplasm |
| 1448728_a_at | 0.64 | 2.42 | 0.12 | -5.61 | 0.19 | -4.82 | 0.40 | -1.24 | Nfkbiz | nuclear factor of kappa light polypeptide gene enhancer in B-cells inhibitor, zeta | transcription / regulation of transcription, DNA-dependent / regulation of transcription, DNA-dependent / inflammatory response | DNA binding / transcription factor activity | nucleus / nucleus |
| 1419267_at | 0.72 | 1.94 | 0.11 | -5.90 | 0.30 | -3.86 | -0.22 | -4.67 | Nfyb | nuclear transcription factor-Y beta | transcription / regulation of transcription, DNA-dependent / positive regulation of transcription, DNA-dependent | DNA binding / DNA binding / DNA binding / transcription factor activity / protein binding / sequence-specific DNA binding | intracellular / nucleus / nucleus / nucleus / transcription factor complex |
| 1417827_at | -0.83 | 3.80 | -0.36 | -2.72 | -0.42 | -1.83 | -0.36 | -2.53 | Ngly1 | N-glycanase 1 | glycoprotein catabolic process | peptide-N4-(N-acetyl-beta-glucosaminyl)asparagine amidase activity / peptide-N4-(N-acetyl-beta-glucosaminyl)asparagine amidase activity / protein binding / zinc ion binding / hydrolase activity / metal ion binding | nucleus / cytoplasm / cytoplasm |
| 1416931_at | 0.39 | -1.66 | 0.23 | -4.25 | 0.65 | 2.49 | 0.37 | -1.75 | Nif3l1 | Ngg1 interacting factor 3-like 1 (S. pombe) | --- | --- | cytoplasm / cytoplasm |
| 1417278_a_at | -1.35 | 0.19 | -1.05 | -1.56 | -0.76 | -3.42 | -0.75 | -3.31 | Nkd1 | naked cuticle 1 homolog (Drosophila) | multicellular organismal development / spermatogenesis / Wnt receptor signaling pathway / cell differentiation | calcium ion binding / calcium ion binding / protein binding / protein binding | cytoplasm / plasma membrane / membrane |
| 1429506_at | -1.39 | 2.17 | -0.94 | -0.98 | -0.64 | -3.32 | -0.65 | -3.06 | Nkd1 | naked cuticle 1 homolog (Drosophila) | multicellular organismal development / spermatogenesis / Wnt receptor signaling pathway / cell differentiation | calcium ion binding / calcium ion binding / protein binding / protein binding | cytoplasm / plasma membrane / membrane |
| 1448133_at | 0.36 | -2.81 | 0.40 | -2.12 | 0.69 | 2.20 | 0.28 | -3.65 | Nmd3 | NMD3 homolog (S. cerevisiae) | ribosomal large subunit export from nucleus / transport / protein transport | ribosomal large subunit binding | nucleus / nucleus / cytoplasm / cytoplasm |
| 1418634_at | 0.17 | -5.53 | 0.47 | -1.44 | 0.62 | 0.69 | 0.42 | -2.03 | Notch1 | Notch gene homolog 1 (Drosophila) | cell fate specification / epithelial to mesenchymal transition / sprouting angiogenesis / transcription / regulation of transcription, DNA-dependent / regulation of transcription from RNA polymerase II promoter / Notch signaling pathway / Notch signaling pathway / multicellular organismal development / determination of left/right symmetry / compartment specification / axonogenesis / axonogenesis / foregut morphogenesis / endoderm development / heart development / positive regulation of cell proliferation / epidermis development / glial cell differentiation / cell differentiation / cell differentiation / keratinocyte differentiation / lung development / embryonic limb morphogenesis / forebrain development / forebrain development / hair follicle morphogenesis / embryonic hindlimb morphogenesis / anagen / positive regulation of apoptosis / positive regulation of apoptosis / negative regulation of apoptosis / cell fate commitment / negative regulation of cell differentiation / positive regulation of keratinocyte differentiation / positive regulation of keratinocyte differentiation / negative regulation of neuron differentiation / positive regulation of transcription from RNA polymerase II promoter / positive regulation of transcription from RNA polymerase II promoter / negative regulation of photoreceptor cell differentiation / somatic stem cell division / somatic stem cell division / neuron fate commitment / branching morphogenesis of a tube / regulation of epithelial cell proliferation / regulation of developmental process | chromatin binding / transcription factor activity / receptor activity / calcium ion binding / protein binding / chromatin DNA binding / sequence-specific DNA binding / metal ion binding | extracellular space / nucleus / nucleus / cytoplasm / plasma membrane / plasma membrane / integral to plasma membrane / membrane / integral to membrane |
| 1453345_at | 0.93 | 0.99 | 0.58 | -2.39 | -0.08 | -6.21 | 0.14 | -5.79 | Npal1 | NIPA-like domain containing 1 | --- | --- | membrane / integral to membrane |
| 1433545_s_at | -0.39 | -4.14 | 0.13 | -6.00 | 0.82 | 0.50 | 0.28 | -4.85 | Nphp3 / Acad11 | nephronophthisis 3 (adolescent) / acyl-Coenzyme A dehydrogenase family, member 11 | lipid metabolic process / metabolic process / maintenance of organ identity / oxidation reduction | acyl-CoA dehydrogenase activity / binding / electron carrier activity / oxidoreductase activity / oxidoreductase activity, acting on the CH-CH group of donors / FAD binding | peroxisome |
| 1423627_at | 0.02 | -6.44 | 0.26 | -5.12 | 1.03 | 2.61 | 0.24 | -5.09 | Nqo1 | NAD(P)H dehydrogenase, quinone 1 | response to oxidative stress | NAD(P)H dehydrogenase (quinone) activity / NAD(P)H dehydrogenase (quinone) activity / electron carrier activity / oxidoreductase activity / coenzyme binding | cytoplasm |
| 1426464_at | 0.42 | -2.41 | 0.57 | -0.09 | 0.71 | 1.68 | 0.19 | -5.07 | Nr1d1 | nuclear receptor subfamily 1, group D, member 1 | transcription / regulation of transcription, DNA-dependent / regulation of transcription, DNA-dependent / circadian rhythm | DNA binding / DNA binding / transcription factor activity / transcription factor activity / steroid hormone receptor activity / receptor activity / ligand-dependent nuclear receptor activity / zinc ion binding / sequence-specific DNA binding / metal ion binding | nucleus / nucleus |
| 1419105_at | 0.61 | -0.54 | 0.67 | 0.33 | 0.35 | -3.71 | -0.21 | -5.06 | Nr1h4 | nuclear receptor subfamily 1, group H, member 4 | transcription / regulation of transcription, DNA-dependent / bile acid metabolic process | DNA binding / transcription factor activity / steroid hormone receptor activity / receptor activity / ligand-dependent nuclear receptor activity / zinc ion binding / sequence-specific DNA binding / metal ion binding | nucleus |
| 1457635_s_at | -0.58 | -1.45 | -0.91 | 2.21 | -0.22 | -5.34 | -0.19 | -5.36 | Nr3c1 | nuclear receptor subfamily 3, group C, member 1 | regulation of gluconeogenesis / transcription / regulation of transcription, DNA-dependent / regulation of transcription, DNA-dependent / chromatin modification / adrenal gland development / regulation of glucocorticoid biosynthetic process / glucocorticoid receptor signaling pathway / glucocorticoid receptor signaling pathway | DNA binding / DNA binding / transcription factor activity / steroid hormone receptor activity / receptor activity / ligand-dependent nuclear receptor activity / glucocorticoid receptor activity / glucocorticoid receptor activity / steroid binding / protein binding / zinc ion binding / lipid binding / transcription regulator activity / sequence-specific DNA binding / metal ion binding | intracellular / nucleus / nucleus / cytoplasm / cytosol / membrane |
| 1447863_s_at | 0.87 | 1.64 | 0.83 | 1.18 | 0.72 | 0.08 | 0.93 | 2.10 | Nr4a2 | nuclear receptor subfamily 4, group A, member 2 | transcription / regulation of transcription, DNA-dependent / nervous system development / neuron differentiation / regulation of dopamine metabolic process / positive regulation of transcription from RNA polymerase II promoter / positive regulation of transcription from RNA polymerase II promoter | DNA binding / DNA binding / transcription factor activity / steroid hormone receptor activity / receptor activity / ligand-dependent nuclear receptor activity / ligand-dependent nuclear receptor activity / protein binding / zinc ion binding / sequence-specific DNA binding / metal ion binding | nucleus / nucleus |
| 1450750_a_at | 0.92 | 2.28 | 0.48 | -2.52 | 0.33 | -4.23 | 0.47 | -2.57 | Nr4a2 | nuclear receptor subfamily 4, group A, member 2 | transcription / regulation of transcription, DNA-dependent / nervous system development / neuron differentiation / regulation of dopamine metabolic process / positive regulation of transcription from RNA polymerase II promoter / positive regulation of transcription from RNA polymerase II promoter | DNA binding / DNA binding / transcription factor activity / steroid hormone receptor activity / receptor activity / ligand-dependent nuclear receptor activity / ligand-dependent nuclear receptor activity / protein binding / zinc ion binding / sequence-specific DNA binding / metal ion binding | nucleus / nucleus |
| 1455034_at | 1.20 | 3.42 | 0.74 | -0.74 | 1.27 | 3.93 | 0.69 | -1.09 | Nr4a2 | nuclear receptor subfamily 4, group A, member 2 | transcription / regulation of transcription, DNA-dependent / nervous system development / neuron differentiation / regulation of dopamine metabolic process / positive regulation of transcription from RNA polymerase II promoter / positive regulation of transcription from RNA polymerase II promoter | DNA binding / DNA binding / transcription factor activity / steroid hormone receptor activity / receptor activity / ligand-dependent nuclear receptor activity / ligand-dependent nuclear receptor activity / protein binding / zinc ion binding / sequence-specific DNA binding / metal ion binding | nucleus / nucleus |
| 1428393_at | -0.87 | -2.04 | -0.78 | -2.60 | -1.22 | 0.51 | -0.86 | -1.93 | Nrn1 | neuritin 1 | axonogenesis | --- | plasma membrane / membrane / anchored to membrane |
| 1426528_at | 1.26 | 5.16 | 0.80 | 0.87 | 0.48 | -2.67 | 0.41 | -3.32 | Nrp2 | neuropilin 2 | neural crest cell migration / cell adhesion / multicellular organismal development / nervous system development / heart development / cell differentiation / negative chemotaxis | receptor activity / semaphorin receptor activity | extracellular space / plasma membrane / membrane / integral to membrane / integral to membrane |
| 1435349_at | 1.72 | 8.57 | 1.37 | 6.10 | 0.85 | 1.69 | 0.76 | 0.68 | Nrp2 | neuropilin 2 | neural crest cell migration / cell adhesion / multicellular organismal development / nervous system development / heart development / cell differentiation / negative chemotaxis | receptor activity / semaphorin receptor activity | extracellular space / plasma membrane / membrane / integral to membrane / integral to membrane |
| 1438684_at | -0.60 | 1.23 | -0.45 | -1.06 | -0.53 | 0.25 | -0.52 | 0.12 | Nuak1 | NUAK family, SNF1-like kinase, 1 | protein amino acid phosphorylation | nucleotide binding / protein kinase activity / protein serine/threonine kinase activity / ATP binding / kinase activity / transferase activity | --- |
| 1416512_at | 0.11 | -6.04 | 0.24 | -4.59 | 0.76 | 2.50 | 0.57 | 0.06 | Nubp2 | nucleotide binding protein 2 | --- | nucleotide binding / ATP binding | nucleus |
| 1418355_at | -0.17 | -5.50 | -0.08 | -6.07 | -0.49 | -1.00 | -0.62 | 0.75 | Nucb2 | nucleobindin 2 | cellular calcium ion homeostasis | DNA binding / calcium ion binding / calcium ion binding | extracellular space / nucleus / nuclear outer membrane / cytoplasm / endoplasmic reticulum / endoplasmic reticulum / ER-Golgi intermediate compartment / perikaryon |
| 1419665_a_at | 3.18 | 14.77 | 2.23 | 11.38 | 1.68 | 9.11 | 1.35 | 6.63 | Nupr1 | nuclear protein 1 | cell growth | --- | nucleus / nucleus |
| 1419666_x_at | 2.09 | 8.41 | 1.65 | 5.88 | 1.09 | 1.97 | 1.02 | 1.36 | Nupr1 | nuclear protein 1 | cell growth | --- | nucleus / nucleus |
| 1418686_at | -0.67 | 1.44 | -0.28 | -3.99 | 0.01 | -6.31 | -0.14 | -5.49 | Oas1c | 2'-5' oligoadenylate synthetase 1C | immune response | 2'-5'-oligoadenylate synthetase activity / nucleic acid binding / RNA binding / double-stranded RNA binding / ATP binding / transferase activity | --- |
| 1427364_a_at | 0.65 | 3.46 | 0.30 | -2.65 | -0.12 | -5.50 | 0.13 | -5.22 | Odc1 | ornithine decarboxylase, structural 1 | kidney development / polyamine biosynthetic process / positive regulation of cell proliferation | catalytic activity / ornithine decarboxylase activity / ornithine decarboxylase activity / protein binding / lyase activity / carboxy-lyase activity | cytosol |
| 1423915_at | -0.72 | 0.22 | -0.10 | -6.05 | -0.23 | -5.21 | 0.10 | -5.87 | Olfml2b | olfactomedin-like 2B | --- | latrotoxin receptor activity / protein homodimerization activity / extracellular matrix binding | extracellular region / membrane / extracellular matrix |
| 1440005_at | 0.32 | -4.44 | 0.82 | 1.46 | -0.10 | -6.07 | -0.01 | -6.10 | Onecut2 | one cut domain, family member 2 | liver development / regulation of cell-matrix adhesion / epithelial cell development / transcription / regulation of transcription, DNA-dependent / anatomical structure morphogenesis / positive regulation of cell migration / negative regulation of transforming growth factor beta receptor signaling pathway / pancreas development / endocrine pancreas development / cilium biogenesis / cell fate commitment / regulation of transcription / positive regulation of transcription from RNA polymerase II promoter / peripheral nervous system neuron development | DNA binding / DNA binding / transcription factor activity / transcription factor activity / sequence-specific DNA binding | nucleus / nucleus |
| 1444980_at | 0.65 | 0.74 | 0.79 | 2.45 | 0.47 | -1.66 | -0.02 | -6.09 | Onecut2 | one cut domain, family member 2 | liver development / regulation of cell-matrix adhesion / epithelial cell development / transcription / regulation of transcription, DNA-dependent / anatomical structure morphogenesis / positive regulation of cell migration / negative regulation of transforming growth factor beta receptor signaling pathway / pancreas development / endocrine pancreas development / cilium biogenesis / cell fate commitment / regulation of transcription / positive regulation of transcription from RNA polymerase II promoter / peripheral nervous system neuron development | DNA binding / DNA binding / transcription factor activity / transcription factor activity / sequence-specific DNA binding | nucleus / nucleus |
| 1434763_at | 0.38 | -3.26 | 0.25 | -4.77 | 0.71 | 1.10 | 0.08 | -5.90 | Orai2 | ORAI calcium release-activated calcium modulator 2 | --- | --- | membrane / integral to membrane |
| 1428557_a_at | -0.75 | 0.68 | -0.39 | -3.46 | -0.36 | -3.81 | -0.23 | -5.01 | Osgepl1 | O-sialoglycoprotein endopeptidase-like 1 | proteolysis / proteolysis | endopeptidase activity / peptidase activity / metallopeptidase activity / zinc ion binding / zinc ion binding / O-sialoglycoprotein endopeptidase activity / O-sialoglycoprotein endopeptidase activity / hydrolase activity / metal ion binding | --- |
| 1428558_at | -0.64 | 3.21 | -0.14 | -5.23 | -0.24 | -3.68 | -0.03 | -6.04 | Osgepl1 | O-sialoglycoprotein endopeptidase-like 1 | proteolysis / proteolysis | endopeptidase activity / peptidase activity / metallopeptidase activity / zinc ion binding / zinc ion binding / O-sialoglycoprotein endopeptidase activity / O-sialoglycoprotein endopeptidase activity / hydrolase activity / metal ion binding | --- |
| 1424022_at | 1.39 | 7.10 | 1.30 | 6.23 | 0.60 | -0.69 | 0.42 | -2.77 | Osgin1 | oxidative stress induced growth inhibitor 1 | --- | --- | --- |
| 1449350_at | -0.74 | 2.38 | 0.04 | -6.22 | -0.11 | -5.94 | 0.04 | -6.05 | Osr1 | odd-skipped related 1 (Drosophila) | metanephros development / heart development / gonad development / intermediate mesoderm development | nucleic acid binding / zinc ion binding / metal ion binding | intracellular / nucleus |
| 1448817_at | -0.07 | -6.26 | -0.12 | -5.70 | 0.58 | 0.84 | 0.20 | -4.65 | Otub1 / LOC100046081 | OTU domain, ubiquitin aldehyde binding 1 / similar to OTU domain, ubiquitin aldehyde binding 1 | ubiquitin cycle / immune response | peptidase activity / cysteine-type peptidase activity / hydrolase activity | --- |
| 1431724_a_at | -1.39 | 6.85 | -0.87 | 2.17 | -0.70 | 0.28 | -0.85 | 1.89 | P2ry12 | purinergic receptor P2Y, G-protein coupled 12 | signal transduction / G-protein coupled receptor protein signaling pathway / G-protein signaling, coupled to cAMP nucleotide second messenger / platelet activation | rhodopsin-like receptor activity / adenosine receptor activity, G-protein coupled / platelet ADP receptor activity / signal transducer activity / receptor activity / G-protein coupled receptor activity / purinergic nucleotide receptor activity, G-protein coupled | plasma membrane / integral to plasma membrane / membrane / integral to membrane / integral to membrane |
| 1424733_at | -0.87 | 2.09 | -0.79 | 1.27 | -0.63 | -0.61 | -0.38 | -3.44 | P2ry14 | purinergic receptor P2Y, G-protein coupled, 14 | immune response / signal transduction / G-protein coupled receptor protein signaling pathway | rhodopsin-like receptor activity / signal transducer activity / receptor activity / G-protein coupled receptor activity / purinergic nucleotide receptor activity, G-protein coupled | plasma membrane / membrane / integral to membrane |
| 1450854_at | 0.34 | -4.64 | 0.21 | -5.50 | 0.89 | 1.21 | 0.50 | -2.68 | Pa2g4 | proliferation-associated 2G4 | transcription / regulation of transcription, DNA-dependent / rRNA processing / regulation of translation / proteolysis / negative regulation of transcription, DNA-dependent / negative regulation of transcription, DNA-dependent | DNA binding / transcription factor activity / transcription factor activity / RNA binding / methionyl aminopeptidase activity / protein binding / protein binding / metalloexopeptidase activity | nucleus / nucleus / cytoplasm / ribonucleoprotein complex |
| 1417251_at | -1.17 | 1.34 | -0.79 | -1.62 | -0.95 | -0.28 | -0.69 | -2.34 | Palmd | palmdelphin | regulation of cell shape | --- | cytoplasm / cytoplasm / membrane |
| 1428859_at | 0.37 | -3.33 | 0.64 | 0.39 | 0.56 | -0.58 | 0.34 | -3.39 | Paox / LOC624275 | polyamine oxidase (exo-N4-amino) / similar to peroxisomal N1-acetyl-spermine/spermidine oxidase | polyamine catabolic process / polyamine catabolic process | electron carrier activity / oxidoreductase activity / polyamine oxidase activity / polyamine oxidase activity / polyamine oxidase activity | cytoplasm / peroxisome |
| 1415947_at | 0.07 | -6.30 | 0.72 | 1.18 | 0.66 | 0.49 | 0.80 | 2.10 | Pappa2 / Creg1 | pappalysin 2 / cellular repressor of E1A-stimulated genes 1 | regulation of cell growth / regulation of transcription, DNA-dependent / proteolysis / proteolysis / cell differentiation | metalloendopeptidase activity / transcription factor binding / metallopeptidase activity / zinc ion binding | extracellular region / transcription factor complex / membrane |
| 1415890_at | -0.43 | -1.72 | -0.50 | -0.68 | -0.64 | 1.35 | -0.48 | -0.83 | Papss1 | 3'-phosphoadenosine 5'-phosphosulfate synthase 1 | sulfate assimilation / 3'-phosphoadenosine 5'-phosphosulfate biosynthetic process | nucleotide binding / catalytic activity / adenylylsulfate kinase activity / adenylylsulfate kinase activity / sulfate adenylyltransferase (ATP) activity / sulfate adenylyltransferase (ATP) activity / sulfate adenylyltransferase (ATP) activity / ATP binding / kinase activity / transferase activity / transferase activity, transferring phosphorus-containing groups / nucleotidyltransferase activity / nucleotidyltransferase activity | sulfate adenylyltransferase complex (ATP) |
| 1434510_at | -0.05 | -6.38 | -0.21 | -5.03 | -0.77 | 2.25 | -0.67 | 1.01 | Papss2 | 3'-phosphoadenosine 5'-phosphosulfate synthase 2 | sulfate assimilation / sulfate assimilation | nucleotide binding / catalytic activity / adenylylsulfate kinase activity / adenylylsulfate kinase activity / sulfate adenylyltransferase (ATP) activity / sulfate adenylyltransferase (ATP) activity / sulfate adenylyltransferase (ATP) activity / ATP binding / kinase activity / transferase activity / transferase activity, transferring phosphorus-containing groups / nucleotidyltransferase activity / nucleotidyltransferase activity | --- |
| 1451969_s_at | 0.14 | -5.77 | 0.22 | -4.69 | 0.60 | 1.01 | 0.35 | -2.61 | Parp3 | poly (ADP-ribose) polymerase family, member 3 | protein amino acid ADP-ribosylation | NAD+ ADP-ribosyltransferase activity / transferase activity | nucleus |
| 1419271_at | -0.03 | -6.43 | 0.66 | 0.25 | 0.11 | -6.02 | -0.07 | -5.97 | Pax6 | paired box gene 6 | cell fate determination / neuron migration / transcription / regulation of transcription, DNA-dependent / regulation of transcription, DNA-dependent / multicellular organismal development / axonogenesis / axon guidance / brain development / salivary gland morphogenesis / negative regulation of cell proliferation / dorsal/ventral axis specification / anterior/posterior pattern formation / anterior/posterior pattern formation / dorsal/ventral pattern formation / pallium development / forebrain anterior/posterior pattern formation / forebrain dorsal/ventral pattern formation / commitment of a neuronal cell to a specific type of neuron in the forebrain / forebrain-midbrain boundary formation / pituitary gland development / cell differentiation / cell differentiation / keratinocyte differentiation / regulation of cell migration / positive regulation of epithelial cell differentiation / forebrain development / eye photoreceptor cell development / camera-type eye development / camera-type eye development / cell fate commitment / cell fate commitment / regulation of transcription / negative regulation of neuron differentiation / positive regulation of transcription from RNA polymerase II promoter / negative regulation of epithelial cell proliferation / regulation of neurogenesis | DNA binding / DNA binding / transcription factor activity / transcription factor activity / protein binding / sequence-specific DNA binding | nucleus / nucleus / transcription factor complex |
| 1421193_a_at | -0.63 | 1.54 | -0.47 | -0.73 | -0.38 | -2.21 | -0.18 | -4.95 | Pbx3 | pre B-cell leukemia transcription factor 3 | transcription / regulation of transcription, DNA-dependent / anterior compartment specification / posterior compartment specification / respiratory gaseous exchange / regulation of transcription | DNA binding / transcription factor activity / protein binding / sequence-specific DNA binding / sequence-specific DNA binding | nucleus / nucleus / transcription factor complex / transcription factor complex |
| 1434037_s_at | -0.47 | 0.64 | -0.45 | 0.27 | -0.61 | 2.92 | -0.39 | -0.68 | Pcaf | p300/CBP-associated factor | transcription / regulation of transcription, DNA-dependent / regulation of transcription, DNA-dependent / protein amino acid acetylation / cell cycle / metabolic process / histone acetylation / positive regulation of transcription | transcription coactivator activity / histone acetyltransferase activity / histone acetyltransferase activity / H3/H4 histone acetyltransferase activity / protein binding / N-acetyltransferase activity / N-acetyltransferase activity / acyltransferase activity / transferase activity | histone acetyltransferase complex / kinetochore / nucleus / nucleus |
| 1429861_at | -0.06 | -6.38 | 0.77 | 0.32 | -0.02 | -6.30 | -0.33 | -4.30 | Pcdh9 | protocadherin 9 | homophilic cell adhesion | calcium ion binding / protein binding | integral to plasma membrane / membrane |
| 1420984_at | -0.10 | -6.17 | 0.06 | -6.17 | 0.68 | 0.61 | 0.33 | -3.75 | Pctp | phosphatidylcholine transfer protein | transport / lipid transport / cholesterol metabolic process | lipid binding | cytoplasm |
| 1433761_at | -0.51 | -2.05 | -0.68 | 0.13 | -0.71 | 0.47 | -0.75 | 0.83 | Pde4dip | phosphodiesterase 4D interacting protein (myomegalin) | --- | protein binding | nucleus / cytoplasm / Golgi apparatus / centrosome |
| 1419123_a_at | -0.40 | -1.52 | 0.01 | -6.28 | 0.59 | 1.61 | 0.76 | 3.79 | Pdgfc | platelet-derived growth factor, C polypeptide | activation of transmembrane receptor protein tyrosine kinase activity / cell proliferation / positive regulation of cell proliferation / organ morphogenesis / organ morphogenesis / platelet-derived growth factor receptor signaling pathway / regulation of peptidyl-tyrosine phosphorylation | platelet-derived growth factor receptor binding / growth factor activity | extracellular region / extracellular space / membrane |
| 1421917_at | -0.70 | -0.97 | -0.20 | -5.59 | -1.02 | 2.26 | -0.72 | -0.63 | Pdgfra | platelet derived growth factor receptor, alpha polypeptide | protein amino acid phosphorylation / transmembrane receptor protein tyrosine kinase signaling pathway / anatomical structure morphogenesis / organ morphogenesis / extracellular matrix organization and biogenesis / lung development / male genitalia development / odontogenesis of dentine-containing teeth | nucleotide binding / protein kinase activity / protein tyrosine kinase activity / transmembrane receptor protein tyrosine kinase activity / receptor activity / vascular endothelial growth factor receptor activity / ATP binding / kinase activity / transferase activity / platelet-derived growth factor binding | membrane / integral to membrane |
| 1428896_at | 0.79 | -0.11 | 0.75 | -0.37 | -0.84 | 0.48 | -0.30 | -4.77 | Pdgfrl | platelet-derived growth factor receptor-like | --- | --- | extracellular region |
| 1427475_a_at | -0.82 | 3.50 | -0.30 | -3.77 | -0.44 | -1.65 | -0.37 | -2.51 | Pdlim5 | PDZ and LIM domain 5 | --- | protein binding / protein binding / zinc ion binding / metal ion binding | Z disc |
| 1416173_at | 0.24 | -4.64 | 0.33 | -3.27 | 0.62 | 0.91 | 0.37 | -2.52 | Pes1 | pescadillo homolog 1, containing BRCT domain (zebrafish) | mitotic metaphase / nucleolus organization and biogenesis / cell proliferation / ribosome biogenesis and assembly | --- | condensed chromosome / intracellular / nucleus / nucleus / nucleolus / nucleolus |
| 1416432_at | 0.91 | 4.50 | 0.27 | -4.05 | 0.51 | -0.64 | 0.28 | -3.81 | Pfkfb3 | 6-phosphofructo-2-kinase/fructose-2,6-biphosphatase 3 | fructose 2,6-bisphosphate metabolic process / fructose 2,6-bisphosphate metabolic process / metabolic process / metabolic process | catalytic activity / catalytic activity / 6-phosphofructo-2-kinase activity / fructose-2,6-bisphosphate 2-phosphatase activity / fructose-2,6-bisphosphate 2-phosphatase activity / ATP binding / ATP binding / kinase activity / kinase activity / transferase activity / transferase activity / hydrolase activity / hydrolase activity | --- |
| 1416780_at | -0.74 | 0.75 | -0.65 | -0.24 | -0.73 | 0.62 | -0.52 | -1.72 | Pfkm | phosphofructokinase, muscle | fructose 6-phosphate metabolic process / glycolysis / fructose 1,6-bisphosphate metabolic process | nucleotide binding / magnesium ion binding / catalytic activity / 6-phosphofructokinase activity / 6-phosphofructokinase activity / ATP binding / sugar binding / phosphofructokinase activity / kinase activity / transferase activity / metal ion binding | soluble fraction / cytoplasm / 6-phosphofructokinase complex / 6-phosphofructokinase complex |
| 1436317_at | -0.02 | -6.45 | -0.52 | -4.49 | -1.56 | 2.28 | -1.18 | -0.05 | Pgap1 | post-GPI attachment to proteins 1 | GPI anchor metabolic process / transport / intracellular protein transport / protein transport / myo-inositol transport / myo-inositol transport | catalytic activity / nuclease activity / nuclease activity / hydrolase activity / hydrolase activity, acting on ester bonds / phosphoric ester hydrolase activity / phosphoric ester hydrolase activity | endoplasmic reticulum / endoplasmic reticulum / endoplasmic reticulum membrane / membrane / integral to membrane / intrinsic to endoplasmic reticulum membrane |
| 1437240_at | -0.27 | -5.88 | -0.36 | -5.33 | -1.16 | 0.01 | -0.20 | -5.79 | Pgm2 | phosphoglucomutase 2 | carbohydrate metabolic process / glucose metabolic process / glucose metabolic process | magnesium ion binding / magnesium ion binding / phosphoglucomutase activity / phosphoglucomutase activity / isomerase activity / intramolecular transferase activity, phosphotransferases / metal ion binding | cytoplasm |
| 1434511_at | -0.64 | 1.55 | -0.65 | 1.72 | -0.23 | -4.59 | -0.40 | -1.79 | Phkb | phosphorylase kinase beta | carbohydrate metabolic process / glycogen metabolic process | catalytic activity / calmodulin binding / kinase activity | plasma membrane / membrane |
| 1427903_at | -0.16 | -5.74 | -0.69 | 1.06 | -0.02 | -6.30 | 0.14 | -5.54 | Phpt1 | phosphohistidine phosphatase 1 | --- | phosphoprotein phosphatase activity / hydrolase activity | cytoplasm |
| 1448896_at | 0.28 | -3.55 | 0.21 | -4.53 | 0.60 | 1.70 | 0.51 | 0.31 | Pigf | phosphatidylinositol glycan anchor biosynthesis, class F | GPI anchor biosynthetic process / GPI anchor biosynthetic process | ethanolaminephosphotransferase activity / protein binding / phosphotransferase activity, for other substituted phosphate groups | endoplasmic reticulum / endoplasmic reticulum membrane / endoplasmic reticulum membrane / membrane / integral to membrane / integral to membrane |
| 1456577_x_at | -0.30 | -4.37 | -0.73 | 1.08 | -0.29 | -4.42 | -0.29 | -4.27 | Pitrm1 | pitrilysin metallepetidase 1 | proteolysis / proteolysis | catalytic activity / metalloendopeptidase activity / metalloendopeptidase activity / peptidase activity / metallopeptidase activity / zinc ion binding / hydrolase activity / metal ion binding | mitochondrion / mitochondrial matrix / mitochondrial matrix |
| 1426448_at | -0.32 | -3.52 | -0.47 | -1.07 | -0.25 | -4.37 | -0.62 | 1.01 | Pja1 | praja1, RING-H2 motif containing | ubiquitin cycle / protein catabolic process | ubiquitin-protein ligase activity / protein binding / protein binding / zinc ion binding / ligase activity / metal ion binding | cytoplasm |
| 1434383_at | -0.62 | 1.81 | -0.40 | -1.55 | -0.53 | 0.47 | -0.60 | 1.54 | Pja2 | praja 2, RING-H2 motif containing | ubiquitin cycle | protein binding / protein binding / zinc ion binding / ligase activity / metal ion binding | Golgi membrane / cytoplasm / endoplasmic reticulum / endoplasmic reticulum membrane / Golgi apparatus / membrane / cell junction / synapse / postsynaptic membrane |
| 1419820_at | -1.07 | 1.38 | -0.88 | -0.19 | -0.66 | -2.21 | -0.47 | -3.71 | Pkhd1 | polycystic kidney and hepatic disease 1 | kidney development / cell-cell adhesion / cell-cell adhesion / negative regulation of cell motility / negative regulation of cell motility | calcium ion binding / protein binding | basal body / apical plasma membrane |
| 1437893_at | 0.18 | -5.65 | 0.09 | -6.09 | 0.68 | 0.41 | 0.29 | -4.28 | Plb1 | phospholipase B1 | lipid metabolic process / lipid catabolic process | lysophospholipase activity / phospholipase A2 activity / lipase activity / hydrolase activity / hydrolase activity, acting on ester bonds | membrane / integral to membrane / apical plasma membrane |
| 1435043_at | -0.63 | -0.13 | -0.59 | -0.57 | -0.65 | 0.14 | -0.95 | 3.30 | Plcb1 | phospholipase C, beta 1 | lipid metabolic process / signal transduction / intracellular signaling cascade / lipid catabolic process / lipid catabolic process | phosphoinositide phospholipase C activity / phosphoinositide phospholipase C activity / phospholipase C activity / phospholipase C activity / signal transducer activity / signal transducer activity / calcium ion binding / hydrolase activity / hydrolase activity | cytoplasm |
| 1423725_at | -0.43 | -1.71 | -0.76 | 2.91 | -0.49 | -0.83 | -0.45 | -1.26 | Pls3 | plastin 3 (T-isoform) | actin filament organization | actin binding / actin binding / calcium ion binding / calcium ion binding / protein binding | cytoplasm |
| 1435254_at | -0.08 | -6.37 | -0.30 | -5.40 | -1.07 | 0.56 | -0.49 | -4.00 | Plxnb1 | plexin B1 | signal transduction / multicellular organismal development / positive regulation of axonogenesis | receptor activity / protein binding | intracellular / membrane / integral to membrane |
| 1424167_a_at | 0.45 | -3.74 | 0.96 | 1.43 | 1.32 | 4.37 | 0.98 | 1.62 | Pmm1 | phosphomannomutase 1 | metabolic process / mannose biosynthetic process | catalytic activity / phosphomannomutase activity / isomerase activity | cytoplasm |
| 1430780_a_at | 0.20 | -5.77 | 1.00 | 1.97 | 1.23 | 3.88 | 0.60 | -1.89 | Pmm1 | phosphomannomutase 1 | metabolic process / mannose biosynthetic process | catalytic activity / phosphomannomutase activity / isomerase activity | cytoplasm |
| 1427893_a_at | -0.25 | -5.43 | -0.80 | 0.08 | -0.91 | 1.10 | -0.38 | -4.08 | Pmvk | phosphomevalonate kinase | steroid biosynthetic process / cholesterol biosynthetic process / lipid biosynthetic process / sterol biosynthetic process | phosphomevalonate kinase activity / phosphomevalonate kinase activity / kinase activity / transferase activity | cytoplasm / peroxisome |
| 1416378_at | -0.23 | -4.47 | 0.06 | -6.14 | 0.64 | 2.19 | 0.38 | -1.73 | Pnkp | polynucleotide kinase 3'- phosphatase | DNA repair / response to DNA damage stimulus | nucleotide binding / catalytic activity / ATP binding / kinase activity / transferase activity / hydrolase activity / polynucleotide 3'-phosphatase activity / ATP-dependent polydeoxyribonucleotide 5'-hydroxyl-kinase activity | nucleus |
| 1448688_at | 0.81 | 0.47 | 0.57 | -2.09 | 0.07 | -6.21 | 0.08 | -5.98 | Podxl | podocalyxin-like | protein amino acid phosphorylation / leukocyte migration | protein kinase activity / ATP binding | plasma membrane / membrane / integral to membrane |
| 1427492_at | -1.25 | 2.24 | -0.21 | -5.77 | -0.47 | -4.15 | -0.11 | -5.97 | Pof1b | premature ovarian failure 1B | --- | actin binding | --- |
| 1439806_at | 0.69 | 3.48 | 0.58 | 1.76 | 0.11 | -5.75 | 0.31 | -2.67 | Pogk | Pogo transposable element with KRAB domain | regulation of transcription, DNA-dependent / regulation of transcription, DNA-dependent / multicellular organismal development / regulation of transcription | nucleic acid binding / nucleic acid binding / DNA binding | intracellular / intracellular / nucleus |
| 1419058_at | 0.31 | -4.69 | 0.10 | -6.07 | 0.77 | 0.48 | 0.54 | -1.93 | Polr1e | polymerase (RNA) I polypeptide E | transcription / rRNA transcription | DNA binding / DNA-directed RNA polymerase activity / protein binding | nucleus / nucleolus |
| 1429514_at | -0.64 | 3.44 | -0.62 | 2.99 | -0.45 | 0.18 | -0.57 | 2.15 | Ppap2b | phosphatidic acid phosphatase type 2B | blood vessel development / gastrulation with mouth forming second / phospholipid metabolic process / multicellular organismal development / regulation of Wnt receptor signaling pathway | catalytic activity / phosphatidate phosphatase activity / hydrolase activity / lipid phosphatase activity | endoplasmic reticulum / endoplasmic reticulum membrane / membrane / integral to membrane / integral to membrane |
| 1448908_at | -0.44 | -2.06 | -0.48 | -1.39 | -0.76 | 2.23 | -0.53 | -0.62 | Ppap2b | phosphatidic acid phosphatase type 2B | blood vessel development / gastrulation with mouth forming second / phospholipid metabolic process / multicellular organismal development / regulation of Wnt receptor signaling pathway | catalytic activity / phosphatidate phosphatase activity / hydrolase activity / lipid phosphatase activity | endoplasmic reticulum / endoplasmic reticulum membrane / membrane / integral to membrane / integral to membrane |
| 1428154_s_at | -0.60 | -2.07 | -0.70 | -0.91 | -0.79 | 0.04 | -0.42 | -3.66 | Ppapdc1 | phosphatidic acid phosphatase type 2 domain containing 1 | --- | catalytic activity / hydrolase activity | membrane / integral to membrane |
| 1460336_at | 0.13 | -6.19 | 0.42 | -3.90 | 0.73 | -0.77 | 1.15 | 2.96 | Ppargc1a | peroxisome proliferative activated receptor, gamma, coactivator 1 alpha | transcription / regulation of transcription, DNA-dependent / respiratory electron transport chain / positive regulation of transcription / positive regulation of transcription from RNA polymerase II promoter | nucleotide binding / nucleic acid binding / DNA binding / transcription coactivator activity / RNA binding / receptor activity / protein binding / transcription activator activity | nucleus / nucleus |
| 1418480_at | -0.61 | 2.12 | -0.30 | -3.04 | 0.01 | -6.31 | -0.03 | -6.05 | Ppbp | pro-platelet basic protein | immune response / immune response | cytokine activity / cytokine activity / chemokine activity / chemokine activity | extracellular region / extracellular region |
| 1449092_at | 0.00 | -6.45 | 0.13 | -5.36 | 0.63 | 3.03 | 0.22 | -3.90 | Ppm1d / LOC100045551 | protein phosphatase 1D magnesium-dependent, delta isoform / similar to Protein phosphatase 1D magnesium-dependent, delta | G2/M transition of mitotic cell cycle / protein amino acid dephosphorylation / cell cycle / response to bacterium | magnesium ion binding / catalytic activity / phosphoprotein phosphatase activity / protein serine/threonine phosphatase activity / magnesium-dependent protein serine/threonine phosphatase activity / hydrolase activity / manganese ion binding / metal ion binding | protein serine/threonine phosphatase complex |
| 1455737_at | -0.33 | -4.36 | 0.23 | -5.19 | 0.91 | 2.25 | 0.27 | -4.70 | Ppm1h | protein phosphatase 1H (PP2C domain containing) | protein amino acid dephosphorylation | catalytic activity / catalytic activity / phosphoprotein phosphatase activity / protein serine/threonine phosphatase activity / hydrolase activity | protein serine/threonine phosphatase complex |
| 1438012_at | 0.62 | 1.16 | 0.30 | -3.57 | 0.46 | -1.11 | 0.31 | -3.23 | Ppm1l | protein phosphatase 1 (formerly 2C)-like | MAPKKK cascade / protein amino acid dephosphorylation / transmembrane receptor protein serine/threonine kinase signaling pathway | magnesium ion binding / catalytic activity / phosphoprotein phosphatase activity / protein serine/threonine phosphatase activity / protein serine/threonine phosphatase activity / protein binding / hydrolase activity / manganese ion binding / metal ion binding | protein serine/threonine phosphatase complex / membrane / integral to membrane |
| 1440285_at | -1.01 | 0.60 | -0.49 | -3.86 | -0.80 | -1.16 | -0.62 | -2.64 | Ppp1r9a | protein phosphatase 1, regulatory (inhibitor) subunit 9A | actin filament organization / neurite development | protein binding / protein binding | cytoskeleton / filopodium / growth cone / cortical actin cytoskeleton / dendritic spine |
| 1432435_s_at | 0.33 | -3.36 | 0.34 | -3.11 | 0.78 | 3.06 | 0.51 | -0.59 | Ppp4r1l | protein phosphatase 4, regulatory subunit 1-like | --- | --- | --- |
| 1454236_a_at | 0.35 | -4.45 | 0.37 | -4.08 | 0.97 | 2.12 | 0.48 | -2.83 | Ppp4r1l | protein phosphatase 4, regulatory subunit 1-like | --- | --- | --- |
| 1424115_at | 0.02 | -6.44 | 0.11 | -5.89 | 0.61 | 0.58 | 0.39 | -2.36 | Ppp5c | protein phosphatase 5, catalytic subunit | protein amino acid dephosphorylation | phosphoprotein phosphatase activity / binding / iron ion binding / protein binding / hydrolase activity / protein domain specific binding / manganese ion binding / metal ion binding | nucleus / cytoplasm / cytosol |
| 1451242_a_at | 0.04 | -6.40 | 0.18 | -5.40 | 0.64 | 0.40 | 0.29 | -4.08 | Ppp5c | protein phosphatase 5, catalytic subunit | protein amino acid dephosphorylation | phosphoprotein phosphatase activity / binding / iron ion binding / protein binding / hydrolase activity / protein domain specific binding / manganese ion binding / metal ion binding | nucleus / cytoplasm / cytosol |
| 1426345_at | -0.71 | 0.64 | -0.36 | -3.63 | -0.02 | -6.30 | -0.28 | -4.33 | Prepl | prolyl endopeptidase-like | proteolysis | serine-type endopeptidase activity / prolyl oligopeptidase activity / peptidase activity / serine-type peptidase activity / hydrolase activity | cytoplasm |
| 1429463_at | 0.28 | -3.62 | 0.31 | -3.00 | 0.64 | 2.20 | 0.38 | -1.81 | Prkaa2 | protein kinase, AMP-activated, alpha 2 catalytic subunit | protein amino acid phosphorylation / fatty acid biosynthetic process / steroid biosynthetic process / cholesterol biosynthetic process / response to stress / lipid biosynthetic process / sterol biosynthetic process | nucleotide binding / magnesium ion binding / protein kinase activity / protein serine/threonine kinase activity / AMP-activated protein kinase activity / ATP binding / kinase activity / transferase activity / metal ion binding | nucleus |
| 1451140_s_at | -0.17 | -5.86 | -0.04 | -6.24 | 0.82 | 1.09 | 0.33 | -4.17 | Prkag2 | protein kinase, AMP-activated, gamma 2 non-catalytic subunit | fatty acid biosynthetic process / fatty acid biosynthetic process / cholesterol biosynthetic process / response to stress / lipid biosynthetic process | AMP-activated protein kinase activity / kinase activity | --- |
| 1434325_x_at | -1.22 | 2.25 | -0.62 | -2.74 | -0.26 | -5.53 | -0.49 | -3.68 | Prkar1b | protein kinase, cAMP dependent regulatory, type I beta | regulation of protein amino acid phosphorylation / protein amino acid phosphorylation / protein amino acid phosphorylation / signal transduction / learning and/or memory / cell proliferation / organ morphogenesis | nucleotide binding / cAMP-dependent protein kinase regulator activity / cAMP-dependent protein kinase regulator activity / kinase activity / cAMP binding | cytoplasm / cAMP-dependent protein kinase complex |
| 1450945_at | -0.85 | 1.70 | -0.36 | -3.85 | -0.64 | -0.63 | -0.84 | 1.55 | Prkca | protein kinase C, alpha | inactivation of MAPK activity / negative regulation of protein amino acid phosphorylation / positive regulation of protein amino acid phosphorylation / regulation of the force of heart contraction / chondrocyte differentiation / protein amino acid phosphorylation / protein amino acid phosphorylation / negative regulation of protein kinase activity / cellular calcium ion homeostasis / regulation of muscle contraction / intracellular signaling cascade / induction of apoptosis by intracellular signals / neutrophil chemotaxis / negative regulation of glucose import / negative regulation of insulin receptor signaling pathway / positive regulation of inflammatory response / regulation of peptidyl-tyrosine phosphorylation / induction of positive chemotaxis | nucleotide binding / protein kinase activity / protein serine/threonine kinase activity / protein serine/threonine kinase activity / protein kinase C activity / protein kinase C activity / calcium-dependent protein kinase C activity / calcium ion binding / protein binding / ATP binding / zinc ion binding / kinase activity / transferase activity / diacylglycerol binding / metal ion binding | nucleus / cytoplasm / mitochondrion |
| 1435162_at | -0.41 | -1.03 | -0.70 | 3.66 | -0.92 | 6.27 | -0.66 | 2.99 | Prkg2 | protein kinase, cGMP-dependent, type II | regulation of protein amino acid phosphorylation / protein amino acid phosphorylation | nucleotide binding / protein kinase activity / protein serine/threonine kinase activity / cGMP-dependent protein kinase activity / ATP binding / cAMP-dependent protein kinase regulator activity / kinase activity / transferase activity / cGMP binding | cAMP-dependent protein kinase complex |
| 1435460_at | -0.17 | -5.68 | -0.45 | -2.38 | -0.66 | 0.38 | -0.64 | 0.16 | Prkg2 | protein kinase, cGMP-dependent, type II | regulation of protein amino acid phosphorylation / protein amino acid phosphorylation | nucleotide binding / protein kinase activity / protein serine/threonine kinase activity / cGMP-dependent protein kinase activity / ATP binding / cAMP-dependent protein kinase regulator activity / kinase activity / transferase activity / cGMP binding | cAMP-dependent protein kinase complex |
| 1418845_at | -0.93 | 1.68 | -0.84 | 0.86 | -1.12 | 3.40 | -0.93 | 1.63 | Proc | protein C | proteolysis / blood coagulation / negative regulation of apoptosis / negative regulation of apoptosis / negative regulation of apoptosis | protein C (activated) activity / catalytic activity / serine-type endopeptidase activity / calcium ion binding / protein binding / peptidase activity / hydrolase activity | extracellular region / extracellular space / extracellular space |
| 1420664_s_at | 0.33 | -2.28 | 0.64 | 3.08 | 0.37 | -1.38 | 0.31 | -2.31 | Procr | protein C receptor, endothelial | immune response / blood coagulation / antigen processing and presentation / negative regulation of coagulation | receptor activity / protein C-terminus binding | centrosome / membrane / integral to membrane / integral to membrane |
| 1431109_at | -0.77 | 4.26 | -0.61 | 2.01 | -0.45 | -0.47 | -0.34 | -2.27 | Prr16 | proline rich 16 | --- | --- | --- |
| 1439922_at | -0.13 | -5.92 | -0.19 | -5.28 | -0.67 | 0.93 | -0.85 | 2.90 | Prrc1 | proline-rich coiled-coil 1 | --- | identical protein binding | Golgi apparatus |
| 1421638_at | -0.52 | -3.73 | -0.38 | -4.75 | -1.10 | 1.28 | -0.54 | -3.31 | Psg17 | pregnancy specific glycoprotein 17 | immune response / female pregnancy / female pregnancy | protein binding | extracellular space |
| 1417166_at | -0.63 | 2.12 | -0.42 | -1.05 | -0.11 | -5.75 | -0.10 | -5.69 | Psip1 | PC4 and SFRS1 interacting protein 1 | transcription / regulation of transcription, DNA-dependent | DNA binding | nucleus |
| 1420467_at | 0.55 | -3.94 | -0.55 | -3.78 | -1.05 | 0.15 | -0.90 | -0.98 | Psors1c2 | psoriasis susceptibility 1 candidate 2 (human) | --- | --- | extracellular region |
| 1415673_at | 0.74 | -0.54 | 1.07 | 2.71 | 0.92 | 1.33 | 0.64 | -1.43 | Psph | phosphoserine phosphatase | L-serine biosynthetic process / L-serine biosynthetic process / metabolic process / metabolic process / amino acid biosynthetic process | magnesium ion binding / catalytic activity / phosphoserine phosphatase activity / phosphoserine phosphatase activity / hydrolase activity / phosphoric monoester hydrolase activity | --- |
| 1450655_at | -0.56 | -0.14 | -0.33 | -3.34 | -0.26 | -4.32 | -0.60 | 0.50 | Pten | phosphatase and tensin homolog | angiogenesis / protein amino acid dephosphorylation / protein amino acid dephosphorylation / induction of apoptosis / cell cycle / central nervous system development / heart development / negative regulation of cell proliferation / negative regulation of cell proliferation / negative regulation of cell proliferation / negative regulation of cell proliferation / dephosphorylation / cell migration / negative regulation of cell migration / negative regulation of cell migration / neurite development / regulation of protein stability / regulation of protein stability / negative regulation of apoptosis / endothelial cell migration / negative regulation of cell cycle / inositol phosphate dephosphorylation / inositol phosphate dephosphorylation / phosphoinositide dephosphorylation / phosphoinositide dephosphorylation / platelet-derived growth factor receptor signaling pathway / cardiac muscle development / negative regulation of focal adhesion formation / negative regulation of focal adhesion formation / negative regulation of protein kinase B signaling cascade / negative regulation of protein kinase B signaling cascade | phosphatidylinositol-3-phosphatase activity / phosphatidylinositol-3-phosphatase activity / protein serine/threonine phosphatase activity / protein serine/threonine phosphatase activity / protein tyrosine phosphatase activity / platelet-derived growth factor receptor binding / protein binding / protein binding / protein tyrosine/serine/threonine phosphatase activity / phosphatidylinositol-3,4,5-trisphosphate 3-phosphatase activity / phosphatidylinositol-3,4,5-trisphosphate 3-phosphatase activity / phosphatidylinositol-3,4,5-trisphosphate 3-phosphatase activity / hydrolase activity / phosphoric monoester hydrolase activity / PDZ domain binding / PDZ domain binding / inositol-1,3,4,5-tetrakisphosphate 3-phosphatase activity / inositol-1,3,4,5-tetrakisphosphate 3-phosphatase activity / phosphatidylinositol-3,4-bisphosphate 3-phosphatase activity / phosphatidylinositol-3,4-bisphosphate 3-phosphatase activity | cytoplasm / cytoplasm |
| 1417591_at | 0.06 | -6.26 | 0.22 | -4.31 | 0.67 | 3.10 | 0.59 | 1.87 | Ptges2 | prostaglandin E synthase 2 | prostaglandin biosynthetic process / fatty acid biosynthetic process / lipid biosynthetic process / regulation of transcription / cell redox homeostasis / secretion | DNA binding / electron carrier activity / protein disulfide oxidoreductase activity / transcription activator activity / isomerase activity / prostaglandin-E synthase activity | Golgi membrane / extracellular space / nucleus / nucleus / cytoplasm / Golgi apparatus / cytosol / membrane / integral to membrane |
| 1450967_at | -0.93 | 1.02 | -0.55 | -2.65 | -0.71 | -1.09 | -0.41 | -3.90 | Ptplad2 | protein tyrosine phosphatase-like A domain containing 2 | --- | --- | membrane / integral to membrane / integral to membrane |
| 1421499_a_at | 1.37 | 3.01 | 1.34 | 2.83 | 1.47 | 3.67 | 1.37 | 2.88 | Ptpn14 | protein tyrosine phosphatase, non-receptor type 14 | protein amino acid dephosphorylation / dephosphorylation | phosphoprotein phosphatase activity / protein tyrosine phosphatase activity / receptor activity / structural molecule activity / binding / hydrolase activity / phosphoric monoester hydrolase activity | cytoplasm / cytoskeleton |
| 1455359_at | 0.98 | 4.34 | 1.06 | 4.95 | 0.76 | 1.85 | 0.47 | -1.79 | Ptpn14 | protein tyrosine phosphatase, non-receptor type 14 | protein amino acid dephosphorylation / dephosphorylation | phosphoprotein phosphatase activity / protein tyrosine phosphatase activity / receptor activity / structural molecule activity / binding / hydrolase activity / phosphoric monoester hydrolase activity | cytoplasm / cytoskeleton |
| 1418540_a_at | 0.01 | -6.45 | -0.26 | -4.56 | -0.67 | 0.72 | -0.72 | 1.28 | Ptpre | protein tyrosine phosphatase, receptor type, E | protein amino acid phosphorylation / protein amino acid dephosphorylation / transmembrane receptor protein tyrosine phosphatase signaling pathway / transmembrane receptor protein tyrosine phosphatase signaling pathway / dephosphorylation | phosphoprotein phosphatase activity / protein tyrosine phosphatase activity / protein tyrosine phosphatase activity / receptor activity / hydrolase activity / phosphoric monoester hydrolase activity / protein homodimerization activity | extracellular space / plasma membrane / membrane / integral to membrane / integral to membrane |
| 1456070_at | -0.53 | 1.35 | -0.60 | 2.41 | -0.26 | -3.42 | -0.40 | -0.93 | Ptprg | protein tyrosine phosphatase, receptor type, G | protein amino acid dephosphorylation / one-carbon compound metabolic process / transmembrane receptor protein tyrosine phosphatase signaling pathway / dephosphorylation | carbonate dehydratase activity / phosphoprotein phosphatase activity / protein tyrosine phosphatase activity / receptor activity / zinc ion binding / hydrolase activity / phosphoric monoester hydrolase activity | extracellular space / plasma membrane / membrane / integral to membrane / integral to membrane |
| 1428254_at | 0.82 | 1.55 | 0.36 | -3.80 | 0.48 | -2.43 | -0.06 | -6.02 | Purb | purine rich element binding protein B | DNA unwinding during replication / transcription / regulation of transcription, DNA-dependent / apoptosis / cell proliferation / cell differentiation / negative regulation of transcription, DNA-dependent | translation repressor activity, nucleic acid binding / DNA binding / double-stranded DNA binding / double-stranded telomeric DNA binding / single-stranded DNA binding / transcription factor activity / mRNA binding / protein binding / transcription factor binding / specific transcriptional repressor activity / purine-rich negative regulatory element binding / SMAD binding | nucleus / nucleus / nucleus / DNA replication factor A complex |
| 1452324_at | 0.60 | 0.94 | 0.40 | -1.98 | 0.18 | -5.15 | 0.17 | -5.11 | Pvt1 | plasmacytoma variant translocation 1 | nucleosome assembly | DNA binding | nucleosome / nucleus / chromosome |
| 1451253_at | -0.79 | 2.57 | -0.31 | -3.81 | -0.34 | -3.47 | -0.19 | -5.05 | Pxk | PX domain containing serine/threonine kinase | protein amino acid phosphorylation / protein amino acid phosphorylation / cell communication / intracellular signaling cascade / regulation of synaptic transmission | actin binding / protein kinase activity / protein kinase activity / protein binding / ATP binding / ATP binding / kinase activity / phosphoinositide binding | cytoplasm / cytoplasm / cytoplasm / plasma membrane / plasma membrane / membrane |
| 1424556_at | 0.71 | 3.96 | 1.23 | 9.09 | 0.72 | 4.07 | 0.14 | -5.12 | Pycr1 | pyrroline-5-carboxylate reductase 1 | proline biosynthetic process / metabolic process / amino acid biosynthetic process / oxidation reduction | catalytic activity / pyrroline-5-carboxylate reductase activity / binding / oxidoreductase activity | mitochondrion / mitochondrion |
| 1427151_at | -0.29 | -5.07 | -0.84 | 0.61 | -0.10 | -6.12 | -0.15 | -5.70 | Qser1 | glutamine and serine rich 1 | --- | --- | --- |
| 1426452_a_at | 0.88 | 0.81 | 0.39 | -4.05 | 0.30 | -4.90 | 0.82 | 0.30 | Rab30 | RAB30, member RAS oncogene family | small GTPase mediated signal transduction / protein transport | nucleotide binding / GTP binding | plasma membrane / membrane |
| 1416527_at | 0.74 | 2.84 | 0.08 | -6.07 | 0.18 | -5.20 | 0.09 | -5.81 | Rab32 | RAB32, member RAS oncogene family | small GTPase mediated signal transduction / protein transport | nucleotide binding / GTP binding | mitochondrion |
| 1418341_at | -0.60 | 1.44 | -0.35 | -2.50 | -0.11 | -5.84 | -0.23 | -4.26 | Rab4a | RAB4A, member RAS oncogene family | transport / small GTPase mediated signal transduction / protein transport / protein transport / regulation of endocytosis | nucleotide binding / protein binding / GTP binding / protein transporter activity | cytoplasm / endosome / membrane |
| 1418404_at | 0.63 | 2.59 | 0.65 | 2.85 | 0.73 | 3.97 | 0.42 | -0.79 | Rad9 | RAD9 homolog (S. pombe) | cell cycle checkpoint / DNA damage checkpoint / DNA repair / response to DNA damage stimulus / response to radiation / positive regulation of apoptosis | nuclease activity / exonuclease activity / protein binding / protein binding / 3'-5' exonuclease activity / exodeoxyribonuclease III activity / hydrolase activity / SH3 domain binding / protein kinase binding / histone deacetylase binding | nucleus / nucleus / cytoplasm |
| 1415850_at | -0.92 | 3.65 | -0.33 | -3.63 | -0.37 | -3.21 | -0.23 | -4.78 | Rasa3 | RAS p21 protein activator 3 | signal transduction / intracellular signaling cascade / regulation of small GTPase mediated signal transduction | GTPase activator activity / zinc ion binding / metal ion binding | intracellular |
| 1454742_at | -1.04 | 4.37 | -1.07 | 4.49 | -0.47 | -2.20 | -0.60 | -0.46 | Rasgef1b / LOC100044232 | RasGEF domain family, member 1B / hypothetical protein LOC100044232 | small GTPase mediated signal transduction / regulation of small GTPase mediated signal transduction | guanyl-nucleotide exchange factor activity | intracellular |
| 1438030_at | -0.99 | 2.24 | -0.87 | 1.10 | -0.48 | -3.00 | -0.44 | -3.31 | Rasgrp3 | RAS, guanyl releasing protein 3 | intracellular signaling cascade / small GTPase mediated signal transduction / Ras protein signal transduction / regulation of GTPase activity / regulation of small GTPase mediated signal transduction | guanyl-nucleotide exchange factor activity / Ras guanyl-nucleotide exchange factor activity / calcium ion binding / zinc ion binding / Ras GTPase binding / diacylglycerol binding / metal ion binding | intracellular / cytoplasm |
| 1427942_at | -1.32 | 3.09 | -0.83 | -0.86 | -0.28 | -5.36 | -0.40 | -4.41 | Rassf9 | Ras association (RalGDS/AF-6) domain family (N-terminal) member 9 | signal transduction / neuropeptide signaling pathway | protein binding / protein binding | endosome / endosome / cytosol / cytosol / trans-Golgi network transport vesicle membrane / trans-Golgi network transport vesicle membrane |
| 1451321_a_at | 0.34 | -2.52 | 0.20 | -4.61 | 0.60 | 1.86 | 0.32 | -2.64 | Rbm43 | RNA binding motif protein 43 | --- | nucleic acid binding / RNA binding | --- |
| 1438069_a_at | -0.39 | -4.90 | -0.82 | -1.23 | -1.00 | 0.17 | 0.27 | -5.35 | Rbm5 | RNA binding motif protein 5 | cell cycle / negative regulation of cell cycle | nucleotide binding / nucleic acid binding / RNA binding / zinc ion binding / metal ion binding | intracellular / nucleus |
| 1418114_at | -0.46 | -1.46 | -0.70 | 1.92 | -0.37 | -2.70 | -0.49 | -0.79 | Rbpj | recombination signal binding protein for immunoglobulin kappa J region | angiogenesis / epithelial to mesenchymal transition / transcription / regulation of transcription, DNA-dependent / regulation of transcription from RNA polymerase II promoter / Notch signaling pathway / Notch signaling pathway / heart development / positive regulation of cell proliferation / negative regulation of cell proliferation / epidermal cell fate specification / pituitary gland development / hemopoiesis / B cell differentiation / keratinocyte differentiation / defense response to bacterium / cell fate commitment / regulation of transcription / negative regulation of cell differentiation / regulation of timing of cell differentiation / sebaceous gland development / hair follicle maturation | DNA binding / DNA binding / chromatin binding / transcription factor activity / protein binding / protein binding / transcription factor binding / transcription activator activity / sequence-specific DNA binding | nucleus / nucleus / nucleus |
| 1454896_at | -0.38 | -3.09 | -0.73 | 1.55 | -0.58 | -0.32 | -0.62 | 0.20 | Rbpj | recombination signal binding protein for immunoglobulin kappa J region | angiogenesis / epithelial to mesenchymal transition / transcription / regulation of transcription, DNA-dependent / regulation of transcription from RNA polymerase II promoter / Notch signaling pathway / Notch signaling pathway / heart development / positive regulation of cell proliferation / negative regulation of cell proliferation / epidermal cell fate specification / pituitary gland development / hemopoiesis / B cell differentiation / keratinocyte differentiation / defense response to bacterium / cell fate commitment / regulation of transcription / negative regulation of cell differentiation / regulation of timing of cell differentiation / sebaceous gland development / hair follicle maturation | DNA binding / DNA binding / chromatin binding / transcription factor activity / protein binding / protein binding / transcription factor binding / transcription activator activity / sequence-specific DNA binding | nucleus / nucleus / nucleus |
| 1450243_a_at | -0.78 | 1.87 | -0.53 | -1.16 | 0.15 | -5.70 | -0.03 | -6.06 | Rcan2 | regulator of calcineurin 2 | calcium-mediated signaling | --- | --- |
| 1418760_at | -0.70 | 1.06 | -0.69 | 0.97 | -0.55 | -0.79 | -0.26 | -4.40 | Rdh11 | retinol dehydrogenase 11 | metabolic process / metabolic process / retinol metabolic process | catalytic activity / alcohol dehydrogenase activity / retinol dehydrogenase activity / binding / oxidoreductase activity / oxidoreductase activity | extracellular space / intracellular / endoplasmic reticulum / endoplasmic reticulum membrane / membrane / integral to membrane |
| 1422922_at | 0.31 | -4.15 | 0.46 | -2.12 | 0.63 | 0.20 | 0.48 | -1.76 | Recql4 | RecQ protein-like 4 | skeletal development / DNA recombination / positive regulation of cell proliferation / pigmentation / negative regulation of sister chromatid cohesion / skeletal morphogenesis | nucleotide binding / nucleic acid binding / helicase activity / ATP binding / ATP-dependent helicase activity / zinc ion binding / hydrolase activity / metal ion binding | nucleus / cytoplasm |
| 1433509_s_at | -0.71 | 1.19 | -0.26 | -4.64 | -0.45 | -2.12 | -0.45 | -2.11 | Reep1 | receptor accessory protein 1 | protein insertion into membrane | olfactory receptor binding | cytoplasm / mitochondrion / membrane / integral to membrane / mitochondrial membrane |
| 1430128_a_at | 0.96 | 2.25 | 1.30 | 5.06 | 1.55 | 6.84 | 0.94 | 2.00 | Reep6 | receptor accessory protein 6 | --- | protein binding | membrane / integral to membrane / integral to membrane |
| 1420710_at | 1.33 | 1.91 | 0.49 | -4.27 | 0.30 | -5.47 | -0.07 | -6.05 | Rel | reticuloendotheliosis oncogene | cytokine production / transcription / regulation of transcription, DNA-dependent / positive regulation of interleukin-12 biosynthetic process / regulation of transcription / positive regulation of transcription, DNA-dependent | DNA binding / transcription factor activity / transcription factor activity / protein binding | nucleus / nucleus / nucleus |
| 1417856_at | 0.61 | 1.09 | 0.02 | -6.27 | -0.10 | -5.94 | -0.03 | -6.05 | Relb | avian reticuloendotheliosis viral (v-rel) oncogene related B | transcription / regulation of transcription, DNA-dependent / cellular process / antigen processing and presentation / T-helper 1 type immune response / myeloid dendritic cell differentiation / T-helper 1 cell differentiation / regulation of transcription / regulation of cell cycle | DNA binding / transcription factor activity | intracellular / nucleus |
| 1424716_at | -0.39 | -2.66 | -0.12 | -5.83 | 0.61 | 0.56 | 0.17 | -5.25 | Retsat | retinol saturase (all trans retinol 13,14 reductase) | carotenoid biosynthetic process / retinol metabolic process / oxidation reduction | electron carrier activity / oxidoreductase activity / oxidoreductase activity / oxidoreductase activity, acting on paired donors, with incorporation or reduction of molecular oxygen / FAD binding / all-trans-retinol 13,14-reductase activity / all-trans-retinol 13,14-reductase activity | nuclear outer membrane / endoplasmic reticulum / endoplasmic reticulum membrane / endoplasmic reticulum membrane / membrane / nuclear membrane |
| 1419458_at | -0.49 | -3.13 | -0.87 | 0.91 | -0.65 | -1.32 | -0.27 | -4.93 | Rgnef | Rho-guanine nucleotide exchange factor | intracellular signaling cascade / central nervous system neuron axonogenesis / cell differentiation / regulation of Rho protein signal transduction / neurofilament cytoskeleton organization and biogenesis | RNA binding / guanyl-nucleotide exchange factor activity / Rho guanyl-nucleotide exchange factor activity / protein binding / zinc ion binding / diacylglycerol binding / metal ion binding | intracellular / cytoplasm / plasma membrane / membrane |
| 1449110_at | 0.28 | -4.98 | 0.06 | -6.22 | 0.74 | 0.16 | 0.26 | -4.86 | Rhob | ras homolog gene family, member B | angiogenesis / transport / apoptosis / transformed cell apoptosis / cell cycle / cell adhesion / cell adhesion / small GTPase mediated signal transduction / Rho protein signal transduction / multicellular organismal development / endosome to lysosome transport / endosome to lysosome transport / cellular process / protein transport / cell differentiation / positive regulation of angiogenesis / negative regulation of cell cycle / negative regulation of cell cycle | nucleotide binding / GTPase activity / protein binding / protein binding / GTP binding / GTP binding | intracellular / nucleus / nucleus / endosome / plasma membrane / plasma membrane / plasma membrane / endosome membrane / membrane / late endosome membrane |
| 1426604_at | -0.83 | 1.90 | -0.59 | -0.89 | -0.15 | -5.77 | -0.19 | -5.32 | Rnasel | ribonuclease L (2', 5'-oligoisoadenylate synthetase-dependent) | mRNA processing / protein amino acid phosphorylation | nucleotide binding / RNA binding / nuclease activity / endonuclease activity / protein kinase activity / ATP binding / zinc ion binding / hydrolase activity / endoribonuclease activity, producing 5'-phosphomonoesters / metal ion binding | cytoplasm / mitochondrion |
| 1455197_at | 0.71 | 0.30 | -0.11 | -6.00 | 0.12 | -5.97 | 0.10 | -5.85 | Rnd1 | Rho family GTPase 1 | actin filament organization / negative regulation of cell adhesion / small GTPase mediated signal transduction / small GTPase mediated signal transduction / neuron remodeling | nucleotide binding / GTP binding / GTP binding | intracellular / cytoplasm / cytoskeleton / plasma membrane / adherens junction / membrane |
| 1427231_at | -0.89 | 1.64 | -0.68 | -0.51 | -0.39 | -3.83 | -0.29 | -4.62 | Robo1 | roundabout homolog 1 (Drosophila) | chemotaxis / multicellular organismal development / nervous system development / axon guidance / axon guidance / cell differentiation | receptor activity / protein binding / protein heterodimerization activity | extracellular space / membrane / integral to membrane / axolemma |
| 1434061_at | -0.61 | 1.34 | -0.41 | -1.60 | -0.34 | -2.70 | -0.11 | -5.63 | Rp2h | retinitis pigmentosa 2 homolog (human) | GTP biosynthetic process / GTP biosynthetic process / UTP biosynthetic process / UTP biosynthetic process / CTP biosynthetic process / CTP biosynthetic process / protein amino acid prenylation | nucleoside diphosphate kinase activity / nucleoside diphosphate kinase activity / binding / ATP binding / ATP binding / protein prenyltransferase activity | plasma membrane / membrane / membrane |
| 1439780_at | 0.44 | -2.59 | 0.45 | -2.38 | 0.71 | 0.88 | 0.18 | -5.30 | Rpl7l1 | ribosomal protein L7-like 1 | translation / translation | structural constituent of ribosome / structural constituent of ribosome / transcription regulator activity / transcription regulator activity | intracellular / intracellular / ribosome / ribosome / large ribosomal subunit / large ribosomal subunit / ribonucleoprotein complex |
| 1419460_at | 0.25 | -5.19 | 0.13 | -5.90 | 0.76 | 0.72 | 0.25 | -4.84 | Rpp14 / LOC100044172 | ribonuclease P 14 subunit (human) / hypothetical protein LOC100044172 | tRNA processing / tRNA processing | RNA binding / ribonuclease P activity / ribonuclease P activity / ribonuclease activity / hydrolase activity / hydrolase activity / identical protein binding | nucleus / nucleus |
| 1424792_at | 0.34 | -5.36 | 0.60 | -3.37 | 1.26 | 1.77 | 0.83 | -1.42 | Rpp40 | ribonuclease P 40 subunit (human) | tRNA 5'-leader removal | ribonuclease P activity / ribonuclease P activity | nucleolar ribonuclease P complex |
| 1443750_s_at | 0.46 | -4.52 | 0.59 | -3.37 | 1.15 | 1.01 | 0.71 | -2.35 | Rpp40 | ribonuclease P 40 subunit (human) | tRNA 5'-leader removal | ribonuclease P activity / ribonuclease P activity | nucleolar ribonuclease P complex |
| 1430978_at | 0.41 | -3.01 | 0.28 | -4.51 | 0.42 | -2.75 | 0.66 | 0.30 | Rps25 | ribosomal protein S25 | --- | structural constituent of ribosome / protein binding | ribosome / ribonucleoprotein complex |
| 1452767_at | 0.08 | -6.36 | -0.21 | -5.80 | -1.04 | 0.69 | -0.40 | -4.48 | Rrbp1 | ribosome binding protein 1 | transport / signal transduction / protein transport / intracellular protein transport across a membrane | receptor activity | endoplasmic reticulum / endoplasmic reticulum membrane / membrane / integral to membrane / integral to membrane / integral to endoplasmic reticulum membrane / integral to endoplasmic reticulum membrane |
| 1436058_at | 0.89 | 5.57 | 0.82 | 4.62 | 0.86 | 5.10 | 0.34 | -2.38 | Rsad2 | radical S-adenosyl methionine domain containing 2 | response to virus / defense response to virus / defense response to virus | catalytic activity / catalytic activity / iron ion binding / metal ion binding / iron-sulfur cluster binding | extracellular space / endoplasmic reticulum / endoplasmic reticulum |
| 1443859_at | 0.40 | -1.97 | 0.71 | 2.73 | -0.02 | -6.29 | 0.05 | -5.99 | Rsbn1 / LOC100045795 | rosbin, round spermatid basic protein 1 / similar to mKIAA3002 protein | --- | protein binding | nucleus / nucleus |
| 1434456_at | -0.14 | -5.96 | -0.61 | -0.42 | -0.57 | -0.94 | -0.64 | 0.03 | Rundc3b | RUN domain containing 3B | --- | --- | --- |
| 1424542_at | 0.15 | -6.05 | 0.54 | -2.42 | 0.87 | 1.04 | 0.67 | -0.91 | S100a4 | S100 calcium binding protein A4 | --- | calcium ion binding / calcium ion binding / protein binding / identical protein binding | --- |
| 1460603_at | -0.34 | -4.36 | -0.84 | 1.44 | -0.62 | -1.05 | -0.34 | -3.99 | Samd9l | sterile alpha motif domain containing 9-like | --- | --- | --- |
| 1426257_a_at | 0.31 | -3.89 | 0.55 | -0.42 | 0.59 | 0.10 | 0.40 | -2.40 | Sars | seryl-aminoacyl-tRNA synthetase | translation / tRNA aminoacylation for protein translation / seryl-tRNA aminoacylation | nucleotide binding / aminoacyl-tRNA ligase activity / serine-tRNA ligase activity / ATP binding / ligase activity | cytoplasm |
| 1452000_s_at | 0.39 | -1.62 | 0.49 | 0.06 | 0.62 | 1.98 | 0.44 | -0.72 | Sars | seryl-aminoacyl-tRNA synthetase | translation / tRNA aminoacylation for protein translation / seryl-tRNA aminoacylation | nucleotide binding / aminoacyl-tRNA ligase activity / serine-tRNA ligase activity / ATP binding / ligase activity | cytoplasm |
| 1420502_at | 0.71 | 2.02 | 0.56 | 0.03 | 0.44 | -1.74 | 0.37 | -2.63 | Sat1 | spermidine/spermine N1-acetyl transferase 1 | polyamine metabolic process / metabolic process / spermine catabolic process | diamine N-acetyltransferase activity / N-acetyltransferase activity / acyltransferase activity / transferase activity / spermidine binding | soluble fraction / cytoplasm |
| 1454704_at | -0.08 | -6.25 | 0.28 | -4.41 | 0.64 | 0.35 | 0.41 | -2.64 | Scarb2 | scavenger receptor class B, member 2 | cell adhesion / cell adhesion | receptor activity / receptor activity / protein binding | lysosome / lysosome / lysosomal membrane / plasma membrane / membrane / membrane / integral to membrane |
| 1460235_at | -0.20 | -5.25 | 0.23 | -4.84 | 0.53 | -0.83 | 0.70 | 1.43 | Scarb2 | scavenger receptor class B, member 2 | cell adhesion / cell adhesion | receptor activity / receptor activity / protein binding | lysosome / lysosome / lysosomal membrane / plasma membrane / membrane / membrane / integral to membrane |
| 1415822_at | -0.92 | 2.39 | -0.73 | 0.39 | -1.08 | 3.93 | -0.51 | -2.00 | Scd2 | stearoyl-Coenzyme A desaturase 2 | lipid metabolic process / fatty acid biosynthetic process / lipid biosynthetic process / myelination | stearoyl-CoA 9-desaturase activity / iron ion binding / oxidoreductase activity / oxidoreductase activity, acting on paired donors, with oxidation of a pair of donors resulting in the reduction of molecular oxygen to two molecules of water | endoplasmic reticulum / endoplasmic reticulum / endoplasmic reticulum membrane / membrane / integral to membrane |
| 1415823_at | -1.46 | 2.39 | -1.47 | 2.39 | -1.33 | 1.51 | -0.92 | -1.28 | Scd2 | stearoyl-Coenzyme A desaturase 2 | lipid metabolic process / fatty acid biosynthetic process / lipid biosynthetic process / myelination | stearoyl-CoA 9-desaturase activity / iron ion binding / oxidoreductase activity / oxidoreductase activity, acting on paired donors, with oxidation of a pair of donors resulting in the reduction of molecular oxygen to two molecules of water | endoplasmic reticulum / endoplasmic reticulum / endoplasmic reticulum membrane / membrane / integral to membrane |
| 1450120_at | -0.30 | -4.19 | -0.14 | -5.71 | -0.23 | -4.90 | -0.62 | 0.26 | Scn1a | sodium channel, voltage-gated, type I, alpha | regulation of action potential / transport / ion transport / ion transport / cation transport / potassium ion transport / sodium ion transport / sodium ion transport / sodium ion transport / adult walking behavior / action potential propagation / regulation of action potential in neuron / regulation of membrane potential / neuromuscular process controlling posture | ion channel activity / ion channel activity / voltage-gated ion channel activity / voltage-gated sodium channel activity / voltage-gated sodium channel activity / voltage-gated sodium channel activity / voltage-gated potassium channel activity / cation channel activity / sodium channel activity | voltage-gated sodium channel complex / membrane fraction / voltage-gated potassium channel complex / intercalated disc / membrane / membrane / integral to membrane / T-tubule / axon / node of Ranvier / cell soma / initial segment |
| 1449686_s_at | -0.60 | 0.02 | -0.72 | 1.55 | -0.38 | -3.03 | -0.33 | -3.56 | Scp2 | sterol carrier protein 2, liver | acyl-CoA metabolic process / transport / lipid transport / peroxisome organization and biogenesis / metabolic process | catalytic activity / sterol carrier protein X-related thiolase activity / sterol carrier activity / protein binding / lipid binding / oxidoreductase activity / transferase activity / propanoyl-CoA C-acyltransferase activity | cytoplasm / mitochondrion / mitochondrion / peroxisome / peroxisome |
| 1426917_s_at | -0.69 | 1.87 | -0.22 | -4.76 | 0.06 | -6.19 | -0.07 | -5.95 | Scrn3 | secernin 3 | proteolysis | dipeptidase activity | --- |
| 1436002_at | 0.00 | -6.45 | -0.25 | -4.93 | -0.70 | 0.17 | -0.18 | -5.39 | Scube3 | signal peptide, CUB domain, EGF-like 3 | protein homooligomerization / protein heterooligomerization | calcium ion binding | extracellular region / cell surface |
| 1424090_at | 1.33 | 4.31 | 1.13 | 2.69 | 0.92 | 0.86 | 0.60 | -2.10 | Sdcbp2 | syndecan binding protein (syntenin) 2 | --- | protein binding / protein C-terminus binding / protein homodimerization activity / protein heterodimerization activity | cytoplasm |
| 1423347_at | -0.46 | -0.77 | -0.46 | -0.73 | -0.52 | 0.29 | -0.67 | 2.30 | Sec23a | SEC23A (S. cerevisiae) | transport / intracellular protein transport / intracellular protein transport / ER to Golgi vesicle-mediated transport / protein transport / vesicle-mediated transport | protein binding / zinc ion binding | Golgi membrane / endoplasmic reticulum / Golgi apparatus / membrane / COPII vesicle coat / smooth endoplasmic reticulum membrane / perinuclear region of cytoplasm |
| 1426972_at | -0.38 | -4.30 | -0.65 | -1.43 | -0.84 | 0.54 | -0.74 | -0.44 | Sec24d | SEC24 related gene family, member D (S. cerevisiae) | transport / intracellular protein transport / ER to Golgi vesicle-mediated transport / protein transport / vesicle-mediated transport | protein binding / zinc ion binding | Golgi apparatus / COPII vesicle coat |
| 1417580_s_at | -0.84 | -5.23 | -1.27 | -3.82 | -2.70 | 0.92 | -1.56 | -2.76 | Selenbp1 / LOC100044204 | selenium binding protein 1 / hypothetical protein LOC100044204 | transport / protein transport | selenium binding / selenium binding | nucleus / cytoplasm / membrane |
| 1425840_a_at | -0.29 | -5.01 | -0.09 | -6.13 | -0.78 | 0.32 | -0.48 | -2.77 | Sema3f | sema domain, immunoglobulin domain (Ig), short basic domain, secreted, (semaphorin) 3F | neural crest cell migration / multicellular organismal development / negative regulation of axon extension involved in axon guidance / negative chemotaxis / negative chemotaxis | receptor activity / chemorepellent activity | extracellular region / extracellular space / membrane |
| 1435361_at | -0.12 | -6.07 | -0.34 | -3.79 | -0.86 | 2.35 | -0.81 | 1.78 | Sema3g | sema domain, immunoglobulin domain (Ig), short basic domain, secreted, (semaphorin) 3G | multicellular organismal development | receptor activity | extracellular region / membrane |
| 1436860_at | -0.63 | 0.11 | -0.04 | -6.24 | -0.17 | -5.55 | 0.46 | -1.94 | Senp7 | SUMO1/sentrin specific peptidase 7 | proteolysis / ubiquitin cycle | peptidase activity / cysteine-type peptidase activity / hydrolase activity | --- |
| 1433571_at | 0.03 | -6.43 | -0.60 | -0.37 | -0.93 | 3.38 | -0.73 | 1.18 | Serinc5 | serine incorporator 5 | lipid metabolic process / phosphatidylserine metabolic process / phospholipid biosynthetic process / myelination / positive regulation of transferase activity | electron carrier activity / electron carrier activity | endoplasmic reticulum / Golgi apparatus / membrane / integral to membrane / myelin sheath |
| 1424758_s_at | -0.59 | 0.09 | -0.82 | 3.11 | -0.67 | 1.29 | -0.24 | -4.43 | Serpina10 | serine (or cysteine) peptidase inhibitor, clade A (alpha-1 antiproteinase, antitrypsin), member 10 | --- | endopeptidase inhibitor activity / serine-type endopeptidase inhibitor activity | extracellular region |
| 1422668_at | 0.22 | -4.98 | 0.67 | 1.48 | 0.75 | 2.41 | 0.56 | 0.04 | Serpinb9b | serine (or cysteine) peptidase inhibitor, clade B, member 9b | cytolysis | serine-type endopeptidase inhibitor activity / protein binding / peptidase activity | --- |
| 1418423_s_at | 0.19 | -5.43 | 0.66 | 0.76 | 0.42 | -2.52 | 0.50 | -1.28 | Serpinb9f / Serpinb9e / Serpinb9g / OTTMUSG00000000724 | serine (or cysteine) peptidase inhibitor, clade B, member 9f / serine (or cysteine) peptidase inhibitor, clade B, member 9e / serine (or cysteine) peptidase inhibitor, clade B, member 9g / predicted gene, OTTMUSG00000000724 | --- | serine-type endopeptidase inhibitor activity / peptidase activity | --- |
| 1438931_s_at | -0.42 | -3.37 | -0.76 | 0.69 | -0.44 | -3.00 | -0.09 | -5.93 | Sesn1 / LOC100047324 | sestrin 1 / similar to Sesn1 protein | cell cycle arrest | --- | nucleus |
| 1425139_at | 0.71 | -2.29 | 0.71 | -2.18 | 0.99 | 0.21 | 0.74 | -1.80 | Sesn2 | sestrin 2 | cell cycle arrest | --- | nucleus / cytoplasm |
| 1425026_at | 0.61 | 2.54 | 0.40 | -0.95 | 0.26 | -3.52 | 0.27 | -3.14 | Sft2d2 | SFT2 domain containing 2 | transport / protein transport | --- | membrane / integral to membrane |
| 1425027_s_at | 0.68 | 2.25 | 0.58 | 0.97 | 0.34 | -2.85 | 0.32 | -3.00 | Sft2d2 | SFT2 domain containing 2 | transport / protein transport | --- | membrane / integral to membrane |
| 1431300_at | -0.60 | -4.22 | -1.00 | -1.44 | -1.68 | 2.80 | -1.04 | -1.05 | Sgip1 | SH3-domain GRB2-like (endophilin) interacting protein 1 | --- | RNA-directed DNA polymerase activity / nuclease activity / endonuclease activity / transferase activity / nucleotidyltransferase activity / hydrolase activity | --- |
| 1426575_at | -0.86 | 1.02 | -0.52 | -2.50 | -0.47 | -3.10 | -0.15 | -5.70 | Sgms1 | sphingomyelin synthase 1 | lipid metabolic process / sphingolipid metabolic process / sphingomyelin biosynthetic process / apoptosis / cell growth / negative regulation of apoptosis | kinase activity / transferase activity / sphingomyelin synthase activity / sphingomyelin synthase activity / ceramide cholinephosphotransferase activity | Golgi trans cisterna / Golgi membrane / nucleus / endoplasmic reticulum / Golgi apparatus / plasma membrane / membrane / integral to membrane / integral to membrane / integral to Golgi membrane |
| 1426576_at | -0.73 | 2.48 | -0.60 | 0.71 | -0.80 | 3.35 | -0.39 | -2.27 | Sgms1 | sphingomyelin synthase 1 | lipid metabolic process / sphingolipid metabolic process / sphingomyelin biosynthetic process / apoptosis / cell growth / negative regulation of apoptosis | kinase activity / transferase activity / sphingomyelin synthase activity / sphingomyelin synthase activity / ceramide cholinephosphotransferase activity | Golgi trans cisterna / Golgi membrane / nucleus / endoplasmic reticulum / Golgi apparatus / plasma membrane / membrane / integral to membrane / integral to membrane / integral to Golgi membrane |
| 1436499_at | -0.79 | 4.58 | -0.60 | 1.86 | -0.76 | 4.10 | -0.44 | -0.52 | Sgms1 | sphingomyelin synthase 1 | lipid metabolic process / sphingolipid metabolic process / sphingomyelin biosynthetic process / apoptosis / cell growth / negative regulation of apoptosis | kinase activity / transferase activity / sphingomyelin synthase activity / sphingomyelin synthase activity / ceramide cholinephosphotransferase activity | Golgi trans cisterna / Golgi membrane / nucleus / endoplasmic reticulum / Golgi apparatus / plasma membrane / membrane / integral to membrane / integral to membrane / integral to Golgi membrane |
| 1436100_at | 0.21 | -6.03 | 0.15 | -6.06 | -1.08 | 0.23 | -0.50 | -4.02 | Sh2d5 | SH2 domain containing 5 | --- | protein binding | --- |
| 1434153_at | -0.57 | -0.01 | -0.51 | -0.85 | -0.59 | 0.31 | -0.37 | -2.74 | Shb | src homology 2 domain-containing transforming protein B | angiogenesis / apoptosis / multicellular organismal development / cell differentiation / B cell proliferation | SH3/SH2 adaptor activity / protein binding / protein binding | cytoplasm / membrane |
| 1426423_at | 0.19 | -5.30 | 0.41 | -2.08 | 0.72 | 2.29 | 0.61 | 0.84 | Shmt2 | serine hydroxymethyltransferase 2 (mitochondrial) | glycine metabolic process / L-serine metabolic process / one-carbon compound metabolic process | catalytic activity / glycine hydroxymethyltransferase activity / glycine hydroxymethyltransferase activity / methyltransferase activity / methyltransferase activity / transferase activity / pyridoxal phosphate binding | cytoplasm / mitochondrion / mitochondrion / mitochondrial inner membrane |
| 1434204_x_at | 0.33 | -3.87 | 0.50 | -1.38 | 0.42 | -2.54 | 0.61 | 0.13 | Shmt2 | Serine hydroxymethyltransferase 2 (mitochondrial) | glycine metabolic process / L-serine metabolic process / one-carbon compound metabolic process | catalytic activity / glycine hydroxymethyltransferase activity / glycine hydroxymethyltransferase activity / methyltransferase activity / methyltransferase activity / transferase activity / pyridoxal phosphate binding | cytoplasm / mitochondrion / mitochondrion / mitochondrial inner membrane |
| 1455084_x_at | 0.42 | -1.03 | 0.57 | 1.39 | 0.59 | 1.71 | 0.57 | 1.40 | Shmt2 | serine hydroxymethyltransferase 2 (mitochondrial) | glycine metabolic process / L-serine metabolic process / one-carbon compound metabolic process | catalytic activity / glycine hydroxymethyltransferase activity / glycine hydroxymethyltransferase activity / methyltransferase activity / methyltransferase activity / transferase activity / pyridoxal phosphate binding | cytoplasm / mitochondrion / mitochondrial inner membrane |
| 1455985_x_at | 0.34 | -3.44 | 0.32 | -3.52 | 0.60 | 0.43 | 0.25 | -4.32 | Shmt2 | serine hydroxymethyltransferase 2 (mitochondrial) | glycine metabolic process / L-serine metabolic process / one-carbon compound metabolic process | catalytic activity / glycine hydroxymethyltransferase activity / glycine hydroxymethyltransferase activity / methyltransferase activity / methyltransferase activity / transferase activity / pyridoxal phosphate binding | cytoplasm / mitochondrion / mitochondrion / mitochondrial inner membrane |
| 1434261_at | -0.29 | -5.32 | -0.38 | -4.45 | -0.89 | 0.23 | -0.72 | -1.32 | Sipa1l2 | signal-induced proliferation-associated 1 like 2 | regulation of small GTPase mediated signal transduction | GTPase activator activity / protein binding | intracellular |
| 1422054_a_at | 0.87 | 0.13 | 0.91 | 0.49 | 0.39 | -4.37 | 0.68 | -1.55 | Skil | SKI-like | cell differentiation | nucleotide binding / binding / protein binding | nucleus |
| 1426441_at | 1.13 | 5.35 | -0.21 | -5.23 | -0.06 | -6.22 | 0.07 | -5.95 | Slc11a2 | solute carrier family 11 (proton-coupled divalent metal ion transporters), member 2 | transport / ion transport / cobalt ion transport / iron ion transport / iron ion transport / iron ion transport | transporter activity / iron ion transmembrane transporter activity / iron ion binding / cobalt ion transmembrane transporter activity | endosome / early endosome / plasma membrane / endomembrane system / membrane / integral to membrane / integral to membrane |
| 1452078_a_at | 0.82 | 0.50 | 0.25 | -5.21 | 0.42 | -3.64 | 0.24 | -5.10 | Slc11a2 | solute carrier family 11 (proton-coupled divalent metal ion transporters), member 2 | transport / ion transport / cobalt ion transport / iron ion transport / iron ion transport / iron ion transport | transporter activity / iron ion transmembrane transporter activity / iron ion binding / cobalt ion transmembrane transporter activity | endosome / early endosome / plasma membrane / endomembrane system / membrane / integral to membrane / integral to membrane |
| 1417622_at | 0.76 | 1.50 | 0.49 | -1.79 | 0.33 | -3.94 | -0.01 | -6.10 | Slc12a2 | solute carrier family 12, member 2 | transport / ion transport / potassium ion transport / sodium ion transport / chloride transport / hyperosmotic response / gamma-aminobutyric acid signaling pathway / positive regulation of cell volume / detection of mechanical stimulus involved in sensory perception of sound | transporter activity / protein binding / sodium:potassium:chloride symporter activity / symporter activity / cation:chloride symporter activity / transmembrane transporter activity / potassium ion binding / sodium ion binding | membrane fraction / membrane / integral to membrane / integral to membrane / basolateral plasma membrane / apical plasma membrane / apical plasma membrane |
| 1417623_at | 0.60 | 2.45 | 0.29 | -2.99 | 0.23 | -3.97 | 0.13 | -5.29 | Slc12a2 | solute carrier family 12, member 2 | transport / ion transport / potassium ion transport / sodium ion transport / chloride transport / hyperosmotic response / gamma-aminobutyric acid signaling pathway / positive regulation of cell volume / detection of mechanical stimulus involved in sensory perception of sound | transporter activity / protein binding / sodium:potassium:chloride symporter activity / symporter activity / cation:chloride symporter activity / transmembrane transporter activity / potassium ion binding / sodium ion binding | membrane fraction / membrane / integral to membrane / integral to membrane / basolateral plasma membrane / apical plasma membrane / apical plasma membrane |
| 1451674_at | -0.12 | -6.10 | -0.37 | -3.43 | -0.65 | 0.05 | -0.40 | -3.04 | Slc12a5 | solute carrier family 12, member 5 | transport / ion transport / potassium ion transport / sodium ion transport / chloride transport / chloride transport / synaptic transmission | transporter activity / symporter activity / cation:chloride symporter activity / potassium:chloride symporter activity / potassium ion binding | plasma membrane / membrane / integral to membrane |
| 1426082_a_at | -1.23 | 5.07 | -1.32 | 5.64 | -1.62 | 7.61 | -0.85 | 1.52 | Slc16a4 | solute carrier family 16 (monocarboxylic acid transporters), member 4 | transport | transporter activity | integral to membrane |
| 1441315_s_at | 0.43 | -1.39 | 0.31 | -3.27 | 0.58 | 1.10 | 0.29 | -3.31 | Slc19a2 | solute carrier family 19 (thiamine transporter), member 2 | transport / transport / G-protein coupled receptor protein signaling pathway / G-protein coupled receptor protein signaling pathway / thiamin transport | rhodopsin-like receptor activity / rhodopsin-like receptor activity / folic acid binding / folic acid binding / folic acid transporter activity / reduced folate carrier activity / reduced folate carrier activity / thiamin transmembrane transporter activity | plasma membrane / membrane / membrane / integral to membrane / integral to membrane |
| 1423549_at | 0.72 | 1.10 | 0.48 | -1.97 | 0.32 | -3.94 | 0.23 | -4.79 | Slc1a4 | solute carrier family 1 (glutamate/neutral amino acid transporter), member 4 | transport / dicarboxylic acid transport | neutral amino acid transmembrane transporter activity / symporter activity / sodium:dicarboxylate symporter activity | intermediate filament / membrane / integral to membrane / integral to membrane / melanosome |
| 1423550_at | 0.66 | 0.42 | 0.38 | -3.12 | 0.58 | -0.55 | 0.27 | -4.37 | Slc1a4 | solute carrier family 1 (glutamate/neutral amino acid transporter), member 4 | transport / dicarboxylic acid transport | neutral amino acid transmembrane transporter activity / symporter activity / sodium:dicarboxylate symporter activity | intermediate filament / membrane / integral to membrane / integral to membrane / melanosome |
| 1456003_a_at | 0.88 | 3.29 | 0.49 | -1.52 | 0.64 | 0.47 | 0.35 | -3.22 | Slc1a4 | solute carrier family 1 (glutamate/neutral amino acid transporter), member 4 | transport / dicarboxylic acid transport | neutral amino acid transmembrane transporter activity / symporter activity / sodium:dicarboxylate symporter activity | intermediate filament / membrane / integral to membrane / integral to membrane / melanosome |
| 1448568_a_at | 1.19 | 0.38 | 1.02 | -0.76 | 1.23 | 0.69 | 1.02 | -0.67 | Slc20a1 | solute carrier family 20, member 1 | transport / phosphate transport / phosphate transport / positive regulation of I-kappaB kinase/NF-kappaB cascade | receptor activity / inorganic phosphate transmembrane transporter activity / inorganic phosphate transmembrane transporter activity / symporter activity / transmembrane transporter activity | extracellular space / integral to plasma membrane / membrane / integral to membrane / integral to membrane |
| 1444312_at | -0.32 | -3.59 | -0.39 | -2.34 | -0.61 | 0.77 | -0.38 | -2.39 | Slc22a15 | solute carrier family 22 (organic anion/cation transporter), member 15 | transport | transporter activity | integral to membrane |
| 1453004_at | 1.27 | 3.34 | 0.44 | -3.96 | 0.57 | -2.80 | 0.42 | -3.96 | Slc22a23 | solute carrier family 22, member 23 | transport / ion transport | transporter activity | membrane / integral to membrane |
| 1428440_at | -0.85 | 3.13 | -0.30 | -4.00 | -0.10 | -6.02 | -0.12 | -5.65 | Slc25a12 | solute carrier family 25 (mitochondrial carrier, Aralar), member 12 | transport / transport / aspartate transport / L-glutamate transport / malate-aspartate shuttle / malate-aspartate shuttle / response to calcium ion | transporter activity / L-glutamate transmembrane transporter activity / binding / binding / calcium ion binding / calcium ion binding / L-aspartate transmembrane transporter activity | mitochondrion / mitochondrial inner membrane / mitochondrial inner membrane / mitochondrial inner membrane / mitochondrial inner membrane / membrane / integral to membrane |
| 1424211_at | 0.84 | 1.22 | 0.69 | -0.32 | 0.49 | -2.57 | 0.57 | -1.54 | Slc25a33 | solute carrier family 25, member 33 | transport / transport / mitochondrial transport | binding / binding | mitochondrion / mitochondrial inner membrane / mitochondrial inner membrane / membrane / membrane / integral to membrane |
| 1427547_a_at | 0.09 | -6.10 | 0.61 | 1.73 | 0.03 | -6.26 | 0.26 | -3.61 | Slc26a3 | solute carrier family 26, member 3 | transport / sulfate transport | transporter activity / secondary active sulfate transmembrane transporter activity / antiporter activity / anion exchanger activity / chloride ion binding | integral to plasma membrane / membrane / integral to membrane |
| 1422786_at | 0.71 | 1.80 | 0.55 | -0.24 | 0.35 | -3.11 | 0.30 | -3.74 | Slc30a1 | solute carrier family 30 (zinc transporter), member 1 | in utero embryonic development / transport / ion transport / cation transport / zinc ion transport / zinc ion transport / cellular zinc ion homeostasis / nutrient import | zinc ion transmembrane transporter activity / zinc ion transmembrane transporter activity / zinc ion binding / cation transmembrane transporter activity | extracellular space / plasma membrane / plasma membrane / membrane / integral to membrane / integral to membrane |
| 1436164_at | 1.26 | 1.79 | 1.11 | 0.71 | 0.83 | -1.46 | 0.31 | -5.17 | Slc30a1 | solute carrier family 30 (zinc transporter), member 1 | in utero embryonic development / transport / ion transport / cation transport / zinc ion transport / zinc ion transport / cellular zinc ion homeostasis / nutrient import | zinc ion transmembrane transporter activity / zinc ion transmembrane transporter activity / zinc ion binding / cation transmembrane transporter activity | extracellular space / plasma membrane / plasma membrane / membrane / integral to membrane / integral to membrane |
| 1423621_a_at | -0.23 | -4.69 | -0.25 | -4.22 | -0.59 | 0.88 | -0.28 | -3.72 | Slc33a1 | solute carrier family 33 (acetyl-CoA transporter), member 1 | transport | acetyl-CoA transporter activity | endoplasmic reticulum / endoplasmic reticulum membrane / membrane / integral to membrane / integral to membrane |
| 1457024_x_at | -0.34 | -3.64 | -0.29 | -4.12 | -0.65 | 0.67 | -0.46 | -1.82 | Slc35a2 | solute carrier family 35 (UDP-galactose transporter), member A2 | transport / transport / carbohydrate transport / carbohydrate transport / nucleotide-sugar transport | nucleotide-sugar transmembrane transporter activity / sugar:hydrogen symporter activity / UDP-galactose transmembrane transporter activity / UDP-galactose transmembrane transporter activity | Golgi membrane / Golgi membrane / Golgi apparatus / membrane / integral to membrane |
| 1420054_s_at | 0.09 | -6.21 | 0.31 | -3.91 | 0.64 | 0.46 | 0.43 | -2.28 | Slc35c2 | solute carrier family 35, member C2 | transport / metabolic process | oxidoreductase activity | membrane / integral to membrane |
| 1434167_at | 0.25 | -4.62 | 0.40 | -2.46 | 0.55 | -0.31 | 0.58 | 0.18 | Slc35e4 | solute carrier family 35, member E4 | --- | --- | membrane / integral to membrane |
| 1436693_x_at | 0.39 | -0.76 | 0.50 | 1.33 | 0.76 | 5.26 | 0.48 | 0.98 | Slc35e4 | solute carrier family 35, member E4 | --- | --- | membrane / integral to membrane |
| 1429594_at | 0.59 | 2.27 | 0.54 | 1.55 | 0.29 | -2.89 | 0.20 | -4.21 | Slc38a2 | solute carrier family 38, member 2 | transport / ion transport / sodium ion transport / amino acid transport | amino acid transmembrane transporter activity / symporter activity / sodium ion binding | plasma membrane / plasma membrane / membrane / integral to membrane |
| 1428111_at | -1.01 | 2.93 | -0.92 | 2.08 | -0.64 | -0.91 | -0.33 | -4.24 | Slc38a4 | solute carrier family 38, member 4 | transport / ion transport / sodium ion transport / amino acid transport / amino acid transport | amine transmembrane transporter activity / symporter activity / sodium ion binding | plasma membrane / membrane / membrane / integral to membrane |
| 1455295_at | 0.29 | -3.82 | 0.29 | -3.68 | 0.58 | 0.79 | 0.44 | -1.31 | Slc38a7 | solute carrier family 38, member 7 | transport / ion transport / sodium ion transport / amino acid transport | sodium ion binding | membrane / integral to membrane |
| 1416832_at | -1.15 | 7.30 | -0.84 | 4.05 | -0.61 | 1.13 | -0.64 | 1.55 | Slc39a8 | solute carrier family 39 (metal ion transporter), member 8 | transport / ion transport / zinc ion transport / metal ion transport / metal ion transport | zinc ion binding / metal ion transmembrane transporter activity / metal ion transmembrane transporter activity | extracellular space / membrane / membrane / integral to membrane / integral to membrane |
| 1425364_a_at | 0.84 | 0.86 | 0.94 | 1.83 | 0.88 | 1.29 | 0.68 | -0.69 | Slc3a2 | solute carrier family 3 (activators of dibasic and neutral amino acid transport), member 2 | carbohydrate metabolic process / transport / amino acid transport / tryptophan transport | catalytic activity / protein binding / protein binding / cation binding | plasma membrane / cell surface / membrane / integral to membrane / integral to membrane / melanosome |
| 1417061_at | -0.88 | 3.23 | -0.09 | -6.05 | -0.07 | -6.15 | -0.14 | -5.54 | Slc40a1 | solute carrier family 40 (iron-regulated transporter), member 1 | transport / transport / ion transport / iron ion transport / iron ion transport | iron ion transmembrane transporter activity / iron ion transmembrane transporter activity / binding / iron ion binding | mitochondrial inner membrane / plasma membrane / synaptic vesicle / membrane / integral to membrane / integral to membrane |
| 1448566_at | -0.93 | 2.03 | -0.08 | -6.17 | 0.20 | -5.53 | -0.09 | -5.94 | Slc40a1 | solute carrier family 40 (iron-regulated transporter), member 1 | transport / transport / ion transport / iron ion transport / iron ion transport | iron ion transmembrane transporter activity / iron ion transmembrane transporter activity / binding / iron ion binding | mitochondrial inner membrane / plasma membrane / synaptic vesicle / membrane / integral to membrane / integral to membrane |
| 1452445_at | 0.19 | -5.68 | -0.18 | -5.60 | -0.87 | 1.74 | -0.46 | -2.72 | Slc41a2 | Solute carrier family 41, member 2 | transport / ion transport / cation transport / cation transport | magnesium ion binding / cation transmembrane transporter activity / cation transmembrane transporter activity | plasma membrane / membrane / integral to membrane / integral to membrane |
| 1422788_at | 0.57 | -0.64 | 0.73 | 1.43 | 0.77 | 1.94 | 0.78 | 1.90 | Slc43a3 | solute carrier family 43, member 3 | --- | --- | extracellular space / membrane / integral to membrane / integral to membrane |
| 1426663_s_at | 0.60 | 0.68 | 0.24 | -4.48 | 0.03 | -6.27 | 0.26 | -4.15 | Slc45a3 | solute carrier family 45, member 3 | transport | --- | membrane / integral to membrane / integral to membrane |
| 1418395_at | 0.13 | -5.89 | 0.71 | 2.25 | 0.45 | -1.37 | 0.10 | -5.72 | Slc47a1 | solute carrier family 47, member 1 | transport / multidrug transport | drug transporter activity / antiporter activity | plasma membrane / membrane / integral to membrane / integral to membrane |
| 1434096_at | -0.60 | -1.14 | -0.56 | -1.45 | -0.80 | 1.27 | -0.40 | -3.24 | Slc4a4 | solute carrier family 4 (anion exchanger), member 4 | transport / ion transport / sodium ion transport / sodium ion transport / anion transport / regulation of pH / bicarbonate transport | transporter activity / inorganic anion exchanger activity / anion transmembrane transporter activity / sodium:bicarbonate symporter activity / symporter activity / anion exchanger activity / sodium ion binding | integral to plasma membrane / membrane / integral to membrane / integral to membrane / basolateral plasma membrane |
| 1435484_at | 0.72 | 1.68 | 0.20 | -5.13 | 0.04 | -6.26 | -0.29 | -3.88 | Slc5a3 | solute carrier family 5 (inositol transporters), member 3 | inositol metabolic process / translation / transport / ion transport / sodium ion transport / peripheral nervous system development / myo-inositol transport / regulation of respiratory gaseous exchange | structural constituent of ribosome / transporter activity / symporter activity / sodium ion binding | intracellular / mitochondrion / mitochondrion / ribosome / membrane / integral to membrane / integral to membrane / ribonucleoprotein complex |
| 1417636_at | 1.28 | 8.22 | 1.17 | 7.06 | 0.84 | 3.96 | 0.54 | -0.01 | Slc6a9 | solute carrier family 6 (neurotransmitter transporter, glycine), member 9 | transport / neurotransmitter transport / amino acid transport | sodium:amino acid symporter activity / neurotransmitter:sodium symporter activity / symporter activity | integral to plasma membrane / membrane / integral to membrane / integral to membrane |
| 1454991_at | 0.74 | 1.51 | 0.39 | -3.00 | 0.41 | -2.73 | 0.03 | -6.07 | Slc7a1 | solute carrier family 7 (cationic amino acid transporter, y+ system), member 1 | transport / amino acid transport / arginine transport | receptor activity / amino acid transmembrane transporter activity / arginine transmembrane transporter activity | membrane / integral to membrane / integral to membrane |
| 1454992_at | 1.11 | 8.67 | 0.63 | 2.95 | 0.27 | -3.14 | 0.07 | -5.87 | Slc7a1 | solute carrier family 7 (cationic amino acid transporter, y+ system), member 1 | transport / amino acid transport / arginine transport | receptor activity / amino acid transmembrane transporter activity / arginine transmembrane transporter activity | membrane / integral to membrane / integral to membrane |
| 1420413_at | 1.43 | 1.50 | 1.65 | 2.83 | 1.74 | 3.32 | 1.06 | -0.82 | Slc7a11 | solute carrier family 7 (cationic amino acid transporter, y+ system), member 11 | transport / amino acid transport | amino acid transmembrane transporter activity | membrane / integral to membrane / integral to membrane |
| 1443536_at | 1.91 | 4.07 | 1.50 | 1.77 | 1.85 | 3.74 | 1.19 | -0.12 | Slc7a11 | solute carrier family 7 (cationic amino acid transporter, y+ system), member 11 | transport / amino acid transport | amino acid transmembrane transporter activity | membrane / integral to membrane / integral to membrane |
| 1422648_at | 1.90 | 8.08 | 1.03 | 2.05 | 0.15 | -5.94 | -0.05 | -6.06 | Slc7a2 | solute carrier family 7 (cationic amino acid transporter, y+ system), member 2 | production of nitric oxide during acute inflammatory response / nitric oxide biosynthetic process / transport / amino acid transport / L-amino acid transport / arginine transport / arginine transport / lysine transport / ornithine transport / macrophage activation / regulation of inflammatory response | L-ornithine transmembrane transporter activity / high affinity arginine transmembrane transporter activity / amino acid transmembrane transporter activity / L-amino acid transmembrane transporter activity / L-amino acid transmembrane transporter activity / arginine transmembrane transporter activity / arginine transmembrane transporter activity / L-lysine transmembrane transporter activity | membrane / integral to membrane / integral to membrane / integral to membrane / integral to membrane |
| 1426008_a_at | 1.47 | 4.32 | 0.82 | -0.78 | 0.28 | -5.33 | 0.22 | -5.50 | Slc7a2 | solute carrier family 7 (cationic amino acid transporter, y+ system), member 2 | production of nitric oxide during acute inflammatory response / nitric oxide biosynthetic process / transport / amino acid transport / L-amino acid transport / arginine transport / arginine transport / lysine transport / ornithine transport / macrophage activation / regulation of inflammatory response | L-ornithine transmembrane transporter activity / high affinity arginine transmembrane transporter activity / amino acid transmembrane transporter activity / L-amino acid transmembrane transporter activity / L-amino acid transmembrane transporter activity / arginine transmembrane transporter activity / arginine transmembrane transporter activity / L-lysine transmembrane transporter activity | membrane / integral to membrane / integral to membrane / integral to membrane / integral to membrane |
| 1436555_at | 2.22 | 9.76 | 1.08 | 2.59 | 0.16 | -5.87 | 0.02 | -6.09 | Slc7a2 | solute carrier family 7 (cationic amino acid transporter, y+ system), member 2 | production of nitric oxide during acute inflammatory response / nitric oxide biosynthetic process / transport / amino acid transport / L-amino acid transport / arginine transport / arginine transport / lysine transport / ornithine transport / macrophage activation / regulation of inflammatory response | L-ornithine transmembrane transporter activity / high affinity arginine transmembrane transporter activity / amino acid transmembrane transporter activity / L-amino acid transmembrane transporter activity / L-amino acid transmembrane transporter activity / arginine transmembrane transporter activity / arginine transmembrane transporter activity / L-lysine transmembrane transporter activity | membrane / integral to membrane / integral to membrane / integral to membrane / integral to membrane |
| 1418326_at | 0.94 | 1.33 | 0.44 | -3.65 | 0.80 | 0.03 | 0.46 | -3.30 | Slc7a5 / LOC100047619 | solute carrier family 7 (cationic amino acid transporter, y+ system), member 5 / similar to solute carrier family 7 (cationic amino acid transporter, y+ system), member 5 | transport / amino acid transport / L-amino acid transport | amino acid transmembrane transporter activity / L-amino acid transmembrane transporter activity / transmembrane transporter activity | integral to plasma membrane / membrane / integral to membrane / integral to membrane |
| 1429596_at | 0.32 | -3.11 | 0.20 | -4.72 | 0.69 | 2.70 | 0.34 | -2.48 | Slc7a6os | solute carrier family 7, member 6 opposite strand | --- | --- | --- |
| 1450165_at | 0.90 | 0.15 | 0.25 | -5.49 | 0.71 | -1.61 | 0.27 | -5.16 | Slfn2 | schlafen 2 | negative regulation of cell proliferation | --- | --- |
| 1449336_a_at | 0.77 | 3.05 | 0.38 | -2.41 | 0.07 | -6.12 | 0.14 | -5.44 | Slk | STE20-like kinase (yeast) | nucleotide-excision repair / protein amino acid phosphorylation / apoptosis | nucleotide binding / DNA binding / nuclease activity / protein kinase activity / protein serine/threonine kinase activity / ATP binding / kinase activity / transferase activity | cytoplasm |
| 1416620_at | -0.61 | 1.62 | -0.27 | -3.69 | 0.17 | -5.22 | -0.05 | -5.98 | Smarcal1 | Swi/SNF related matrix associated, actin dependent regulator of chromatin, subfamily a-like 1 | chromatin modification | nucleotide binding / nucleic acid binding / DNA binding / helicase activity / ATP binding / DNA-dependent ATPase activity / hydrolase activity | nucleus |
| 1448621_a_at | -0.64 | 2.22 | -0.26 | -3.85 | -0.25 | -4.07 | -0.03 | -6.06 | Smpd1 | sphingomyelin phosphodiesterase 1, acid lysosomal | sphingomyelin catabolic process / metabolic process | sphingomyelin phosphodiesterase activity / integrase activity / hydrolase activity / hydrolase activity, acting on glycosyl bonds | lysosome |
| 1455794_at | -0.41 | -5.15 | -0.44 | -4.83 | -1.16 | 0.26 | -0.49 | -4.35 | Smtnl2 | smoothelin-like 2 | --- | --- | --- |
| 1452817_at | -0.59 | 0.41 | -0.07 | -6.10 | -0.06 | -6.18 | -0.07 | -5.93 | Smyd3 | SET and MYND domain containing 3 | chromatin modification | methyltransferase activity / zinc ion binding / transferase activity / histone-lysine N-methyltransferase activity / metal ion binding | nucleus / cytoplasm |
| 1415756_a_at | -0.60 | 0.92 | -0.13 | -5.65 | -0.20 | -4.93 | 0.01 | -6.09 | Snapin | SNAP-associated protein | exocytosis / synaptic vesicle exocytosis | SNARE binding / protein binding | cytoplasm / cytosol / membrane / synaptosome / synaptosome / cell junction / cytoplasmic vesicle membrane / cytoplasmic vesicle / synapse |
| 1459601_at | -0.13 | -6.24 | -0.74 | -1.41 | -0.99 | 0.86 | -0.12 | -5.90 | Snf1lk | SNF1-like kinase | protein amino acid phosphorylation / protein amino acid phosphorylation / cell cycle / protein kinase cascade / protein kinase cascade / multicellular organismal development / regulation of mitotic cell cycle / cell differentiation / regulation of cell differentiation | nucleotide binding / magnesium ion binding / magnesium ion binding / protein kinase activity / protein serine/threonine kinase activity / protein serine/threonine kinase activity / protein binding / ATP binding / ATP binding / kinase activity / transferase activity / metal ion binding | nucleus / nucleus / cytoplasm / cytoplasm |
| 1433674_a_at | 0.90 | 2.94 | 0.87 | 2.66 | 0.43 | -2.65 | 0.28 | -4.33 | Snhg1 | small nucleolar RNA host gene (non-protein coding) 1 | --- | --- | --- |
| 1433675_at | 0.92 | 1.51 | 0.83 | 0.65 | 0.48 | -3.08 | 0.31 | -4.53 | Snhg1 | small nucleolar RNA host gene (non-protein coding) 1 | --- | --- | --- |
| 1452789_at | -0.01 | -6.44 | 0.41 | -2.52 | 0.80 | 2.52 | 0.17 | -5.30 | Snn | stannin | --- | --- | membrane / integral to membrane / integral to membrane |
| 1428822_a_at | -0.65 | 3.52 | -0.39 | -0.72 | -0.46 | 0.45 | -0.35 | -1.41 | Snx24 | sorting nexing 24 | transport / cell communication / protein transport | protein binding / phosphoinositide binding | --- |
| 1455020_at | -0.27 | -4.77 | -0.26 | -4.67 | -0.64 | 0.00 | -0.41 | -2.82 | Snx25 | sorting nexin 25 | cell communication / intracellular signaling cascade | signal transducer activity / protein binding / phosphoinositide binding | --- |
| 1437197_at | -1.29 | 3.76 | -0.89 | 0.39 | -0.36 | -4.57 | -0.18 | -5.62 | Sorbs2 | sorbin and SH3 domain containing 2 | --- | --- | --- |
| 1426584_a_at | -0.72 | 0.14 | -0.45 | -2.97 | -0.24 | -5.18 | 0.03 | -6.08 | Sord | sorbitol dehydrogenase | response to osmotic stress / metabolic process / response to hormone stimulus / response to nutrient levels / response to drug / response to cadmium ion / response to copper ion | catalytic activity / L-iditol 2-dehydrogenase activity / L-iditol 2-dehydrogenase activity / binding / zinc ion binding / oxidoreductase activity / oxidoreductase activity, acting on the CH-OH group of donors, NAD or NADP as acceptor / identical protein binding / metal ion binding / NAD binding | --- |
| 1417254_at | 0.60 | -1.60 | 0.31 | -4.63 | 0.84 | 1.10 | 0.39 | -3.67 | Spata5 | spermatogenesis associated 5 | multicellular organismal development / spermatogenesis / cell differentiation | nucleotide binding / nucleotide binding / binding / ATP binding / nucleoside-triphosphatase activity | cytoplasm / mitochondrion |
| 1438844_x_at | 0.34 | -4.53 | 0.30 | -4.68 | 0.80 | 0.56 | 0.20 | -5.36 | Spata5 | spermatogenesis associated 5 | multicellular organismal development / spermatogenesis / cell differentiation | nucleotide binding / nucleotide binding / binding / ATP binding / nucleoside-triphosphatase activity | cytoplasm / mitochondrion |
| 1448639_a_at | 0.53 | -2.15 | 0.37 | -3.86 | 0.86 | 1.59 | 0.42 | -3.22 | Spata5 | spermatogenesis associated 5 | multicellular organismal development / spermatogenesis / cell differentiation | nucleotide binding / nucleotide binding / binding / ATP binding / nucleoside-triphosphatase activity | cytoplasm / mitochondrion |
| 1455863_at | 0.37 | -2.91 | 0.13 | -5.71 | 0.81 | 3.22 | 0.54 | -0.32 | Spata5l1 | spermatogenesis associated 5-like 1 | --- | nucleotide binding / ATP binding / nucleoside-triphosphatase activity | --- |
| 1428260_at | -0.72 | -1.41 | -0.54 | -3.06 | -1.02 | 1.32 | -0.67 | -1.71 | Spg3a | spastic paraplegia 3A homolog (human) | --- | nucleotide binding / DNA binding / GTPase activity / GTP binding | Golgi membrane / nucleus / Golgi apparatus / membrane / integral to membrane |
| 1454962_at | 0.72 | 2.64 | 0.38 | -2.26 | 0.06 | -6.18 | 0.01 | -6.09 | Spire1 | spire homolog 1 (Drosophila) | transport / Golgi vesicle transport | actin binding / zinc ion binding | cytoplasm / Golgi apparatus / cytoskeleton |
| 1451601_a_at | -0.79 | -2.09 | -0.82 | -1.80 | -1.19 | 0.98 | -0.91 | -1.04 | Spns2 | spinster homolog 2 (Drosophila) | transport | transporter activity | membrane / integral to membrane |
| 1435274_at | -0.21 | -5.18 | -0.83 | 2.97 | -0.12 | -5.87 | -0.28 | -4.05 | Spopl | speckle-type POZ protein-like | ubiquitin cycle | protein binding | nucleus |
| 1422672_at | 0.54 | -4.24 | 1.53 | 2.87 | 1.35 | 1.78 | 1.30 | 1.42 | Sprr1b | small proline-rich protein 1B | regulation of cell shape / keratinization | structural molecule activity / structural constituent of cytoskeleton | cornified envelope / cytoplasm |
| 1422240_s_at | 0.94 | 3.82 | 0.63 | 0.20 | 0.28 | -4.32 | 0.17 | -5.32 | Sprr2h | small proline-rich protein 2H | epidermis development / keratinocyte differentiation / keratinization | structural molecule activity | cornified envelope / cytoplasm |
| 1434885_at | 0.59 | -0.59 | 0.69 | 0.76 | 0.37 | -3.37 | 0.23 | -4.85 | Spty2d1 | SPT2, Suppressor of Ty, domain containing 1 (S. cerevisiae) | --- | --- | --- |
| 1415993_at | -0.49 | -3.48 | -0.61 | -2.19 | -0.85 | 0.09 | -0.32 | -4.73 | Sqle | squalene epoxidase | metabolic process | squalene monooxygenase activity / oxidoreductase activity | extracellular space / endoplasmic reticulum / endoplasmic reticulum membrane / microsome / membrane / integral to membrane / integral to membrane |
| 1444021_at | 1.53 | 1.15 | 1.13 | -1.20 | 1.11 | -1.35 | 0.95 | -2.18 | Sqstm1 | sequestosome 1 | apoptosis / immune response / cell differentiation / regulation of I-kappaB kinase/NF-kappaB cascade | transcription cofactor activity / protein binding / protein binding / zinc ion binding / protein kinase binding / SH2 domain binding / ubiquitin binding / metal ion binding | nucleus / cytoplasm / endosome / late endosome |
| 1450957_a_at | 0.96 | 5.04 | 0.97 | 5.05 | 0.83 | 3.54 | 0.44 | -1.60 | Sqstm1 | sequestosome 1 | apoptosis / immune response / cell differentiation / regulation of I-kappaB kinase/NF-kappaB cascade | transcription cofactor activity / protein binding / protein binding / zinc ion binding / protein kinase binding / SH2 domain binding / ubiquitin binding / metal ion binding | nucleus / cytoplasm / endosome / late endosome |
| 1426690_a_at | -0.62 | 3.17 | -0.39 | -0.64 | -0.44 | 0.24 | -0.22 | -3.85 | Srebf1 | sterol regulatory element binding transcription factor 1 | transcription / regulation of transcription, DNA-dependent / lipid metabolic process / steroid metabolic process / cholesterol metabolic process / cellular response to starvation / regulation of transcription / regulation of transcription | DNA binding / DNA binding / transcription factor activity / transcription regulator activity / sterol response element binding | Golgi membrane / nucleus / nucleus / endoplasmic reticulum / endoplasmic reticulum membrane / Golgi apparatus / membrane / integral to membrane / cytoplasmic vesicle |
| 1436867_at | 0.16 | -5.90 | 0.17 | -5.65 | 0.85 | 1.62 | 0.44 | -2.95 | Srl | sarcalumenin | --- | calcium ion binding / calcium ion binding | extracellular space / sarcoplasmic reticulum / sarcoplasmic reticulum lumen |
| 1426875_s_at | 0.43 | -2.66 | 0.89 | 3.06 | 0.54 | -1.02 | 0.29 | -4.17 | Srxn1 | sulfiredoxin 1 homolog (S. cerevisiae) | response to oxidative stress / response to oxidative stress | nucleotide binding / magnesium ion binding / DNA binding / ATP binding / antioxidant activity / oxidoreductase activity / oxidoreductase activity, acting on sulfur group of donors / sulfiredoxin activity | cytoplasm / cytosol / cytosol |
| 1441327_a_at | -0.27 | -3.73 | -0.20 | -4.72 | -0.59 | 1.57 | -0.63 | 2.07 | Ssr1 | signal sequence receptor, alpha | --- | receptor activity / calcium ion binding | endoplasmic reticulum / endoplasmic reticulum membrane / membrane / integral to membrane / integral to membrane |
| 1418946_at | -0.44 | -4.49 | -0.71 | -2.10 | -0.98 | 0.14 | -0.51 | -3.67 | St3gal1 | ST3 beta-galactoside alpha-2,3-sialyltransferase 1 | protein amino acid glycosylation | beta-galactoside alpha-2,3-sialyltransferase activity / sialyltransferase activity / transferase activity / transferase activity, transferring glycosyl groups | extracellular region / extracellular space / Golgi apparatus / membrane / integral to membrane / integral to Golgi membrane |
| 1420928_at | -0.59 | 3.70 | -0.29 | -2.07 | -0.41 | 0.54 | -0.48 | 1.75 | St6gal1 | beta galactoside alpha 2,6 sialyltransferase 1 | protein amino acid glycosylation | beta-galactoside alpha-2,6-sialyltransferase activity / sialyltransferase activity / sialyltransferase activity / transferase activity / transferase activity, transferring glycosyl groups | extracellular region / Golgi apparatus / membrane / integral to membrane / integral to membrane / integral to Golgi membrane |
| 1418074_at | 0.66 | 0.89 | 0.66 | 0.98 | 0.51 | -1.07 | 0.31 | -3.75 | St6galnac4 | ST6 (alpha-N-acetyl-neuraminyl-2,3-beta-galactosyl-1,3)-N-acetylgalactosaminide alpha-2,6-sialyltransferase 4 | protein amino acid glycosylation | sialyltransferase activity / transferase activity / transferase activity, transferring glycosyl groups / (alpha-N-acetylneuraminyl-2,3-beta-galactosyl-1,3)-N-acetyl-galactosaminide 6-alpha-sialyltransferase activity | Golgi membrane / extracellular space / Golgi apparatus / membrane / integral to membrane / integral to Golgi membrane |
| 1421849_at | -0.78 | 2.92 | -0.61 | 0.72 | -0.41 | -2.13 | -0.37 | -2.57 | Stag2 | stromal antigen 2 | cell cycle / chromosome segregation / mitosis / meiosis / cell division | binding | nucleus |
| 1431962_a_at | -0.60 | 0.38 | -0.10 | -5.98 | 0.15 | -5.63 | -0.19 | -5.07 | Stambp | Stam binding protein | ubiquitin cycle / anti-apoptosis / signal transduction | ubiquitin thiolesterase activity / protein binding / peptidase activity / metallopeptidase activity / zinc ion binding / hydrolase activity / metal ion binding | nucleus / cytoplasm / membrane |
| 1429239_a_at | -0.23 | -5.02 | -0.65 | 0.83 | -0.66 | 0.88 | -0.28 | -4.05 | Stard4 | StAR-related lipid transfer (START) domain containing 4 | steroid biosynthetic process / transport / lipid transport | lipid binding / cholesterol binding / cholesterol binding / cholesterol transporter activity | mitochondrion |
| 1429240_at | -0.72 | 0.12 | -1.00 | 3.12 | -0.97 | 2.80 | -0.53 | -1.87 | Stard4 | StAR-related lipid transfer (START) domain containing 4 | steroid biosynthetic process / transport / lipid transport | lipid binding / cholesterol binding / cholesterol binding / cholesterol transporter activity | mitochondrion |
| 1455011_at | -0.75 | 0.43 | -0.82 | 1.22 | -0.96 | 2.64 | -0.56 | -1.59 | Stard4 | StAR-related lipid transfer (START) domain containing 4 | steroid biosynthetic process / transport / lipid transport | lipid binding / cholesterol binding / cholesterol binding / cholesterol transporter activity | mitochondrion |
| 1450259_a_at | 0.75 | 5.42 | -0.19 | -4.40 | -0.29 | -2.35 | -0.02 | -6.07 | Stat5a | signal transducer and activator of transcription 5A | luteinization / natural killer cell differentiation / transcription / regulation of transcription, DNA-dependent / signal transduction / JAK-STAT cascade / female pregnancy / lactation / positive regulation of cell proliferation / positive regulation of cell proliferation / peptidyl-tyrosine phosphorylation / regulation of steroid metabolic process / cytokine and chemokine mediated signaling pathway / sequestering of lipid / regulation of cell adhesion / regulation of epithelial cell differentiation / mammary gland development / positive regulation of multicellular organism growth / positive regulation of activated T cell proliferation / positive regulation of activated T cell proliferation / progesterone metabolic process / T cell homeostasis / negative regulation of apoptosis / response to peptide hormone stimulus / positive regulation of interleukin-2 biosynthetic process / regulation of transcription / positive regulation of B cell differentiation / negative regulation of erythrocyte differentiation / positive regulation of survival gene product expression / positive regulation of mitotic cell cycle / positive regulation of transcription from RNA polymerase II promoter / positive regulation of transcription from RNA polymerase II promoter / development of secondary female sexual characteristics / development of secondary male sexual characteristics / development of secondary male sexual characteristics / positive regulation of inflammatory response | DNA binding / DNA binding / transcription factor activity / signal transducer activity / signal transducer activity / calcium ion binding / protein binding / protein binding | nucleus / nucleus / cytoplasm / cytoplasm |
| 1460197_a_at | 0.77 | 1.39 | 0.00 | -6.28 | -0.05 | -6.23 | -0.01 | -6.09 | Steap4 | STEAP family member 4 | transport / ion transport / iron ion transport / metabolic process / fat cell differentiation / oxidation reduction | catalytic activity / binding / iron ion binding / copper ion binding / electron carrier activity / oxidoreductase activity / metal ion binding / FAD binding | Golgi membrane / Golgi apparatus / plasma membrane / integral to plasma membrane / membrane / integral to membrane |
| 1417751_at | 0.65 | 3.52 | 0.41 | -0.44 | 0.13 | -5.48 | 0.07 | -5.86 | Stk10 | serine/threonine kinase 10 | protein amino acid phosphorylation | nucleotide binding / protein kinase activity / protein serine/threonine kinase activity / protein serine/threonine kinase activity / ATP binding / kinase activity / transferase activity | --- |
| 1423452_at | 0.67 | 1.42 | 0.48 | -1.14 | 0.55 | -0.15 | 0.46 | -1.34 | Stk17b | serine/threonine kinase 17b (apoptosis-inducing) | protein amino acid phosphorylation / protein amino acid phosphorylation / protein amino acid phosphorylation / apoptosis / apoptosis / induction of apoptosis / induction of apoptosis / induction of apoptosis / protein kinase cascade / protein kinase cascade / protein kinase cascade | nucleotide binding / protein kinase activity / protein kinase activity / protein serine/threonine kinase activity / protein serine/threonine kinase activity / protein serine/threonine kinase activity / protein binding / ATP binding / ATP binding / kinase activity / transferase activity | nucleus / nucleus / nucleus / actin cytoskeleton / actin cytoskeleton |
| 1450997_at | 0.50 | -1.94 | 0.28 | -4.51 | 0.72 | 0.82 | 0.31 | -4.11 | Stk17b | serine/threonine kinase 17b (apoptosis-inducing) | protein amino acid phosphorylation / protein amino acid phosphorylation / protein amino acid phosphorylation / apoptosis / apoptosis / induction of apoptosis / induction of apoptosis / induction of apoptosis / protein kinase cascade / protein kinase cascade / protein kinase cascade | nucleotide binding / protein kinase activity / protein kinase activity / protein serine/threonine kinase activity / protein serine/threonine kinase activity / protein serine/threonine kinase activity / protein binding / ATP binding / ATP binding / kinase activity / transferase activity | nucleus / nucleus / nucleus / actin cytoskeleton / actin cytoskeleton |
| 1453228_at | 0.93 | 4.64 | -0.25 | -4.42 | -0.16 | -5.52 | -0.43 | -1.73 | Stx11 | syntaxin 11 | transport / intracellular protein transport / membrane fusion / protein transport / vesicle-mediated transport | SNAP receptor activity / SNAP receptor activity / protein binding | Golgi apparatus / membrane |
| 1451063_at | -0.66 | 1.76 | -0.19 | -5.04 | 0.13 | -5.71 | 0.00 | -6.10 | Stxbp4 | syntaxin binding protein 4 | protein targeting / intracellular signaling cascade / insulin receptor signaling pathway / glucose transport | protein binding / protein binding | cytoplasm |
| 1437190_at | -0.37 | -3.32 | -0.65 | 0.61 | -0.20 | -5.23 | -0.32 | -3.67 | Styk1 | serine/threonine/tyrosine kinase 1 | protein amino acid phosphorylation | nucleotide binding / protein kinase activity / protein tyrosine kinase activity / non-membrane spanning protein tyrosine kinase activity / ATP binding / kinase activity / transferase activity | cytoplasm / membrane / integral to membrane |
| 1419702_at | 0.50 | -0.71 | 0.58 | 0.63 | 0.40 | -2.07 | 0.21 | -4.68 | Taf1a | TATA box binding protein (Tbp)-associated factor, RNA polymerase I, A | transcription / regulation of transcription, DNA-dependent / transcription from RNA polymerase I promoter / transcription from RNA polymerase I promoter / transport | DNA binding / RNA polymerase I transcription factor activity / transporter activity / protein binding / protein binding / transcription regulator activity | RNA polymerase I transcription factor complex / RNA polymerase I transcription factor complex / nucleus / membrane |
| 1451544_at | 0.59 | 2.18 | 0.08 | -5.96 | -0.19 | -4.63 | -0.21 | -4.22 | Tapbpl | TAP binding protein-like | antigen processing and presentation of endogenous peptide antigen via MHC class I | --- | endoplasmic reticulum / endoplasmic reticulum membrane / microsome / plasma membrane / membrane / integral to membrane |
| 1443896_at | -0.60 | 3.23 | -0.10 | -5.74 | 0.18 | -4.63 | 0.05 | -5.92 | Tbc1d5 | TBC1 domain family, member 5 | regulation of Rab GTPase activity | GTPase activator activity / Rab GTPase activator activity | intracellular |
| 1416996_at | -0.24 | -5.29 | 0.12 | -5.99 | 0.95 | 2.61 | 0.42 | -3.06 | Tbc1d8 | TBC1 domain family, member 8 | regulation of Rab GTPase activity | GTPase activator activity / Rab GTPase activator activity | intracellular |
| 1430133_at | -0.55 | -2.47 | -1.14 | 3.47 | -1.02 | 2.41 | -0.90 | 1.30 | Tbc1d8b | TBC1 domain family, member 8B | --- | --- | --- |
| 1434224_at | -0.15 | -6.07 | -0.35 | -4.38 | -0.81 | 0.31 | -0.26 | -4.99 | Tbl2 | transducin (beta)-like 2 | --- | --- | extracellular space |
| 1415750_at | 0.09 | -6.00 | 0.19 | -4.61 | 0.67 | 3.40 | 0.43 | -0.36 | Tbl3 | transducin (beta)-like 3 | rRNA processing / proteolysis | serine-type endopeptidase activity | small subunit processome |
| 1445757_at | -0.32 | -3.53 | -0.64 | 1.34 | -0.59 | 0.66 | -0.36 | -2.73 | Tbx3 | T-box 3 | skeletal development / blood vessel development / in utero embryonic development / heart morphogenesis / transcription / regulation of transcription, DNA-dependent / anti-apoptosis / multicellular organismal development / cell aging / positive regulation of cell proliferation / determination of anterior/posterior axis, embryo / organ morphogenesis / negative regulation of transcription / limbic system development / male genitalia development / female genitalia development / mammary gland development / mammary gland development / luteinizing hormone secretion / limb morphogenesis / embryonic arm morphogenesis / forelimb morphogenesis / embryonic digit morphogenesis / regulation of transcription / negative regulation of myoblast differentiation / positive regulation of cell cycle / negative regulation of transcription, DNA-dependent / negative regulation of transcription, DNA-dependent / positive regulation of transcription, DNA-dependent / follicle-stimulating hormone secretion / mesoderm morphogenesis / branching morphogenesis of a tube | DNA binding / transcription factor activity / transcription factor activity / transcription repressor activity / general transcriptional repressor activity / sequence-specific DNA binding | nucleus / nucleus / nucleus |
| 1438781_at | 0.04 | -6.44 | -0.65 | -4.21 | -1.52 | 0.85 | -0.78 | -3.35 | Tet2 | Tet oncogene family member 2 | --- | --- | --- |
| 1421943_at | 0.10 | -6.06 | -0.57 | 0.41 | -0.47 | -1.06 | -0.66 | 1.51 | Tgfa | transforming growth factor alpha | activation of MAPK activity / activation of MAPK activity / angiogenesis / cell proliferation / positive regulation of epidermal growth factor receptor activity / positive regulation of epidermal growth factor receptor activity / positive regulation of mitosis / positive regulation of mitosis / positive regulation of epithelial cell proliferation / positive regulation of epithelial cell proliferation | glycoprotein binding / MAP kinase kinase activity / MAP kinase kinase activity / epidermal growth factor receptor activating ligand activity / epidermal growth factor receptor activating ligand activity / growth factor activity / growth factor activity | extracellular region / membrane / integral to membrane / integral to membrane |
| 1423308_at | 0.79 | 1.56 | 0.61 | -0.57 | 0.30 | -4.33 | 0.49 | -1.88 | Tgoln1 / LOC100038890 | trans-golgi network protein / hypothetical protein LOC100038890 | --- | protein binding | Golgi apparatus / Golgi apparatus / trans-Golgi network / plasma membrane / membrane / integral to membrane / trans-Golgi network transport vesicle |
| 1416623_at | -0.07 | -6.35 | 0.08 | -6.14 | 0.36 | -3.88 | 0.79 | 1.18 | Thbs3 | thrombospondin 3 | cell adhesion | structural molecule activity / calcium ion binding / protein binding | extracellular region / extracellular space |
| 1417499_at | 0.79 | 0.94 | 0.68 | -0.21 | 0.86 | 1.68 | 0.67 | -0.36 | Timm13 / Timm10 | translocase of inner mitochondrial membrane 13 homolog (yeast) / translocase of inner mitochondrial membrane 10 homolog (yeast) | protein targeting / protein targeting to mitochondrion / protein targeting to mitochondrion / transport / transport / intracellular protein transport / sensory perception of sound / protein transport / protein transport / protein import into mitochondrial inner membrane / intracellular protein transport across a membrane | protein binding / protein binding / zinc ion binding / zinc ion binding / protein transporter activity / P-P-bond-hydrolysis-driven protein transmembrane transporter activity / metal ion binding | mitochondrion / mitochondrion / mitochondrion / mitochondrial inner membrane / mitochondrial inner membrane / mitochondrial inner membrane presequence translocase complex / membrane / organelle inner membrane / mitochondrial intermembrane space protein transporter complex |
| 1416258_at | -0.82 | 3.31 | -0.48 | -1.16 | -0.21 | -4.99 | 0.11 | -5.73 | Tk1 | thymidine kinase 1 | DNA replication | nucleotide binding / thymidine kinase activity / thymidine kinase activity / ATP binding / kinase activity / transferase activity | cytoplasm |
| 1423295_at | -0.43 | -2.53 | -0.65 | 0.53 | -0.27 | -4.49 | -0.25 | -4.54 | Tm9sf2 | transmembrane 9 superfamily member 2 | --- | --- | extracellular space / endosome / endosome membrane / membrane / integral to membrane / integral to membrane |
| 1435258_at | -0.02 | -6.44 | 0.08 | -6.05 | 0.37 | -2.34 | 0.65 | 1.78 | Tmem141 | transmembrane protein 141 | --- | --- | membrane / integral to membrane |
| 1424726_at | 0.19 | -4.89 | 0.25 | -3.83 | 0.62 | 2.32 | 0.50 | 0.41 | Tmem150 | transmembrane protein 150 | --- | --- | plasma membrane / membrane / integral to membrane / integral to membrane |
| 1459713_s_at | -0.24 | -5.87 | 0.36 | -5.10 | 1.18 | 1.07 | 0.80 | -1.74 | Tmem16a | transmembrane protein 16A | --- | --- | cytoplasm / plasma membrane / membrane / integral to membrane / integral to membrane |
| 1425603_at | -0.24 | -4.44 | -0.51 | -0.26 | -0.68 | 2.12 | -0.33 | -2.95 | Tmem176a | transmembrane protein 176A | --- | --- | membrane / integral to membrane / integral to membrane |
| 1428586_at | -0.41 | -2.10 | -0.61 | 0.99 | -0.35 | -2.94 | -0.21 | -4.71 | Tmem41b | transmembrane protein 41B | --- | --- | membrane / integral to membrane / integral to membrane |
| 1460122_at | -0.74 | 0.50 | -0.42 | -3.12 | -0.69 | 0.06 | -0.33 | -4.02 | Tmem41b | transmembrane protein 41B | --- | --- | membrane / integral to membrane / integral to membrane |
| 1436212_at | -1.34 | 3.83 | -1.16 | 2.47 | -1.26 | 3.22 | -0.74 | -1.14 | Tmem71 | transmembrane protein 71 | --- | --- | membrane / integral to membrane |
| 1420725_at | -0.62 | 1.64 | -0.48 | -0.30 | -0.27 | -3.79 | -0.34 | -2.51 | Tmlhe | trimethyllysine hydroxylase, epsilon | carnitine biosynthetic process / carnitine biosynthetic process | iron ion binding / electron carrier activity / oxidoreductase activity / oxidoreductase activity, acting on single donors with incorporation of molecular oxygen, incorporation of two atoms of oxygen / oxidoreductase activity, acting on single donors with incorporation of molecular oxygen, incorporation of two atoms of oxygen / L-ascorbic acid binding / trimethyllysine dioxygenase activity | mitochondrion / mitochondrion / mitochondrial matrix |
| 1440894_at | -0.73 | 0.42 | -0.34 | -4.01 | 0.36 | -3.84 | -0.34 | -3.89 | Tmtc3 | transmembrane and tetratricopeptide repeat containing 3 | --- | binding | membrane / integral to membrane |
| 1416273_at | 0.95 | 2.36 | 0.04 | -6.25 | 0.10 | -6.12 | -0.09 | -5.93 | Tnfaip2 | tumor necrosis factor, alpha-induced protein 2 | angiogenesis / multicellular organismal development / cell differentiation | --- | --- |
| 1438855_x_at | 1.76 | 7.40 | 0.21 | -5.57 | 0.14 | -5.98 | 0.07 | -6.03 | Tnfaip2 | tumor necrosis factor, alpha-induced protein 2 | angiogenesis / multicellular organismal development / cell differentiation | --- | --- |
| 1433699_at | 1.00 | 4.25 | 0.36 | -3.36 | 0.23 | -4.99 | 0.06 | -6.01 | Tnfaip3 | tumor necrosis factor, alpha-induced protein 3 | ubiquitin cycle / apoptosis / negative regulation of I-kappaB kinase/NF-kappaB cascade | DNA binding / protein binding / protein binding / peptidase activity / cysteine-type peptidase activity / zinc ion binding / hydrolase activity / metal ion binding | nucleus / cytoplasm / cytoplasm |
| 1448147_at | -1.02 | 1.72 | -0.76 | -0.73 | -0.52 | -3.05 | -0.16 | -5.70 | Tnfrsf19 | tumor necrosis factor receptor superfamily, member 19 | --- | receptor activity | extracellular region / extracellular space / plasma membrane / membrane / integral to membrane / integral to membrane |
| 1426095_a_at | -0.27 | -4.46 | -0.25 | -4.56 | -0.61 | 0.36 | -0.26 | -4.31 | Tnfrsf22 | tumor necrosis factor receptor superfamily, member 22 | regulation of apoptosis | receptor activity / receptor activity / protein binding / TRAIL binding | extracellular region / plasma membrane / membrane / integral to membrane / integral to membrane / anchored to membrane |
| 1447522_s_at | -0.62 | 1.36 | -0.60 | 1.02 | -0.34 | -2.97 | -0.29 | -3.45 | Tnks2 | tankyrase, TRF1-interacting ankyrin-related ADP-ribose polymerase 2 | telomere maintenance / telomere maintenance / multicellular organism growth / regulation of multicellular organism growth | NAD+ ADP-ribosyltransferase activity | nucleus |
| 1418726_a_at | -0.86 | 4.30 | -0.83 | 3.91 | -0.52 | -0.15 | -0.63 | 1.42 | Tnnt2 | troponin T2, cardiac | muscle contraction / heart development / regulation of heart contraction / sarcomere organization / atrial cardiac muscle morphogenesis / ventricular cardiac muscle morphogenesis | structural constituent of cytoskeleton | cytoplasm / troponin complex / myofibril / sarcomere |
| 1424967_x_at | -0.78 | 3.64 | -0.57 | 0.80 | -0.41 | -1.64 | -0.57 | 0.76 | Tnnt2 | troponin T2, cardiac | muscle contraction / heart development / regulation of heart contraction / sarcomere organization / atrial cardiac muscle morphogenesis / ventricular cardiac muscle morphogenesis | structural constituent of cytoskeleton | cytoplasm / troponin complex / myofibril / sarcomere |
| 1435055_a_at | 0.32 | -2.81 | 0.66 | 2.77 | 0.35 | -2.09 | 0.52 | 0.68 | Tom1 / EG545878 | target of myb1 homolog (chicken) / predicted gene, EG545878 | transport / intracellular protein transport / protein transport | protein binding / protein binding | intracellular / cytoplasm / early endosome / cytosol / membrane / membrane |
| 1421998_at | 0.71 | 3.72 | 0.02 | -6.26 | 0.01 | -6.30 | 0.26 | -3.44 | Tor3a / LOC100047963 | torsin family 3, member A / similar to ADIR1 | aromatic amino acid family biosynthetic process / chaperone cofactor-dependent protein folding | nucleotide binding / 3-dehydroquinate dehydratase activity / ATP binding | cytoplasm / endoplasmic reticulum |
| 1428660_s_at | 0.64 | 3.52 | 0.25 | -3.39 | -0.05 | -6.16 | 0.24 | -3.54 | Tor3a / LOC100047963 | torsin family 3, member A / similar to ADIR1 | aromatic amino acid family biosynthetic process / chaperone cofactor-dependent protein folding | nucleotide binding / 3-dehydroquinate dehydratase activity / ATP binding | cytoplasm / endoplasmic reticulum |
| 1425388_a_at | -0.73 | 4.40 | -0.08 | -5.96 | -0.19 | -4.68 | -0.17 | -4.67 | Tpk1 | thiamine pyrophosphokinase | thiamin metabolic process / thiamin metabolic process / thiamin diphosphate biosynthetic process | nucleotide binding / thiamin diphosphokinase activity / thiamin diphosphokinase activity / ATP binding / kinase activity / transferase activity | --- |
| 1423602_at | 1.98 | 7.19 | 0.22 | -5.68 | 0.28 | -5.35 | 0.10 | -5.96 | Traf1 | Tnf receptor-associated factor 1 | apoptosis / signal transduction / regulation of apoptosis | receptor activity / protein binding / protein binding / zinc ion binding | cytoplasm |
| 1448861_at | -0.91 | 0.04 | -0.49 | -3.74 | -0.51 | -3.58 | -0.35 | -4.65 | Traf5 | Tnf receptor-associated factor 5 | apoptosis / signal transduction / signal transduction / positive regulation of cell proliferation / regulation of apoptosis / positive regulation of I-kappaB kinase/NF-kappaB cascade | signal transducer activity / signal transducer activity / receptor activity / protein binding / zinc ion binding / metal ion binding | cytoplasm |
| 1426065_a_at | 2.47 | 13.80 | 2.23 | 12.18 | 1.73 | 10.36 | 1.28 | 7.11 | Trib3 | tribbles homolog 3 (Drosophila) | transcription / regulation of transcription, DNA-dependent / protein amino acid phosphorylation / negative regulation of protein kinase activity / apoptosis / regulation of MAP kinase activity | transcription corepressor activity / protein kinase activity / protein kinase inhibitor activity / protein binding / protein binding / ATP binding / kinase activity / protein kinase binding / protein kinase binding | nucleus / nucleus |
| 1456225_x_at | 2.94 | 12.93 | 2.22 | 9.96 | 2.18 | 9.91 | 1.88 | 7.92 | Trib3 | tribbles homolog 3 (Drosophila) | transcription / regulation of transcription, DNA-dependent / protein amino acid phosphorylation / negative regulation of protein kinase activity / apoptosis / regulation of MAP kinase activity | transcription corepressor activity / protein kinase activity / protein kinase inhibitor activity / protein binding / protein binding / ATP binding / kinase activity / protein kinase binding / protein kinase binding | nucleus / nucleus |
| 1417027_at | -0.84 | 2.25 | -0.36 | -3.63 | 0.11 | -6.02 | -0.44 | -2.47 | Trim2 | tripartite motif-containing 2 | --- | protein binding / protein binding / zinc ion binding / zinc ion binding / myosin binding / metal ion binding | intracellular / cytoplasm / cytoplasm |
| 1427258_at | -0.47 | -1.89 | -0.39 | -2.88 | -0.50 | -1.46 | -0.62 | 0.20 | Trim24 | tripartite motif-containing 24 | transcription / regulation of transcription, DNA-dependent | DNA binding / protein binding / protein binding / zinc ion binding / ligand-dependent nuclear receptor binding / metal ion binding | intracellular / nucleus / cytoplasm |
| 1436393_a_at | -0.34 | -2.78 | 0.24 | -4.16 | 0.64 | 2.09 | 0.27 | -3.66 | Trim37 | tripartite motif-containing 37 | --- | protein binding / zinc ion binding / zinc ion binding / metal ion binding | intracellular / intracellular / cytoplasm / peroxisome |
| 1436394_at | 0.12 | -5.88 | 0.11 | -5.79 | 0.72 | 2.87 | 0.40 | -1.67 | Trim37 | tripartite motif-containing 37 | --- | protein binding / zinc ion binding / zinc ion binding / metal ion binding | intracellular / intracellular / cytoplasm / peroxisome |
| 1423899_at | -0.65 | 0.20 | -0.62 | -0.11 | -0.41 | -2.80 | -0.99 | 3.81 | Trip12 | thyroid hormone receptor interactor 12 | protein modification process / protein modification process / ubiquitin cycle / ubiquitin cycle | ubiquitin-protein ligase activity / ubiquitin-protein ligase activity / receptor activity / binding / ligase activity | intracellular / intracellular |
| 1416926_at | 0.81 | 3.62 | 0.61 | 0.99 | 1.03 | 5.96 | 0.32 | -3.21 | Trp53inp1 | transformation related protein 53 inducible nuclear protein 1 | apoptosis / apoptosis / induction of apoptosis / response to stress / cell cycle arrest | --- | nucleus / nucleus / nucleolus / cytoplasm |
| 1416927_at | 0.58 | -0.91 | 0.58 | -0.85 | 0.79 | 1.63 | 0.17 | -5.39 | Trp53inp1 | transformation related protein 53 inducible nuclear protein 1 | apoptosis / apoptosis / induction of apoptosis / response to stress / cell cycle arrest | --- | nucleus / nucleus / nucleolus / cytoplasm |
| 1421096_at | 0.02 | -6.44 | 0.00 | -6.28 | -0.67 | 0.07 | -0.38 | -3.28 | Trpc1 | transient receptor potential cation channel, subfamily C, member 1 | transport / ion transport / calcium ion transport | ion channel activity / calcium channel activity / calcium ion binding / protein binding | membrane / integral to membrane / integral to membrane / protein complex / membrane raft |
| 1438877_at | -0.66 | 2.13 | -0.35 | -2.60 | -0.43 | -1.21 | -0.34 | -2.55 | Trpm6 | transient receptor potential cation channel, subfamily M, member 6 | protein amino acid phosphorylation / transport / ion transport / calcium ion transport / metal ion transport | nucleotide binding / protein serine/threonine kinase activity / ion channel activity / cation channel activity / calcium channel activity / calcium ion binding / ATP binding / zinc ion binding / kinase activity / transferase activity / metal ion binding | membrane / integral to membrane / apical plasma membrane / brush border membrane |
| 1416483_at | -0.35 | -3.89 | -0.21 | -5.24 | -0.69 | 0.43 | -0.43 | -2.65 | Ttc3 | tetratricopeptide repeat domain 3 | --- | binding / protein binding / zinc ion binding / metal ion binding | --- |
| 1424410_at | -0.94 | 5.16 | -0.38 | -2.33 | -0.38 | -2.36 | -0.37 | -2.42 | Ttc8 | tetratricopeptide repeat domain 8 | cell projection organization and biogenesis | binding | centrosome |
| 1455868_a_at | -0.60 | 0.63 | -0.17 | -5.36 | -0.15 | -5.52 | -0.02 | -6.08 | Tubgcp2 | tubulin, gamma complex associated protein 2 | microtubule cytoskeleton organization and biogenesis | --- | spindle pole / centrosome / microtubule organizing center / microtubule |
| 1421529_a_at | 0.39 | -3.84 | 0.76 | 0.48 | 0.71 | 0.00 | 0.28 | -4.65 | Txnrd1 | thioredoxin reductase 1 | mesoderm formation / response to oxidative stress / gastrulation / cell proliferation / hydrogen peroxide catabolic process / cell redox homeostasis / oxidation reduction | thioredoxin-disulfide reductase activity / thioredoxin-disulfide reductase activity / selenium binding / electron carrier activity / oxidoreductase activity / oxidoreductase activity, acting on NADH or NADPH, disulfide as acceptor / FAD binding / NADP binding | nucleus / cytoplasm / cytoplasm / cytosol / cytosol |
| 1428689_at | -0.03 | -6.42 | 0.11 | -5.89 | 0.63 | 0.50 | 0.38 | -2.73 | Tysnd1 | trypsin domain containing 1 | proteolysis | catalytic activity / serine-type endopeptidase activity / peptidase activity / serine-type peptidase activity / hydrolase activity | peroxisome |
| 1416743_at | 0.03 | -6.42 | -0.26 | -4.61 | -0.47 | -1.97 | -0.72 | 1.17 | Uap1 / LOC640502 | UDP-N-acetylglucosamine pyrophosphorylase 1 / similar to UDP-N-acetylhexosamine pyrophosphorylase | metabolic process / metabolic process | UDP-N-acetylglucosamine diphosphorylase activity / transferase activity / nucleotidyltransferase activity / nucleotidyltransferase activity | cytoplasm |
| 1437490_x_at | -0.03 | -6.43 | -0.41 | -2.56 | -0.64 | 0.46 | -0.75 | 1.77 | Uap1 / LOC640502 | UDP-N-acetylglucosamine pyrophosphorylase 1 / similar to UDP-N-acetylhexosamine pyrophosphorylase | metabolic process / metabolic process | UDP-N-acetylglucosamine diphosphorylase activity / transferase activity / nucleotidyltransferase activity / nucleotidyltransferase activity | cytoplasm |
| 1456516_x_at | 0.03 | -6.42 | -0.48 | -1.79 | -0.60 | -0.18 | -0.64 | 0.41 | Uap1 / LOC640502 | UDP-N-acetylglucosamine pyrophosphorylase 1 / similar to UDP-N-acetylhexosamine pyrophosphorylase | metabolic process / metabolic process | UDP-N-acetylglucosamine diphosphorylase activity / transferase activity / nucleotidyltransferase activity / nucleotidyltransferase activity | cytoplasm |
| 1436890_at | 0.05 | -6.35 | 0.38 | -2.23 | 0.59 | 0.95 | 0.52 | -0.06 | Uap1l1 | UDP-N-acteylglucosamine pyrophosphorylase 1-like 1 | metabolic process | transferase activity / nucleotidyltransferase activity | --- |
| 1426971_at | -0.28 | -5.02 | -0.36 | -4.14 | -0.83 | 0.97 | -0.38 | -3.77 | Ube1l | ubiquitin-activating enzyme E1-like | protein modification process / ubiquitin cycle / metabolic process / modification-dependent protein catabolic process / ISG15-protein conjugation | catalytic activity / binding / small protein activating enzyme activity / ISG15 activating enzyme activity | --- |
| 1426460_a_at | -1.15 | 6.83 | -0.74 | 2.51 | -0.45 | -1.56 | -0.41 | -1.94 | Ugp2 | UDP-glucose pyrophosphorylase 2 | regulation of transcription, DNA-dependent / metabolic process / metabolic process | transcription factor activity / UTP:glucose-1-phosphate uridylyltransferase activity / transferase activity / nucleotidyltransferase activity / nucleotidyltransferase activity | cytoplasm |
| 1426461_at | -0.98 | 5.52 | -0.63 | 1.30 | -0.50 | -0.61 | -0.37 | -2.37 | Ugp2 | UDP-glucose pyrophosphorylase 2 | regulation of transcription, DNA-dependent / metabolic process / metabolic process | transcription factor activity / UTP:glucose-1-phosphate uridylyltransferase activity / transferase activity / nucleotidyltransferase activity / nucleotidyltransferase activity | cytoplasm |
| 1434485_a_at | -0.82 | 3.41 | -0.52 | -0.57 | -0.59 | 0.37 | -0.29 | -3.75 | Ugp2 | UDP-glucose pyrophosphorylase 2 | regulation of transcription, DNA-dependent / metabolic process / metabolic process | transcription factor activity / UTP:glucose-1-phosphate uridylyltransferase activity / transferase activity / nucleotidyltransferase activity / nucleotidyltransferase activity | cytoplasm |
| 1434486_x_at | -0.66 | 1.08 | -0.47 | -1.50 | -0.50 | -1.09 | -0.26 | -4.29 | Ugp2 | UDP-glucose pyrophosphorylase 2 | regulation of transcription, DNA-dependent / metabolic process / metabolic process | transcription factor activity / UTP:glucose-1-phosphate uridylyltransferase activity / transferase activity / nucleotidyltransferase activity / nucleotidyltransferase activity | cytoplasm |
| 1451742_a_at | -0.80 | 4.58 | -0.54 | 0.86 | -0.47 | -0.16 | -0.26 | -3.53 | Ugp2 | UDP-glucose pyrophosphorylase 2 | regulation of transcription, DNA-dependent / metabolic process / metabolic process | transcription factor activity / UTP:glucose-1-phosphate uridylyltransferase activity / transferase activity / nucleotidyltransferase activity / nucleotidyltransferase activity | cytoplasm |
| 1427961_s_at | -0.89 | 2.71 | -0.73 | 1.03 | -0.62 | -0.27 | -0.36 | -3.47 | Ugt2b34 | UDP glucuronosyltransferase 2 family, polypeptide B34 | metabolic process / metabolic process | transferase activity / transferase activity, transferring glycosyl groups / transferase activity, transferring hexosyl groups / transferase activity, transferring hexosyl groups | --- |
| 1439624_at | -1.04 | 4.38 | -0.77 | 1.54 | -0.68 | 0.50 | -0.40 | -2.98 | Ugt2b35 | UDP glucuronosyltransferase 2 family, polypeptide B35 | metabolic process | glucuronosyltransferase activity / transferase activity / transferase activity, transferring glycosyl groups / transferase activity, transferring hexosyl groups | --- |
| 1423513_at | 0.26 | -4.17 | 0.28 | -3.76 | 0.61 | 1.23 | 0.25 | -4.08 | Uhrf1bp1l | UHRF1 (ICBP90) binding protein 1-like | protein amino acid phosphorylation / cell adhesion / myoblast fusion / regulation of transcription | receptor activity / vascular endothelial growth factor receptor activity / protein binding / protein binding / ATP binding / transcription regulator activity / cadherin binding | plasma membrane / membrane / integral to membrane |
| 1417847_at | -0.71 | 1.20 | -0.45 | -2.05 | -0.28 | -4.41 | -0.49 | -1.52 | Ulk2 | Unc-51 like kinase 2 (C. elegans) | protein amino acid phosphorylation / signal transduction / axonogenesis / negative regulation of collateral sprouting / axon extension | nucleotide binding / protein kinase activity / protein serine/threonine kinase activity / protein binding / ATP binding / kinase activity / transferase activity | --- |
| 1418191_at | -0.69 | 1.91 | -0.23 | -4.60 | 0.03 | -6.27 | 0.14 | -5.43 | Usp18 / LOC100048346 | ubiquitin specific peptidase 18 / similar to ubiquitin specific protease UBP43 | ubiquitin-dependent protein catabolic process / ubiquitin cycle / ISG15-protein conjugation | ubiquitin thiolesterase activity / peptidase activity / cysteine-type peptidase activity / hydrolase activity / ISG15-specific protease activity | --- |
| 1458079_at | -0.02 | -6.44 | -0.09 | -6.08 | -0.70 | 0.26 | 0.07 | -5.98 | Usp40 | ubiquitin specific peptidase 40 | ubiquitin-dependent protein catabolic process / ubiquitin cycle | ubiquitin thiolesterase activity / peptidase activity / cysteine-type peptidase activity / hydrolase activity | --- |
| 1416231_at | -0.18 | -5.50 | 0.21 | -4.97 | 0.59 | 0.10 | 0.50 | -1.02 | Vac14 | Vac14 homolog (S. cerevisiae) | response to osmotic stress | binding / kinase activator activity | vacuolar membrane |
| 1415989_at | 1.64 | 6.70 | -0.27 | -5.14 | -0.18 | -5.76 | -0.27 | -5.01 | Vcam1 | vascular cell adhesion molecule 1 | cell adhesion / cell adhesion / heterophilic cell adhesion / leukocyte adhesion / cell-cell adhesion | protein binding | extracellular space / plasma membrane / membrane / integral to membrane / integral to membrane / anchored to membrane |
| 1436003_at | 1.28 | 2.11 | 0.28 | -5.50 | -0.08 | -6.25 | -0.01 | -6.10 | Vcam1 | Vascular cell adhesion molecule 1 | cell adhesion / cell adhesion / heterophilic cell adhesion / leukocyte adhesion / cell-cell adhesion | protein binding | extracellular space / plasma membrane / membrane / integral to membrane / integral to membrane / anchored to membrane |
| 1448162_at | 1.83 | 7.83 | -0.41 | -3.93 | -0.41 | -3.99 | -0.39 | -3.99 | Vcam1 | vascular cell adhesion molecule 1 | cell adhesion / cell adhesion / heterophilic cell adhesion / leukocyte adhesion / cell-cell adhesion | protein binding | extracellular space / plasma membrane / membrane / integral to membrane / integral to membrane / anchored to membrane |
| 1451314_a_at | 1.09 | 3.78 | 0.75 | 0.40 | 0.21 | -5.45 | -0.17 | -5.49 | Vcam1 | vascular cell adhesion molecule 1 | cell adhesion / cell adhesion / heterophilic cell adhesion / leukocyte adhesion / cell-cell adhesion | protein binding | extracellular space / plasma membrane / membrane / integral to membrane / integral to membrane / anchored to membrane |
| 1448837_at | -0.66 | -0.45 | -0.56 | -1.60 | -0.70 | 0.06 | -0.47 | -2.50 | Vil1 | villin 1 | cytoskeleton organization and biogenesis / barbed-end actin filament capping | actin binding / calcium ion binding / protein binding | cytoplasm / cytoskeleton / brush border |
| 1447845_s_at | 0.57 | -2.37 | 0.85 | 0.51 | 0.09 | -6.18 | 0.15 | -5.75 | Vnn1 | vanin 1 | nitrogen compound metabolic process | hydrolase activity / hydrolase activity, acting on carbon-nitrogen (but not peptide) bonds / hydrolase activity, acting on carbon-nitrogen (but not peptide) bonds, in linear amides / pantetheine hydrolase activity | extracellular space / plasma membrane / membrane / anchored to membrane |
| 1421051_s_at | -0.67 | 0.75 | -0.17 | -5.46 | 0.01 | -6.31 | 0.20 | -5.03 | Vps25 | vacuolar protein sorting 25 (yeast) | transcription / regulation of transcription, DNA-dependent / transport / protein transport | --- | nucleus / cytoplasm / mitochondrion |
| 1424380_at | 0.94 | 4.89 | 0.78 | 3.02 | 0.35 | -2.98 | 0.46 | -1.27 | Vps37b | vacuolar protein sorting 37B (yeast) | transport / protein transport | --- | cytoplasm / endosome / membrane / late endosome membrane |
| 1423962_at | -0.36 | -2.76 | 0.03 | -6.25 | -0.21 | -4.83 | -0.89 | 4.39 | Wdr26 | WD repeat domain 26 | --- | --- | cytoplasm |
| 1424849_at | -0.13 | -5.80 | 0.04 | -6.22 | 0.64 | 2.00 | 0.30 | -3.19 | Wdr62 | WD repeat domain 62 | --- | --- | --- |
| 1416890_at | 0.19 | -5.39 | 0.34 | -3.32 | 0.66 | 1.01 | 0.44 | -1.89 | Wdr74 | WD repeat domain 74 | --- | --- | nucleus |
| 1423808_at | 0.04 | -6.39 | 0.17 | -5.37 | 0.63 | 0.96 | 0.44 | -1.65 | Wdr79 | WD repeat domain 79 | --- | --- | --- |
| 1436791_at | -0.53 | -2.24 | -0.47 | -2.88 | -0.85 | 1.36 | -0.90 | 1.84 | Wnt5a | wingless-related MMTV integration site 5A | signal transduction / Wnt receptor signaling pathway, calcium modulating pathway / JNK cascade / cell-cell signaling / multicellular organismal development / embryonic development / organ morphogenesis / Wnt receptor signaling pathway / lung development / embryonic limb morphogenesis | signal transducer activity / receptor binding / protein binding | extracellular region / proteinaceous extracellular matrix / extracellular space |
| 1437223_s_at | -0.15 | -5.71 | -0.34 | -3.11 | -0.68 | 1.63 | -0.61 | 0.79 | Xbp1 | X-box binding protein 1 | transcription / regulation of transcription, DNA-dependent | DNA binding / transcription factor activity / sequence-specific DNA binding / protein dimerization activity | extracellular space / nucleus |
| 1424602_s_at | -0.65 | 0.92 | -0.22 | -4.85 | -0.35 | -3.16 | 0.03 | -6.07 | Xrcc4 | X-ray repair complementing defective repair in Chinese hamster cells 4 | in utero embryonic development / pro-B cell differentiation / DNA ligation / DNA repair / double-strand break repair / double-strand break repair / double-strand break repair via nonhomologous end joining / DNA recombination / response to DNA damage stimulus / central nervous system development / response to X-ray / response to ionizing radiation / response to gamma radiation / T cell differentiation in the thymus / V(D)J recombination / immunoglobulin V(D)J recombination / negative regulation of neuron apoptosis / isotype switching / positive regulation of fibroblast proliferation / positive regulation of neurogenesis | DNA binding / protein binding | nucleus / DNA-dependent protein kinase complex / DNA-dependent protein kinase complex |
| 1453156_s_at | -0.60 | 2.00 | -0.01 | -6.28 | 0.08 | -5.98 | -0.05 | -5.97 | Zadh1 | zinc binding alcohol dehydrogenase, domain containing 1 | metabolic process | catalytic activity / binding / zinc ion binding / oxidoreductase activity | cytoplasm |
| 1444402_at | 0.74 | 1.86 | 0.07 | -6.11 | -0.19 | -5.29 | -0.23 | -4.68 | Zc3h12c | zinc finger CCCH type containing 12C | --- | --- | --- |
| 1418495_at | 0.71 | 0.24 | 0.28 | -4.65 | 0.49 | -2.32 | 0.21 | -5.17 | Zc3h8 | zinc finger CCCH type containing 8 | --- | nucleic acid binding / zinc ion binding / metal ion binding | nucleus / nucleus |
| 1434401_at | 0.48 | -1.57 | 0.37 | -3.05 | 0.64 | 0.70 | 0.23 | -4.68 | Zcchc2 | zinc finger, CCHC domain containing 2 | cell communication | nucleic acid binding / nucleic acid binding / protein binding / zinc ion binding / phosphoinositide binding / metal ion binding | cytoplasm |
| 1418408_at | -0.60 | 0.42 | -0.21 | -4.92 | -0.04 | -6.26 | -0.03 | -6.07 | Zfand1 | zinc finger, AN1-type domain 1 | --- | zinc ion binding / metal ion binding | --- |
| 1415940_at | 1.11 | 1.89 | 1.12 | 1.98 | 0.62 | -2.43 | 0.66 | -1.92 | Zfand2a | zinc finger, AN1-type domain 2A | --- | zinc ion binding / metal ion binding | nucleus / cytoplasm |
| 1415941_s_at | 1.30 | 1.75 | 0.85 | -1.55 | 0.62 | -3.34 | 0.78 | -1.99 | Zfand2a | zinc finger, AN1-type domain 2A | --- | zinc ion binding / metal ion binding | nucleus / cytoplasm |
| 1449702_at | 1.12 | 2.37 | 1.08 | 2.08 | 0.70 | -1.40 | 0.68 | -1.47 | Zfand2a | zinc finger, AN1-type domain 2A | --- | zinc ion binding / metal ion binding | nucleus / cytoplasm |
| 1419165_at | -0.68 | 0.20 | -0.33 | -4.09 | -0.15 | -5.80 | -0.36 | -3.61 | Zfp260 | zinc finger protein 260 | transcription / regulation of transcription, DNA-dependent / multicellular organismal development | nucleic acid binding / DNA binding / zinc ion binding / metal ion binding | intracellular / nucleus |
| 1449231_at | 0.44 | -0.78 | 0.60 | 1.85 | 0.84 | 4.98 | 0.51 | 0.36 | Zfp296 | zinc finger protein 296 | --- | nucleic acid binding / zinc ion binding | intracellular |
| 1452376_at | 0.09 | -6.31 | 0.16 | -5.80 | 0.79 | 0.36 | 0.27 | -4.90 | Zfp444 | zinc finger protein 444 | transcription / regulation of transcription, DNA-dependent | nucleic acid binding / transcription factor activity / zinc ion binding / metal ion binding | intracellular / nucleus |
| 1456789_at | -0.52 | -1.64 | -0.38 | -3.30 | -0.12 | -5.95 | -0.66 | 0.15 | Zfp462 | zinc finger protein 462 | negative regulation of DNA binding / positive regulation of transcription from RNA polymerase II promoter | nucleic acid binding / zinc ion binding | intracellular / nucleus |
| 1441727_s_at | -1.37 | 2.12 | -0.68 | -2.99 | -0.95 | -0.90 | -0.38 | -4.85 | Zfp467 | zinc finger protein 467 | transcription / regulation of transcription, DNA-dependent / regulation of transcription | nucleic acid binding / DNA binding / DNA binding / protein binding / zinc ion binding / metal ion binding | intracellular / nucleus / nucleus |
| 1459929_at | 0.38 | -4.14 | 0.26 | -5.11 | 0.83 | 0.78 | 0.16 | -5.65 | Zfp568 | zinc finger protein 568 | regulation of transcription, DNA-dependent | nucleic acid binding / zinc ion binding / metal ion binding | intracellular / nucleus |
| 1449946_a_at | 0.29 | -3.79 | 0.27 | -4.04 | 0.64 | 1.48 | 0.29 | -3.52 | Zfp593 | zinc finger protein 593 | transcription / regulation of transcription, DNA-dependent | nucleic acid binding / DNA binding / zinc ion binding / metal ion binding | intracellular / nucleus |
| 1428341_at | 0.59 | 0.92 | 0.48 | -0.75 | 0.56 | 0.50 | 0.33 | -2.96 | Zfp655 | zinc finger protein 655 | regulation of transcription, DNA-dependent | nucleic acid binding / zinc ion binding | intracellular |
| 1437925_at | -0.02 | -6.44 | 0.71 | 1.38 | -0.27 | -4.41 | -0.26 | -4.38 | Zfp787 | Zinc finger protein 787 | transcription / regulation of transcription, DNA-dependent | nucleic acid binding / DNA binding / zinc ion binding / metal ion binding | intracellular / nucleus |
| 1429517_at | -0.05 | -6.41 | 0.25 | -5.38 | 0.85 | 0.03 | 0.51 | -3.06 | Zfyve20 | zinc finger, FYVE domain containing 20 | transport / protein transport | nucleic acid binding / zinc ion binding / zinc ion binding / metal ion binding | intracellular / endosome / plasma membrane / membrane / early endosome membrane |
| 1451189_at | 0.08 | -6.15 | 0.11 | -5.66 | 0.61 | 2.46 | 0.33 | -2.04 | Zswim1 | zinc finger, SWIM domain containing 1 | --- | zinc ion binding / metal ion binding | nucleus |
