# Supplementary material for: Host cell transcriptional profiling during malaria liver stage infection reveals a coordinated and sequential set of biological events
Source: BMC Genomics. 2009 Jun 17;10:270. doi: 10.1186/1471-2164-10-270 (PMC2706893; doi:10.1186/1471-2164-10-270)
Supplement: Additional file 2 — List of DE genes at all time in P. berghei ANKA infected cells compared to non-infected ones. The data provided represent 24 DE genes at all time in P. berghei ANKA infected cells compared to non-infected ones. The annotation was obtained from NetAffx Centre and Ensembl . The rows highlighted in grey indicate an up-regulation of a gene at all the time points assessed. [file 1471-2164-10-270-S2.doc]

| **Additional File 2**: 27 DE genes at all time in *P. berghei* ANKA infected cells compared to non-infected ones. The annotation was obtained from NetAffx Centre (www.affymetrix.com) and Ensembl (www.ensembl.org). The rows highlighted in grey indicate an up-regulation of this gene at all times. | | | | | |  |
| --- | --- | --- | --- | --- | --- | --- |
| **Gene Symbol** | **Gene Title** | **Pathway** | **go biological process term** | **go molecular function term** | **go cellular component term** | **expression** |
| 1110020G09Rik | RIKEN cDNA 1110020G09 gene | --- | metabolic process | NAD+ kinase activity | mitochondrion | DOWN |
| 5033414K04Rik | RIKEN cDNA 5033414K04 gene | --- | --- | --- | cytoplasm | DOWN |
| A130090K04Rik | RIKEN cDNA A130090K04 gene | --- | --- | --- | cytoplasm /// plasma membrane /// membrane | DOWN |
| Aars | alanyl-tRNA synthetase | --- | translation /// alanyl-tRNA aminoacylation /// tRNA aminoacylation | nucleotide binding /// nucleic acid binding /// aminoacyl-tRNA ligase activity /// alanine-tRNA ligase activity /// ATP binding /// ligase activity /// ligase activity, forming aminoacyl-tRNA and related compounds | cytoplasm | UP |
| Abcd2 | ATP-binding cassette, sub-family D (ALD), member 2 | --- | transport /// transport | nucleotide binding /// nucleotide binding /// protein binding /// protein binding /// ATP binding /// ATPase activity /// nucleoside-triphosphatase activity /// ATPase activity, coupled to transmembrane movement of substances | peroxisome /// peroxisomal membrane /// membrane /// membrane /// integral to membrane | DOWN |
| Calcrl | calcitonin receptor-like | GPCRDB_Class_B_Secretin-like | signal transduction /// G-protein coupled receptor protein signaling pathway /// G-protein coupled receptor protein signaling pathway /// G-protein signaling, adenylate cyclase activating pathway /// heart development /// positive regulation of cell proliferation /// positive regulation of smooth muscle cell proliferation | calcitonin gene-related polypeptide receptor activity /// signal transducer activity /// receptor activity /// G-protein coupled receptor activity /// G-protein coupled receptor activity /// calcitonin receptor activity | extracellular space /// plasma membrane /// membrane /// membrane /// integral to membrane /// integral to membrane | DOWN |
| Cebpb | CCAAT/enhancer binding protein (C/EBP), beta | Circadian_Exercise /// Ovarian_Infertility_Genes | embryonic placenta development /// transcription /// regulation of transcription, DNA-dependent /// anti-apoptosis /// induction of apoptosis /// neuron differentiation /// regulation of interleukin-6 biosynthetic process /// fat cell differentiation /// positive regulation of transcription /// positive regulation of transcription from RNA polymerase II promoter | DNA binding /// DNA binding /// transcription factor activity /// RNA polymerase II transcription factor activity, enhancer binding /// protein binding /// transcription activator activity /// protein homodimerization activity /// protein homodimerization activity /// sequence-specific DNA binding /// sequence-specific DNA binding /// protein heterodimerization activity /// protein heterodimerization activity /// protein dimerization activity | nucleus /// nucleus /// cytoplasm | UP |
| Chac1 | ChaC, cation transport regulator-like 1 (E. coli) | --- | --- | --- | --- | UP |
| Cyb5r1 | cytochrome b5 reductase 1 | --- | steroid biosynthetic process /// lipid biosynthetic process /// sterol biosynthetic process | cytochrome-b5 reductase activity /// electron carrier activity /// oxidoreductase activity /// oxidoreductase activity | membrane /// integral to membrane | UP |
| Ddit3 | DNA-damage inducible transcript 3 | --- | response to amphetamine /// transcription /// regulation of transcription, DNA-dependent /// regulation of transcription, DNA-dependent /// response to oxidative stress /// ER overload response /// ER overload response /// cell cycle /// cell cycle arrest /// aging /// response to nutrient /// regulation of cell redox homeostasis /// regulation of cell redox homeostasis /// unfolded protein response /// negative regulation of CREB transcription factor activity /// response to drug /// response to hydrogen peroxide /// mRNA transcription from RNA polymerase II promoter /// mRNA transcription from RNA polymerase II promoter /// regulation of apoptosis /// positive regulation of apoptosis /// positive regulation of transcription /// embryonic organ development | DNA binding /// DNA binding /// DNA binding /// transcription factor activity /// transcription factor activity /// protein binding /// sequence-specific DNA binding /// protein dimerization activity | nucleus /// nucleus /// nucleus /// cytoplasm | UP |
| Dpp7 | dipeptidylpeptidase 7 | --- | proteolysis | aminopeptidase activity /// serine-type endopeptidase activity /// protein binding /// peptidase activity /// serine-type peptidase activity /// hydrolase activity | extracellular space /// lysosome | UP |
| Esd | esterase D/formylglutathione hydrolase | --- | --- | catalytic activity /// carboxylesterase activity /// carboxylesterase activity /// hydrolase activity /// hydrolase activity /// hydrolase activity, acting on ester bonds /// S-formylglutathione hydrolase activity | cytoplasm /// cytoplasmic membrane-bounded vesicle /// cytoplasmic vesicle | UP |
| Gpr137b-ps | G protein-coupled receptor 137B, pseudogene | --- | --- | --- | membrane /// integral to membrane /// integral to membrane | UP |
| Gpr155 | G protein-coupled receptor 155 | --- | intracellular signaling cascade | receptor activity | integral to membrane | DOWN |
| Kif5c | Kinesin family member 5C | --- | microtubule-based process /// microtubule-based movement /// motor axon guidance | nucleotide binding /// motor activity /// microtubule motor activity /// microtubule motor activity /// protein binding /// ATP binding | cytoplasm /// kinesin complex /// kinesin complex /// microtubule /// microtubule associated complex /// ciliary rootlet /// neuron projection | DOWN |
| Klf4 | Kruppel-like factor 4 (gut) | --- | transcription /// regulation of transcription, DNA-dependent /// negative regulation of cell proliferation /// epidermal cell differentiation /// stem cell maintenance /// cell differentiation /// post-embryonic camera-type eye development /// negative regulation of transcription, DNA-dependent /// negative regulation of transcription, DNA-dependent /// positive regulation of transcription from RNA polymerase II promoter /// epidermis morphogenesis | nucleic acid binding /// DNA binding /// DNA binding /// transcription factor activity /// zinc ion binding /// transcription repressor activity /// transcription repressor activity /// sequence-specific DNA binding /// metal ion binding | intracellular /// nucleus /// nucleus | UP |
| Maf | avian musculoaponeurotic fibrosarcoma (v-maf) AS42 oncogene homolog | --- | cytokine production /// transcription /// regulation of transcription, DNA-dependent /// cellular process /// regulation of chondrocyte differentiation /// regulation of transcription /// regulation of transcription /// positive regulation of transcription from RNA polymerase II promoter /// regulation of cell cycle | DNA binding /// DNA binding /// transcription factor activity /// protein binding /// sequence-specific DNA binding /// protein dimerization activity | nucleus /// nucleus /// cytoplasm | DOWN |
| Nr4a2 | nuclear receptor subfamily 4, group A, member 2 | Nuclear_Receptors | transcription /// regulation of transcription, DNA-dependent /// nervous system development /// neuron differentiation /// regulation of dopamine metabolic process /// positive regulation of transcription from RNA polymerase II promoter /// positive regulation of transcription from RNA polymerase II promoter | DNA binding /// DNA binding /// transcription factor activity /// steroid hormone receptor activity /// receptor activity /// ligand-dependent nuclear receptor activity /// ligand-dependent nuclear receptor activity /// protein binding /// zinc ion binding /// sequence-specific DNA binding /// metal ion binding | nucleus /// nucleus | UP |
| Nrp | neural regeneration protein | --- | in utero embryonic development /// neuron migration /// placenta development /// regulation of gene expression /// cell differentiation /// neurite development /// neuroprotection /// developmental growth | protein binding /// chemoattractant activity | nuclear euchromatin | UP |
| **Nupr1** | nuclear protein 1 | --- | cell growth |  |  | UP |
| P2ry12 | purinergic receptor P2Y, G-protein coupled 12 | GPCRDB_Class_A_Rhodopsin-like | signal transduction /// G-protein coupled receptor protein signaling pathway /// G-protein signaling, coupled to cAMP nucleotide second messenger /// platelet activation | rhodopsin-like receptor activity /// adenosine receptor activity, G-protein coupled /// platelet ADP receptor activity /// signal transducer activity /// receptor activity /// G-protein coupled receptor activity /// purinergic nucleotide receptor activity, G-protein coupled | plasma membrane /// integral to plasma membrane /// membrane /// integral to membrane /// integral to membrane | DOWN |
| Proc | protein C | --- | proteolysis /// blood coagulation /// negative regulation of apoptosis /// negative regulation of apoptosis /// negative regulation of apoptosis | protein C (activated) activity /// catalytic activity /// serine-type endopeptidase activity /// calcium ion binding /// protein binding /// peptidase activity /// hydrolase activity | extracellular region /// extracellular space /// extracellular space | DOWN |
| Ptpn14 | protein tyrosine phosphatase, non-receptor type 14 | --- | protein amino acid dephosphorylation /// dephosphorylation | phosphoprotein phosphatase activity /// protein tyrosine phosphatase activity /// receptor activity /// structural molecule activity /// binding /// hydrolase activity /// phosphoric monoester hydrolase activity | cytoplasm /// cytoskeleton | UP |
| Reep6 | receptor accessory protein 6 | --- | --- | protein binding | membrane /// integral to membrane /// integral to membrane | UP |
| Slc16a4 | Solute carrier family 16 (monocarboxylic acid transporters), member 4 | --- | transport | transporter activity | integral to membrane | DOWN |
| Slc39a8 | solute carrier family 39 (metal ion transporter), member 8 | --- | transport /// ion transport /// zinc ion transport /// metal ion transport /// metal ion transport | zinc ion binding /// metal ion transmembrane transporter activity /// metal ion transmembrane transporter activity | extracellular space /// membrane /// membrane /// integral to membrane /// integral to membrane | DOWN |
| **Trib3** | tribbles homolog 3 (Drosophila) | --- | transcription /// regulation of transcription, DNA-dependent /// protein amino acid phosphorylation /// negative regulation of protein kinase activity /// apoptosis /// regulation of MAP kinase activity | transcription corepressor activity /// protein kinase activity /// protein kinase inhibitor activity /// protein binding /// protein binding /// ATP binding /// kinase activity /// protein kinase binding /// protein kinase binding | nucleus /// nucleus | UP |
