# Supplementary figures and images for: Host cell transcriptional profiling during malaria liver stage infection reveals a coordinated and sequential set of biological events
Source: BMC Genomics. 2009 Jun 17;10:270. doi: 10.1186/1471-2164-10-270 (PMC2706893; doi:10.1186/1471-2164-10-270)

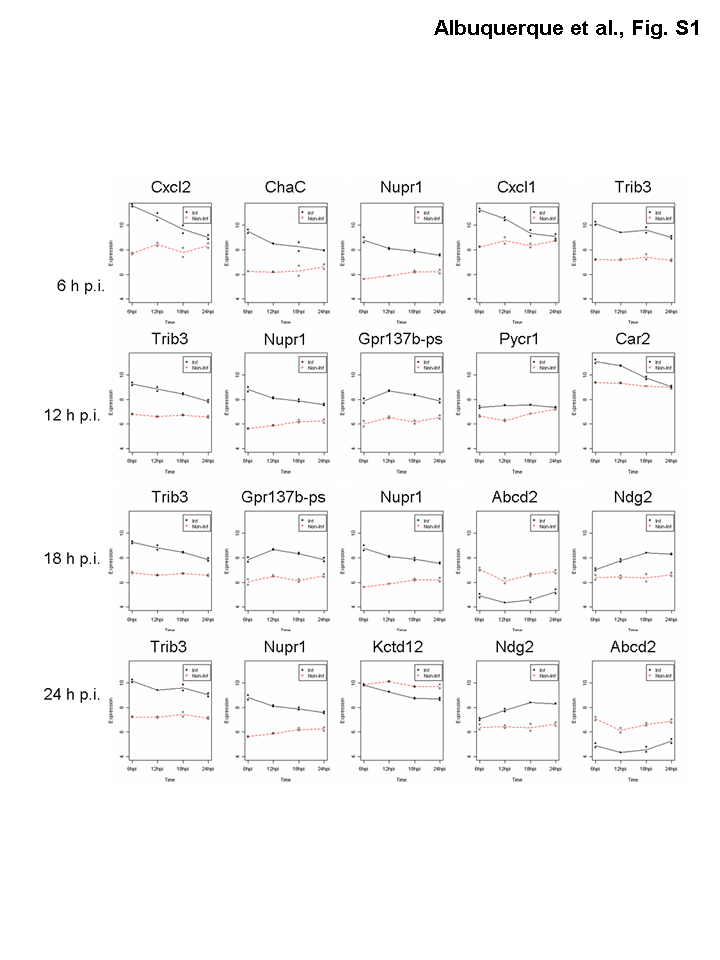

Supplement: Additional file 3 — Expression profiles of the top 5 ranked genes for each time point. The data provided represent the expression profiles of the top 5 ranked genes for each time point. Expression profiles of each gene are shown in black (solid) for infected cells time course and in red (dashed) for non-infected cells time course. Note that the expressions of the biological values are given by the symbols while the average of the 2 is drawn to join the time points. Expression values are given as log2. [file 1471-2164-10-270-S3.tiff]

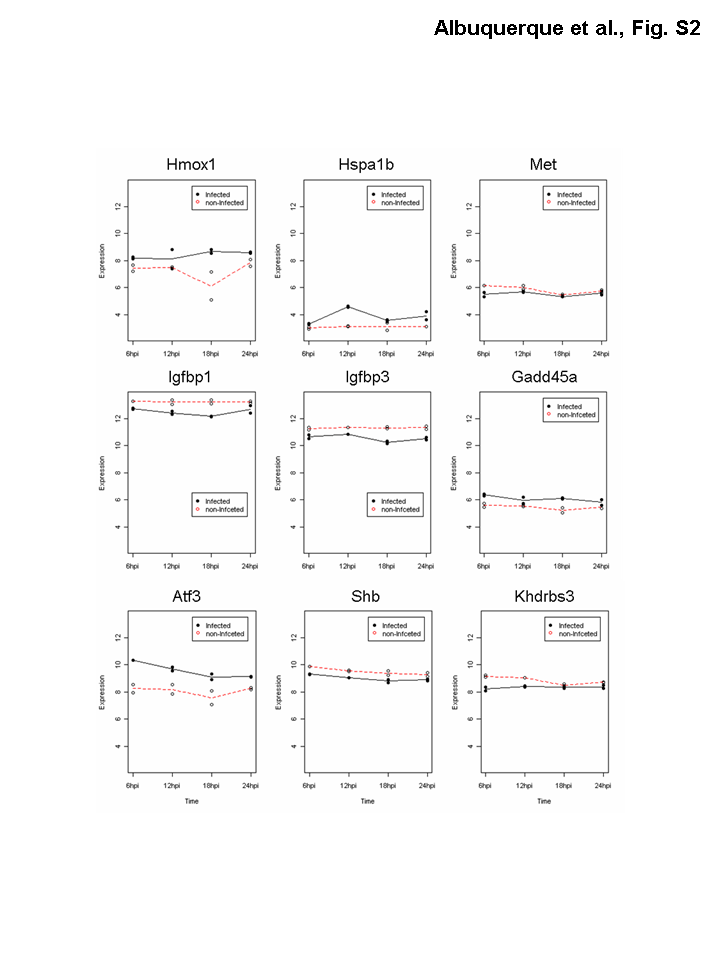

Supplement: Additional file 4 — Expression profiles of further genes mentioned in the body text. The data provided represent the expression profiles of further genes mentioned in the body text. Expression profiles of each gene are shown in black (solid) for infected cells time course and in red (dashed) for non-infected cells time course. Note that the expressions of the biological values are given by the symbols while the average of the 2 is drawn to join the time points. Expression values are given as log2. [file 1471-2164-10-270-S4.tiff]
