# Supplementary material for: Host cell transcriptional profiling during malaria liver stage infection reveals a coordinated and sequential set of biological events
Source: BMC Genomics. 2009 Jun 17;10:270. doi: 10.1186/1471-2164-10-270 (PMC2706893; doi:10.1186/1471-2164-10-270)
Supplement: Additional file 5 — Enrichment analysis of Gene Ontology terms for every transcript differentailly expressed (DE) at any of the time points. The data provided represent the enrichment analysis of Gene Ontology terms for every transcript differentailly expressed (DE) at any of the time points. Gostats package for R was used to compute the hypergeometric test. Each list of DE transcripts at each time point was tested against the total list of transcripts in our analysis after filtering out transcripts acting as control, transcripts showing little variation accross samples, and transcripts without Entrez Gene ID or GO annotation. [file 1471-2164-10-270-S5.doc]

**Additional File 5**: Enrichment analysis of Gene Ontology terms for every transcript differentailly expressed (DE) at any of the time points. Gostats package for R was used to compute the hypergeometric test. Each list of DE transcripts at each time point was tested against the total list of transcripts in our analysis after filtering out transcripts acting as control, transcripts showing little variation accross samples, and transcripts without Entrez Gene ID or GO annotation.

GOBPID Gene Ontology Biological Process Identification number

GOMFID Gene Ontology Molecular Function Identification number

Pvalue p value given by the hypergeometric test (p<0.01)

OddsRatio ratio of odds that a GO term is enriched in the selected category

ExpCount expected number of transcripts found associated with the GO term for enrichment

Count real number of transcripts found associated with the GO term

Size population size of transcripts found associated with the GO term within the analysis

Term Gene Ontology Biological Process description term

Inf Infinite value
