# Supplementary material for: Host cell transcriptional profiling during malaria liver stage infection reveals a coordinated and sequential set of biological events
Source: BMC Genomics. 2009 Jun 17;10:270. doi: 10.1186/1471-2164-10-270 (PMC2706893; doi:10.1186/1471-2164-10-270)
Supplement: Additional file 6 — Similarities found between P. berghei infected cells and P. yoelii infected livers analysis. The data provided represent the similarities found between P. berghei infected cells and P. yoelii infected livers analysis. [file 1471-2164-10-270-S6.doc]

|  | **Additional File 6**: Similarities found between *P. berghei* infected cells and *P.yoelii* infected livers analysis. The grey boxes correspond to the genes highlighted in grey in Additional File 7 and presenting the highest concordance between the 2 datasets. n/a = not applicable. n/d = not determined. | | | | | | | | | | |
| --- | --- | --- | --- | --- | --- | --- | --- | --- | --- | --- | --- |
|  |  |  |  |  |  |  |  |  |  |  |  |
|  | Category of genes in the *P. berghei* analysis | Nb of genes in *P. berghei* analysis | Proportion of those genes found in *P. yoelii* analysis | | Proportion of those genes DE in *P.yoelii* analysis in at least one condition | | Proportion of those genes being DE in *P. yoelii* analysis and showing the same modulation direction as in *P. berghei* analysis | | | | |
|  |  |  | % | Nb | % | Nb | % | Nb | at both time points | at 24 h p.i. | at 40 h p.i. |
|  | differentially expressed at both time points | 1064 | 67.29 | 716 | 42.01 | 447 | n/a | n/a | n/a | n/a | n/a |
|  | over-expressed at all time point | 41 | 78.05 | 32 | 51.22 | 21 | 37.50 | 12 | 6 | 2 | 4 |
|  | under-expressed at all time point | 44 | 72.73 | 32 | 50.00 | 22 | 43.75 | 14 | 3 | 1 | 10 |
|  | over-expressed at 24h p.i. | 106 | 45.28 | 48 | 29.25 | 31 | 33.33 | 16 | 2 | 7 | 7 |
|  | under-expressed at 24 h p.i. | 157 | 49.04 | 77 | 29.94 | 47 | 44.16 | 34 | 5 | 3 | 26 |
|  | Other differentially expressed genes | 716 | 73.74 | 528 | 45.53 | 326 | n/d | n/d | n/d | n/d | n/d |
|  |  |  |  |  |  |  |  |  |  |  |  |
