# Supplementary material for: Host cell transcriptional profiling during malaria liver stage infection reveals a coordinated and sequential set of biological events
Source: BMC Genomics. 2009 Jun 17;10:270. doi: 10.1186/1471-2164-10-270 (PMC2706893; doi:10.1186/1471-2164-10-270)
Supplement: Additional file 7 — List of P. berghei-infected cells modulated genes in P. yoelii-liver infected experiment. The data provided represent the differentially expressed P. berghei-infected cell modulated genes in P. yoelii-liver infected experiment. [file 1471-2164-10-270-S7.doc]

| **Additional File 7**: differentially expressed *P. berghei*-modulated genes in *P. yoelii*-liver infected experiment. For clarity the data set was split into 5 groups corresponding to the 5 groups highlighted in **Additional File 6**. The genes annotated with ** have been found significantly DE in the *P. yoelii*-infected mouse livers experiment using "limma" as described in M&M. The bolded genes are found in the 27 DE genes in *P. berghei* experiment throughout development and showing the same modulation direction. The underlined genes are found in the top-ranking positions in *P. berghei* experiment and showing the same modulation direction. | | | |
| --- | --- | --- | --- |
|  |  |  |  |
| **Genes over-expressed in *P. berghei* at all time points** |  | log2 fold change in *P. yoelii*-infected mouse livers | |
| description | gene symbols | 24hpi-control | 40hpi-control |
| Activating transcription factor 3 | Atf3 | 0.51 | 0.45 |
| Activating transcription factor 5 | Atf5 | -0.55 | 0.61 |
| Aldehyde dehydrogenase 1 family, member L2 | Aldh1l2 | 0.15 | 0.35 |
| Ankyrin repeat domain 12 ** | Ankrd12 | -2.29 | -2.06 |
| B-cell linker | Blnk | -1.76 | -0.29 |
| CCAAT/enhancer binding protein (C/EBP), beta | Cebpb | -0.96 | -0.22 |
| ChaC, cation transport regulator-like 1 (E. coli) | Chac1 | -0.34 | 1.28 |
| Chemokine (C-C motif) ligand 2 | Ccl2 | 1.73 | 1.39 |
| Chemokine (C-X-C motif) ligand 2 | Cxcl2 | 1.72 | 1.29 |
| Cytochrome b5 reductase 1 | Cyb5r1 | 0.00 | 0.21 |
| Dipeptidylpeptidase 7 | Dpp7 | -0.71 | 0.15 |
| DNA-damage inducible transcript 3 | Ddit3 | -0.29 | -0.91 |
| Fibronectin leucine rich transmembrane protein 3 | Flrt3 | 0.03 | 0.33 |
| **G protein-coupled receptor 137B **** | **Gpr137b** | 2.02 | -0.45 |
| Growth differentiation factor 15 | Gdf15 | -2.42 | 1.76 |
| Heme oxygenase (decycling) 1 | Hmox1 | 1.63 | 2.54 |
| Interleukin 1 receptor antagonist | Il1rn | 1.22 | 2.24 |
| **Kruppel-like factor 4 (gut)** | **Klf4** | 1.85 | 0.63 |
| Metallothionein 2 | Mt2 | -0.21 | -0.03 |
| Methylenetetrahydrofolate dehydrogenase (NAD+ dependent), methenyltetrahydrofolate cyclohydrolase | Mthfd2 | 1.51 | 2.12 |
| Myeloid differentiation primary response gene 116 | Myd116 | -0.69 | -0.56 |
| Neuropilin 2 | Nrp2 | 0.61 | 0.26 |
| **Nuclear receptor subfamily 4, group A, member 2** | **Nr4a2** | 0.21 | 0.19 |
| Nur77 downstream gene 2 | Ndg2 | -1.31 | 0.17 |
| Phosphoserine phosphatase | Psph | -0.43 | 0.49 |
| Protein tyrosine phosphatase, non-receptor type 14 | Ptpn14 | -0.12 | 0.79 |
| Receptor accessory protein 6 | Reep6 | -0.19 | -2.62 |
| Sequestosome 1 | Sqstm1 | 0.16 | -0.06 |
| Sestrin 2 | Sesn2 | -0.10 | 0.41 |
| Solute carrier family 20, member 1 | Slc20a1 | 0.02 | -0.37 |
| Solute carrier family 3 (activators of dibasic and neutral amino acid transport), member 2 | Slc3a2 | -0.12 | -0.97 |
| Syndecan binding protein (syntenin) 2 | Sdcbp2 | 0.05 | -0.06 |
|  |  |  |  |
|  |  |  |  |
| **Genes under-expressed in *P. berghei* at all time points** |  | log2 fold change in *P. yoelii*-infected mouse livers | |
| description | gene symbols | 24hpi-control | 40hpi-control |
| Acyl-CoA synthetase long-chain family member 1 | Acsl1 | 0.37 | -0.62 |
| Aldolase 3, C isoform | Aldoc | -0.09 | 0.05 |
| Angiopoietin 1 | Angpt1 | -0.28 | -0.90 |
| Ankyrin repeat domain 37 | Ankrd37 | -0.67 | 0.01 |
| Arylsulfatase G | Arsg | 0.28 | -0.37 |
| **ATP-binding cassette, sub-family D (ALD), member 2** | **Abcd2** | -1.60 | -1.27 |
| Calcitonin receptor-like | Calcrl | 1.20 | -0.61 |
| CAMP responsive element binding protein 3-like 2 | Creb3l2 | 0.33 | -0.64 |
| Chemokine (C-X-C motif) ligand 15 | Cxcl15 | -0.15 | 0.15 |
| Cortactin binding protein 2 | Cttnbp2 | 0.11 | 0.16 |
| Endothelial PAS domain protein 1 | Epas1 | 0.25 | 0.62 |
| Erythrocyte protein band 4.1-like 2 | Epb4.1l2 | 0.09 | 0.12 |
| FK506 binding protein 14 | Fkbp14 | 1.07 | -0.66 |
| Flavin containing monooxygenase 2 | Fmo2 | -0.49 | -0.03 |
| **G protein-coupled receptor 155** | **Gpr155** | -0.65 | -4.12 |
| Glutamyl aminopeptidase | Enpep | -0.46 | -2.49 |
| Hydrocephalus inducing | Hydin | 0.17 | 1.13 |
| Kinesin family member 5C | Kif5c | -0.06 | 0.00 |
| Mitochondrial tumor suppressor 1 | Mtus1 | 0.22 | -1.23 |
| Neuritin 1 | Nrn1 | 1.83 | 0.77 |
| Palmdelphin | Palmd | -0.11 | -2.15 |
| Phospholipase C, beta 1 | Plcb1 | -1.18 | -1.05 |
| Potassium channel, subfamily K, member 2 | Kcnk2 | -0.01 | 0.59 |
| Protein C | Proc | -0.02 | -0.28 |
| Purinergic receptor P2Y, G-protein coupled 12 | P2ry12 | 0.00 | -0.99 |
| RIKEN cDNA 5033414K04 gene | 5033414K04Rik | 1.57 | 1.06 |
| RIKEN cDNA A130090K04 gene | A130090K04Rik | -0.03 | 0.78 |
| SH3-domain GRB2-like (endophilin) interacting protein 1 | Sgip1 | -0.09 | 0.13 |
| Solute carrier family 16 (monocarboxylic acid transporters), member 4 | Slc16a4 | 0.22 | 1.60 |
| Solute carrier family 39 (metal ion transporter), member 8 | Slc39a8 | 0.07 | -4.12 |
| Spinster homolog 2 (Drosophila) | Spns2 | -0.28 | 1.11 |
| Transmembrane protein 71 | Tmem71 | 0.13 | 0.50 |
|  |  |  |  |
|  |  |  |  |
|  |  |  |  |
| **Genes over-expressed in *P. berghei* at 24 h p.i.** |  | log fold change in *P. yoelii*-infected mouse livers | |
| description | gene symbols | 24hpi-control | 40hpi-control |
| 3-hydroxybutyrate dehydrogenase, type 2 | Bdh2 | -2.90 | -2.87 |
| 4-hydroxyphenylpyruvate dioxygenase-like | Hpdl | 0.05 | 0.02 |
| Abhydrolase domain containing 5 | Abhd5 | 0.73 | -0.17 |
| Acyl-CoA thioesterase 2 | Acot2 | -0.41 | 1.25 |
| Adenosine deaminase | Ada | 1.34 | -0.36 |
| Angiopoietin-like 6 | Angptl6 | 0.18 | 2.01 |
| ATPase, H+ transporting, lysosomal V0 subunit B | Atp6v0b | -0.17 | 0.16 |
| Bcl-2-related ovarian killer protein | Bok | 0.16 | 0.93 |
| Cache domain containing 1 | Cachd1 | 0.28 | 0.21 |
| Ceroid lipofuscinosis, neuronal 3, juvenile (Batten, Spielmeyer-Vogt disease) | Cln3 | -0.53 | -0.78 |
| Coiled-coil-helix-coiled-coil-helix domain containing 6 | Chchd6 | -1.29 | -0.62 |
| C-type lectin domain family 4, member d | Clec4d | 1.02 | 2.77 |
| DPH2 homolog (S. cerevisiae) | Dph2 | -0.37 | 0.12 |
| Esterase D/formylglutathione hydrolase | Esd | 0.18 | -0.16 |
| Glutamate-cysteine ligase , modifier subunit | Gclm | -0.07 | 0.10 |
| Glutathione S-transferase omega 2 | Gsto2 | -0.59 | -1.10 |
| Glutathione S-transferase, alpha 3 | Gsta3 | -0.17 | -0.25 |
| Glutathione S-transferase, alpha 4 | Gsta4 | -1.27 | -1.11 |
| Glutathione S-transferase, theta 2 | Gstt2 | -2.45 | 0.12 |
| Glycoprotein (transmembrane) nmb | Gpnmb | -0.12 | 0.09 |
| Hexosaminidase A | Hexa | 1.21 | 0.04 |
| Integrin beta 1 binding protein 1 | Itgb1bp1 | 0.14 | -0.47 |
| Interferon gamma inducible protein 30 | Ifi30 | 0.22 | -0.29 |
| Interferon-induced protein with tetratricopeptide repeats 2 | Ifit2 | 1.76 | -0.74 |
| Kit ligand | Kitl | 0.15 | -0.07 |
| Leucine rich repeat containing 28 | Lrrc28 | 1.10 | -1.40 |
| Leucine rich repeat containing 8D | Lrrc8d | 0.61 | -0.34 |
| Monoamine oxidase A | Maoa | 0.32 | -0.72 |
| Peroxisome proliferative activated receptor, gamma, coactivator 1 alpha | Ppargc1a | -0.26 | -2.03 |
| Phosphomannomutase 1 | Pmm1 | -1.95 | -1.98 |
| Platelet-derived growth factor, C polypeptide | Pdgfc | 1.42 | -0.83 |
| Prostaglandin E synthase 2 | Ptges2 | -1.14 | -0.10 |
| RAB30, member RAS oncogene family | Rab30 | -0.55 | 0.95 |
| Ribosomal protein S25 | Rps25 | -0.41 | -0.01 |
| RIKEN cDNA 1110008P14 gene | 1110008P14Rik | -1.05 | -0.27 |
| RIKEN cDNA 1110020P15 gene | 1110020P15Rik | -2.54 | -1.31 |
| RIKEN cDNA 2410018M08 gene | 2410018M08Rik | -0.17 | 0.42 |
| RIKEN cDNA 2410022L05 gene | 2410022L05Rik | -0.86 | 1.14 |
| RIKEN cDNA 2610528E23 gene | 2610528E23Rik | -0.37 | -0.26 |
| RIKEN cDNA 4930570C03 gene | 4930570C03Rik | -0.22 | -2.85 |
| Scavenger receptor class B, member 2 | Scarb2 | 0.32 | -1.66 |
| SKI-like | Skil | 0.02 | 0.31 |
| Small proline-rich protein 1B | Sprr1b | 0.34 | 1.44 |
| Solute carrier family 35, member E4 | Slc35e4 | 0.23 | 0.60 |
| Solute carrier family 43, member 3 | Slc43a3 | 3.79 | 3.54 |
| Thrombospondin 3 | Thbs3 | -0.05 | 0.08 |
| Transmembrane protein 141 | Tmem141 | -1.41 | -0.13 |
| Transmembrane protein 16A | Tmem16a | 0.11 | 0.21 |
|  |  |  |  |
|  |  |  |  |
| **Genes under-expressed in *P. berghei* at 24 h p.i.** |  | log fold change in *P. yoelii*-infected mouse livers | |
| description | gene symbols | 24hpi-control | 40hpi-control |
| 3-hydroxybutyrate dehydrogenase, type 1 | Bdh1 | 0.03 | 0.14 |
| 3'-phosphoadenosine 5'-phosphosulfate synthase 2 | Papss2 | -0.40 | 0.02 |
| Adaptor protein, phosphotyrosine interaction, PH domain and leucine zipper containing 2 | Appl2 | 1.19 | -1.97 |
| ADP-ribosylation factor guanine nucleotide-exchange factor 1(brefeldin A-inhibited) | Arfgef1 | -1.57 | -1.65 |
| Albumin | Alb | 0.16 | -0.02 |
| Ankyrin 3, epithelial** | Ank3 | -2.08 | -4.55 |
| Aspartate-beta-hydroxylase | Asph | 0.13 | -0.30 |
| ATP-binding cassette, sub-family A (ABC1), member 5 | Abca5 | -0.31 | -0.13 |
| Basic helix-loop-helix domain containing, class B5 | Bhlhb5 | 0.01 | 1.24 |
| Blocked early in transport 1 homolog (S. cerevisiae) | Bet1 | 0.06 | -2.80 |
| Bromodomain and WD repeat domain containing 3 | Brwd3 | -0.17 | 0.18 |
| Carbonic anhydrase 8 | Car8 | -0.91 | -1.41 |
| Carboxypeptidase D | Cpd | -0.05 | 0.47 |
| CD38 antigen | Cd38 | 2.68 | 0.10 |
| Chemokine (C-C motif) ligand 20 | Ccl20 | 0.44 | 0.07 |
| Chemokine (C-X-C motif) ligand 11 | Cxcl11 | 1.12 | 1.02 |
| Coatomer protein complex, subunit beta 2 (beta prime) | Copb2 | 2.08 | -1.71 |
| Collagen, type VIII, alpha 1 | Col8a1 | 0.06 | -0.14 |
| Cyclin T2 | Ccnt2 | -0.07 | 0.44 |
| Cytochrome P450, family 51 | Cyp51 | 0.72 | -0.74 |
| Decorin | Dcn | 2.36 | -1.01 |
| Dystrophin, muscular dystrophy | Dmd | -0.12 | -1.30 |
| Ectonucleotide pyrophosphatase/phosphodiesterase 2 | Enpp2 | -0.18 | -2.00 |
| EP300 interacting inhibitor of differentiation 1 | Eid1 | 0.73 | 0.67 |
| Expressed sequence AI427122 | AI427122 | -0.99 | -3.70 |
| Fibronectin leucine rich transmembrane protein 2 | Flrt2 | 0.54 | 1.21 |
| Fibronectin type III domain containing 3a | Fndc3a | -0.29 | -2.48 |
| FK506 binding protein 7 | Fkbp7 | 0.30 | -3.70 |
| Forkhead box P2 | Foxp2 | -2.40 | -0.46 |
| Fyn-related kinase | Frk | -1.12 | -2.31 |
| Heparan sulfate (glucosamine) 3-O-sulfotransferase 1 | Hs3st1 | -0.17 | -0.09 |
| Hepatocyte growth factor activator | Hgfac | -0.87 | -0.28 |
| Histone cluster 2, H2be ** | Hist2h2be | 0.57 | -2.84 |
| Hydroxyacid oxidase 1, liver | Hao1 | 0.21 | 0.98 |
| Insulin-like growth factor binding protein 3 | Igfbp3 | -0.10 | 0.24 |
| Inter-alpha (globulin) inhibitor H5 | Itih5 | -0.23 | -0.19 |
| Inter-alpha trypsin inhibitor, heavy chain 2 | Itih2 | 0.33 | -0.34 |
| Interleukin 13 receptor, alpha 1 | Il13ra1 | 0.33 | -1.87 |
| Laminin B1 subunit 1 | Lamb1-1 | 0.21 | 0.06 |
| Latrophilin 2 | Lphn2 | -0.69 | -0.54 |
| Meprin 1 beta | Mep1b | -0.09 | 0.21 |
| Mitogen activated protein kinase kinase 6 | Map2k6 | -0.15 | -1.76 |
| Myeloid/lymphoid or mixed-lineage leukemia 3 | Mll3 | 0.01 | 0.27 |
| Myosin, light polypeptide kinase | Mylk | 0.47 | -1.01 |
| Nucleobindin 2 | Nucb2 | 1.21 | -3.74 |
| Phosphatase and tensin homolog | Pten | 0.05 | -1.10 |
| Phosphodiesterase 4D interacting protein (myomegalin) | Pde4dip | 1.04 | -2.49 |
| Platelet derived growth factor receptor, alpha polypeptide | Pdgfra | 2.42 | 0.29 |
| Potassium channel tetramerisation domain containing 12 | Kctd12 | -0.03 | 0.40 |
| Praja 2, RING-H2 motif containing | Pja2 | 0.15 | -1.88 |
| Praja1, RING-H2 motif containing | Pja1 | 0.23 | -0.41 |
| Proline-rich coiled-coil 1 | Prrc1 | -0.40 | -0.52 |
| Protein kinase C, alpha | Prkca | -0.40 | -2.88 |
| Protein kinase, cGMP-dependent, type II | Prkg2 | 1.55 | 3.49 |
| Protein tyrosine phosphatase, receptor type, E | Ptpre | -0.27 | 0.10 |
| Psoriasis susceptibility 1 candidate 2 (human) | Psors1c2 | 0.12 | 0.03 |
| RasGEF domain family, member 1B | Rasgef1b | 1.31 | 0.28 |
| Recombination signal binding protein for immunoglobulin kappa J region | Rbpj | 1.27 | -1.84 |
| RIKEN cDNA 1110020G09 gene | 1110020G09Rik | 0.58 | -0.21 |
| RIKEN cDNA 1810023F06 gene | 1810023F06Rik | 0.58 | 2.53 |
| RIKEN cDNA D030011O10 gene | D030011O10Rik | -0.07 | 0.82 |
| SEC23A (S. cerevisiae) | Sec23a | -0.09 | -1.52 |
| SEC24 related gene family, member D (S. cerevisiae) | Sec24d | 0.48 | -0.98 |
| Serine incorporator 5 | Serinc5 | 1.74 | -0.84 |
| Signal-induced proliferation-associated 1 like 2 | Sipa1l2 | -0.53 | 0.23 |
| Sodium channel, voltage-gated, type I, alpha | Scn1a | 0.16 | 0.37 |
| Spastic paraplegia 3A homolog (human) | Spg3a | 1.55 | -0.93 |
| TBC1 domain family, member 8B | Tbc1d8b | 0.15 | -0.64 |
| Thyroid hormone receptor interactor 12 | Trip12 | -0.26 | 0.88 |
| Transforming growth factor alpha | Tgfa | 0.19 | 0.50 |
| Tripartite motif protein 24 | Trim24 | -0.48 | -2.09 |
| Troponin T2, cardiac | Tnnt2 | -0.29 | 0.10 |
| UDP-N-acetylglucosamine pyrophosphorylase 1 | Uap1 | 0.55 | -2.08 |
| WD repeat domain 26 | Wdr26 | 0.13 | -0.10 |
| Wingless-related MMTV integration site 5A | Wnt5a | 0.49 | 0.19 |
| X-box binding protein 1 | Xbp1 | -0.17 | 0.05 |
| Zinc finger protein 462 | Zfp462 | -0.03 | 0.20 |
|  |  |  |  |
|  |  |  |  |
|  | |  |  |
| **Other differentially expressed genes in *P. berghei* infected hepatoma cells** |  | log fold change in *P. yoelii*-infected mouse livers | |
| description | gene symbols | 24hpi-control | 40hpi-control |
| 2,4-dienoyl CoA reductase 1, mitochondrial | Decr1 | 0.06 | -0.93 |
| 2'-5' oligoadenylate synthetase 1C | Oas1c | -0.11 | -1.34 |
| 3-hydroxy-3-methylglutaryl-Coenzyme A reductase | Hmgcr | 0.38 | -2.12 |
| 3'-phosphoadenosine 5'-phosphosulfate synthase 1 | Papss1 | 0.27 | 1.09 |
| 6-phosphofructo-2-kinase/fructose-2,6-biphosphatase 3 | Pfkfb3 | 0.90 | -0.69 |
| A disintegrin-like and metallopeptidase (reprolysin type) with thrombospondin type 1 motif, 1 | Adamts1 | 2.30 | -0.78 |
| Abl-interactor 1 | Abi1 | 0.22 | -0.16 |
| Acid phosphatase-like 2 | Acpl2 | 0.05 | 0.04 |
| Activated leukocyte cell adhesion molecule | Alcam | 0.13 | -0.98 |
| Activating signal cointegrator 1 complex subunit 2 | Ascc2 | -0.43 | -1.39 |
| Activating signal cointegrator 1 complex subunit 3 | Ascc3 | -0.20 | -0.52 |
| Activating transcription factor 6 | Atf6 | -0.16 | -0.36 |
| Acyl-CoA synthetase long-chain family member 3 | Acsl3 | 1.87 | -5.09 |
| Acyl-CoA synthetase long-chain family member 4 | Acsl4 | -0.82 | -2.50 |
| Acyl-Coenzyme A dehydrogenase family, member 11 | Acad11 | 0.20 | -0.35 |
| ADAMTS-like 5 | Adamtsl5 | 0.08 | -0.20 |
| Adenosine kinase | Adk | 0.43 | -1.16 |
| ADP-ribosylation factor GTPase activating protein 3 | Arfgap3 | -0.44 | -0.05 |
| ADP-ribosylation factor related protein 1 | Arfrp1 | 1.05 | -0.08 |
| ADP-ribosylation factor-like 5B | Arl5b | -0.35 | -0.71 |
| Alcohol dehydrogenase 7 (class IV), mu or sigma polypeptide | Adh7 | 1.25 | 0.73 |
| Aldehyde dehydrogenase family 1, subfamily A7 | Aldh1a7 | 0.19 | -0.55 |
| Amyotrophic lateral sclerosis 2 (juvenile) chromosome region, candidate 2 (human) | Als2cr2 | -0.18 | -0.77 |
| Anaphase promoting complex subunit 1 | Anapc1 | 0.60 | -1.27 |
| Angiopoietin-like 4 | Angptl4 | -1.36 | -0.93 |
| Ankyrin repeat and BTB (POZ) domain containing 2 | Abtb2 | 0.06 | 1.39 |
| Ankyrin repeat and MYND domain containing 2 | Ankmy2 | 0.80 | -0.91 |
| Ankyrin repeat and SOCS box-containing protein 1 | Asb1 | -0.15 | 0.32 |
| Anthrax toxin receptor 1 | Antxr1 | 0.62 | -0.53 |
| Apolipoprotein B editing complex 1 | Apobec1 | 0.89 | -0.12 |
| Apolipoprotein C-IV | Apoc4 | -1.89 | 0.06 |
| Apoptosis inhibitor 5 | Api5 | -0.06 | -0.52 |
| Apoptosis-inducing factor, mitochondrion-associated 1 | Aifm1 | 0.57 | -0.99 |
| Arginine vasopressin receptor 1A | Avpr1a | 1.44 | 0.82 |
| Asparagine-linked glycosylation 6 homolog (yeast, alpha-1,3,-glucosyltransferase) | Alg6 | 0.22 | 0.23 |
| Asparaginyl-tRNA synthetase | Nars | 0.66 | -0.40 |
| Aspartoacylase (aminoacylase) 2 | Aspa | -2.63 | -2.32 |
| ATP/GTP binding protein-like 3 | Agbl3 | -0.10 | 0.06 |
| ATPase, Ca++ transporting, cardiac muscle, slow twitch 2 | Atp2a2 | 1.38 | -0.83 |
| ATPase, Ca++ transporting, plasma membrane 1 | Atp2b1 | -0.25 | -1.96 |
| ATPase, Cu++ transporting, alpha polypeptide | Atp7a | -0.67 | 1.11 |
| ATP-binding cassette, sub-family A (ABC1), member 8b | Abca8b | -0.73 | -0.32 |
| ATP-binding cassette, sub-family C (CFTR/MRP), member 4 | Abcc4 | -0.27 | 0.44 |
| Avian reticuloendotheliosis viral (v-rel) oncogene related B | Relb | 0.33 | 0.53 |
| B-cell leukemia/lymphoma 6 | Bcl6 | 0.42 | -0.17 |
| BCL2/adenovirus E1B interacting protein 3-like | Bnip3l | -0.33 | -2.11 |
| BCL2-antagonist/killer 1 | Bak1 | -0.22 | 1.84 |
| Beta galactoside alpha 2,6 sialyltransferase 1 | St6gal1 | 0.40 | 0.26 |
| Bmi1 polycomb ring finger oncogene | Bmi1 | -1.15 | -2.68 |
| BolA-like 2 (E. coli) | Bola2 | -1.74 | -0.47 |
| Bone marrow stromal cell antigen 1 | Bst1 | 1.14 | 0.50 |
| Brain protein 16 | Brp16 | -0.58 | -0.85 |
| Butyrylcholinesterase | Bche | -0.46 | -0.56 |
| Calcium/calmodulin-dependent protein kinase II, beta | Camk2b | -0.16 | -0.06 |
| Calcium/calmodulin-dependent protein kinase kinase 2, beta | Camkk2 | 0.01 | 0.60 |
| Calpastatin | Cast | 0.18 | -3.31 |
| Calponin 2 | Cnn2 | 0.65 | 0.80 |
| Carbonic anhydrase 2 | Car2 | 0.45 | -1.72 |
| Caspase 4, apoptosis-related cysteine peptidase | Casp4 | 0.33 | 0.39 |
| Caspase 6 | Casp6 | -0.11 | 0.71 |
| Catenin (cadherin associated protein), alpha-like 1 | Ctnnal1 | -0.09 | -1.11 |
| CCAAT/enhancer binding protein (C/EBP), delta | Cebpd | 1.07 | 0.85 |
| CD47 antigen (Rh-related antigen, integrin-associated signal transducer) | Cd47 | 0.47 | -0.75 |
| CDC14 cell division cycle 14 homolog A (S. cerevisiae) | Cdc14a | 0.09 | 1.25 |
| CDC28 protein kinase 1b | Cks1b | -0.09 | -0.14 |
| CDC42 effector protein (Rho GTPase binding) 3 | Cdc42ep3 | 1.81 | 1.78 |
| CDNA sequence AB112350 | AB112350 | 0.11 | 0.23 |
| CDNA sequence BC022623 | BC022623 | 0.26 | 0.49 |
| CDNA sequence BC022687 | BC022687 | -0.16 | 0.31 |
| CDNA sequence BC027231 | BC027231 | 0.26 | -2.01 |
| CDNA sequence BC031853 | BC031853 | -0.17 | -1.13 |
| CDNA sequence BC035537 | BC035537 | -0.14 | -1.83 |
| CDNA sequence BC048355 | BC048355 | -1.09 | -0.20 |
| Cell adhesion molecule 1 | Cadm1 | -0.08 | -0.15 |
| Centaurin, beta 2 | Centb2 | -0.01 | -0.21 |
| Centrosomal protein 57 | Cep57 | -0.67 | -2.46 |
| Centrosome and spindle pole associated protein 1 | Cspp1 | -0.33 | 0.10 |
| Chemokine (C-C motif) ligand 5 | Ccl5 | 3.39 | 1.74 |
| Chemokine (C-X3-C motif) ligand 1 | Cx3cl1 | -0.07 | 0.10 |
| Chemokine (C-X-C motif) ligand 10 | Cxcl10 | 0.50 | 0.79 |
| Chemokine (C-X-C motif) ligand 3 | Cxcl3 | 0.33 | 0.39 |
| Chemokine (C-X-C motif) ligand 5 | Cxcl5 | -0.13 | 0.58 |
| Chloride channel calcium activated 2 | Clca2 | 1.10 | -1.47 |
| Chromodomain protein, Y chromosome-like | Cdyl | 0.21 | 0.15 |
| Cleavage and polyadenylation specific factor 6 | Cpsf6 | 0.19 | -1.99 |
| Coiled-coil domain containing 137 | Ccdc137 | -0.30 | -0.28 |
| Coiled-coil domain containing 86 | Ccdc86 | 0.00 | -0.08 |
| Coiled-coil-helix-coiled-coil-helix domain containing 4 | Chchd4 | -0.08 | 0.51 |
| Collagen, type IV, alpha 3 (Goodpasture antigen) binding protein | Col4a3bp | 0.00 | -3.02 |
| Colony stimulating factor 1 (macrophage) | Csf1 | 0.52 | 0.82 |
| Core 1 synthase, glycoprotein-N-acetylgalactosamine 3-beta-galactosyltransferase, 1 | C1galt1 | -0.11 | -1.75 |
| Coronin, actin binding protein 1C | Coro1c | 0.41 | 1.35 |
| CTD (carboxy-terminal domain, RNA polymerase II, polypeptide A) phosphatase, subunit 1 | Ctdp1 | 0.31 | 0.24 |
| Cyclin B2 | Ccnb2 | 0.63 | -1.00 |
| Cyclin D3 | Ccnd3 | -0.32 | 0.36 |
| Cyclin I | Ccni | 0.51 | -0.24 |
| Cyclin-dependent kinase inhibitor 2B (p15, inhibits CDK4) | Cdkn2b | -0.01 | 0.98 |
| Cysteinyl-tRNA synthetase | Cars | 0.13 | -0.19 |
| Cytochrome b, ascorbate dependent 3 | Cybasc3 | 0.06 | 0.64 |
| Cytochrome P450, family 2, subfamily s, polypeptide 1 | Cyp2s1 | 0.13 | 0.71 |
| Cytoplasmic FMR1 interacting protein 2 | Cyfip2 | 0.14 | 0.14 |
| DEAD (Asp-Glu-Ala-Asp) box polypeptide 10 | Ddx10 | -1.13 | 0.25 |
| DEAD (Asp-Glu-Ala-Asp) box polypeptide 20 | Ddx20 | 0.23 | -0.62 |
| Deafness, autosomal dominant 5 homolog (human) | Dfna5h | 0.18 | 0.59 |
| Dedicator of cytokinesis 4 | Dock4 | 0.11 | 0.06 |
| Deltex 4 homolog (Drosophila) | Dtx4 | 0.60 | -0.13 |
| Deoxynucleotidyltransferase, terminal, interacting protein 2 | Dnttip2 | -0.47 | -3.15 |
| DEP domain containing 7 | Depdc7 | 0.13 | -0.52 |
| Diacylglycerol O-acyltransferase 2 | Dgat2 | 0.05 | -0.06 |
| Dihydrofolate reductase | Dhfr | -1.22 | -0.98 |
| Dihydrouridine synthase 4-like (S. cerevisiae) | Dus4l | -0.05 | -0.02 |
| Discoidin domain receptor family, member 2 | Ddr2 | -0.01 | 0.06 |
| Discs, large homolog 3 (Drosophila) | Dlg3 | -0.04 | 1.77 |
| Distal-less homeobox 1 | Dlx1 | 0.24 | 0.13 |
| DnaJ (Hsp40) homolog, subfamily B, member 9 | Dnajb9 | 0.29 | -3.17 |
| Dopa decarboxylase | Ddc | 0.61 | 0.83 |
| Dopamine receptor 3 | Drd3 | 0.05 | 0.10 |
| Down-regulator of transcription 1 | Dr1 | 0.90 | -1.89 |
| Dual specificity phosphatase 1 | Dusp1 | 1.00 | 1.40 |
| Dual specificity phosphatase 4 | Dusp4 | 0.41 | 0.71 |
| Dymeclin | Dym | 0.37 | -0.35 |
| Dynein light chain Tctex-type 3 | Dynlt3 | -1.21 | -1.48 |
| Dynein, axonemal, heavy chain 6 | Dnahc6 | 0.01 | 0.18 |
| Endoplasmic reticulum (ER) to nucleus signalling 1 | Ern1 | 0.01 | 0.15 |
| Eph receptor A7 | Epha7 | 0.18 | 1.26 |
| ERO1-like beta (S. cerevisiae) | Ero1lb | 0.37 | -0.73 |
| Eukaryotic translation initiation factor 2B, subunit 3 | Eif2b3 | 0.16 | -1.00 |
| Expressed sequence AI597468 | AI597468 | 0.10 | 1.15 |
| Expressed sequence AI597479 | AI597479 | 0.11 | -0.89 |
| Farnesyl diphosphate farnesyl transferase 1 | Fdft1 | 0.29 | -2.56 |
| Fatty acid desaturase 2 | Fads2 | -0.03 | -0.87 |
| FBJ osteosarcoma oncogene B | Fosb | 0.98 | 1.40 |
| F-box and WD-40 domain protein 2 | Fbxw2 | 0.66 | -0.97 |
| F-box protein 33 | Fbxo33 | 0.42 | -1.80 |
| FCH domain only 2 | Fcho2 | -2.39 | -4.34 |
| Feline leukemia virus subgroup C cellular receptor family, member 2 | Flvcr2 | -1.15 | 0.05 |
| Fibrinogen, B beta polypeptide | Fgb | 0.20 | -0.11 |
| Fibroblast growth factor 1 | Fgf1 | 0.78 | -1.12 |
| Fibroblast growth factor binding protein 3 | Fgfbp3 | -0.08 | 0.32 |
| Fibroblast growth factor receptor 4 | Fgfr4 | -0.23 | -0.46 |
| Forkhead box A2 | Foxa2 | -0.24 | -0.11 |
| Forkhead-associated (FHA) phosphopeptide binding domain 1 | Fhad1 | -0.25 | -0.03 |
| Fos-like antigen 1 | Fosl1 | 0.19 | 1.10 |
| Frizzled homolog 4 (Drosophila) | Fzd4 | 0.40 | -0.35 |
| G protein-coupled receptor 124 | Gpr124 | 0.14 | 0.30 |
| G protein-coupled receptor 87 | Gpr87 | 0.09 | 0.17 |
| G two S phase expressed protein 1 | Gtse1 | 0.33 | 0.47 |
| General transcription factor II E, polypeptide 2 (beta subunit) | Gtf2e2 | 0.69 | -0.45 |
| Gephyrin | Gphn | -0.30 | 0.51 |
| GH regulated TBC protein 1 | Grtp1 | -0.20 | -0.04 |
| Glucose-6-phosphate dehydrogenase 2 | G6pd2 | 0.91 | -0.01 |
| Glutamate-rich WD repeat containing 1 | Grwd1 | -0.29 | 0.27 |
| Glutamic pyruvate transaminase (alanine aminotransferase) 2 | Gpt2 | 0.02 | -0.19 |
| Glutamine and serine rich 1 | Qser1 | -1.18 | -0.91 |
| Glutamine fructose-6-phosphate transaminase 2 | Gfpt2 | 0.24 | 0.18 |
| Glutaredoxin | Glrx | -0.33 | -1.22 |
| Glutathione S-transferase, mu 7 | Gstm7 | 0.03 | 0.23 |
| Glycosyltransferase 8 domain containing 1 | Glt8d1 | 0.59 | -0.35 |
| Golgi associated PDZ and coiled-coil motif containing | Gopc | -0.17 | -1.98 |
| Grancalcin | Gca | -0.04 | -3.00 |
| Growth arrest and DNA-damage-inducible 45 alpha | Gadd45a | -0.24 | 0.33 |
| Growth arrest and DNA-damage-inducible 45 gamma | Gadd45g | 0.62 | -0.42 |
| Growth factor receptor bound protein 7 | Grb7 | -1.24 | 1.31 |
| GRP1 (general receptor for phosphoinositides 1)-associated scaffold protein | Grasp | 0.01 | -0.14 |
| GTPase activating RANGAP domain-like 4 | Garnl4 | 0.48 | 1.88 |
| Guanine nucleotide binding protein (G protein), beta polypeptide 1-like | Gnb1l | -1.36 | 0.18 |
| Guanine nucleotide binding protein (G protein), gamma 11 | Gng11 | 0.60 | -0.10 |
| Guanylate nucleotide binding protein 3 | Gbp3 | 0.52 | 2.53 |
| HEAT repeat containing 5B | Heatr5b | -0.28 | -0.02 |
| Heat-responsive protein 12 | Hrsp12 | -1.19 | -2.16 |
| HECT, UBA and WWE domain containing 1 | Huwe1 | -0.10 | -1.27 |
| Hexamthylene bis-acetamide inducible 2 | Hexim2 | -0.99 | 0.64 |
| High density lipoprotein (HDL) binding protein | Hdlbp | 0.75 | 0.34 |
| Histocompatibility 2, T region locus 10 | H2-T10 | 0.67 | 1.22 |
| HIV-1 Rev binding protein-like | Hrbl | -0.04 | 0.08 |
| Hydroxymethylbilane synthase | Hmbs | -0.08 | 1.61 |
| Hydroxyprostaglandin dehydrogenase 15 (NAD) | Hpgd | -0.26 | -0.85 |
| Immediate early response 5 | Ier5 | 0.26 | 0.38 |
| Immunity-related GTPase family, M | Irgm | 0.38 | 0.35 |
| Immunoglobulin mu binding protein 2 | Ighmbp2 | 0.16 | 0.50 |
| Imprinted and ancient | Impact | -1.10 | -3.08 |
| Influenza virus NS1A binding protein | Ivns1abp | 0.66 | -0.89 |
| Inhibitor of DNA binding 2 | Id2 | -0.27 | 0.78 |
| Insulin receptor substrate 1 | Irs1 | -0.03 | 0.73 |
| Insulin-like growth factor binding protein 1 | Igfbp1 | 1.91 | 0.99 |
| Integrin alpha FG-GAP repeat containing 1 | Itfg1 | 0.20 | -6.17 |
| Integrin beta 6 | Itgb6 | 0.15 | 0.34 |
| Intercellular adhesion molecule | Icam1 | 1.97 | -0.25 |
| Interferon gamma receptor 1 | Ifngr1 | 0.76 | -2.40 |
| Interferon inducible GTPase 1 | Iigp1 | -0.26 | 0.34 |
| Interferon-related developmental regulator 1 | Ifrd1 | 0.32 | 0.68 |
| Interleukin 1 receptor, type I | Il1r1 | 1.18 | -1.17 |
| Interleukin 7 | Il7 | -0.74 | -0.57 |
| IQ motif and WD repeats 1 | Iqwd1 | 0.03 | -1.93 |
| Isocitrate dehydrogenase 1 (NADP+), soluble | Idh1 | 0.31 | -1.40 |
| Isopentenyl-diphosphate delta isomerase | Idi1 | 0.06 | -3.55 |
| Kelch repeat and BTB (POZ) domain containing 8 | Kbtbd8 | -0.43 | -0.08 |
| Kelch-like 21 (Drosophila) | Klhl21 | -0.06 | 0.25 |
| Kelch-like 23 (Drosophila) | Klhl23 | 0.86 | 0.26 |
| Kelch-like 28 (Drosophila) | Klhl28 | -0.56 | 1.05 |
| Keratin 5 | Krt5 | 0.09 | 0.74 |
| KH domain containing, RNA binding, signal transduction associated 3 | Khdrbs3 | 0.31 | -3.06 |
| Kinesin family member 17 | Kif17 | 0.14 | 0.41 |
| Kinesin family member 3B | Kif3b | 0.72 | 0.00 |
| Kruppel-like factor 11 | Klf11 | -0.40 | 0.07 |
| Kruppel-like factor 6 | Klf6 | 0.34 | 1.05 |
| Lamin B2 | Lmnb2 | -0.01 | -0.15 |
| Laminin, gamma 2 | Lamc2 | 0.09 | 0.17 |
| Late cornified envelope 1F | Lce1f | 0.06 | 0.82 |
| Latent transforming growth factor beta binding protein 3 | Ltbp3 | 0.12 | 0.48 |
| Leucine rich repeat containing 20 | Lrrc20 | -0.09 | 0.09 |
| Leucine rich repeat containing 4C | Lrrc4c | 0.34 | 0.30 |
| Leucine rich repeat containing G protein coupled receptor 5 | Lgr5 | 0.08 | -0.08 |
| Leucine zipper transcription factor-like 1 | Lztfl1 | -2.08 | -3.22 |
| Ligand dependent nuclear receptor corepressor-like | Lcorl | -0.63 | -0.42 |
| LIM and senescent cell antigen like domains 2 | Lims2 | 0.13 | 0.09 |
| LIM domain only 1 | Lmo1 | 0.16 | 0.65 |
| Lon peptidase 2, peroxisomal | Lonp2 | 0.25 | -0.05 |
| LON peptidase N-terminal domain and ring finger 3 | Lonrf3 | 0.69 | -0.06 |
| Low density lipoprotein receptor | Ldlr | 0.11 | -0.65 |
| Major facilitator superfamily domain containing 8 | Mfsd8 | -0.34 | -1.70 |
| Mal, T-cell differentiation protein 2 | Mal2 | 0.14 | -2.67 |
| Mannan-binding lectin serine peptidase 1 | Masp1 | 0.31 | -2.27 |
| Mannosidase 1, alpha | Man1a | 0.59 | -2.42 |
| Matrin 3 | Matr3 | -0.97 | -1.09 |
| Melanoma inhibitory activity 3 | Mia3 | -1.21 | -1.69 |
| Membrane bound O-acyltransferase domain containing 2 | Mboat2 | 0.04 | 0.73 |
| Membrane bound O-acyltransferase domain containing 5 | Mboat5 | -0.60 | -0.23 |
| Membrane protein, palmitoylated 7 (MAGUK p55 subfamily member 7) | Mpp7 | -0.19 | 0.43 |
| Mesoderm induction early response 1 homolog (Xenopus laevis | Mier1 | -0.29 | -0.33 |
| Met proto-oncogene | Met | -0.34 | -2.07 |
| Metallothionein 1 | Mt1 | -0.11 | 0.05 |
| Metastasis associated 3 | Mta3 | -0.36 | 0.44 |
| Methionine sulfoxide reductase B2 | Msrb2 | 0.22 | -0.21 |
| Methionine sulfoxide reductase B3 | Msrb3 | -0.24 | 0.92 |
| Methylcrotonoyl-Coenzyme A carboxylase 1 (alpha) | Mccc1 | 0.14 | -0.78 |
| Methyltransferase like 2 | Mettl2 | 0.05 | 0.38 |
| Microsomal triglyceride transfer protein | Mttp | 0.05 | 0.02 |
| Mitogen activated protein kinase kinase kinase 12 | Map3k12 | -0.12 | -0.12 |
| M-phase phosphoprotein 9 | Mphosph9 | -0.16 | 0.47 |
| Myeloid ecotropic viral integration site 1 | Meis1 | -0.39 | -0.34 |
| Myosin Vb | Myo5b | -0.46 | 0.33 |
| N-acetyltransferase 12 | Nat12 | 0.72 | 0.20 |
| NAD(P)H dehydrogenase, quinone 1 | Nqo1 | 0.25 | 0.01 |
| NADH dehydrogenase (ubiquinone) 1 alpha subcomplex 10 | Ndufa10 | -0.49 | -0.09 |
| Neogenin | Neo1 | 0.10 | 0.35 |
| N-ethylmaleimide sensitive fusion protein attachment protein alpha | Napa | -1.26 | -1.62 |
| Neuron navigator 3 | Nav3 | 0.21 | 0.16 |
| Ngg1 interacting factor 3-like 1 (S. pombe) | Nif3l1 | 0.14 | 0.33 |
| N-glycanase 1 | Ngly1 | -0.57 | -2.48 |
| NIPA-like domain containing 1 | Npal1 | 0.06 | -1.62 |
| NMD3 homolog (S. cerevisiae) | Nmd3 | -0.60 | -2.62 |
| Notch gene homolog 1 (Drosophila) | Notch1 | 0.18 | 1.25 |
| NUAK family, SNF1-like kinase, 1 | Nuak1 | 0.20 | 2.23 |
| Nuclear factor of kappa light chain gene enhancer in B-cells inhibitor, beta | Nfkbib | -0.43 | -0.01 |
| Nuclear factor of kappa light polypeptide gene enhancer in B-cells inhibitor, zeta | Nfkbiz | 0.25 | 0.58 |
| Nuclear factor, interleukin 3, regulated | Nfil3 | 1.13 | 0.63 |
| Nuclear receptor subfamily 1, group D, member 1 | Nr1d1 | -0.87 | -0.20 |
| Nuclear receptor subfamily 3, group C, member 1 | Nr3c1 | -0.83 | -0.98 |
| Nuclear transcription factor-Y beta | Nfyb | 0.58 | -0.23 |
| Nucleotide binding protein 2 | Nubp2 | -0.55 | -0.08 |
| Odd-skipped related 1 (Drosophila) | Osr1 | 0.08 | 1.09 |
| Olfactomedin-like 2B | Olfml2b | -0.15 | -0.24 |
| One cut domain, family member 2 | Onecut2 | -2.07 | 1.38 |
| Ornithine decarboxylase, structural 1 | Odc1 | 0.45 | 0.03 |
| OTU domain, ubiquitin aldehyde binding 1 | Otub1 | -0.35 | -0.90 |
| Oxidative stress induced growth inhibitor 1 | Osgin1 | -0.03 | 0.99 |
| P300/CBP-associated factor | Pcaf | 0.03 | -1.80 |
| Paired box gene 6 | Pax6 | -0.13 | 0.81 |
| PC4 and SFRS1 interacting protein 1 | Psip1 | -1.06 | -1.42 |
| Peptidylglycine alpha-amidating monooxygenase COOH-terminal interactor | Pamci | -0.03 | 0.64 |
| Pescadillo homolog 1, containing BRCT domain (zebrafish) | Pes1 | 0.24 | -2.07 |
| Phosphatidic acid phosphatase type 2 domain containing 1 | Ppapdc1 | 0.35 | -2.12 |
| Phosphatidic acid phosphatase type 2B | Ppap2b | -0.01 | 1.23 |
| Phosphatidylcholine transfer protein | Pctp | 0.93 | 1.72 |
| Phosphatidylinositol glycan anchor biosynthesis, class F | Pigf | -0.12 | 0.08 |
| Phosphofructokinase, muscle | Pfkm | -0.19 | 0.11 |
| Phosphoglucomutase 2 | Pgm2 | -0.24 | 0.17 |
| Phosphohistidine phosphatase 1 | Phpt1 | -1.09 | 0.56 |
| Phospholipase B1 | Plb1 | -0.08 | 0.24 |
| Phosphomevalonate kinase | Pmvk | -0.13 | 0.26 |
| Phosphorylase kinase beta | Phkb | -0.13 | -2.51 |
| Pitrilysin metallepetidase 1 | Pitrm1 | 0.12 | -3.34 |
| Plasmacytoma variant translocation 1 | Pvt1 | 0.05 | -0.09 |
| Plastin 3 (T-isoform) | Pls3 | 0.51 | -1.59 |
| Platelet-derived growth factor receptor-like | Pdgfrl | -0.05 | 0.22 |
| Pleckstrin homology domain containing, family C (with FERM domain) member 1 | Plekhc1 | 0.30 | -0.34 |
| Plexin B1 | Plxnb1 | 0.14 | -0.29 |
| Podocalyxin-like | Podxl | 0.80 | -0.09 |
| Poly (ADP-ribose) polymerase family, member 3 | Parp3 | -0.42 | 0.60 |
| Polyamine oxidase (exo-N4-amino) | Paox | -0.33 | 0.69 |
| Polycystic kidney and hepatic disease 1 | Pkhd1 | -0.24 | -0.55 |
| Polymerase (RNA) I polypeptide E | Polr1e | -0.23 | -2.68 |
| Polynucleotide kinase 3'- phosphatase | Pnkp | -1.00 | 0.01 |
| Potassium channel tetramerisation domain containing 15 | Kctd15 | -0.83 | -0.75 |
| Potassium channel tetramerisation domain containing 20 | Kctd20 | 0.30 | 0.17 |
| Potassium channel, subfamily K, member 5 | Kcnk5 | -0.58 | 0.11 |
| Pre B-cell leukemia transcription factor 3 | Pbx3 | -0.16 | 0.13 |
| Predicted gene, EG381438 | EG381438 | 0.19 | 0.64 |
| Predicted gene, ENSMUSG00000052976 | ENSMUSG00000052976 | -1.27 | -1.80 |
| Pregnancy specific glycoprotein 17 | Psg17 | -0.03 | 0.08 |
| Premature ovarian failure 1B | Pof1b | 0.12 | 0.11 |
| Proliferation-associated 2G4 | Pa2g4 | 0.80 | 0.60 |
| Proline rich 16 | Prr16 | -0.50 | -4.12 |
| Prolyl endopeptidase-like | Prepl | 0.08 | -1.87 |
| Pro-platelet basic protein | Ppbp | 0.13 | 2.21 |
| Protein C receptor, endothelial | Procr | 0.45 | 0.52 |
| Protein kinase, AMP-activated, alpha 2 catalytic subunit | Prkaa2 | -0.20 | -0.07 |
| Protein kinase, AMP-activated, gamma 2 non-catalytic subunit | Prkag2 | -0.12 | -3.57 |
| Protein kinase, cAMP dependent regulatory, type I beta | Prkar1b | 0.15 | 0.46 |
| Protein phosphatase 1 (formerly 2C)-like | Ppm1l | -0.04 | -0.03 |
| Protein phosphatase 1, regulatory (inhibitor) subunit 9A | Ppp1r9a | 0.03 | 0.20 |
| Protein phosphatase 1D magnesium-dependent, delta isoform | Ppm1d | 0.37 | -1.28 |
| Protocadherin 9 | Pcdh9 | 0.20 | 0.17 |
| Purine rich element binding protein A | Pura | -0.13 | 2.81 |
| Purine rich element binding protein B | Purb | -0.62 | -1.07 |
| Purinergic receptor P2Y, G-protein coupled, 14 | P2ry14 | 0.75 | -0.50 |
| PX domain containing serine/threonine kinase | Pxk | -0.27 | -1.64 |
| Pyrroline-5-carboxylate reductase 1 | Pycr1 | 0.17 | 0.29 |
| RAB32, member RAS oncogene family | Rab32 | 0.94 | -0.28 |
| RAB4A, member RAS oncogene family | Rab4a | 0.43 | 0.11 |
| RAD9 homolog (S. pombe) | Rad9 | 0.14 | -0.08 |
| Radical S-adenosyl methionine domain containing 2 | Rsad2 | 1.20 | 2.02 |
| RAR-related orphan receptor alpha | Rora | -0.51 | -0.09 |
| Ras homolog gene family, member B | Rhob | 0.46 | 1.39 |
| RAS p21 protein activator 3 | Rasa3 | 0.09 | 0.12 |
| RAS, guanyl releasing protein 3 | Rasgrp3 | 2.28 | -2.50 |
| Receptor accessory protein 1 | Reep1 | 0.69 | -1.44 |
| RecQ protein-like 4 | Recql4 | -0.16 | -0.12 |
| Regulator of calcineurin 2 | Rcan2 | 0.36 | -5.07 |
| Reticuloendotheliosis oncogene | Rel | 0.33 | -0.99 |
| Retinitis pigmentosa 2 homolog (human) | Rp2h | -1.69 | -1.59 |
| Retinol dehydrogenase 11 | Rdh11 | 0.40 | 0.38 |
| Retinol saturase (all trans retinol 13,14 reductase) | Retsat | -1.25 | -0.27 |
| Rho family GTPase 1 | Rnd1 | 1.51 | 2.96 |
| Rho GTPase activating protein 18 | Arhgap18 | -0.75 | -1.58 |
| Rho GTPase activating protein 5 | Arhgap5 | -2.06 | -2.88 |
| Rho guanine nucleotide exchange factor (GEF) 10 | Arhgef10 | 0.51 | -1.46 |
| Rho/rac guanine nucleotide exchange factor (GEF) 2 | Arhgef2 | 0.12 | -0.23 |
| Rho-guanine nucleotide exchange factor | Rgnef | -0.18 | 0.66 |
| Ribonuclease L (2', 5'-oligoisoadenylate synthetase-dependent) | Rnasel | 0.30 | 0.35 |
| Ribonuclease P 14 subunit (human) | Rpp14 | 0.01 | 0.21 |
| Ribosomal protein L7-like 1 | Rpl7l1 | 0.41 | 0.69 |
| Ribosomal protein L9 | Rpl9 | 0.07 | 0.03 |
| Ribosome binding protein 1 | Rrbp1 | -0.35 | -1.01 |
| RIKEN cDNA 0610007L01 gene | 0610007L01Rik | 1.30 | 0.23 |
| RIKEN cDNA 0610007P14 gene | 0610007P14Rik | 0.09 | 0.36 |
| RIKEN cDNA 0610011L14 gene | 0610011L14Rik | -0.37 | 0.11 |
| RIKEN cDNA 1110054O05 gene | 1110054O05Rik | 0.33 | -1.46 |
| RIKEN cDNA 1200009F10 gene | 1200009F10Rik | 1.15 | -0.61 |
| RIKEN cDNA 1700008I05 gene | 1700008I05Rik | -0.27 | 0.04 |
| RIKEN cDNA 1700011H14 gene | 1700011H14Rik | -0.09 | 0.72 |
| RIKEN cDNA 1810013L24 gene | 1810013L24Rik | 0.11 | -1.16 |
| RIKEN cDNA 1810074P20 gene | 1810074P20Rik | 0.10 | -4.96 |
| RIKEN cDNA 2310007B03 gene | 2310007B03Rik | 0.12 | 0.90 |
| RIKEN cDNA 2310007H09 gene | 2310007H09Rik | -0.77 | -0.30 |
| RIKEN cDNA 2310008M10 gene | 2310008M10Rik | -0.17 | 0.41 |
| RIKEN cDNA 2310056P07 gene | 2310056P07Rik | -1.31 | -0.41 |
| RIKEN cDNA 2410025L10 gene | 2410025L10Rik | -0.07 | -0.08 |
| RIKEN cDNA 2410131K14 gene | 2410131K14Rik | -0.48 | -2.79 |
| RIKEN cDNA 2410187C16 gene | 2410187C16Rik | 0.25 | 0.79 |
| RIKEN cDNA 2610207I05 gene | 2610207I05Rik | -0.01 | -0.19 |
| RIKEN cDNA 2700007P21 gene | 2700007P21Rik | 0.30 | -1.86 |
| RIKEN cDNA 2700078E11 gene | 2700078E11Rik | 1.08 | -0.16 |
| RIKEN cDNA 2900024C23 gene | 2900024C23Rik | -0.30 | -4.22 |
| RIKEN cDNA 2900024O10 gene | 2900024O10Rik | -0.60 | 0.37 |
| RIKEN cDNA 3110004L20 gene | 3110004L20Rik | -0.07 | -0.89 |
| RIKEN cDNA 3110032G18 gene | 3110032G18Rik | -0.04 | 0.19 |
| RIKEN cDNA 3110043O21 gene | 3110043O21Rik | 0.16 | 0.23 |
| RIKEN cDNA 3830406C13 gene | 3830406C13Rik | 0.17 | -2.02 |
| RIKEN cDNA 4631426J05 gene | 4631426J05Rik | 2.13 | 1.96 |
| RIKEN cDNA 4732479N06 gene | 4732479N06Rik | 0.04 | -1.11 |
| RIKEN cDNA 5730469M10 gene | 5730469M10Rik | -0.70 | 0.73 |
| RIKEN cDNA 6430527G18 gene | 6430527G18Rik | 0.10 | 0.28 |
| RIKEN cDNA 6720458F09 gene | 6720458F09Rik | 0.08 | 1.38 |
| RIKEN cDNA 9130213B05 gene | 9130213B05Rik | 0.39 | 0.83 |
| RIKEN cDNA 9530058B02 gene | 9530058B02Rik | -1.46 | 1.54 |
| RIKEN cDNA A930035D04 gene | A930035D04Rik | -0.24 | 0.15 |
| RIKEN cDNA A930037G23 gene | A930037G23Rik | -0.47 | -0.28 |
| RIKEN cDNA B930006L02 gene | B930006L02Rik | 0.39 | -0.29 |
| RIKEN cDNA C030007I09 gene | C030007I09Rik | 1.00 | -0.15 |
| RIKEN cDNA C230013L11 gene | C230013L11Rik | -0.22 | -0.12 |
| RIKEN cDNA C230093N12 gene | C230093N12Rik | -0.51 | -0.13 |
| RIKEN cDNA C330011K17 gene | C330011K17Rik | -0.67 | -0.34 |
| RIKEN cDNA D230012E17 gene | D230012E17Rik | 0.02 | 0.11 |
| RIKEN cDNA D530033C11 gene | D530033C11Rik | 0.37 | 1.13 |
| RIKEN cDNA D630023B12 gene | D630023B12Rik | -0.37 | 0.21 |
| RIKEN cDNA D630039A03 gene | D630039A03Rik | 0.51 | -0.68 |
| RIKEN cDNA E030049G20 gene | E030049G20Rik | 0.11 | 0.10 |
| RIKEN cDNA E130014J05 gene | E130014J05Rik | -0.04 | 0.42 |
| RNA binding motif protein 43 | Rbm43 | 0.77 | -0.03 |
| RNA binding motif protein 5 | Rbm5 | 0.26 | -0.96 |
| RNA pseudouridylate synthase domain containing 2 | Rpusd2 | -0.96 | -0.99 |
| Rosbin, round spermatid basic protein 1 | Rsbn1 | -1.40 | -0.41 |
| Roundabout homolog 1 (Drosophila) | Robo1 | 1.52 | -0.71 |
| S100 calcium binding protein A4 | S100a4 | 0.33 | 2.80 |
| Schlafen 2 | Slfn2 | 0.75 | 2.49 |
| Secernin 3 | Scrn3 | -0.29 | -1.43 |
| Selenium binding protein 2 | Selenbp2 | -0.86 | 0.13 |
| Sema domain, immunoglobulin domain (Ig), short basic domain, secreted, (semaphorin) 3F | Sema3f | 0.31 | 0.21 |
| Serine (or cysteine) peptidase inhibitor, clade A (alpha-1 antiproteinase, antitrypsin), member 10 | Serpina10 | 0.76 | 0.46 |
| Serine (or cysteine) peptidase inhibitor, clade B, member 9b | Serpinb9b | 0.16 | 0.44 |
| Serine (or cysteine) peptidase inhibitor, clade B, member 9g | Serpinb9g | -0.05 | 1.10 |
| Serine/threonine kinase 10 | Stk10 | 1.27 | 0.20 |
| Serine/threonine kinase 17b (apoptosis-inducing) | Stk17b | 0.17 | -1.94 |
| Serine/threonine/tyrosine kinase 1 | Styk1 | 0.02 | 0.42 |
| Sestrin 1 | Sesn1 | 1.26 | -0.83 |
| SET and MYND domain containing 3 | Smyd3 | -0.10 | -0.30 |
| SFT2 domain containing 2 | Sft2d2 | 1.02 | -3.73 |
| Signal transducer and activator of transcription 5A | Stat5a | 1.13 | 0.89 |
| Small proline-rich protein 2H | Sprr2h | 0.15 | -0.22 |
| Smoothelin-like 2 | Smtnl2 | -0.03 | 1.55 |
| SNAP-associated protein | Snapap | 0.42 | -1.67 |
| SNF1-like kinase | Snf1lk | 0.88 | 1.02 |
| Solute carrier family 1 (glutamate/neutral amino acid transporter), member 4 | Slc1a4 | 1.52 | -1.00 |
| Solute carrier family 12, member 5 | Slc12a5 | 0.00 | -0.09 |
| Solute carrier family 19 (thiamine transporter), member 2 | Slc19a2 | 0.20 | -0.74 |
| Solute carrier family 22 (organic anion/cation transporter), member 15 | Slc22a15 | -0.41 | -0.81 |
| Solute carrier family 25 (mitochondrial carrier, Aralar), member 12 | Slc25a12 | 0.29 | -0.26 |
| Solute carrier family 25, member 33 | Slc25a33 | -0.05 | 1.35 |
| Solute carrier family 26, member 3 | Slc26a3 | -0.10 | 0.12 |
| Solute carrier family 30 (zinc transporter), member 1 | Slc30a1 | 0.70 | -1.39 |
| Solute carrier family 33 (acetyl-CoA transporter), member 1 | Slc33a1 | 0.06 | -1.38 |
| Solute carrier family 35 (UDP-galactose transporter), member A2 | Slc35a2 | -0.09 | -0.08 |
| Solute carrier family 35, member C2 | Slc35c2 | -0.32 | 0.55 |
| Solute carrier family 38, member 2 | Slc38a2 | -0.80 | -3.07 |
| Solute carrier family 38, member 4 | Slc38a4 | 0.05 | -0.54 |
| Solute carrier family 4 (anion exchanger), member 4 | Slc4a4 | 0.24 | 0.61 |
| Solute carrier family 41, member 2 | Slc41a2 | 0.02 | -0.61 |
| Solute carrier family 45, member 3 | Slc45a3 | 0.65 | -0.32 |
| Solute carrier family 47, member 1 | Slc47a1 | 0.11 | -0.76 |
| Solute carrier family 6 (neurotransmitter transporter, glycine), member 9 | Slc6a9 | -0.93 | -0.20 |
| Solute carrier family 7 (cationic amino acid transporter, y+ system), member 1 | Slc7a1 | 0.12 | 0.32 |
| Solute carrier family 7 (cationic amino acid transporter, y+ system), member 5 | Slc7a5 | 0.33 | 1.26 |
| Solute carrier family 7, member 6 opposite strand | Slc7a6os | 0.26 | 0.10 |
| Sorbin and SH3 domain containing 2 | Sorbs2 | 0.03 | -1.25 |
| Sorbitol dehydrogenase | Sord | 0.12 | -0.01 |
| Sorting nexin 25 | Snx25 | -0.15 | -3.56 |
| Sorting nexing 24 | Snx24 | -0.67 | -2.30 |
| Spermidine/spermine N1-acetyl transferase 1 | Sat1 | -0.23 | 0.02 |
| Sphingomyelin phosphodiesterase 1, acid lysosomal | Smpd1 | -0.34 | -0.78 |
| Spire homolog 1 (Drosophila) | Spire1 | 0.11 | -0.04 |
| SPT2, Suppressor of Ty, domain containing 1 (S. cerevisiae) | Spty2d1 | -0.16 | -0.31 |
| Squalene epoxidase | Sqle | 1.42 | -1.82 |
| Src homology 2 domain-containing transforming protein B | Shb | -0.34 | -1.56 |
| ST6 (alpha-N-acetyl-neuraminyl-2,3-beta-galactosyl-1,3)-N-acetylgalactosaminide alpha-2,6-sialyltransferase 4 | St6galnac4 | -0.23 | 0.89 |
| Stam binding protein | Stambp | 0.16 | -1.08 |
| Stannin | Snn | 0.10 | 2.76 |
| STE20-like kinase (yeast) | Slk | -2.14 | -2.55 |
| STEAP family member 4 | Steap4 | 1.83 | 0.91 |
| Sterile alpha motif domain containing 9-like | Samd9l | -1.35 | -0.98 |
| Sterol carrier protein 2, liver | Scp2 | 0.13 | 0.01 |
| Sterol regulatory element binding factor 1 | Srebf1 | -1.98 | -1.02 |
| Stromal antigen 2 | Stag2 | -2.34 | -2.89 |
| Sulfiredoxin 1 homolog (S. cerevisiae) | Srxn1 | -0.24 | -0.61 |
| SUMO1/sentrin specific peptidase 7 | Senp7 | -1.17 | -1.11 |
| Swi/SNF related matrix associated, actin dependent regulator of chromatin, subfamily a-like 1 | Smarcal1 | -0.73 | -0.21 |
| Syntaxin 11 | Stx11 | 0.19 | 0.79 |
| Tankyrase, TRF1-interacting ankyrin-related ADP-ribose polymerase 2 | Tnks2 | -0.23 | 0.48 |
| TAP binding protein-like | Tapbpl | 0.42 | 0.43 |
| TATA box binding protein (Tbp)-associated factor, RNA polymerase I, A | Taf1a | 0.15 | 0.45 |
| TBC1 domain family, member 5 | Tbc1d5 | -0.88 | 0.64 |
| TBC1 domain family, member 8 | Tbc1d8 | -0.17 | -0.30 |
| T-box 3 | Tbx3 | 1.07 | 0.02 |
| Tetratricopeptide repeat domain 3 | Ttc3 | -0.31 | -1.11 |
| Tetratricopeptide repeat domain 8 | Ttc8 | -0.65 | -2.00 |
| Thiamine pyrophosphokinase | Tpk1 | 1.63 | -2.88 |
| Thioredoxin reductase 1 | Txnrd1 | 0.93 | 0.16 |
| Thymidine kinase 1 | Tk1 | -1.77 | 0.59 |
| Tnf receptor-associated factor 1 | Traf1 | 0.11 | 0.16 |
| Tnf receptor-associated factor 5 | Traf5 | -0.01 | 0.18 |
| Transcriptional adaptor 1 (HFI1 homolog, yeast) like | Tada1l | 0.23 | 0.80 |
| Transducin (beta)-like 2 | Tbl2 | 0.05 | -0.11 |
| Transducin (beta)-like 3 | Tbl3 | -1.16 | 0.37 |
| Transformation related protein 53 inducible nuclear protein 1 | Trp53inp1 | -0.14 | 0.09 |
| Transformed mouse 3T3 cell double minute 2 | Mdm2 | 0.19 | -1.71 |
| Trans-golgi network protein | Tgoln1 | 0.49 | 0.22 |
| Transient receptor potential cation channel, subfamily C, member 1 | Trpc1 | -0.26 | 0.32 |
| Transient receptor potential cation channel, subfamily M, member 6 | Trpm6 | -0.19 | -0.43 |
| Translocase of inner mitochondrial membrane 10 homolog (yeast) | Timm10 | -0.62 | 0.53 |
| Transmembrane 9 superfamily member 2 | Tm9sf2 | 0.33 | -0.51 |
| Transmembrane and tetratricopeptide repeat containing 3 | Tmtc3 | 0.02 | 0.01 |
| Transmembrane protein 150 | Tmem150 | 0.13 | 0.19 |
| Transmembrane protein 176A | Tmem176a | 0.24 | -0.21 |
| Transmembrane protein 41B | Tmem41b | 0.68 | -1.34 |
| Trimethyllysine hydroxylase, epsilon | Tmlhe | -0.55 | -1.12 |
| Tripartite motif protein 2 | Trim2 | -0.20 | -1.50 |
| Trypsin domain containing 1 | Tysnd1 | -0.36 | -0.01 |
| Tubulin, gamma complex associated protein 2 | Tubgcp2 | -0.32 | 0.24 |
| Tumor necrosis factor receptor superfamily, member 19 | Tnfrsf19 | -0.16 | 0.07 |
| Tumor necrosis factor receptor superfamily, member 22 | Tnfrsf22 | 0.83 | 0.20 |
| Tumor necrosis factor, alpha-induced protein 3 | Tnfaip3 | 0.28 | -0.55 |
| Ubiquitin specific peptidase 18 | Usp18 | 1.01 | 0.47 |
| Ubiquitin specific peptidase 40 | Usp40 | -0.36 | 1.12 |
| Ubiquitin-activating enzyme E1-like | Ube1l | -0.82 | 0.36 |
| UDP glucuronosyltransferase 2 family, polypeptide B34 | Ugt2b34 | -0.64 | -3.30 |
| UDP glucuronosyltransferase 2 family, polypeptide B35 | Ugt2b35 | -0.33 | -1.14 |
| UDP-glucose pyrophosphorylase 2 | Ugp2 | -0.40 | -1.98 |
| UDP-N-acteylglucosamine pyrophosphorylase 1-like 1 | Uap1l1 | 0.13 | 0.50 |
| Unc-51 like kinase 2 (C. elegans) | Ulk2 | -0.74 | -3.35 |
| Vac14 homolog (S. cerevisiae) | Vac14 | -0.82 | -1.09 |
| Vacuolar protein sorting 25 (yeast) | Vps25 | -1.65 | -0.41 |
| Vacuolar protein sorting 37B (yeast) | Vps37b | -0.62 | 0.09 |
| Vanin 1 | Vnn1 | 1.01 | -0.06 |
| Vascular cell adhesion molecule 1 | Vcam1 | 3.14 | -0.20 |
| Villin 1 | Vil1 | 0.04 | 0.35 |
| V-maf musculoaponeurotic fibrosarcoma oncogene family, protein K (avian) | Mafk | 0.40 | 0.70 |
| WD repeat domain 62 | Wdr62 | -0.12 | 0.24 |
| WD repeat domain 74 | Wdr74 | -0.91 | 0.14 |
| WD repeat domain 79 | Wdr79 | -1.43 | 1.24 |
| X-ray repair complementing defective repair in Chinese hamster cells 4 | Xrcc4 | 0.03 | 0.11 |
| Zinc binding alcohol dehydrogenase, domain containing 1 | Zadh1 | 0.26 | 0.48 |
| Zinc finger CCCH type containing 8 | Zc3h8 | -1.49 | -2.25 |
| Zinc finger protein 236 | Zfp236 | 0.20 | -2.53 |
| Zinc finger protein 260 | Zfp260 | -1.21 | -0.09 |
| Zinc finger protein 296 | Zfp296 | -0.18 | 0.11 |
| Zinc finger protein 296 | Zfp296 | -0.18 | 0.11 |
| Zinc finger protein 444 | Zfp444 | -0.61 | 0.23 |
| Zinc finger protein 467 | Zfp467 | 0.01 | 0.18 |
| Zinc finger protein 593 | Zfp593 | -1.82 | 0.57 |
| Zinc finger protein 655 | Zfp655 | -1.86 | -2.05 |
| Zinc finger protein 787 | Zfp787 | -0.13 | -0.29 |
| Zinc finger, AN1-type domain 1 | Zfand1 | 0.71 | -1.96 |
| Zinc finger, CCHC domain containing 2 | Zcchc2 | -0.02 | 0.34 |
| Zinc finger, FYVE domain containing 20 | Zfyve20 | 0.09 | -0.51 |
| Zinc finger, MIZ-type containing 1 | Zmiz1 | 0.17 | 0.44 |
| Zinc finger, SWIM domain containing 1 | Zswim1 | 0.13 | -0.14 |
