# Supplementary material for: Host cell transcriptional profiling during malaria liver stage infection reveals a coordinated and sequential set of biological events
Source: BMC Genomics. 2009 Jun 17;10:270. doi: 10.1186/1471-2164-10-270 (PMC2706893; doi:10.1186/1471-2164-10-270)
Supplement: Additional file 8 — Primer Sequences. The data provided represent all the primer sequences used in the qRT-PCR throughout the work. [file 1471-2164-10-270-S8.doc]

**Additional File 8**. Primer Sequences used in the qRT-PCR.

Gene name Locus ID forward primer 5’-3’ reverse primer 5’-3’

*nupr1* 56312 GAGAAGCTGCTGCCAATACC CTCTGCTTCTTGCTCCCATC

*trib3* 13198 CCGAGAGCTGCTCAGTTAGG CTCTGCTTCTTGCTCCCATC

*vcam1* 22329 TCTCTCAGGAAATGCCATCC GGCTCAGGCTCATCTCTCAC

*scd2* 13076 GCTCTTCCTTCTATGCTAAGC ACGTCAGAACAACCGAATCC

*cyp1a1* 20250 GAAAGTGCATCGGAGAGACC GTTGGTGTACTCTGGAAGGTG

*abcd2* 26874 TTATGTGAGCCAAGCAGTGG AAGCCTGAAGATGCTGAGGA

*hprt1* 15452 GTAATGATCGTCAACGGGGGAC CAGTGCTCACCTCTAACGAG
